# A revision of dragon millipedes I: genus *Desmoxytes* Chamberlin, 1923, with the description of eight new species (Diplopoda, Polydesmida, Paradoxosomatidae)

**DOI:** 10.3897/zookeys.761.24214

**Published:** 2018-05-29

**Authors:** Ruttapon Srisonchai, Henrik Enghoff, Natdanai Likhitrakarn, Somsak Panha

**Affiliations:** 1 Biological Sciences Program, Faculty of Science, Chulalongkorn University, Phaya Thai Road, Patumwan, Bangkok 10330, Thailand; 2 Animal Systematics Research Unit, Department of Biology, Faculty of Science, Chulalongkorn University, Phayathai Road, Patumwan, Bangkok 10330, Thailand; 3 Natural History Museum of Denmark, University of Copenhagen, Universitetsparken 15, DK-2100 København Ø, Denmark; 4 Division of Plant Protection, Faculty of Agricultural Production, Maejo University, San Sai, Chiang Mai 50290, Thailand

**Keywords:** aposematic, dragon millipede, new species, Southeast Asia, taxonomy

## Abstract

The dragon millipede genus *Desmoxytes* s.l. is split into five genera, based on morphological characters and preliminary molecular phylogenetic analyses. The present article includes a review of *Desmoxytes* s.s., while future articles will deal with *Hylomus* Cook and Loomis, 1924 and three new genera which preliminarily are referred to as the ‘*acantherpestes*’, ‘*gigas*’, and ‘*spiny*’ groups. Diagnostic morphological characters of each group are discussed. *Hylomus* is resurrected as a valid genus and the following 33 species are assigned to it: *H. asper* (Attems, 1937), **comb. n.**, *H. cattienensis* (Nguyen, Golovatch & Anichkin, 2005), **comb. n.**, *H. cervarius* (Attems, 1953), **comb. n.**, *H. cornutus* (Zhang & Li, 1982), **comb. n.**, *H. draco* Cook & Loomis, 1924, **stat. rev.**, *H. enghoffi* (Nguyen, Golovatch & Anichkin, 2005), **comb. n.**, *H. eupterygotus* (Golovatch, Li, Liu & Geoffroy, 2012), **comb. n.**, *H. getuhensis* (Liu, Golovatch & Tian, 2014), **comb. n.**, *H. grandis* (Golovatch, VandenSpiegel & Semenyuk, 2016), **comb. n.**, *H. hostilis* (Golovatch & Enghoff, 1994), **comb. n.**, *H. jeekeli* (Golovatch & Enghoff, 1994), **comb. n.**, *H. lingulatus* (Liu, Golovatch & Tian, 2014), **comb. n.**, *H. laticollis* (Liu, Golovatch & Tian, 2016), **comb. n.**, *H. longispinus* (Loksa, 1960), **comb. n.**, *H. lui* (Golovatch, Li, Liu & Geoffroy, 2012), **comb. n.**, *H. minutuberculus* (Zhang, 1986), **comb. n.**, *H. nodulosus* (Liu, Golovatch & Tian, 2014), **comb. n.**, *H. parvulus* (Liu, Golovatch & Tian, 2014), **comb. n.**, *H. phasmoides* (Liu, Golovatch & Tian, 2016), **comb. n.**, *H. pilosus* (Attems, 1937), **comb. n.**, *H. proximus* (Nguyen, Golovatch & Anichkin, 2005), **comb. n.**, *H. rhinoceros* (Likhitrakarn, Golovatch & Panha, 2015), **comb. n.**, *H. rhinoparvus* (Likhitrakarn, Golovatch & Panha, 2015), **comb. n.**, *H. scolopendroides* (Golovatch, Geoffroy & Mauriès, 2010), **comb. n.**, *H. scutigeroides* (Golovatch, Geoffroy & Mauriès, 2010), **comb. n.**, *H. similis* (Liu, Golovatch & Tian, 2016), **comb. n.**, *H. simplex* (Golovatch, VandenSpiegel & Semenyuk, 2016), **comb. n.**, *H. simplipodus* (Liu, Golovatch & Tian, 2016), **comb. n.**, *H. specialis* (Nguyen, Golovatch & Anichkin, 2005), **comb. n.**, *H. spectabilis* (Attems, 1937), **comb. n.**, *H. spinitergus* (Liu, Golovatch & Tian, 2016), **comb. n.**, *H. spinissimus* (Golovatch, Li, Liu & Geoffroy, 2012), **comb. n.** and *H. variabilis* (Liu, Golovatch & Tian, 2016), **comb. n.** *Desmoxytes* s.s. includes the following species: *D. breviverpa* Srisonchai, Enghoff & Panha, 2016; *D. cervina* (Pocock,1895); *D. delfae* (Jeekel, 1964); *D. des* Srisonchai, Enghoff & Panha, 2016; *D. pinnasquali* Srisonchai, Enghoff & Panha, 2016; *D. planata* (Pocock, 1895); *D. purpurosea* Enghoff, Sutcharit & Panha, 2007; *D. takensis* Srisonchai, Enghoff & Panha, 2016; *D. taurina* (Pocock, 1895); *D. terae* (Jeekel, 1964), all of which are re-described based mainly on type material. Two new synonyms are proposed: *Desmoxytes pterygota* Golovatch & Enghoff, 1994, **syn. n.** (= *Desmoxytes cervina* (Pocock, 1895)), *Desmoxytes rubra* Golovatch & Enghoff, 1994, **syn. n.** (= *Desmoxytes delfae* (Jeekel, 1964)). Six new species are described from Thailand: *D. aurata* Srisonchai, Enghoff & Panha, **sp. n.**, *D. corythosaurus* Srisonchai, Enghoff & Panha, **sp. n.**, *D. euros* Srisonchai, Enghoff & Panha, **sp. n.**, *D. flabella* Srisonchai, Enghoff & Panha, **sp. n.**, *D. golovatchi* Srisonchai, Enghoff & Panha, **sp. n.**, *D. octoconigera* Srisonchai, Enghoff & Panha, **sp. n.**, as well as one from Malaysia: *D. perakensis* Srisonchai, Enghoff & Panha, **sp. n.**, and one from Myanmar: *D. waepyanensis* Srisonchai, Enghoff & Panha, **sp. n.** The species can mostly be easily distinguished by gonopod structure in combination with other external characters; some cases of particularly similar congeners are discussed. All species of *Desmoxytes* s.s. seem to be endemic to continental Southeast Asia (except the ‘tramp’ species *D. planata*). Some biological observations (relationship with mites, moulting) are recorded for the first time. Complete illustrations of external morphological characters, an identification key, and distribution maps of all species are provided.

## Introduction

The dragon millipede genus *Desmoxytes* Chamberlin, 1923 is one of the most spectacular genera in the large family Paradoxosomatidae. The genus currently contains 45 described species which are broadly distributed in China, Laos, Malaysia, Myanmar, Thailand, and Vietnam. The “tramp” species *D. planata* (Pocock, 1895) has become widely dispersed to Fiji, French Polynesia, India, Indonesia, Seychelles, and Sri Lanka (Attems 1936, Chamberlin 1923, 1941, Golovatch and Korsós 1992, Jeekel 1980b, Mauriès 1980, Ramage 2017, Shelley and Lehtinen 1998, Tikader and Das 1985). Most species are best represented in or even restricted to limestone habitats and caves (Enghoff et al. 2007, Liu et al. 2014, 2016, Srisonchai et al. 2016), and some are probably troglobites (Loksa 1960, Golovatch et al. 2010, 2012, Liu et al. 2014, 2016).

*Desmoxytes* has been taxonomically discussed on several occasions (Jeekel 1964, 1980a, Golovatch and Enghoff 1994, Golovatch et al. 2012). The genus was revised by Golovatch and Enghoff (1994), and further variable gonopod characters were added to the diagnosis by Golovatch et al. (2012). Several new species were described recently (Liu et al. 2014, 2016, Likhitrakarn et al. 2015, Golovatch et al. 2016, Srisonchai et al. 2016). However, for the time being, no updated diagnosis exists for *Desmoxytes*.

Intensive field surveys focusing on this genus were made by our team (ASRU), mainly in Southeast Asia (Malaysia, Myanmar, Laos, Thailand). After examination of newly collected specimens, and comparison with type material of all congeners, we found distinctive morphological characters, mainly in gonopods and paraterga, indicating heterogeneity of *Desmoxytes* s.s. A preliminary study on phylogeny of dragon millipedes based on mtDNA and nuclear DNA shows a perfect congruence with morphology (Srisonchai et al. 2017) and further indicates that *Desmoxytes* as hitherto understood (i. e., *sensu* Golovatch and Enghoff 1994) is not a monophyletic taxon. Therefore, we find it necessary to subdivide the dragon millipedes into five genera. In the present study, the first in a series of articles about dragon millipedes, *Desmoxytes* is re-diagnosed, and the four other genera (*Hylomus* Cook & Loomis 1924, and three new genera yet to be named) are outlined. Ten species of *Desmoxytes* in the new, restricted sense are revised, eight new species are described, a new identification key to *Desmoxytes* species is provided, and 33 *Desmoxytes* species are assigned to the reinstated genus *Hylomus*.

## Materials and methods

### Specimen collecting and preservation

The specimens were collected by hand from different localities in China, Laos, Malaysia, Myanmar, and Thailand during the rainy season. The GPS coordinates were recorded by using the Garmin GPSMAP 60CSx and the elevation was obtained by checking in Google Earth.

Intensive surveys in several parts of those countries, especially in Thailand, have been made since 2007 by staff and students from Animal Systematics Research Unit, Department of Biology, Faculty of Science, Chulalongkorn University, referred to as “ASRU members” in the lists of material. Specimens were preserved in 70% ethanol for morphological study and partly in 95% ethanol for molecular study.

### Illustrations

Photos of living specimens were taken in the field and after collecting using a Nikon 700D+AFS VR 105 mm lens. The gonopods were illustrated with a scanning electron microscope (JEOL, JSM–5410 LV); gonopods were coated with gold and mounted on aluminium stubs, and after imaging the gonopod was removed from the stub to be kept in dry condition. Drawings were made using dot-line techniques under a stereo microscope.

### Morphological descriptions

General descriptions of the tribe and of the genus are provided. All specimens were carefully examined for non-gonopodal (male, female and juvenile) and gonopodal characters under a stereo microscope. Non-gonopodal characters were examined those of size, colour, head, antenna, collum, tegument, prozona, metaterga, paraterga, telson, sterna and legs. We use the terms of gonopod morphology from previous papers (Pocock 1895, Chamberlin 1923, 1941, Attems 1937, Jeekel 1964, 1980a, Zhang 1986, Jeekel 2003, Golovatch and Enghoff 1994, Enghoff et al. 2007, Srisonchai et al. 2016), in part adapted, and we add further morphological characters (see Table 1).

**Table 1.** Gonopod structures in *Desmoxytes* s.s., and their abbreviations (in **Bold**: structure only occurring in certain species).

Gonopodal part	Abb.	Description
Acropodite		The apical part of the gonopod; including femorite, solenophore, and solenomere.
Coxa	cx	The basal part of the gonopod, connecting to body ring, attached to the apertural rim dorsally; rather stout; ca. half as long as femur, sometimes quite short (equal in length with prefemur); with distoanterior group of setae.
Broad lobe of lamina medialis	blm	A broad lobe originating from lamina medialis, lamella-like; normally broad with thick edge.
Cannula	ca	A short tube, lever-like, curved, long and slender; originating from coxa, tip inserted into concavity in prefemur.
Distal lobe of lamina medialis	dlm	A lamella-like process, situated on the top of lamina medialis, consisting one or two small lobe(s)/ lamella(e).
Femur	fe	The longest part of the gonopod, straight; accommodates the seminal groove.
Lamina medialis	lm	A large part distally on the gonopod, normally with one process and one/two lobe(s)
Lamina lateralis	ll	A distinct lobe in the distal part of gonopod; sometimes comprising a ridge and/or lobe
Lateral sulcus	ls	A distinct sulcus distally on femur, visible in lateral view
Mesal sulcus	ms	A distinct sulcus distally on femur, usually seen in mesal view.
Postfemur	pof	A short part of telopodite, supporting solenophore and solenomere, demarcated from femur by lateral sulcus and mesal sulcus.
Prefemur	pfe	The basal portion of the telopodite, with densely setose.
Process of lamina medialis	plm	A protruding process originating from lamina medialis
Seminal groove	sg	A conspicuous groove, similar to a tunnel, seen as a transparent line, visible on femur in mesal view.
Solenomere	sl	A usually long, flagella-like appendage, originating on base of solenophore.
Solenophore (= tibiotarsus)	sph	Apical part of telopodite, consisting of lamina lateralis and lamina medialis.
Telopodite		The main part of the gonopod pivoting on coxa; including prefemur, femorite, solenophore, and solenomere.
**Ventral lobe of lamina lateralis**	**vll**	An additional lobe on lamina lateralis, normally digitiform, visible in ventral view, seen in *D. purpurosea, D. breviverpa, D. takensis, D. waepyanensis* sp. n.
**Ventral ridge of lamina lateralis**	**vrl**	An additional ridge on lamina lateralis, seen in ventral view, present in *D. aurata* sp. n., *D. cervina*, *D. delfae*, *D. euros* sp. n., *D. flabella* sp. n., *D. perakensis* sp. n., *D. planata*.

### Deposition of holotypes, paratypes and other new specimens

All holotypes, some paratypes of the new species, and most additional specimens will be deposited at CUMZ. Some paratypes and some new specimens will be donated to NHMUK, NHMW, ZMUC, and ZMUM.

**Figure 1.**
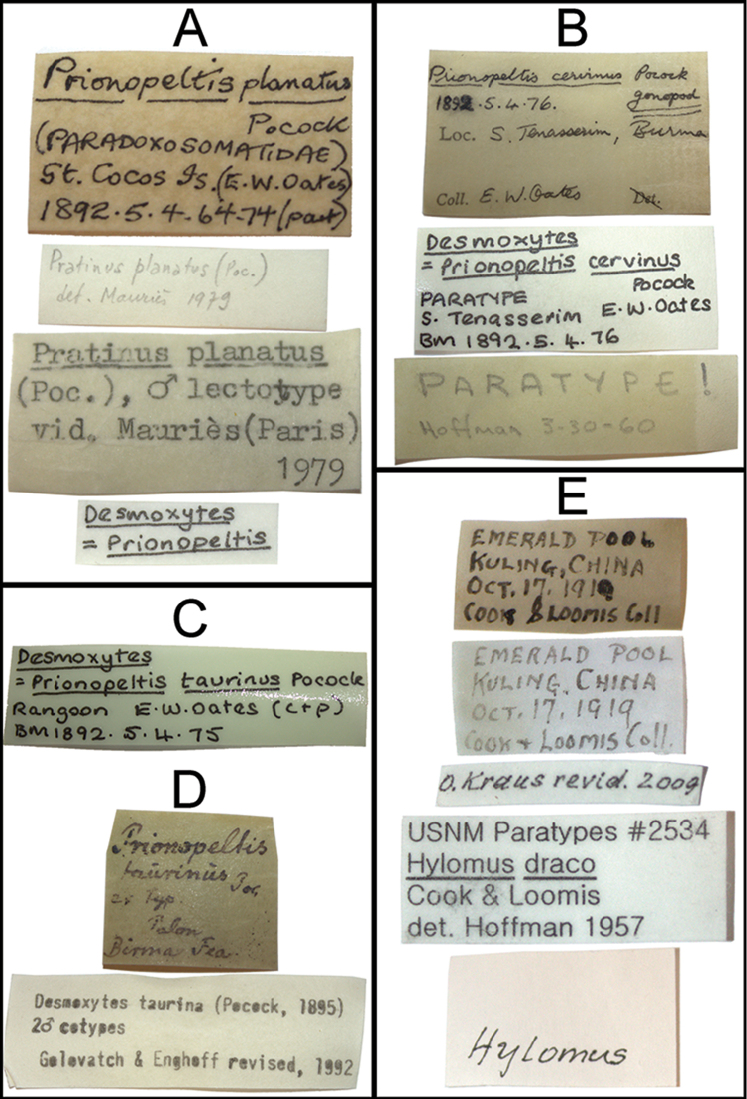
Some original and additional labels of some lectotypes and paratypes. **A** *Desmoxytes planata* (Pocock, 1895) (NHMUK) **B** *D. cervina* (Pocock, 1895) (NHMUK) **C** *D. taurina* (Pocock, 1895) (NHMUK) **D** *D. taurina* (Pocock, 1895) (ZMUC) **D** *Hylomus draco* Cook & Loomis, 1924 (USNM).

All species of *Desmoxytes* s.s. have been examined, notably Pocock’s specimens in NHMUK, type material of *Hylomus draco* in USNM, and some material in ZMUC. Figure 1 shows original labels of the type material for *Desmoxytes planata*, *D. cervina*, *D. taurina*, and *Hylomus draco*.

### Abbreviation of institutions

**ASRU** Animal Systematics Research Unit, Department of Biology, Faculty of Science, Chulalongkorn University, Bangkok, Thailand

**CUMZ** Chulalongkorn University Museum of Zoology, Bangkok, Thailand

**MCZ** Museum of Comparative Zoology, Harvard University, USA

**MHNG** Natural History Museum of Geneva, Switzerland

**MSNG** Museo Civico di Storia Naturale, Genova, Italy

**NBC** Naturalis Biodiversity Center (including the collections of the Zoological Museum Amsterdam), Leiden, the Netherlands

**NHMUK** Natural History Museum of London, England

**NHMW** Natural History Museum Vienna, Austria

**USNM** National Museum of Natural History, Smithsonian Institution, Washington DC, USA

**ZMH** Biozentrum Grindel und Zoologisches Museum, University of Hamburg, Germany

**ZMUC** Natural History Museum of Denmark (Zoological Museum), University of Copenhagen, Denmark

**ZMUM** Zoological Museum, University of Moscow, Russia

### Other abbreviations used in the text

**a.s.l.** above sea level

**ca.** approximately, around (circa)

**FFI** Fauna and Flora International, Myanmar

### Positional/directional terms in gonopod description

Morphologically the gonopods are traditionally depicted as rotated 90° up from their position *in situ*.

*Dorsal* refers to a position on the side nearest to the body ring. *Ventral* refers to a position on the side farthest away from the body ring. *Mesal* refers to a position on the side nearest to the midline. *Lateral* refers to a position on the side away from the midline.

*Dorsad* refers to a direction towards the body ring. *Ventrad* refers to a direction away from the body ring. *Mesad* refers to a direction towards the midline. *Laterad* refers to a direction away from the midline.

“Sub-” is used as a prefix to indicate positions and directions slightly different from the ones given above. For example, “submesal” means a position close to, but not quite on the mesal side.

## Results

### Taxonomy

#### Class Diplopoda Blainville-Gervais, 1844

##### Order Polydesmida Pocock, 1887

###### Family Paradoxosomatidae Daday, 1889

####### Subfamily Paradoxosomatinae Daday, 1889

######## Tribe Orthomorphini Brölemann, 1916

The tribe Orthomorphini was established by Brölemann (1916), as belonging to family Strongylosomidae. Later, the correct name for this family was found to be Paradoxosomatidae (Jeekel 1963). The tribe Orthomorphini is characterised by the following gonopodal characters:

1. Femur demarcated from postfemur by distinct constriction (in most species).

2. Seminal groove running along mesal side of femur/femorite.

3. Apical part of telopodite consisting of a solenophore supporting the solenomere. The solenophore consists of a lamina lateralis and a lamina medialis.

4. Most species without a femoral process and a tibiotarsal process.

The tribe Orthomorphini currently contains 22 genera (Nguyen and Sierwald 2013). Around 200 named species have been recorded so far. The tribe is broadly distributed mainly in Southeast Asia (Cambodia, Indonesia, Laos, Malaysia, Myanmar, Thailand, Vietnam, a few synanthropic species more or less globally widespread).

######### Subdivision of *Desmoxytes* s.l.

It is here proposed to split *Desmoxytes* s.l. into five groups. These groups are morphologically distinct, as detailed below, and are all supported by a molecular phylogeny (work ongoing). The molecular phylogenetic tree yields five groups of *Desmoxytes* s.l., and *Orthomorpha* Bollman, 1893 and *Antheromorpha* Jeekel, 1968 appear as ingroups. *Desmoxytes* s.l. thus comes out as non-monophyletic (Srisonchai et al. 2017).

The present paper deals with one of the five groups, viz., *Desmoxytes* s.s. The remaining species (see Table 2) will be assigned to their proper genus in forthcoming papers. For one of the groups, a genus name is already available, viz, *Hylomus* Cook & Loomis, 1924, and this name is herewith re-instated as a valid genus name.

**Table 2.** Species assigned to *Desmoxytes* s.l. and their placement according to our analysis.

	Placement
1	*Desmoxytes acantherpestes* Golovatch & Enghoff, 1994 (to be placed in new genus)
2	*Desmoxytes aspera* (Attems, 1937) = *Hylomus asper* (Attems, 1937), comb. n.
3	*Desmoxytes breviverpa* Srisonchai, Enghoff & Panha, 2016 (in *Desmoxytes* s.s.)
4	*Desmoxytes cattienensis* Nguyen, Golovatch & Anichkin, 2005 = *Hylomus cattienensis* (Nguyen, Golovatch & Anichkin, 2005), comb. n.
5	*Desmoxytes cervaria* (Attems, 1953) = *Hylomus cervarius* (Attems, 1953), comb. n.
6	*Desmoxytes cervina* (Pocock, 1895) (in *Desmoxytes* s.s.)
7	*Desmoxytes cornuta* (Zhang & Li, 1982) = *Hylomus cornutus* (Zhang & Li, 1982), comb. n.
8	*Desmoxytes delfae* (Jeekel, 1964) (in *Desmoxytes* s.s.)
9	*Desmoxytes des* Srisonchai, Enghoff & Panha, 2016 (in *Desmoxytes* s.s.)
10	*Desmoxytes draco* (Cook & Loomis, 1924) = *Hylomus draco* Cook & Loomis, 1924, stat. rev.
11	*Desmoxytes enghoffi* Nguyen, Golovatch & Anichkin, 2005 = *Hylomus enghoffi* (Nguyen, Golovatch & Anichkin, 2005), comb. n.
12	*Desmoxytes eupterygota* Golovatch, Li, Liu & Geoffroy, 2012 = *Hylomus eupterygotus* (Golovatch, Li, Liu & Geoffroy, 2012), comb. n.
13	*Desmoxytes getuhensis* Liu, Golovatch & Tian, 2014 = *Hylomus getuhensis* (Liu, Golovatch & Tian, 2014), comb. n.
14	*Desmoxytes gigas* Golovatch & Enghoff, 1994 (to be placed in new genus)
15	*Desmoxytes grandis* Golovatch, VandenSpiegel & Semenyuk, 2016 = *Hylomus grandis* (Golovatch, VandenSpiegel & Semenyuk, 2016), comb. n.
16	*Desmoxytes hostilis* Golovatch & Enghoff, 1994 = *Hylomus hostilis* (Golovatch & Enghoff, 1994), comb. n.
17	*Desmoxytes jeekeli* Golovatch & Enghoff, 1994 = *Hylomus jeekeli* (Golovatch & Enghoff, 1994), comb. n.
18	*Desmoxytes lingulata* Liu, Golovatch & Tian, 2014 = *Hylomus lingulatus* (Liu, Golovatch & Tian, 2014), comb. n.
19	*Desmoxytes laticollis* Liu, Golovatch and Tian, 2016 = *Hylomus laticollis* (Liu, Golovatch and Tian, 2016) comb. n.
20	*Desmoxytes longispina* (Loksa, 1960) = *Hylomus longispinus* (Loksa, 1960), comb. n.
21	*Desmoxytes lui* Golovatch, Li, Liu & Geoffroy, 2012 = *Hylomus lui* (Golovatch, Li, Liu & Geoffroy, 2012), comb. n.
22	*Desmoxytes minutubercula* (Zhang, 1986) = *Hylomus minutuberculus* (Zhang, 1986), comb. n.
23	*Desmoxytes nodulosa* Liu, Golovatch & Tian, 2014 = *Hylomus nodulosus* (Liu, Golovatch & Tian, 2014), comb. n.
24	*Desmoxytes parvula* Liu, Golovatch & Tian, 2014 = *Hylomus parvulus* (Liu, Golovatch & Tian, 2014), comb. n.
25	*Desmoxytes phasmoide*s Liu, Golovatch & Tian, 2016 = *Hylomus phasmoides* (Liu, Golovatch & Tian, 2016), comb. n.
26	*Desmoxytes pilosa* (Attems, 1937) = *Hylomus pilosus* (Attems, 1937), comb. n.
27	*Desmoxytes pinnasquali* Srisonchai, Enghoff & Panha, 2016 (in *Desmoxytes* s.s.)
28	*Desmoxytes planata* (Pocock, 1895) (in *Desmoxytes* s.s.)
29	*Desmoxytes proxima* Nguyen, Golovatch & Anichkin, 2005 = *Hylomus proximus* (Nguyen, Golovatch & Anichkin, 2005), comb. n.
30	*Desmoxytes purpurosea* Enghoff, Sutcharit & Panha, 2007 (in *Desmoxytes* s.s.)
31	*Desmoxytes rhinoceros* Likhitrakarn, Golovatch & Panha, 2015 = *Hylomus rhinoceros* (Likhitrakarn, Golovatch & Panha, 2015), comb. n.
32	*Desmoxytes rhinoparva* Likhitrakarn, Golovatch & Panha, 2015 = *Hylomus rhinoparvus* (Likhitrakarn, Golovatch & Panha, 2015), comb. n.
33	*Desmoxytes scolopendroides* Golovatch, Geoffroy & Mauriès, 2010 = *Hylomus scolopendroides* (Golovatch, Geoffroy & Mauriès, 2010), comb. n.
34	*Desmoxytes scutigeroides* Golovatch, Geoffroy and Mauriès, 2010 = *Hylomus scutigeroides* (Golovatch, Geoffroy and Mauriès, 2010) comb. n.
35	*Desmoxytes similis* Liu, Golovatch & Tian, 2016 = *Hylomus similis* (Liu, Golovatch & Tian, 2016), comb. n.
36	*Desmoxytes simplex* Golovatch, VandenSpiegel & Semenyuk, 2016 = *Hylomus simplex* (Golovatch, VandenSpiegel & Semenyuk, 2016), comb. n.
37	*Desmoxytes simplipoda* Liu, Golovatch & Tian, 2016 = *Hylomus simplipodus* (Liu, Golovatch & Tian, 2016), comb. n.
38	*Desmoxytes specialis* Nguyen, Golovatch & Anichkin, 2005 = *Hylomus specialis* (Nguyen, Golovatch & Anichkin, 2005), comb. n.
39	*Desmoxytes spectabilis* (Attems, 1937) = *Hylomus spectabilis* (Attems, 1937), comb. n.
40	*Desmoxytes spiniterga* Liu, Golovatch & Tian, 2016 = *Hylomus spinitergus* (Liu, Golovatch & Tian, 2016), comb. n.
41	*Desmoxytes spinissima* Golovatch, Li, Liu & Geoffroy, 2012 = *Hylomus spinissimus* (Golovatch, Li, Liu & Geoffroy, 2012), comb. n.
42	*Desmoxytes takensis* Srisonchai, Enghoff & Panha, 2016 (in *Desmoxytes* s.s.)
43	*Desmoxytes taurina* (Pocock, 1895) (in *Desmoxytes* s.s.)
44	*Desmoxytes terae* (Jeekel, 1964) (in *Desmoxytes* s.s.)
45	*Desmoxytes variabilis* Liu, Golovatch & Tian, 2016 = *Hylomus variabilis* (Liu, Golovatch & Tian, 2016), comb. n.

**Figure 2.**
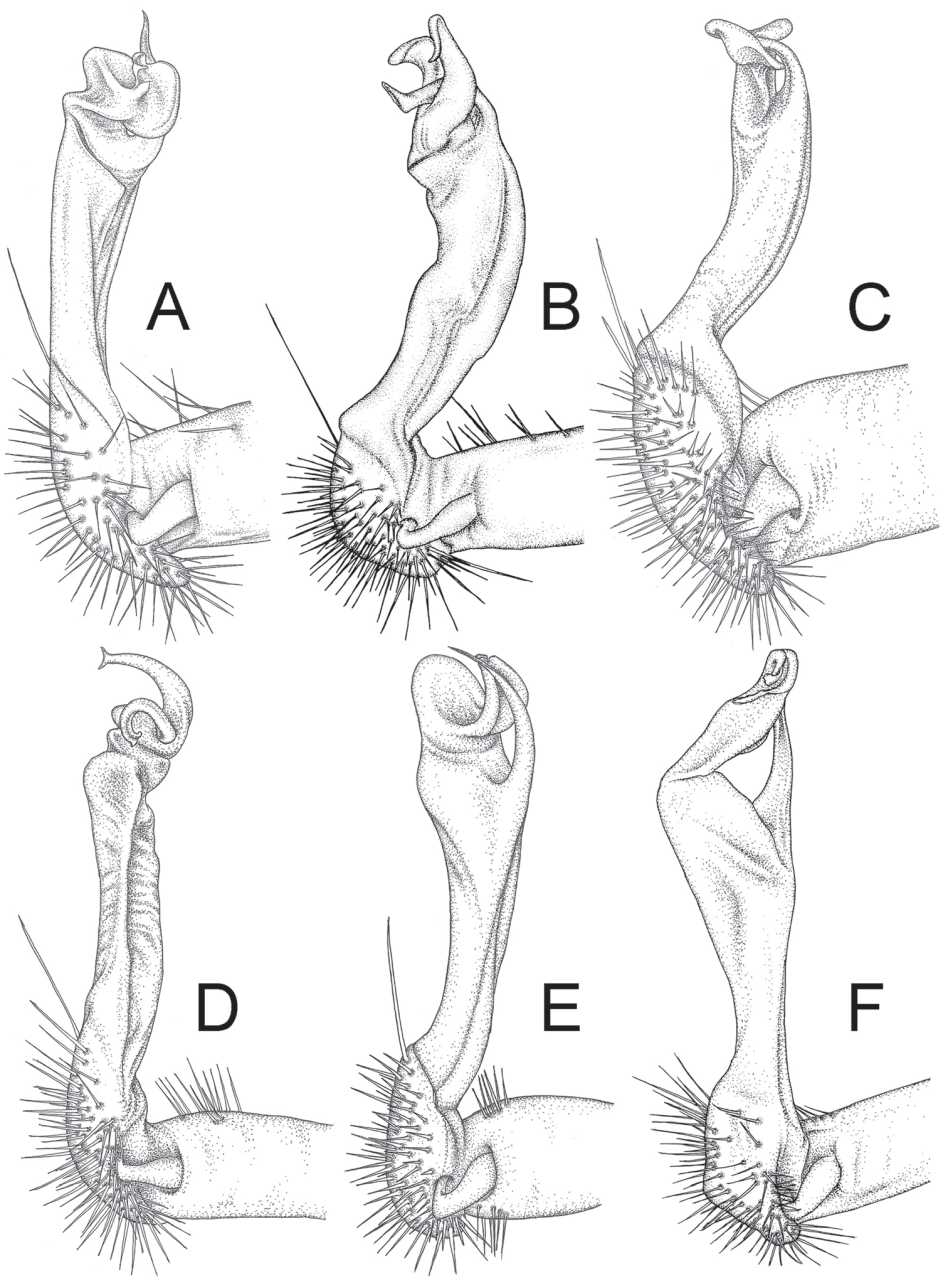
Shape of gonopod of *Desmoxytes* s.l. **A** *Desmoxytes* s.s. (*D. planata* (Pocock, 1895)) – specimen from Wat Puang Malai **B** the ‘*acantherpestes*’ group (specimen from Kanchanaburi, Thailand) **C** the ‘*gigas*’ group (specimen from Krabi, Thailand) **D** the ‘spiny’ group (specimen from Krabi, Thailand) **E, F** *Hylomus* Cook & Loomis, 1924 (**E** = *H. draco* Cook & Loomis, 1924 stat. rev. (paratype) **F** = *Hylomus* sp. (specimen from Laos)).

**Figure 3.**
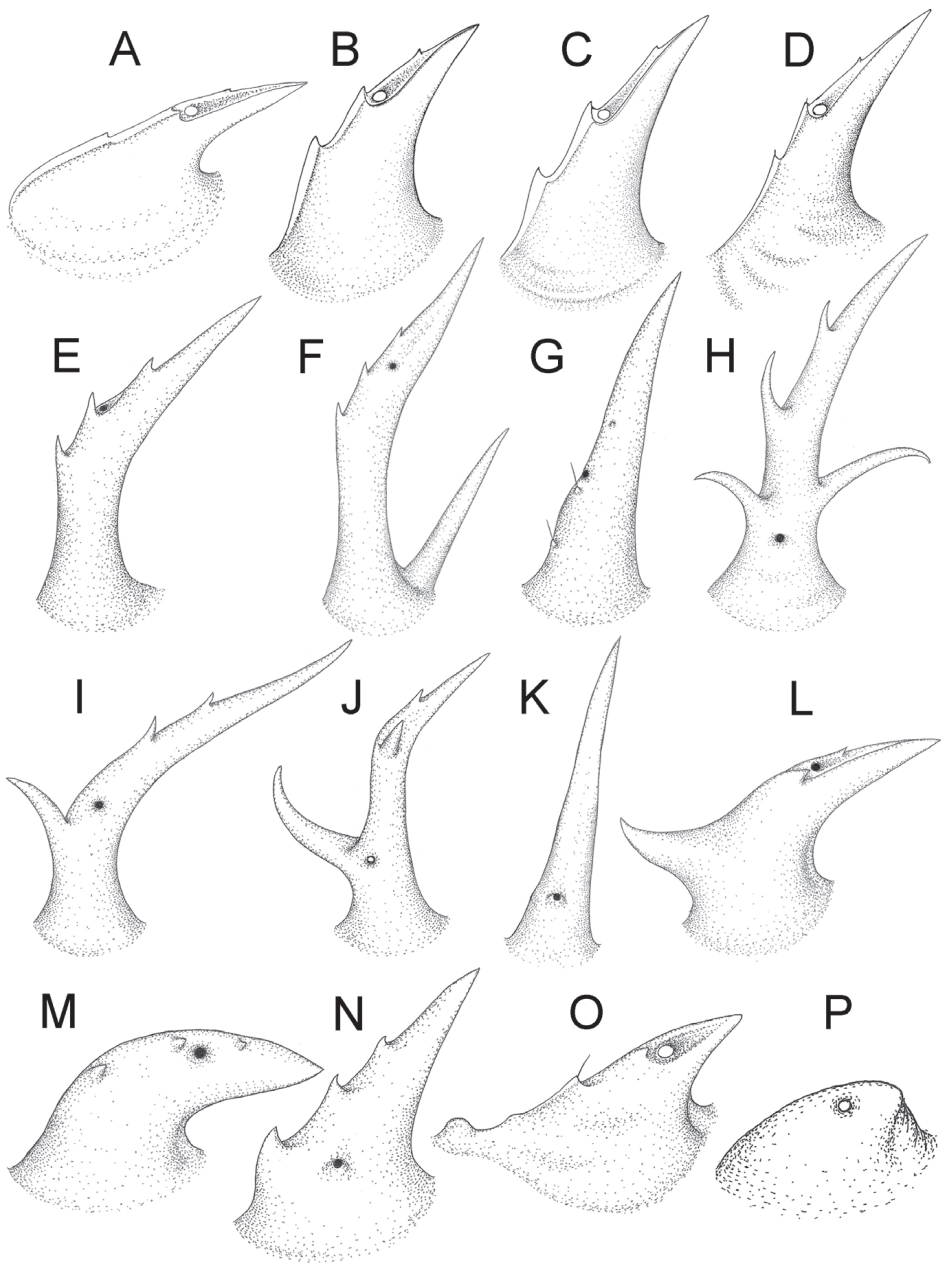
Type of paranota (paraterga) of *Desmoxytes* s.l. **A–D** *Desmoxytes* s.s. **A** *D. delfae* (Jeekel, 1964), specimen from Tham Khan Ti Phol **B** *D. planata* (Pocock, 1895), specimen from Suan Sai Thong Restaurant **C** *D. purpurosea* Enghoff et al., 2007, specimen from Hup Pa Tard **D** *D. cervina* (Pocock, 1895), specimen from Wat Satit Khirirom **E** the ‘*acantherpestes*’ group **F** the ‘*gigas*’ group **G** the ‘spiny’ group **H–P** *Hylomus* Cook & Loomis, 1924 **H** *H. draco* Cook & Loomis, 1924, stat. rev., paratype **I** *H. cervarius* (Attems, 1953), comb. n., ZMUM specimen **J** *H. rhinoceros* (Likhitrakarn et al., 2015), comb. n., paratype **K** *H. scolopendroides* (Golovatch et al., 2010), comb. n., paratype **L** *H. nodulosus* (Liu et al., 2014), comb. n., paratype **M** *H. eupterygotus* (Golovatch et al., 2012), comb. n., paratype **N** *H. scutigeroides* (Golovatch et al., 2010), comb. n., paratype **O** *H. simplex* (Golovatch et al., 2016), comb. n., paratype **P** *H. laticollis* (Liu et al., 2016), comb. n., paratype.

The five groups of *Desmoxytes* s.l. are characterised as follows (see Table 3 for all comparable characters, and Figs 2, 3):

***Desmoxytes* s.s.** (Figs 2A, 3A–D) can be easily distinguished from others by the following combination of characters: wing-shaped paraterga; male femora 5 and 6 swollen or humped (except *D. terae* without modification); lamina lateralis obviously demarcated from lamina medialis; lamina medialis and lamina lateralis equal in size, with process and lobes.

Two groups, the ‘*acantherpestes*’ group and the ‘*gigas*’ group are characterised by having subspiniform paraterga and tegument of metaterga microgranulate.

**The ‘*acantherpestes*’ group** contains species with two rows of tubercles/cones/spines on metaterga; tegument of metaterga microgranulate; gonopodal telopodite straight; postfemur conspicuous, broad laterally, demarcated from femur by a deep mesal sulcus and a deep/shallow lateral sulcus; lamina lateralis obviously demarcated from lamina medialis; lamina lateralis shorter and smaller than lamina medialis; lamina medialis long and curved (Figs 2B, 3E).

**The ‘*gigas*’ group** contain species with three rows of tubercles/cones/spines (uniform) on metaterga; tegument of metaterga microgranulate; long caudolateral spines on the metaterga; gonopodal telopodite curved, falcate; postfemur inconspicuous, mesal sulcus and lateral sulcus absent; lamina lateralis indistinctly demarcated from lamina medialis, larger than lamina medialis; lamina medialis short (Figs 2C, 3F).

**The ‘*spiny***’ **group**, still without described members, differs from the others by the following combination of characters: paraterga spiniform; tegument of metaterga smooth (some species microgranulate); gonopodal telopodite straight; postfemur conspicuous, narrow laterally, demarcated from femur by deep mesal and lateral sulci; lamina lateralis obviously demarcated from lamina medialis; lamina lateralis smaller than lamina medialis (lamina lateralis very small); lamina medialis long and curved, without process and lobe (Figs 2D, 3G).

The fifth group, corresponding to genus *Hylomus* and including numerous species, has wing-like, antler-like or spiniform paraterga, but is well-defined by gonopod characters: postfemur inconspicuous or absent (mesal and lateral sulci shallow or absent), lamina medialis short (except in a few species), lamina medialis mostly without lobe and process (except some species which have a process, a spine or a hook) (Figs 2E, F, 3H–P).

As Figs 2 and 3 clearly show, the differences in morphology (paraterga and gonopod) easily allow separation of the five groups of *Desmoxytes* s.l. species.

The distribution of each group based on data from both described and undescribed species is as follows:

• *Desmoxytes* s.s. – Malaysia, Myanmar, Thailand (includes the widely distributed ‘tramp’ species *D. planata*)

• The ‘*acantherpestes*’ group – Thailand

• The ‘*gigas*’ group – Thailand

• The ‘*spiny*’ group – Malaysia, Myanmar, Thailand

• *Hylomus* – China, Laos, Myanmar, Vietnam, Thailand

**Table 3.** Comparison of the five groups (genera) of *Desmoxytes* s.l.

	*Desmoxytes* s.s.	‘*acantherpestes*’	‘*gigas*’	‘*spiny*’	*Hylomus*
**Paraterga**	Wing-like	Subspiniform	Subspiniform	Spiniform	Wing-like, antler-like, spiniform
**Tegument (metaterga)**	Microgranulate	Microgranulate	Microgranulate	Smooth (some species microgranulate)	Microgranulate (some spp. smooth)
**Row of setae/tubercles/cones/spines on metaterga 2**–**19**	2 rows	2 rows	3 rows (uniform)	2 rows	1, 2, 3 (uniform/random), or 4 rows
**Caudolateral spine**	Absent	Absent	Present - long	Absent	Absent
**Femora 5, 6, 7, 8, 9** **Modified/Unmodified** - **If modified** - **Shape**	Modified (ex. *D. terae*)	Unmodified	Modified and unmodified	Modified and unmodified	Modified and unmodified
	5, 6		5, 6 or 5, 6, 7	6, 7 or 7 or 8, 9	5, 6 or 5, 6, 7 or 6 or 6, 7 or 6, 7, 8
	Swollen/humped		Apophysis	Apophysis	Mostly apophysis (some spp. humped, some spp. mixed - apophysis+humped)
**Pores on lobe of sternum 5**	2 pores	2 pores	1 pore	1 or 2 pores	2 pores
**Gonopod telopodite overall shape (especially femorite)**	Straight	Straight	Curved (falcate)	Straight	Curved (some spp. straight)
**Postfemoral part**	Conspicuous, broad laterally	Conspicuous, broad laterally	Inconspicuous	Conspicuous, narrow laterally	Inconspicuous
**Mesal sulcus (ms)/lateral sulcus (ls)**	ms deep, ls deep/ shallow	ms deep, ls deep/shallow	ms absent, ls absent	ms deep, ls deep	ms shallow/absent, ls shallow/absent
**Lamina lateralis (ll)**	Obviously demarcated from lm	Obviously demarcated from lm	Indistinctly demarcated from lm	Obviously demarcated from lm	Mostly - indistinctly demarcated from lm (some spp. obvious)
**ll larger/smaller than lm**	Equal size	Smaller than lm	Larger than lm	Smaller than lm (ll very small)	Mostly larger than lm (some spp. smaller than lm)
**Lamina medialis (lm)**	Short	Long, curved	Short	Long, curved	Short
**Process on Lamina medialis (lm)**	With process	Absent	Absent	Absent	Absent
**Lobe(s) on Lamina medialis (lm)**	With 1 or 2 lobe(s)	Absent	Absent	Absent	Absent (ex. some spp. with process/spine/hook)

########## Genus Hylomus

Taxon classificationAnimaliaPolydesmidaParadoxosomatidae

Cook & Loomis, 1924 stat. rev.

Hylomus Cook & Loomis, 1924: 105

########### Notes.

*Hylomus* was established as a monotypic genus by Cook and Loomis (1924), who were so impressed by the remarkable external features of the type species, *H. draco* Cook & Loomis, 1924, viz., the strongly elevated paraterga (like trees), that they placed the new species not only in a new genus but even in a new family, Hylomidae.

Jeekel (1964, 1968) maintained *Hylomus* as a valid genus, including only *H. draco*, and moved it into family Paradoxosomatidae, stating that the genus *Pratinus* Attems, 1937 (=*Desmoxytes*) was closely related to *Hylomus*.

Later, Jeekel (1980a) re-assessed the generic allocation of all members of *Hylomus*, *Desmoxytes* Chamberlin, 1923, *Pratinus* Attems, 1937, *Prionopeltis* Pocock, 1895 and *Ceylonesmus* Chamberlin, 1941, leading to the recognition of three genera: *Hylomus, Desmoxytes* (= *Prionopeltis*, *Pratinus*, *Ceylonesmus*) and *Pteroxytes* Jeekel, 1980. Jeekel (1980a) stated that the morphological characters of *Hylomus* showed clear differences from *Desmoxytes* and *Pteroxytes* (paranota antler-shaped and detail of gonopodal apex). The same author emphasised that his work was just a preliminary outline, and that “the discovery of new species … may considerably change the picture”.

Golovatch and Enghoff (1994) disagreed with Jeekel’s idea (1980a) to maintain *Hylomus* and *Pteroxytes* as separate genera. Based on new evidence from examination of old and new material, previous literature and the first cladistic analysis based on morphology, the authors synonymised *Hylomus* and *Pteroxytes* under *Desmoxytes* and assigned the genus to tribe Orthomorphini, of which they regarded Hylomini a synonym.

As mentioned above, our morphological analysis, as well as the initial molecular study, support recognition of *Hylomus* as a valid genus in agreement with Jeekel (1980a). We therefore consider *Hylomus* as a valid genus, separate from *Desmoxytes*. We further reallocate 33 species of *Desmoxytes* s.l. from China (19 species), Vietnam (ten species), Laos (three species) and Thailand (one species) to *Hylomus* (see Table 2).

########## Genus Desmoxytes

Taxon classificationAnimaliaPolydesmidaParadoxosomatidae

Chamberlin, 1923

Prionopeltis Pocock, 1895: 828 (preoccupied name). Jeekel 1980a: 652 (synonymised with Desmoxytes).
Desmoxytes Chamberlin, 1923: 165.
Hylomus Cook & Loomis, 1924: 105. Golovatch and Enghoff 1994: 46 (synonymised with Desmoxytes).
Pratinus Attems, 1937: 113 (replacement name for Prionopeltis). Jeekel 1980a: 652 (synonymised with Desmoxytes).
Ceylonesmus Chamberlin, 1941: 33. Jeekel 1980a: 652 (synonymised with Desmoxytes).
Pteroxytes Jeekel, 1980a: 655. Golovatch and Enghoff 1994: 46 (synonymised with Desmoxytes).

########### Type species

**Type species.** *Desmoxytes coniger* Chamberlin, 1923 (MCZ, USA). This species was later synonymised with *Desmoxytes planata* by Jeekel (1980a).

########### Included species

**(18).**

– *D. aurata* sp. n.

– *D. breviverpa* Srisonchai, Enghoff & Panha, 2016

– *D. cervina* (Pocock, 1895)

– *D. corythosaurus* sp. n.

– *D. delfae* (Jeekel, 1964)

– *D. des* Srisonchai, Enghoff & Panha, 2016

– *D. euros* sp. n.

– *D. flabella* sp. n.

– *D. golovatchi* sp. n.

– *D. octoconigera* sp. n.

– *D. perakensis* sp. n.

– *D. pinnasquali* Srisonchai, Enghoff & Panha, 2016

– *D. planata* (Pocock, 1895) (= *D. coniger* Chamberlin, 1923, type species)

– *D. purpurosea* Enghoff, Sutcharit & Panha, 2007

– *D. takensis* Srisonchai, Enghoff & Panha, 2016

– *D. taurina* (Pocock, 1895)

– *D. terae* (Jeekel, 1964)

– *D. waepyanensis* sp. n.

*Desmoxytes* s.l. was reviewed by Golovatch and Enghoff (1994), and the diagnosis of the genus was treated in detail again by Golovatch et al. (2012). Here we propose a restricted diagnosis for *Desmoxytes* s.s. based on morphological characters (gonopod, tegument, paraterga, metaterga, sterna, femora, epiproct, hypoproct) which have been extracted from previous taxonomic works (Pocock 1895, Chamberlin 1923, 1941, Jeekel 1964, 1980a, Golovatch and Enghoff 1994, Enghoff et al. 2007, Srisonchai et al. 2016).

########### The name “dragon millipede”.

For *Desmoxytes* *sensu* Golovatch and Enghoff (1994) we will retain the name “dragon millipede”, originally coined by Cook and Loomis (1924) for *Hylomus draco*. Golovatch et al. (2012) outlined the history of the name “dragon millipede” and further argued that *Desmoxytes philippina* Nguyen & Sierwald, 2010 (Philippine Isl.), and *Desmoxytoides hasenpuschorum* Mesibov, 2006 (Australia) have been wrongly assigned to *Desmoxytes* and “dragon millipedes”, respectively.

########### Diagnosis.

*Desmoxytes* s.s. differs from other genera of Orthomorphini by the combination of the following characters:

1. Gonopod suberect: solenophore strongly condensed; lamina lateralis developed, lobe-like, without process or spine; lamina medialis distinctly demarcated from lamina lateralis, bearing process and lobes.

2. Metaterga with 2 transverse rows of setiferous setae/ tubercles/ cones/ spines.

3. Paraterga wing-shaped, well-elevated.

4. Sternal cone between male coxae 4 present; subtrapeziform/ subquadrate/ subsemicircular/ incompletely bilobed.

5. Male femora 5 and 6 modified; swollen/humped (exception: *D. terae* without modification).

########### General description of the genus *Desmoxytes* s.s.

The description applies to adult males and females, except for the gonopods section and when “male” is specified (Figs 5, 6, 7, 8). The description hereunder is mainly based on illustrations of *D. planata*.

**Figure 4.**
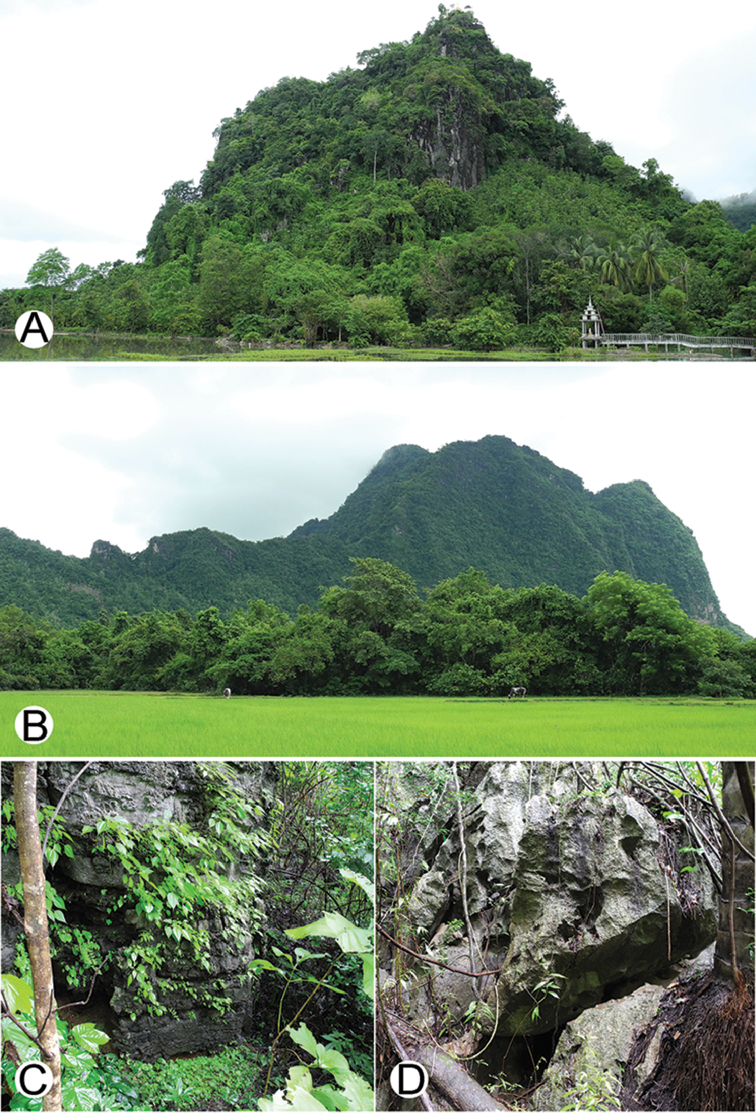
Common habitats for *Desmoxytes* Chamberlin, 1923. **A, B** limestone mountain **C** rock wall and plants **D** rock wall and small cave.

**Figure 5.**
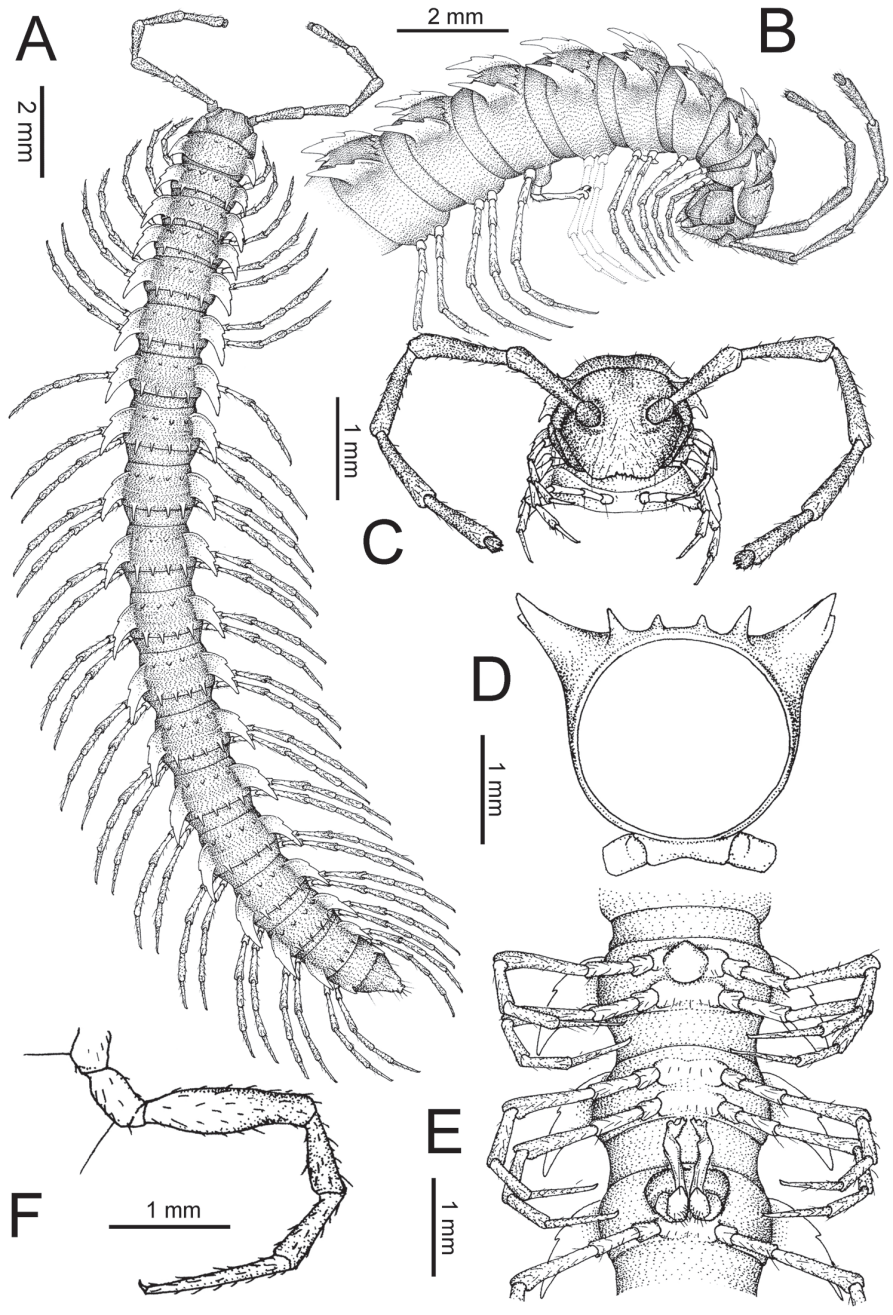
General body characters of *Desmoxytes* – *D. planata* (Pocock, 1895), male specimen from Wat Puang Malai. **A** whole body **B** anterior body part **C** head region **D** body ring **E** body rings 5–7, showing sternal lobe between coxae 4 and gonopods on ring 7 **F** male femora 5 or 6.

**Figure 6.**
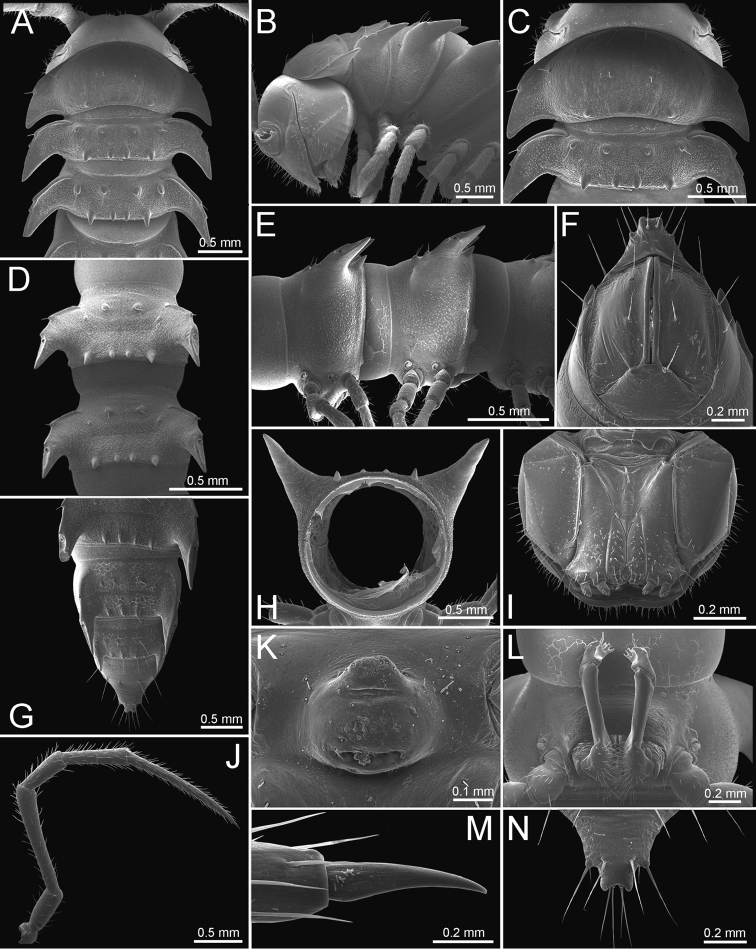
General body characters of *Desmoxytes* (*D. planata* (Pocock, 1895), male specimen from Wat Puang Malai) – SEM images. **A, B** anterior body part **C** collum **D, E** body rings 9–10 **F** last ring and telson **G** posteriormost rings **H** body ring 10 **I** mouth parts **J** leg 13 (right) **K** sternal lobe between male coxae 4 **L** gonopods on ring 7 **M** tip of tarsus and claw of leg 13 **N** tip of epiproct.

**Figure 7.**
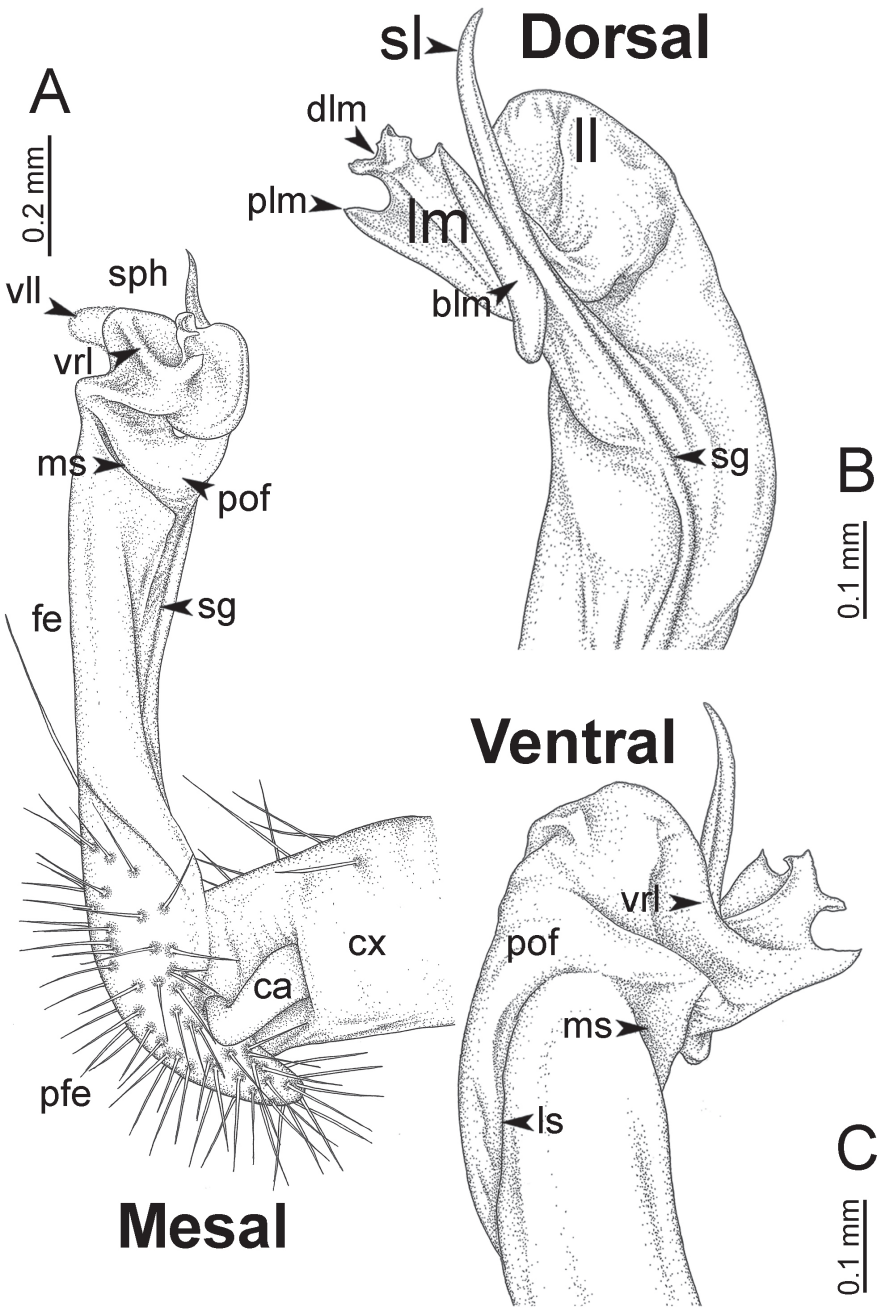
Diagrammatic drawings of right gonopod of *Desmoxytes* – *D. planata* (Pocock, 1895), specimen from Wat Puang Malai. **A** mesal view **B** dorsal view **C** ventral view.

**Figure 8.**
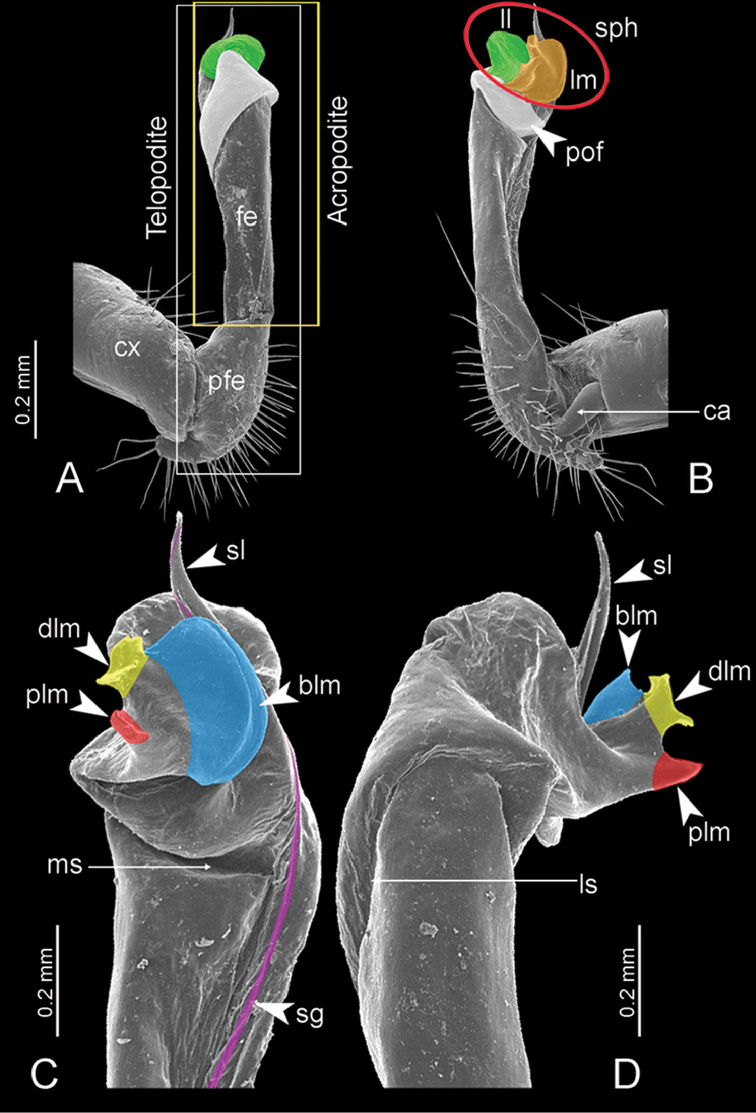
SEM images of right gonopod of *Desmoxytes planata* (Pocock, 1895), specimen from Wat Puang Malai. **A** lateral view **B** mesal view **C** submesal view **D** ventral view.

SIZE: Body length 16–35 mm (male) 20–38 mm (female), width ca. 1.7–2.5 mm (male) 2.0–3.6 mm (female); size varies between species, usually female wider and longer than male.

COLOUR: Most species in life with aposematic colouration: purplish pink, red, orange, brown, black, brownish black (piceous), brownish red (testaceous). Colour in alcohol: all specimens partly faded after one year’s preservation in alcohol; specimens kept in darkness faded more slowly.

HEAD (Figs 5C, 6A, B, I): Sparsely setose; vertex bare; labrum and genae sparsely setose; epicranial suture conspicuous as a brown or black stripe.

ANTENNAE (Fig. 5A–C): Often long and slender, rarely short, covered by delicate setation, usually reaching backwards to body ring 3–8 (male) and 3–6 (female) when stretched dorsally. Antennomere lengths 3 = 4 = 5 > 2 > 6 > 1 > 7 > 8.

COLLUM (Fig. 6A, C): With 1–3 transverse rows of setae/tubercles, the exact number in each row varying between species (sometimes lateral setae/tubercles of anterior row located nearly at the base of paraterga, on anterior margin). Paraterga wing-shaped, usually elevated at ca. 0°–30°.

TEGUMENT (Fig. 6A–E, G): Often shining, sometimes dull; collum and metaterga often microgranulate; surface below paraterga coarsely or finely microgranulate; prozona finely shagreened. Suture between prozona and metazona usually shallow, quite narrow.

METATERGA (Figs 5A, 6A, C, D, G): With 2 transverse rows of setae/tubercles/cones/spines, number in each row varying between species, lateral cones/spines of posterior row often longer and larger than inner and/or mesal ones. Suture (transverse sulcus) on metaterga present, usually conspicuous on body rings 5–17 in all species. Mid-dorsal (axial) line missing. Pleurosternal carinae forming complete, tooth-liked crests on ring 2, small ridges on ring 3, missing on remaining body rings.

PARATERGA (Figs 5A, B, D, 6A–E, G, H): Wing-shaped, well-elevated, directed caudolaterad. Callus and shoulder present, usually conspicuous, sometimes inconspicuous. Anterior margin with two distinct notches; on body rings 9, 10, 12, 13, 15–18 a tiny denticle present on lateral margin, near tip (but denticle virtually absent in some species). Elevation of paraterga in male usually higher than in female. Posterior edge concave. Tip pointed and sharp. Ozopore visible from above, round.

TELSON (Fig. 6F, G, N): Epiproct often flattened ventrally, tip often truncate or subtruncate, sometimes emarginate; lateral tubercles often conspicuous, sometimes inconspicuous; apical tubercles usually evident, sometimes indistinct, apically with two pairs of setae. Paraprocts convex. Hypoproct often subtrapeziform, sometimes subsemicircular, rarely subtriangular; caudal margin often round, sometimes emarginate, with two small setiferous tubercles.

STERNA (Figs 5E, 6K): Sparsely setose; cross-impressions faint, usually shallow, rarely quite deep. Sternal lobe between male coxae 4 usually subtrapeziform, subrectangular, subsemicircular or round, varying between species and sometimes varying between populations, usually with two pores on posterior side.

LEGS (Figs 5F, 6J, M): Very long and slender. Podomere lengths 3 = 6 > 5 > 2 = 4 > 1 > 7. Male femora 5 and 6 modified, often humped ventrally in middle part, sometimes swollen (exception: *D. terae*, without modification).

GONOPODS (Figs 6L, 7, 8): Coxa (cx) usually subequal in length to or longer than prefemur, with distoanterior group of seta. Prefemur (pfe) almost half or 2/3 as long as femur. Femur often long. Seminal groove (sg) running entirely on mesal surface of femur. Mesal sulcus (ms) conspicuous, deep; lateral sulcus (ls) conspicuous, usually deep, sometimes shallow. Postfemur shorter than femur, conspicuous. Solenophore (sph) variable between species: lamina lateralis (ll) swollen, sometimes with furrow(s) anterolaterally, sometimes with conspicuous ventral lobe (vll) or ventral ridge (vrl); lamina medialis (lm) variously species-specifically modified, consisting of one process (plm) and two lobes (distal lobe = dlm and broad lobe = blm, dlm and blm demarcated by indentation). Solenomere (sl) usually long, sometimes short.

########### Distribution and habitat.

From south China to Malaysia. Most species seem to be local endemics (only *D. planata* is dispersed, certainly by anthropochory, through mainland Southeast Asia and in many islands). *Desmoxytes* specimens were usually found by ASRU personnel in limestone habitats or on granitic mountains, and some were seen crawling on rocks or vegetation or tree branches (Figure 4).

########### Key to species of *Desmoxytes* s.s.

**Table:** 

1	Male femora 5 and 6 without modification (Fig. 90H, I)	***D. terae* (Jeekel, 1964)**
–	Male femora 5 and 6 modified (e.g., Fig. 11H, I)	**2**
2	Metaterga 2–8 with 2(1)+2(1) setae/tubercles/cones/spines in anterior row, 2+2 setae/tubercles/cones/spines in posterior row (e.g., Figs 18A, B, 38A, B). Metaterga 9–19 with 2(1)+2(1) setae/tubercles/cones/spines in anterior row, 2+2 setae/tubercles/cones/ spines in posterior row (e.g., Figs 18B, C, 38B, C)	**3**
–	Metaterga 2–8 with 2+2 or 3+3 tubercles/cones/spines in anterior row, 2+2 or 3+3 tubercles/cones/spines in posterior row (e.g., Figs 56A, B, 76A, B). Metaterga 9–19 with 2+2 or 3+3 or 4+4 tubercles/cones/spines in anterior row, 3+3 or 4(5)+4(5) tubercles/cones/spines in posterior row (e.g., Figs 56B, C, 76B, C)	**12**
3	Paraterga knife-like or blade-shaped (fig. 4B, E, H in Srisonchai et al. 2016)	***D. des* Srisonchai et al., 2016**
–	Paraterga wing-like (not knife-like or blade-shaped) (e.g., Fig. 3A–D)	**4**
4	Collum with one row of setae (anterior row) (e.g., Figs 18A, 24A, 44A). Metaterga with rows of tubercles (e.g., Fig. 18A–C). Gonopod; lamina lateralis (ll) with 1 or 2–3 conspicuous furrows (e.g., Figs 21B, D, 22F, 47B, 48E)	**5**
–	Collum with three rows of setae and/or tubercles (anterior, intermediate and posterior row) (e.g., Figs 38A, 50A, 56A). Metaterga with rows of cones/spines (e.g., Fig. 38A–C). Gonopod; lamina lateralis (ll) with an inconspicuous furrow or without furrow (e.g., Figs 41B, 53B)	**10**
5	Gonopod; lamina lateralis (ll) long, crest-like, without ventral ridge (vrl) (Fig. 27C); distal lobe (dlm) of lamina medialis long (Figs 27B–D, 28E); without or at most with inconspicuous indentation between distal lobe (dlm) and broad lobe (blm) of lamina medialis (Figs 27D, 28E)	***D. corythosaurus* sp. n.**
–	Gonopod; lamina lateralis (ll) with ventral ridge (vrl) (e.g., Figs 21C, 22C); distal lobe (dlm) of lamina medialis short (e.g., Figs 21B, 22E, 47D, 48E); with conspicuous indentation between distal lobe (dlm) and broad lobe (blm) of lamina medialis (e.g., Figs 21D, 22D, 47D, 48E)	**6**
6	Gonopod; lamina lateralis (ll) anterolaterally with two or three furrows (e.g., Figs 47B, 48E)	**7**
–	Gonopod; lamina lateralis (ll) anterolaterally with one furrow (e.g., Figs 34E, 35F)	**8**
7	Paraterga wide (Fig. 61B). Gonopod; process (plm) of lamina medialis distinctly demarcated from distal lobe (dlm) of lamina medialis (Figs 64B, C, 65C, D); distal lobe (dlm) of lamina medialis with two lamellae (Figs 64B, 65D)	***D. perakensis* sp. n.**
–	Paraterga narrow (Fig. 44B). Gonopod; process (plm) of lamina medialis indistinctly demarcated from distal lobe (dlm) of lamina medialis (Figs 47B, 48E); distal lobe (dlm) of lamina medialis with one lamella (Figs 47B, 48E)	***D. flabella* sp. n.**
8	Body brownish red or brown. Paraterga strongly elevated (40°–45°) (Fig. 18E, F)	***D. cervina* (Pocock, 1895)**
–	Body orange. Paraterga at most moderately elevated (10°–35°) (e.g., Fig. 10E, F)	**9**
9	Paraterga of collum long (Fig. 10A). Gonopod; lateral sulcus (ls) shallow (Fig. 13E); lamina lateralis (ll) compact, stout (Figs 13B, C, 14C, E); ventral ridge (vrl) of lamina lateralis short (Figs 13C, 14C); distal lobe (dlm) of lamina medialis with one lamella (Figs 13B, 14E). Sternal lobe between male coxae 4 slender when seen in lateral view (Fig. 12A, B)	***D. aurata* sp. n.**
–	Paraterga of collum short (Fig. 31A). Gonopod; lateral sulcus (ls) deep (Fig. 34E); lamina lateralis (ll) slender (Figs 34B, C, 35C, E); ventral ridge (vrl) of lamina lateralis well-developed, long, crest-like (Figs 34C, 35C); distal lobe (dlm) of lamina medialis with two lamellae (Figs 34B, 35E). Sternal lobe between male coxae 4 stout when seen in lateral view (Fig. 33A)	***D. delfae* (Jeekel, 1964)**
10	Body brownish pink. Collum with 3(4)+3(4) tubercles in anterior row (fig. 13A in Srisonchai et al 2016). Epiproct: tip extremely emarginate; apical setiferous tubercles conspicuous, very long, digitiform (fig. 13J, L in Srisonchai et al. 2016)	***D. pinnasquali* Srisonchai et al., 2016**
–	Body brown or black. Collum with 4+4 setae/tubercles in anterior row (e. g., Figs 38A, 69A). Epiproct: tip subtruncate or slightly emarginate; apical setiferous tubercles inconspicuous or conspicuous (if conspicuous – short, not digitiform) (Figs 39F, G, 70F, G)	**11**
11	Paraterga pink. Hypoproct subtrapeziform, with inconspicuous setiferous tubercles (Fig. 70C, D)	***D. planata* (Pocock, 1895)**
–	Paraterga yellow to orange. Hypoproct subtriangular, with conspicuous setiferous tubercles (Fig. 39C, D)	***D. euros* sp. n.**
12	Metaterga 9–19 with 2+2 tubercles/cones/spines in anterior row (e.g., Fig. 76B, C)	**13**
–	Metaterga 9–19 with 3+3 or 4+4 setae/tubercles/cones/spines in anterior row (e.g., Figs 50B, C, 56B, C)	**16**
13	Gonopod; lamina lateralis (ll) without ventral lobe (vll) (Fig. 87); process (plm) of lamina medialis short and thick (Fig. 87E)	***D. taurina* (Pocock, 1895)**
–	Gonopod; lamina lateralis (ll) with ventral lobe (vll) (e.g., Figs 79F, 81C); process (plm) of lamina medialis quite long and slender (Figs 15B, 79E, 83B)	**14**
14	Gonopod; ventral lobe (vll) of lamina lateralis thumb-like, large, stout (Fig. 83C, D); distal lobe (dlm) of lamina medialis with one lamella (Fig. 83B)	***D. takensis* Srisonchai et al., 2016**
–	Gonopod; ventral lobe (vll) of lamina lateralis quite long and slender, digitiform (e.g., Figs 15C, 79F); distal lobe (dlm) of lamina medialis with two lamellae (e.g., Figs 15B, 79E)	**15**
15	Gonopod; lamina lateralis (ll) more swollen (Figs 79E, 81E), surface smooth (Figs 79D, 81F); tip of process (plm) of lamina medialis terminating in several spines (Fig. 80)	***D. purpurosea* Enghoff et al., 2007**
–	Gonopod; lamina lateralis (ll) less swollen (Fig. 15B), surface rough (Fig. 15E); tip of process (plm) of lamina medialis blunt or not terminating in spines (Fig. 15B)	***D. breviverpa* Srisonchai et al., 2016**
16	Metaterga 9–19 with 3(4)+3(4) tubercles/cones/spines in posterior row (Fig. 50B, C). Gonopod; broad lobe (blm) of lamina medialis expanded dorsally (Figs 53B, 54E)	***D. golovatchi* sp. n.**
–	Metaterga 9–19 with 4(3)+4(3) or 4(5)+4(5) tubercles/cones/spines in post-erior row (Figs 56B, C, 94B, C). Gonopod; broad lobe (blm) of lamina medialis not expanded dorsally (e.g., Figs 59B, 97B)	**17**
17	Body dark brown or brown (Fig. 55A–C). Metaterga 9–19 with 4(3)+4(3) cones/spines in anterior row, 4(5)+4(5) cones/spines in posterior row (Fig. 56A–C). Male femora 5 and 6 swollen (Fig. 57H, I). Sternal lobe between male coxae 4 subtrapeziform (Fig. 58)	***D. octoconigera* sp. n.**
–	Body pinkish brown (Fig. 93A, B, D). Metaterga 9–19 with 3+3 setae/tubercles/cones in anterior row, 4(3)+4(3) setae/tubercles/cones/spines in posterior row (Fig. 94A–C). Male femora 5 and 6 strongly humped in middle portion (Fig. 95H, I). Sternal lobe between male coxae 4 incompletely bilobed (Fig. 96)	***D. waepyanensis* sp. n.**


######### Species descriptions

########## Desmoxytes aurata

Taxon classificationAnimaliaPolydesmidaParadoxosomatidae

Srisonchai, Enghoff & Panha sp. n.

http://zoobank.org/E0C412B2-9218-4B68-B925-774000C4F149

**Figure 9.**
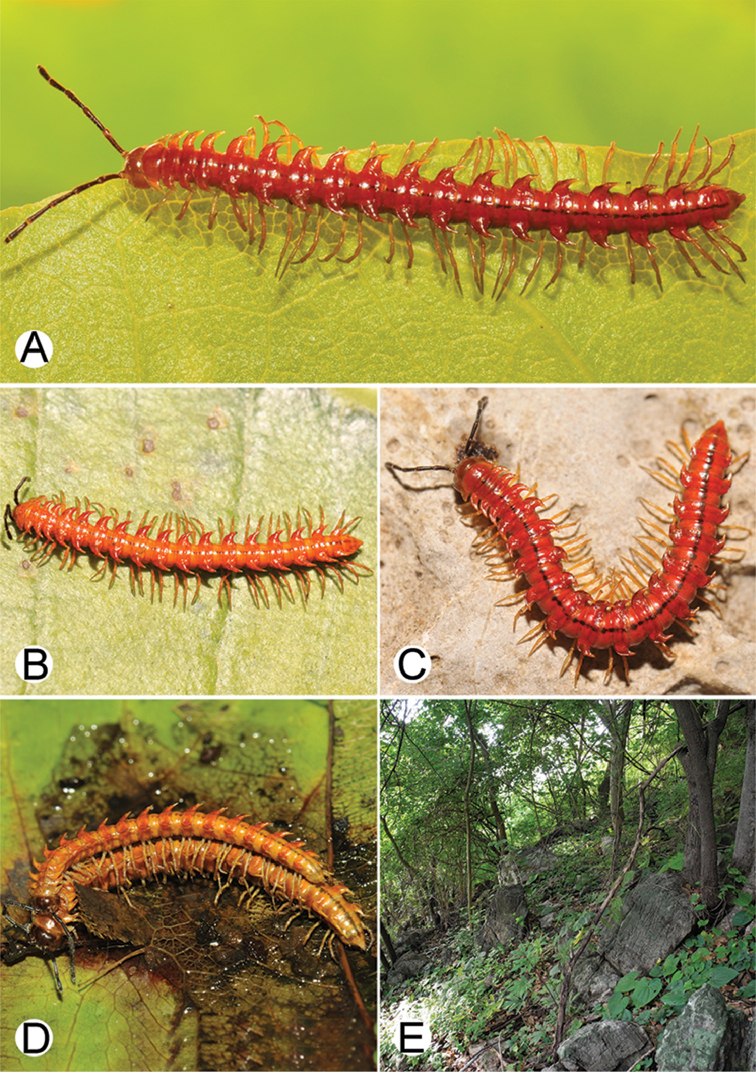
Photographs of live *Desmoxytes aurata* sp. n. and habitat. **A, B** male paratype **C** female paratype **D** mating couple **E** habitat.

**Figure 10.**
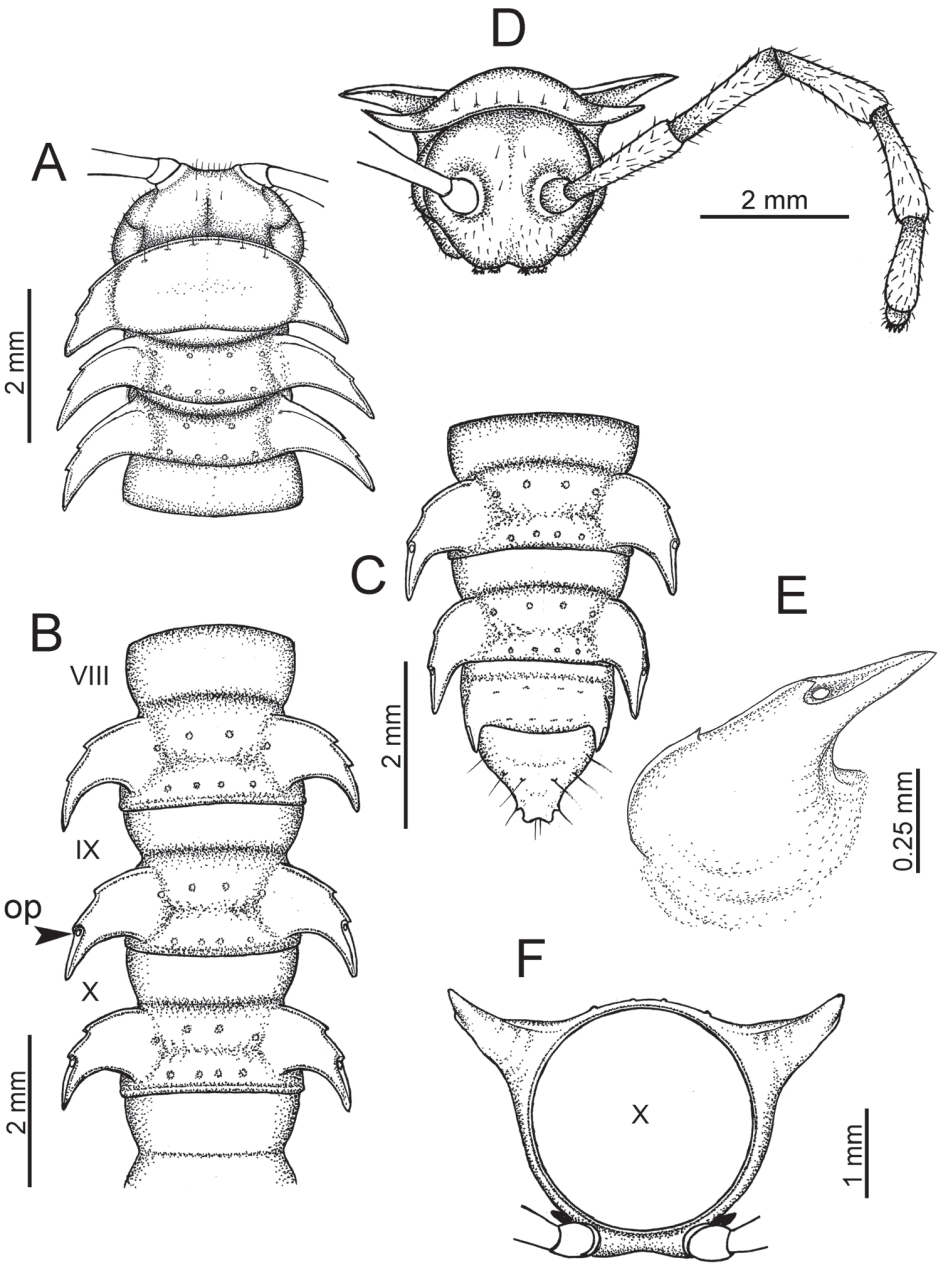
*Desmoxytes aurata* sp. n. (male paratype). **A** anterior body part **B** body rings 8–10 (op = ozopore) **C** posteriormost body rings and telson **D** head and antenna **E** paraterga of ring 10 **F** body ring 10.

**Figure 11.**
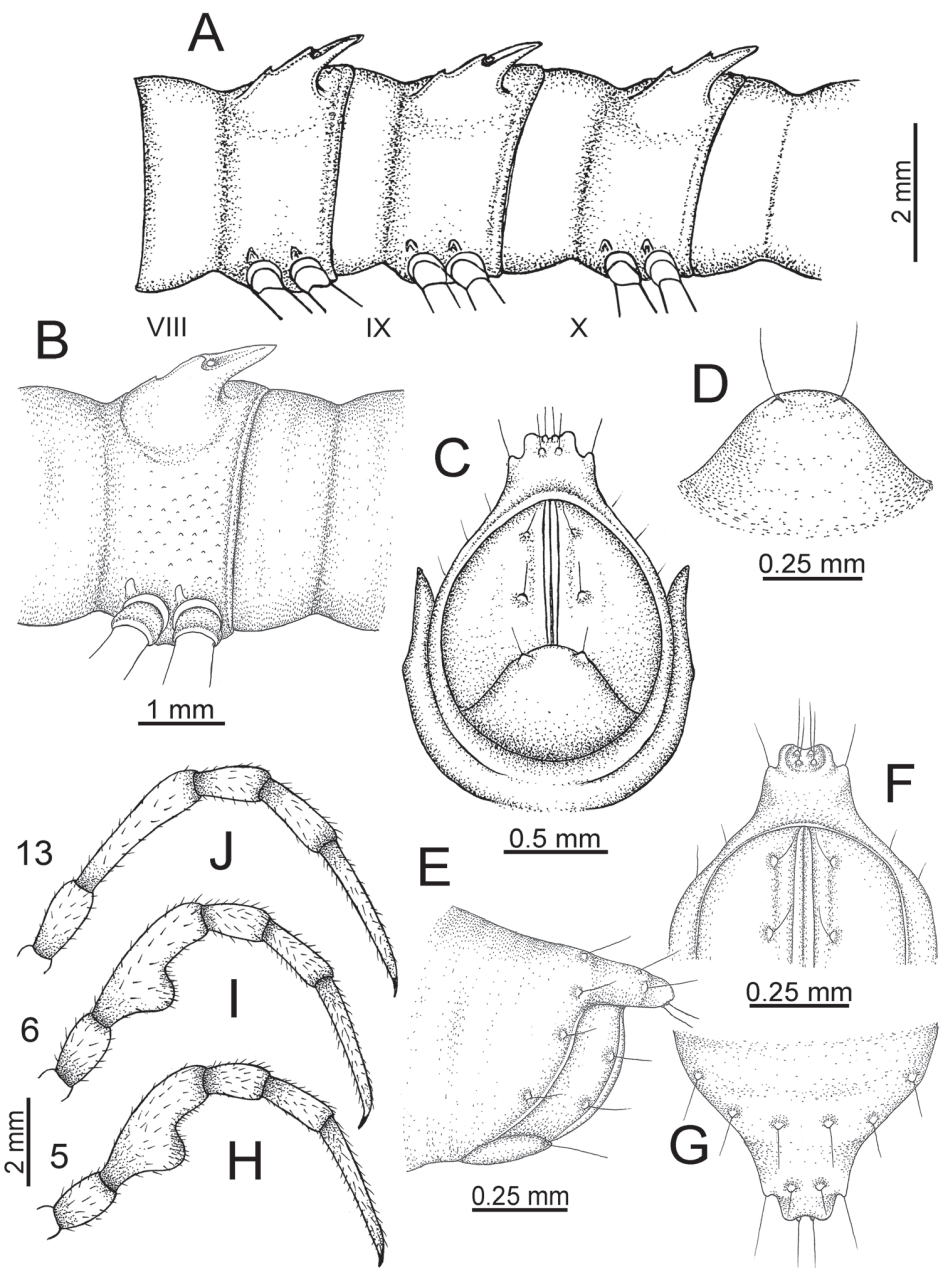
*Desmoxytes aurata* sp. n. (paratypes). **A** body rings 8–10 **B** sculpture of ring 10 **C, E** last ring and telson **D** hypoproct **F, G** epiproct **H** male leg 5 (right) **I** male leg 6 (right) **J** male leg 13 (right).

**Figure 12.**
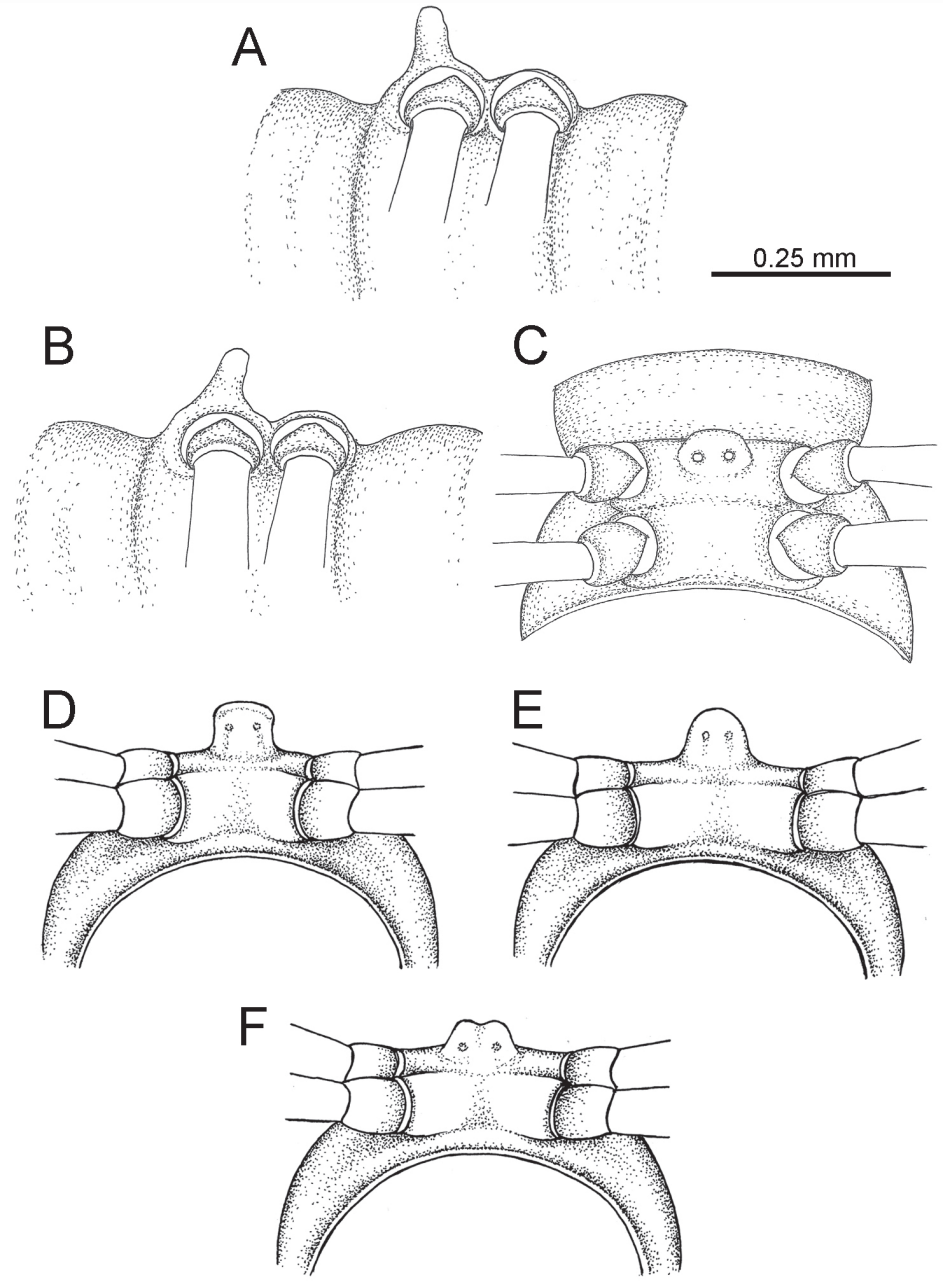
*Desmoxytes aurata* sp. n. (male holotype, male paratypes) – sternal lobe between male coxae 4. **A, B** lateral view **C** ventral view **D–F** caudal view.

**Figure 13.**
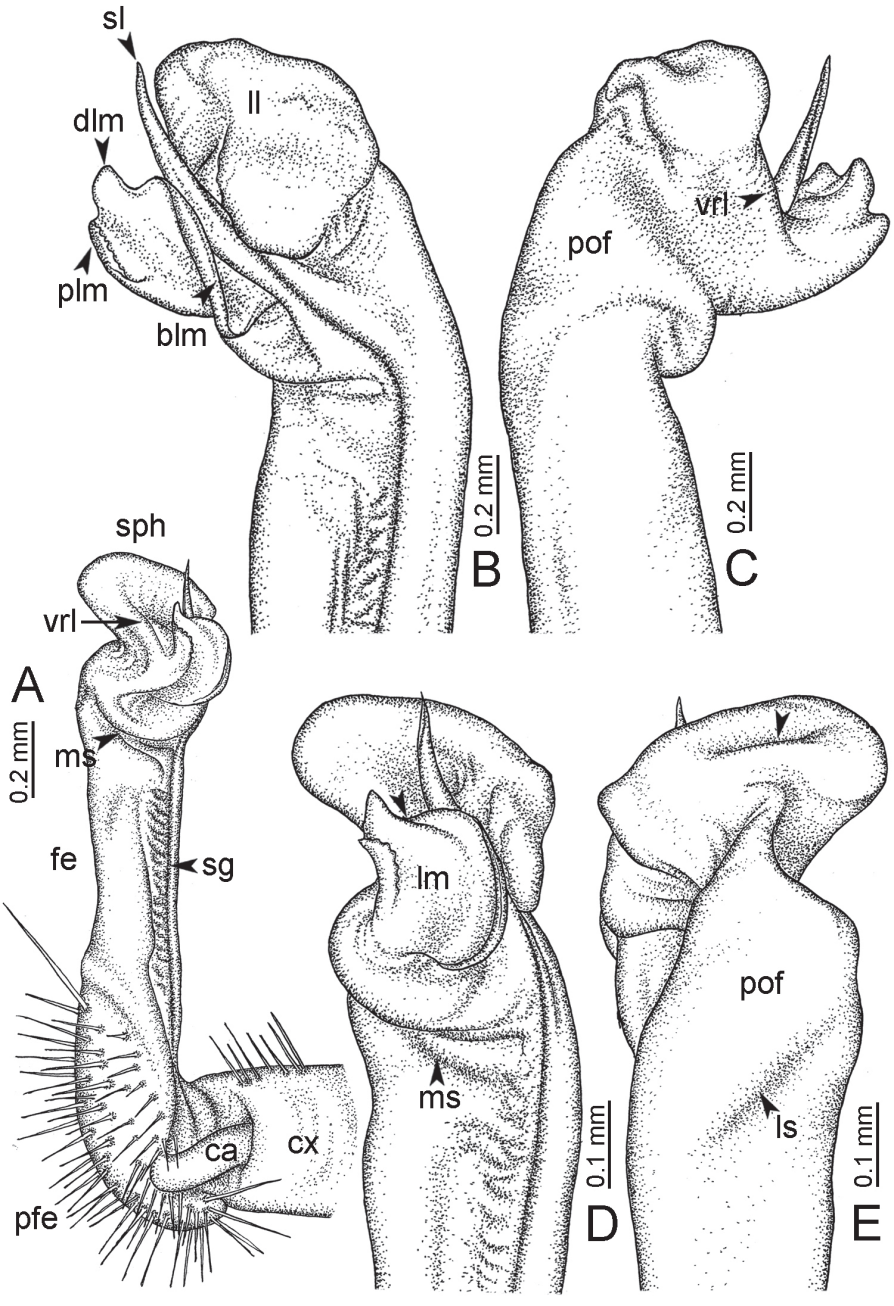
*Desmoxytes aurata* sp. n. (paratype) – right gonopod. **A** mesal view **B** dorsal view **C** ventral view **D** submesal view (arrow = indentation) **E** lateral view (arrow = furrow).

**Figure 14.**
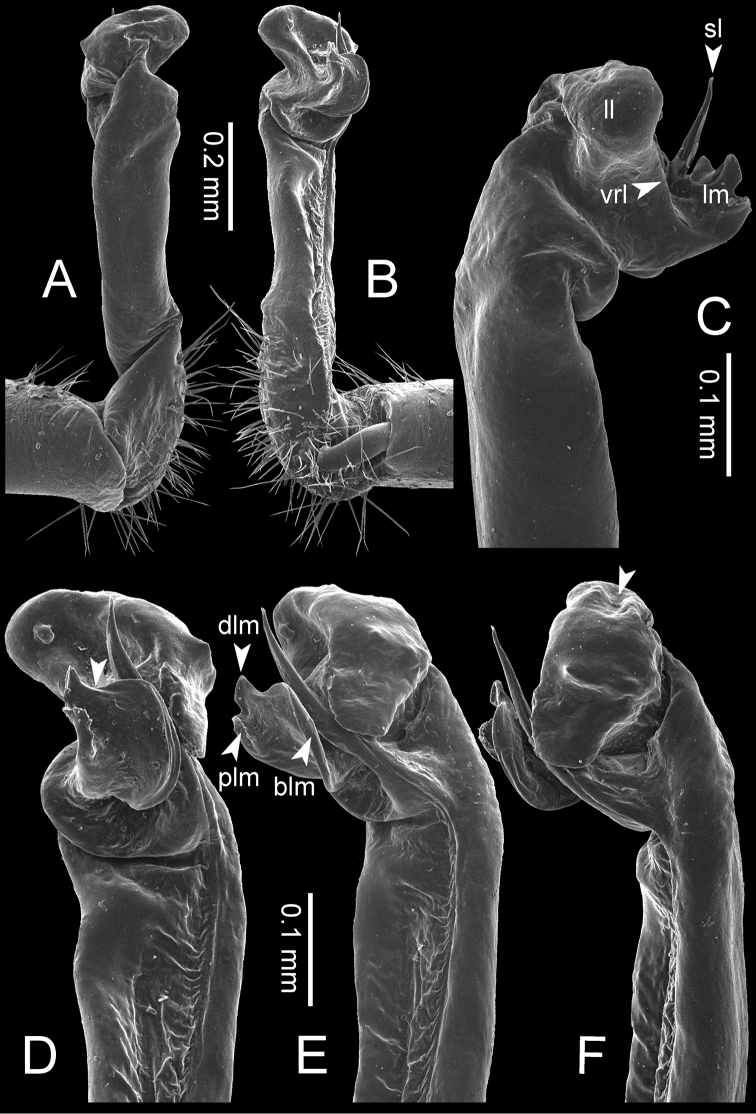
*Desmoxytes aurata* sp. n. (paratype) – right gonopod. **A** lateral view **B** mesal view **C** ventral view **D** subdorsal view (arrow = indentation) **E** dorsal view **F** subdorsal view (arrow = furrow).

Figs 9 , 10 , 11 , 12 , 13 , 14

########### Holotype.

Male (CUMZ), THAILAND, Surat Thani Province, Kanchanadit District, Khao Phanom Wang Cave, 9°05'27"N, 99°36'28"E, ca. 52 m a.s.l., 7 August 2015, leg. C. Sutcharit, R. Srisonchai, and ASRU members.

########### Paratypes.

10 males, 7 females (CUMZ), same data as holotype. 17 males, 3 females (CUMZ), 1 male, 1 female (ZMUC), 1 male (ZMUM), 1 male (NHMW), 1 male (NHMUK), THAILAND, Surat Thani Province, Kanchanadit District, Wat Praphutthabart Sri Surat, 9°11'11"N, 99°34'47"E, ca. 19 m a.s.l., 6 December 2016, leg. S. Panha and ASRU members.

**Further specimens, not paratypes, all from THAILAND, Surat Thani Province**: 8 males, 18 females, 3 broken females, 1 broken male missing right gonopod (CUMZ), Donsak District, Nang Gam Beach, limestone mountain, 9°18'53"N, 99°45'40"E, ca. 20 m a.s.l., 10 October 2008, leg. S. Panha, P. Tongkerd, and ASRU members. 7 males, 1 male missing left gonopod, 6 females, 1 male missing gonopods, 1 broken male missing left gonopod (CUMZ), Ko Samui District, Mo Ko Ang Thong National Marine Park, Ko Mae Koh, 9°39'06"N, 99°40'02"E, ca. 23 m a.s.l., 6 June 2009, leg. S. Panha and ASRU members. 1 broken male missing left gonopod, 4 males, 3 females, 1 male missing right gonopod, 1 male missing gonopods (CUMZ), Ko Samui District, Mo Ko Ang Thong National Marine Park, Ko Wua Talap, 9°38'08"N, 99°40'16"E, ca. 20 m a.s.l., 6 June 2009, leg. S. Panha and ASRU members. 1 male, 3 females (CUMZ), Donsak District, Nang Gam Beach, 9°18'53"N, 99°45'41"E, ca. 26 m a.s.l., 2 December 2015, leg. S. Panha, P. Tongkerd, and ASRU members.

**Nakhon Si Thammarat Province**: 1 male (CUMZ), Khanom District, Khao Krot Bureau of Monks, near Khao Krot Cave, 9°14'29"N, 99°48'07"E, ca. 19 m a.s.l., 23 October 2016, leg. W. Siriwut and ASRU members.

########### Diagnosis.

Body bright orange, low degree of elevation of paraterga, femora 5 and 6 strongly humped ventrally in middle part, collum with row of 3+3 anterior setae and metaterga with rows of 2+2 anterior and 2+2 posterior small tubercles. Similar in these respects to *D. delfae* and *D. perakensis* sp. n., but differs from those by having paraterga of collum quite long; lateral sulcus (ls) quite shallow; lamina lateralis (ll) stout and compact, ventral ridge (vrl) short; process (plm) of lamina medialis crenate; sternal lobe between male coxae 4 thin when seen in lateral view.

########### Etymology.

The name is Latin adjective and refers to the lamina lateralis (ll) of the gonopod which bears some resemblance to the “hooded” head of the oranda breed of goldfish (*Carassius auratus*).

########### Description.

SIZE: Length 21–24 mm (male), 25–27 mm (female); width of midbody metazona ca. 1.7 mm (male), 2.1 mm (female). Width of head < collum < body ring 2 < 3 = 4 < 5–16, thereafter body gradually tapering towards telson.

COLOUR (Fig. 9A–D): In life with body bright orange; antenna dark brown, except distal part of antennomere 7 and antennomere 8 whitish; head brown; paraterga, metaterga and surface below paraterga orange; legs, sterna and epiproct orange-ish yellow; a few basal podomeres whitish orange; prozona and metazona (metaterga) with wide black stripe, conspicuous on rings 4–19.

ANTENNAE (Fig. 10D): Moderately long and slender, reaching to body ring 6 (male) and 5 (female) when stretched dorsally.

COLLUM (Fig. 10A): With 1 transverse anterior row of 3+3 setae; paraterga of collum low, almost horizontal, directed caudolaterad, with two inconspicuous setiferous notches on lateral margin.

TEGUMENT: Strongly shining and smooth; prozona finely shagreened; collum, metaterga, sterna and epiproct smooth; surface below paraterga finely microgranulate.

METATERGA (Fig. 10A–C): With 2 transverse rows of setae and inconspicuous tubercles; metaterga 2–18 with 2+2 anterior and 2+2 posterior tubercles; metatergum 19 with 2+2 anterior and 2+2 posterior setae.

PARATERGA (Fig. 10E, F): Directed caudolaterad on body rings 2–17, elevated at ca. 30°–35° (male) 30° (female), directed increasingly caudad on body rings 18 and 19; anterior margin with 2 distinct notches, without tiny denticle near tip.

TELSON (Fig. 11C–G): Epiproct: tip emarginate; lateral setiferous tubercles small and inconspicuous; apical tubercles inconspicuous. Hypoproct subsemicircular; caudal margin round, with very small and inconspicuous setiferous tubercles.

STERNA (Fig. 12): Cross-impressions shallow. Sternal lobe between male coxae 4 subrectangular (in some specimens subtrapeziform), tip subtruncate (in some specimens subemarginate or round).

LEGS (Fig. 11H–J): Long and slender. Male femora 5 and 6 strongly humped ventrally in middle portion.

GONOPODS (Figs 13, 14): Coxa (cx) longer than prefemur. Cannula (ca) slightly stout. Telopodite quite stout. Prefemur (pfe) ca. 2/3 as long as femur. Femur (fe) somewhat stout. Mesal sulcus (ms) conspicuous and very deep; lateral sulcus (ls) shallow. Postfemur (pof) conspicuous, ventrally narrow and short. Solenophore (sph) well-developed: lamina lateralis (ll) swollen, stout and compact, anterolaterally with a distinct furrow; ventral ridge (vrl) short: lamina medialis (lm) well-developed; process (plm) short, wide and thin, tip crenate; distal lobe (dlm) distally with one lamella, tip directed anteriad; broad lobe (blm) indistinctly separated from distal lobe (dlm) by a shallow indentation. Solenomere (sl) long.

########### Distribution and habitat.

Known only from Surat Thani and Nakhon Si Thammarat Provinces. All specimens were collected in limestone mountains (on the mainland and on two islands) (Fig. 9E). We also surveyed other islands in Mo Ko Ang Thong National Marine Park, but found no specimens of *D. aurata* sp. n. The new species is probably distributed along the islands in the Gulf of Thailand and also on the mainland near the type locality. We regard it as endemic for Thailand.

*Desmoxytes aurata* sp. n. is morphologically similar to *D. delfae* and *D. perakensis* sp. n. in the remarkable orange colouration, as well as some morphological characters (except characters in diagnosis). These three species show allopatric distribution ranges, and the big mountain ranges known as the Nakhon Si Thammarat and Sunkala Khiri mountains possibly act as dispersal barriers.

########### Remarks.

The bright orange colouration is without doubt aposematic. There is some distinct variation within populations in the sternal lobe between male coxae 4, especially its shape: in most specimens the lobe is subrectangular, in others subtrapeziform, and its tip also varies – subtruncate/ subemarginate/ round. The shape of the sternal lobe of the new species is similar to that seen in *D. delfae* and *D. perakensis* sp. n., however, it looks thinner than those when seen in lateral view.

########### Coexisting species.

*Desmoxytes cervina* was found together with the new species in some localities.

########## Desmoxytes breviverpa

Taxon classificationAnimaliaPolydesmidaParadoxosomatidae

Srisonchai, Enghoff & Panha, 2016

Fig. 15

Desmoxytes breviverpa Srisonchai, Enghoff & Panha, 2016: 99.

########### Material examined. Holotype.

Male (CUMZ), THAILAND, Phrae Province, Long District, in front of Sareethai Cave, 18°16'43"N, 100°03'29"E, ca. 292 m a.s.l., 21 October 2014, leg. C. Sutcharit, W. Siriwut, K. Inkhavilay and R. Srisonchai.

########### Paratypes.

27 males, 7 females (CUMZ), 3 males, 2 females (ZMUC), same data as holotype. 5 males, 3 females (CUMZ), THAILAND, Phrae Province, Long District, in front of Sareethai Cave, 18°16'43"N, 100°03'29"E, ca. 292 m a.s.l., 21 July 2008, leg. N. Likhitrakarn. 1 male, 3 females (CUMZ), THAILAND, Lampang Province, Mae Tha District, Nakraua Subdistrict, Wat Tham Phra Sabai, 18°05'32"N, 99°32'03"E, ca. 328 m a.s.l., 21 July 2008, leg. S. Panha, P. Tongkerd and N. Likhitrakarn. 1 male, 5 females (CUMZ), THAILAND, Lampang Province, Mae Tha District, Tham Chakkrabhat Monastery (Wat Tham Chakkrabhat), 18°06'02"N, 99°56'48"E, ca. 210 m a.s.l., 8 October 2007, leg. U. Bantaowong, R. Chanabun, P. Pimvichai and T. Krutchuen.

########### Further specimens,

**all from THAILAND**: 2 males, 5 broken males, 1 male with rings 1–8, 6 broken females (CUMZ), Uttaradit Province, Thong Saen Khan District, Tham Chan (Chan Cave), 17°35'00"N, 100°25'21"E, ca. 164 m a.s.l., 22 July 2008, leg. S. Panha and ASRU members. 1 male, 1 female (CUMZ), Lampang Province, Mae Tha District, Nakraua Subdistrict, Wat Tham Phra Sabai, 18°05'32"N, 99°32'03"E, ca. 328 m a.s.l., 21 July 2008, leg. S. Panha and ASRU members.

########### Diagnosis.

Differs from all other *Desmoxytes* species by the combination of the following characters; body purple pink; collum with rows of 3+3 anterior, 1+1 intermediate and 2+2 posterior setiferous tubercles; sternal lobe between male coxae 4 subrectangular, quite long and thick when seen in lateral view; ventral lobe (vll) of lamina lateralis quite long, digitiform, directed ventrad; distal lobe (dlm) distally with two lamellae (mesal lamella smaller than lateral one; lateral lamella thin, tip directed almost in vertical plane); broad lobe (blm) thick, obviously demarcated from distal lobe by a deep and wide indentation; solenomere (sl) short.

########### Type locality.

THAILAND, Phrae Province, Long District, in front of Sareethai Cave.

########### Redescription

**(updated from Srisonchai et al. 2016).** SIZE: Length 28–33 mm (male), 33–38 mm (female); width of midbody metazona ca. 2.2 mm (male), 3.5 mm (female). Width of head < collum = body ring 2 = 3 ≤ 4 < 5 < 6–17, thereafter body gradually tapering toward telson.

COLOUR: In life with body shocking pink to purple (some female specimens brownish pink); paraterga vivid pink; metaterga and surface below paraterga brownish pink to brownish purple; head brown; antenna blackish brown (except distal part of antennomere 7 and antennomere 8 whitish); legs, sterna and epiproct pink; a few basal podomeres whitish pink. Colour in alcohol: after two years changed to pale brown.

ANTENNAE: Long and slender, reaching to body ring 6 (male), and 5 (female) when stretched dorsally.

COLLUM: With 3 transverse rows of setiferous tubercles, 3+3 anterior, 1+1 intermediate and 2+2 posterior tubercles (excluding small setiferous notches at base of paraterga), lateral tubercles of posterior row located at almost halfway to intermediate row; paraterga of collum low, elevated at ca. 30°, directed caudolaterad, with two setiferous notches on lateral margin (first inconspicuous notch located at the base of paratergum, second one conspicuous).

TEGUMENT: Moderately shining; collum, metaterga and surface below paraterga coarsely microgranulate; prozona finely shagreened; paraterga, sterna and epiproct smooth.

METATERGA: With 2 transverse rows of setiferous tubercles and rose thorn-like spines; metaterga 2–18 with 2+2 anterior and 2+2 posterior spines; metatergum 19 with 2+2 anterior and 2+2 posterior spines (tubercles in some specimens).

PARATERGA: Directed caudolaterad on body rings 2–17, elevated at ca. 45° (male) 40° (female); directed increasingly caudad on body rings 18 and 19; anterior margin with 2 distinct notches, on lateral margin of body rings 9, 10, 12, 13, 15–18 with tiny denticle near the tip.

TELSON: Epiproct: tip subtruncate; lateral setiferous tubercles inconspicuous; apical tubercles inconspicuous. Hypoproct subsemicircular (in some specimens subtrapeziform); caudal margin round, with inconspicuous setiferous tubercles.

STERNA: Cross-impressions shallow. Sternal lobe between male coxae 4 swollen, usually subrectangular (in some specimens subtrapeziform), quite long and slightly thick when seen in lateral view, tip usually emarginate (some specimens subtruncate).

LEGS: Very long and slender. Male femora 5 and 6 strongly humped ventrally in middle part.

**Figure 15.**
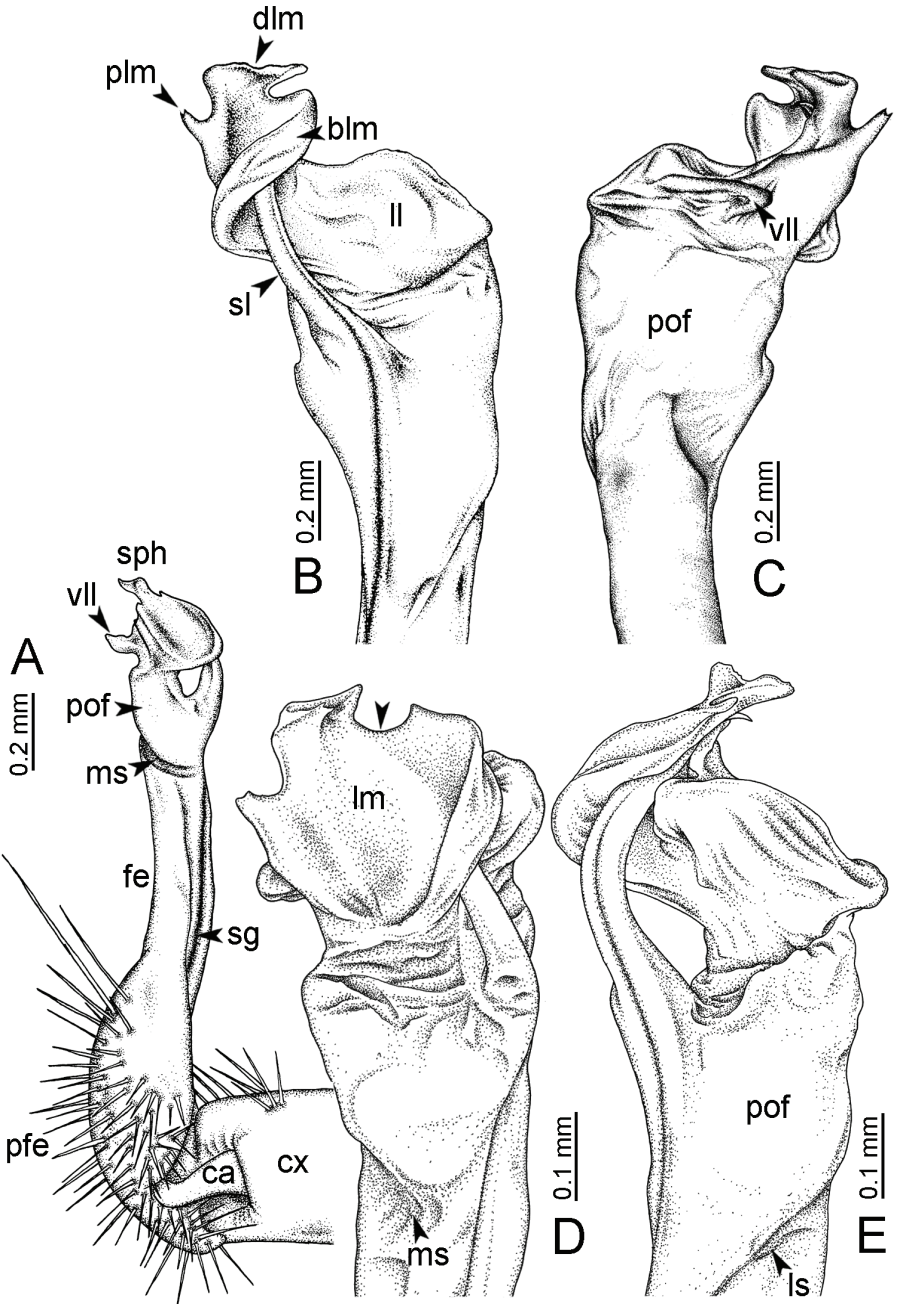
*Desmoxytes breviverpa* Srisonchai et al., 2016 (paratype) – right gonopod (modified from Srisonchai et al. 2016). **A** mesal view **B** dorsal view **C** ventral view **D** submesal view (arrow = indentation) **E** lateral view.

GONOPODS (Fig. 15): Coxa (cx) longer than prefemur. Cannula (ca) long and slender. Prefemur (pfe) ca. 2/3 as long as femur. Femur (fe) long and slender. Mesal sulcus (ms) and lateral sulcus (ls) very deep and wide. Postfemur (pof) conspicuous, ventrally very wide. Solenophore (sph) well-developed: lamina lateralis (ll) swollen, surface rough; ventral lobe (vll) quite long, digitiform, directed ventrad: lamina medialis (lm) well-developed; process (plm) long, spine-like, tip emarginate – not terminating in spines (in some specimens almost blunt), directed mesoanteriad; distal lobe (dlm) distally with two distinct lamellae (mesal lamella slightly smaller than lateral one; lateral lamella thin and broad, tip directed almost in vertical plane); broad lobe (blm) thick, obviously demarcated from distal lobe (dlm) by a deep and wide indentation. Solenomere (sl) quite short.

########### Distribution and habitat.

Known only from the type locality and nearby areas. *Desmoxytes breviverpa* was collected from limestone forest, crawling on logs and litter (Srisonchai et al. 2016). We believe it may be distributed though the central and southern parts of north Thailand. This species has been reported from Phrae, Lampang and Uttaradit Provinces. Therefore, *D. breviverpa* should be regarded as endemic for Thailand.

########### Remarks.

Specimens collected from Uttaradit Province south of the type locality showed the same morphological characters as the type specimens. No variation was found between populations although most limestone areas in north Thailand are geographically isolated by several big mountain ranges and quite far from each other.

Srisonchai et al. (2016) discussed variation of the tip of sternal lobe between male coxae 4; we found additional variation within populations as follows:

– Tip of sternal lobe in some specimens almost truncate (=subtruncate), albeit other specimens show an emarginate tip (slightly or deeply emarginate).

– Tip of hypoproct in some individuals subsemicircular, in others subtrapeziform.

– Tip of process (plm) of lamina medialis emarginate in most specimens, but almost blunt in some.

########### Coexisting species.

None known.

########### Corrections to Srisonchai et al. (2016)

Srisonchai et al. (2016, pp. 99–103) wrote in the description of this species that the paraterga (including paraterga of collum) are directed dorsolaterad. In fact they are directed caudolaterad as described above.

########## Desmoxytes cervina

Taxon classificationAnimaliaPolydesmidaParadoxosomatidae

(Pocock, 1895)

**Figure 16.**
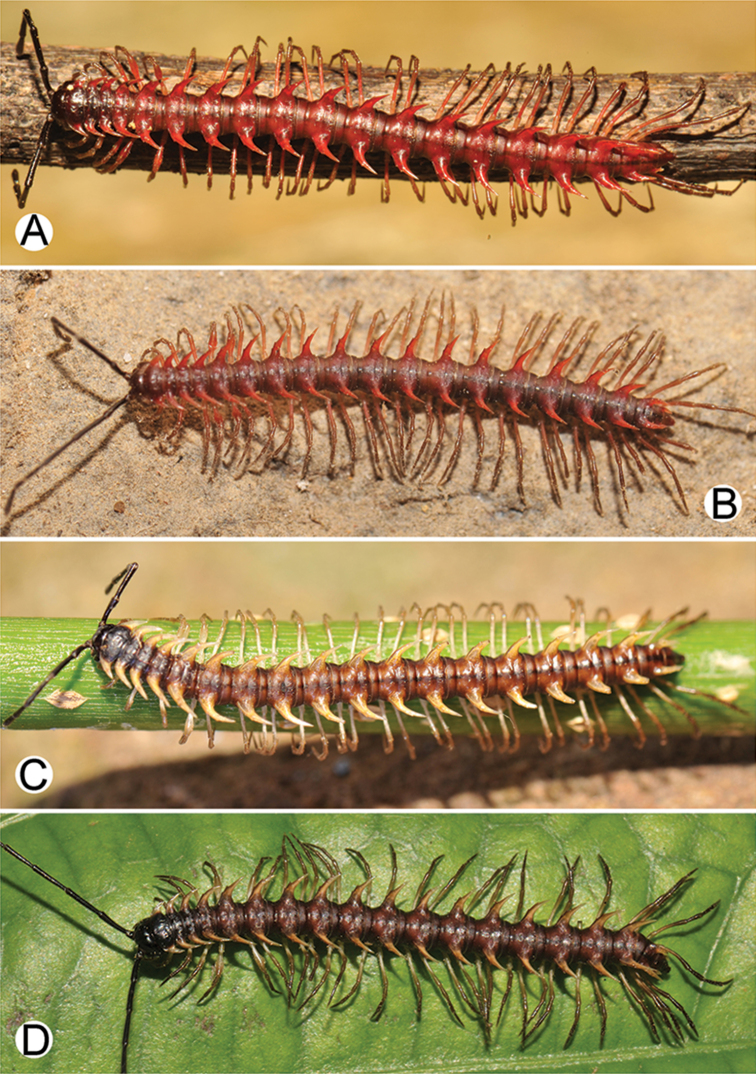
Photographs of live *Desmoxytes cervina* (Pocock, 1895) – males. **A** specimen from Wat Satit Khiri Rom **B** specimen from Phrayathtan Cave (Myanmar) **C** specimen from Ban Song Phi Nong **D** specimen from Wat Suwan Khuha.

**Figure 17.**
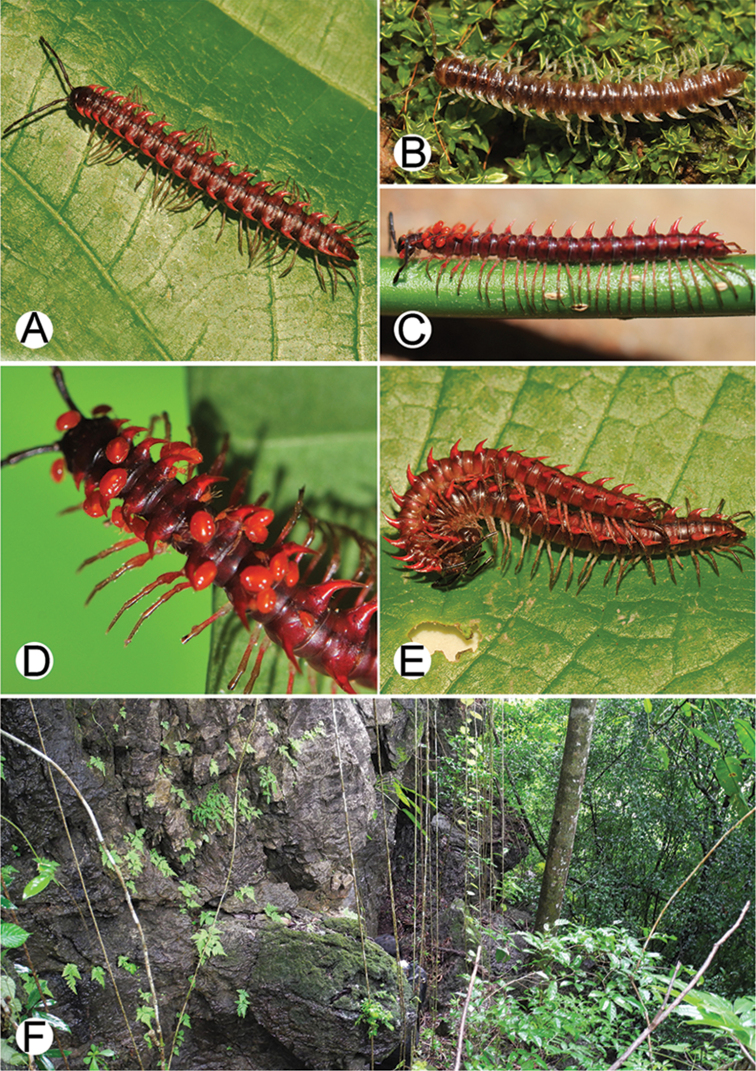
Photographs of live *Desmoxytes cervina* (Pocock, 1895) and habitat. **A–E** specimens from Ratchaprapa Dam **A** female **B** juvenile **C, D** male with parasitic mites **E** mating couple **F** habitat.

**Figure 18.**
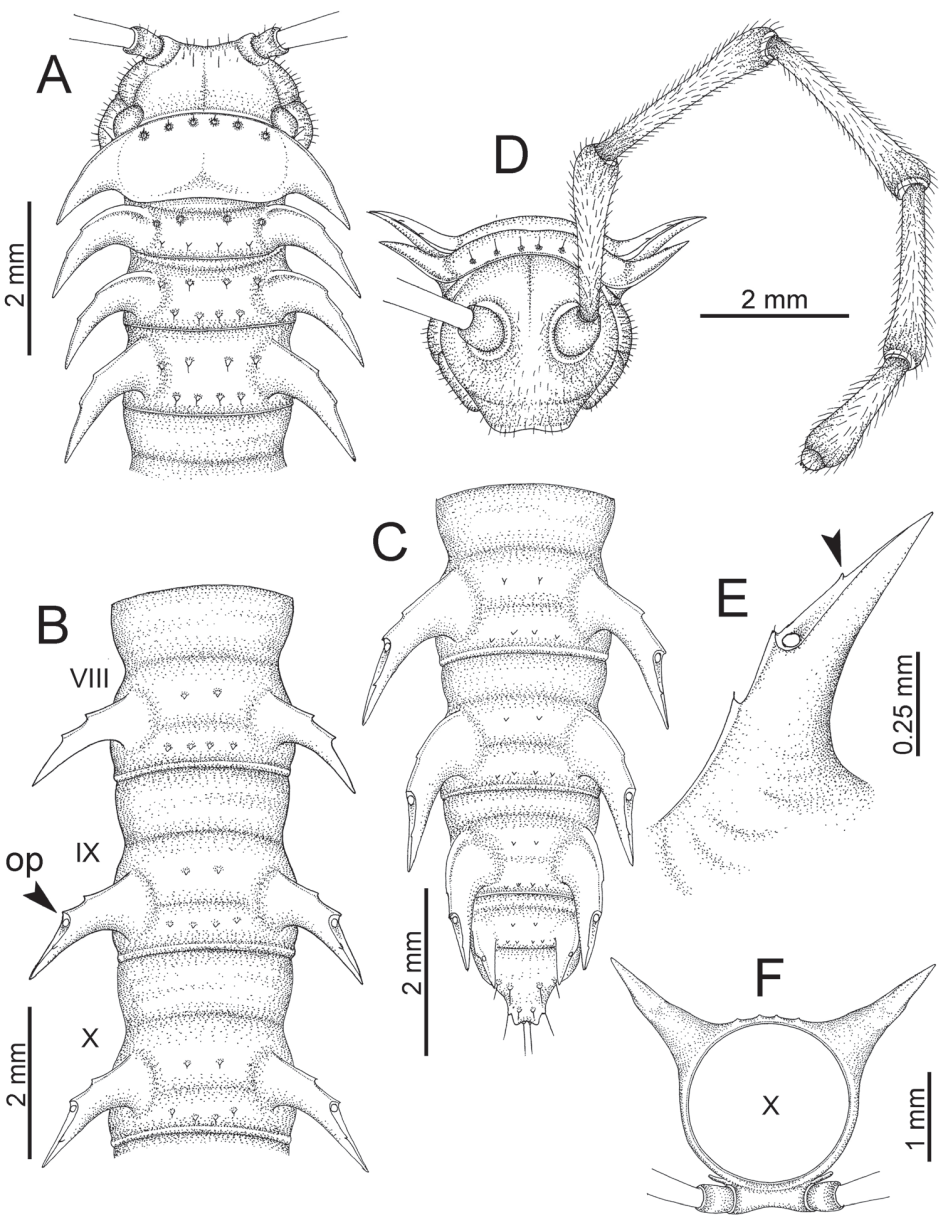
*Desmoxytes cervina* (Pocock, 1895) – specimen from Wat Satit Khirirom. **A** anterior body part **B** body rings 8–10 (op = ozopore) **C** posteriormost body rings and telson **D** head and antenna **E** paraterga of ring 10 (arrow = tiny denticle) **F** body ring 10.

**Figure 19.**
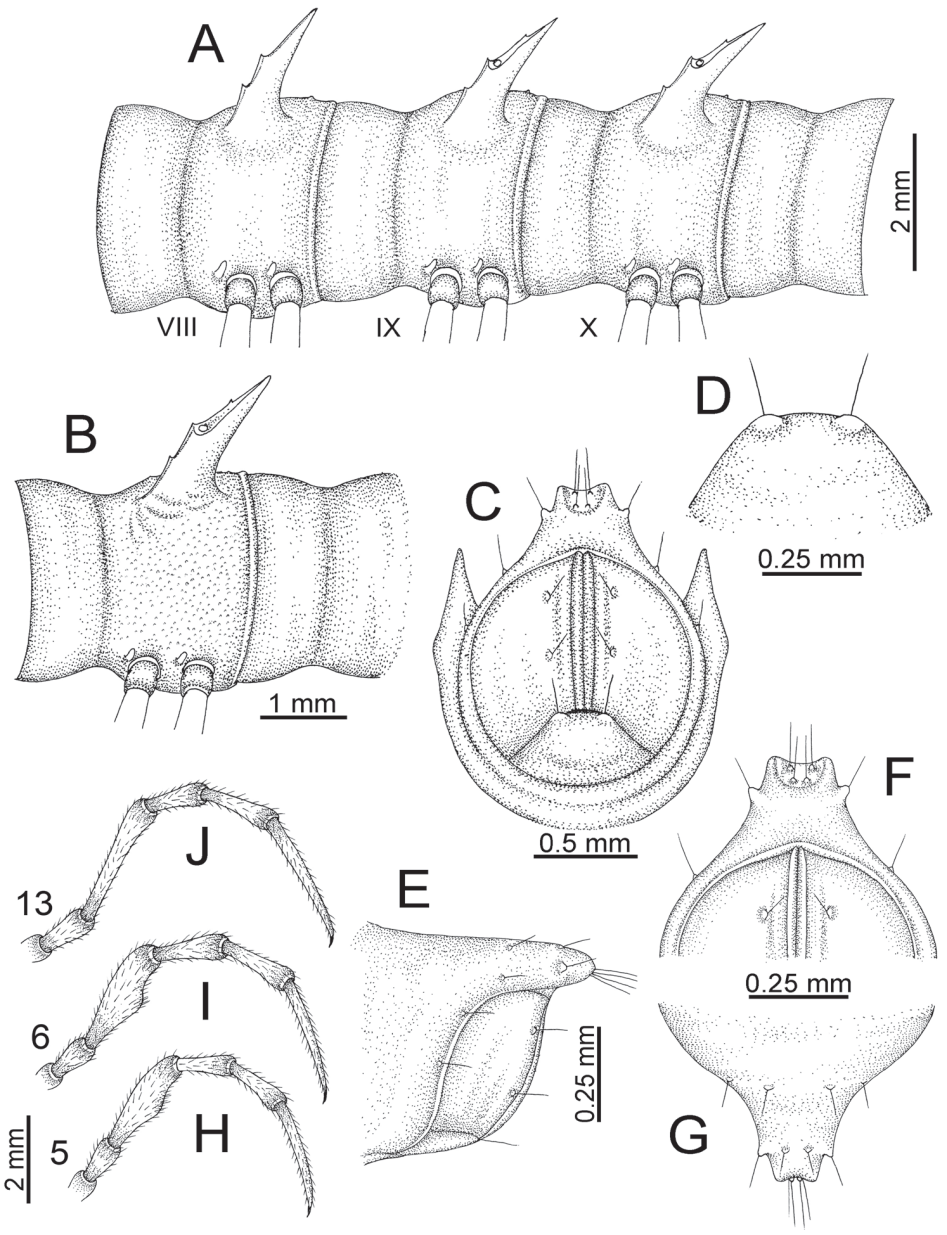
*Desmoxytes cervina* (Pocock, 1895) – specimen from Wat Satit Khirirom. **A** body rings 8–10 **B** sculpture of ring 10 **C, E** last ring and telson **D** hypoproct **F, G** epiproct **H** male leg 5 (right) **I** male leg 6 (right) **J** male leg 13 (right).

**Figure 20.**
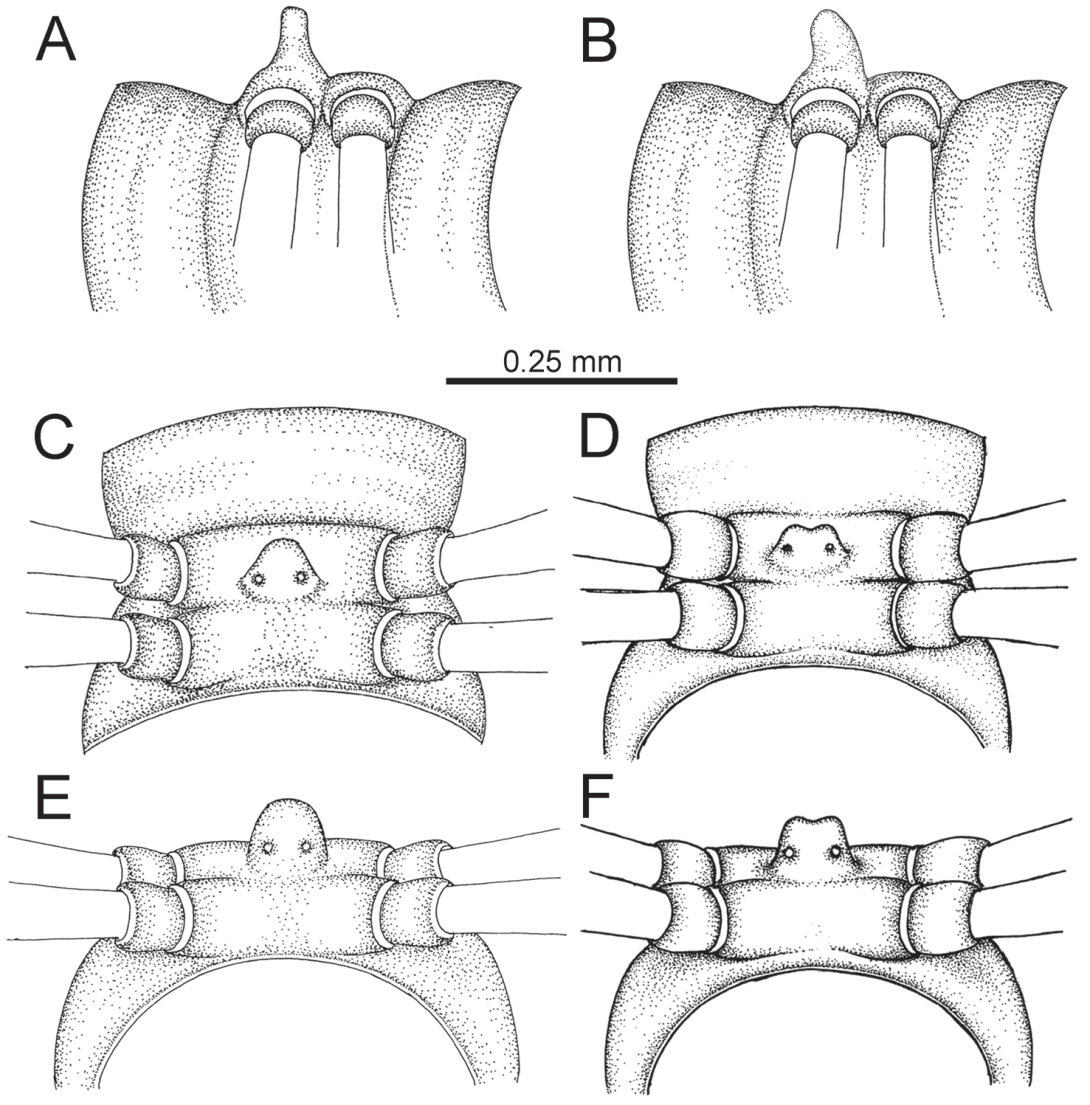
*Desmoxytes cervina* (Pocock, 1895), specimens from Wat Satit Khirirom – sternal lobe between male coxae 4. **A, B** lateral view **C, D** ventral view **E, F** caudal view.

**Figure 21.**
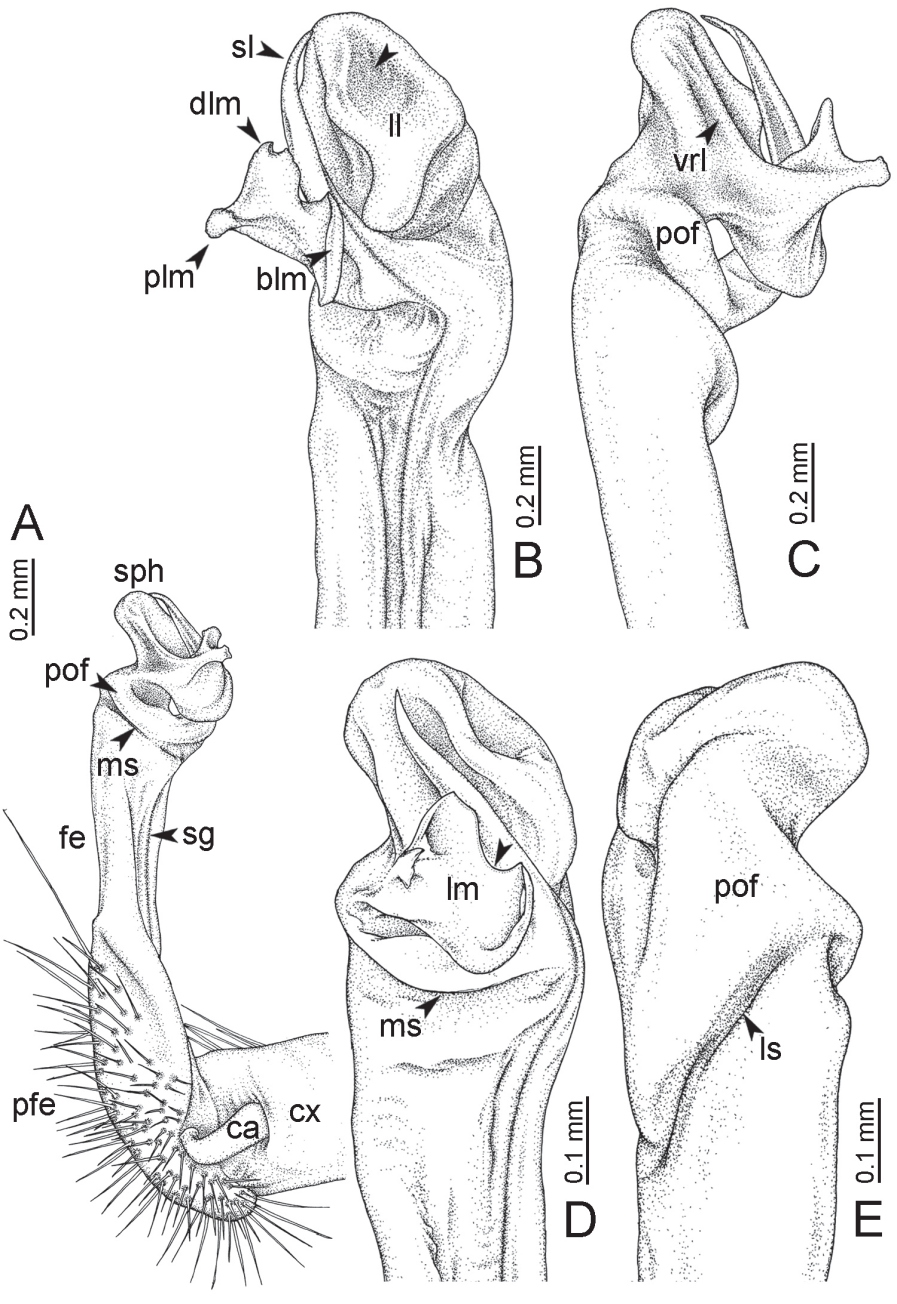
*Desmoxytes cervina* (Pocock, 1895), specimen from Wat Satit Khirirom – right gonopod. **A** mesal view **B** dorsal view (arrow = furrow) **C** ventral view **D** submesal view (arrow = indentation) **E** lateral view.

**Figure 22.**
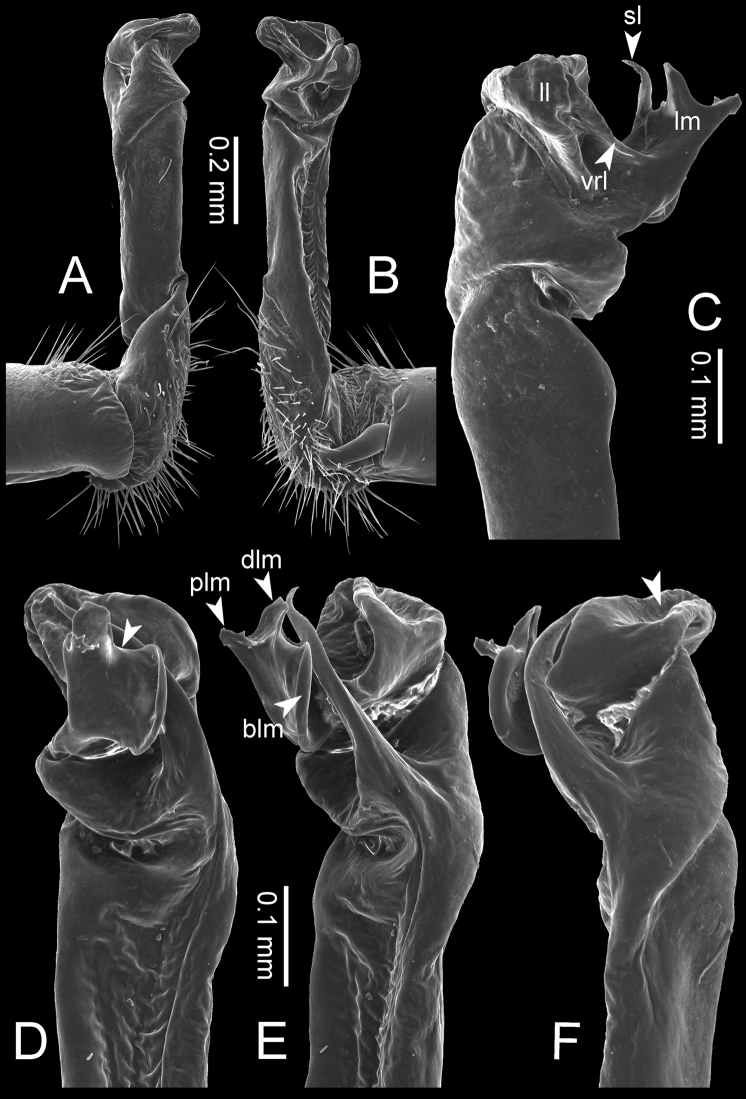
*Desmoxytes cervina* (Pocock, 1895), specimen from Wat Satit Khirirom – right gonopod. **A** lateral view **B** mesal view **C** ventral view **D** subdorsal view (arrow = indentation) **E** dorsal view **F** subdorsal view (arrow = furrow).

Figs 16 , 17 , 18 , 19 , 20 , 21 , 22

Prionopeltis cervinus Pocock, 1895: 831. Attems 1914: 203; 1936: 215.
Pratinus cervinus – Attems 1937: 120. Jeekel 1964: 63. Jeekel 1968: 61.
Desmoxytes cervina – Jeekel 1980a: 654. Golovatch and Enghoff 1994: 61. Nguyen and Sierwald 2013: 1241. Likhitrakarn et al. 2017: 19.
Desmoxytes pterygota Golovatch & Enghoff, 1994: 55, syn. n. Enghoff 2005: 96. Decker 2010: 30. Nguyen and Sierwald 2013: 1242.
Desmoxytes sp. – Golovatch and Enghoff 1994: 60.

########### Material examined.

**Lectotype.** Male (NHMUK, Bm 1892.5.4.76), MYANMAR, south Tenasserim, leg. E. W. Oates, most legs missing. Lectotype here designated.

Holotype (*D. pterygota*): Male (ZMUC), THAILAND, Ranong Province, Kapoe District, in forest at big waterfalls south of Kapoe (Khao Phra Narai Waterfall?), 15 November 1990, leg. M. Andersen and A. R. Rasmussen.

Paratypes (*D. pterygota*): 2 males (ZMUC), 1 male (ZMUM), THAILAND, Ranong Province, Kapoe District, in forest at big waterfalls south of Kapoe, 15 November 1990, leg. M. Andersen and A. R. Rasmussen.

########### Other material examined.

**MYANMAR**: 10 males, 7 females (CUMZ), Tanintharyi Region, Lenya National Park, Phayarhtan Cave (Buddha Cave), approximately 10 km from Ban Nam Yen Village, inside the deep rainforest near limestone mountain, on decaying wood and under bark, 11°13'50"N, 99°10'35"E, ca. 85 m a.s.l., 6 June 2015, leg. C. Sutcharit, R. Chanabun and R. Srisonchai.

**THAILAND: Chumphon Province**: 1 male, 2 females (CUMZ), Mueang Chumphon District, Tham Chang Phuek Bureau of Monks, 10°26'47"N, 99°02'06"E, ca. 93 m a.s.l., 13 March 2017, leg. C. Sutcharit, R. Srisonchai and ASRU members. 1 male (CUMZ), Sawi District, Wat Nam Cha, 10°17'54"N, 99°01'57"E, ca. 105 m a.s.l., 3 July 2017, leg. C. Sutcharit, R. Srisonchai and ASRU members.

**Krabi Province**: 1 male (CUMZ), Ao-Luek District, P.N. Moutain Resort, 8°24'09"N, 98°44'18"E, ca. 60 m a.s.l., 30 August 2015, leg. C. Sutcharit and ASRU members. 1 male remaining rings 7–20 (CUMZ), Khlong Thom District, Emerald Blue Pool, 7°55'30"N, 99°16'05"E, ca. 67 m a.s.l., 15 January 2009, leg. S. Panha and ASRU members. 1 female (CUMZ), Muang Krabi District, Wat Tham Sue (Tiger Cave), valley behind Tiger Cave, 8°07'38"N, 98°55'27"E, ca. 86 m a.s.l., 18 May 2010, leg. S. Panha and ASRU members. 2 males, 3 females, 1 broken male and missing gonopods (CUMZ), Muang Krabi District, Wat Tham Sue (Tiger Cave), valley behind Tiger Cave, 8°07'38"N, 98°55'27"E, ca. 86 m a.s.l., 7 October 2006, leg. S. Panha and ASRU members. 1 male, 4 females (CUMZ), Muang Krabi District, Wat Tham Sue (Tiger Cave), valley behind Tiger Cave, 8°07'38"N, 98°55'27"E, ca. 86 m a.s.l., 24 August 2014, leg. S. Panha and ASRU members. 2 males, 1 female, 1 juvenile (CUMZ), Muang Krabi District, Wat Tham Sue (Tiger Cave), valley behind Tiger Cave, 8°07'38"N, 98°55'27"E, ca. 86 m a.s.l., 30 August 2015, leg. C. Sutcharit, R. Srisonchai and ASRU members. 15 males, 3 females, 1 juvenile (CUMZ), Muang Krabi District, Wat Tham Sue (Tiger Cave), valley behind Tiger Cave, 8°07'38"N, 98°55'27"E, ca. 86 m a.s.l., 9 July 2017, leg. C. Sutcharit, R. Srisonchai and ASRU members. 11 males, 6 females, 1 juvenile (CUMZ), Muang Krabi District, Wat Tham Sue (Tiger Cave), valley behind Tiger Cave, 8°07'38"N, 98°55'27"E, ca. 86 m a.s.l., 25 July 2017, leg. C. Sutcharit, R. Srisonchai and ASRU members.

**Nakhon Si Thammarat Province**: 2 males, 2 females, 4 juveniles (ZMUC), Sichon District, Khao Lark Waterfall, 25 August 2007, leg. ASRU members. 1 female (CUMZ), Thung Song District, Yong Waterfall, 8°10'21"N, 99°44'34"E, ca. 138 m a.s.l., 20 July 2008, leg. S. Panha and ASRU members. 1 male missing gonopods (CUMZ), Nopphitam District, Krung Ching Waterfall, 8°43'27"N, 99°40'04"E, ca. 173 m a.s.l., 17 January 2013, leg. C. Sutcharit, R. Srisonchai and ASRU members. 1 female (CUMZ), Khanom District, Nai Plao Beach, 9°07'26"N, 99°52'60"E, ca. 20 m a.s.l., 4 December 2015, leg. S. Panha and ASRU members. Many specimens (CUMZ), Tham Phannara District, Wat Tham Kanlaya Namit, 8°30'48"N, 99°22'52"E, ca. 51 m a.s.l., 4 July 2017, leg. C. Sutcharit, R. Srisonchai and ASRU members. Many specimens (CUMZ), Tham Phannara District, Wat Tham Thong Phannara, 8°25'21"N, 99°22'47"E, ca. 32 m a.s.l., 4 July 2017, leg. C. Sutcharit, R. Srisonchai and ASRU members.

**Phang Nga Province**: 1 male missing gonopods, 3 males, 1 female (CUMZ), Thai Mueang District, Khaolak-Lumru National Park, 8°37'35"N, 98°14'25"E, ca. 72 m a.s.l., 7 October 2006, leg. S. Panha and ASRU members. 1 male, 1 female (ZMUC), Thap Put District, Highway No. 4 Phet Kasem Road ca. 0.5 km north of the Headquarters of the Khao Lak–Lamru National Park, on the street next to secondary rainforest. 8°37'N, 98°14'E, ca. 30–40 m a.s.l., 29 August–12 September 2008, leg. N. Laufer. 1 male missing gonopods, 1 male (CUMZ), Khura Buri District, Mu Koh Surin National Park, Koh Surin Nuea, 9°26'27"N, 97°52'11"E, ca. 39 m a.s.l., 8 April 2012, leg. S. Panha and ASRU members. 1 broken male and missing gonopods (CUMZ), Mueang Phang Nga District, Tham Nam Pud, 8°27'50"N, 98°32'36"E, ca. 58 m a.s.l., 7 October 2006, leg. S. Panha and ASRU members. Many specimens (CUMZ), Mueang Phang Nga District, Tham Nam Pud, 8°27'50"N, 98°32'36"E, ca. 58 m a.s.l., 5 August 2015, leg. C. Sutcharit, R. Srisonchai and ASRU members. 1 male (CUMZ), Mueang Phang Nga District, Tham Pha Sue Bureau of Monks, 8°28'24"N, 98°32'15"E, ca. 78 m a.s.l., 10 July 2017, leg. C. Sutcharit, R. Srisonchai and ASRU members. 2 females (CUMZ), Mueang Phang Nga District, Tham Nam Pud Bureau of Monks, 8 October 2006, leg. S. Panha and ASRU members. 1 male, 1 female (CUMZ), Mueang Phang Nga District, Tao Thong Waterfall, 8°29'08"N, 98°35'09"E, ca. 25 m a.s.l., 7 October 2006, leg. S. Panha and ASRU members. 1 female (CUMZ), Mueang Phang Nga District, Phung Chang Cave, 8°26'34"N, 98°30'59"E, ca. 24 m a.s.l., 26 September 2009, leg. S. Panha and ASRU members. 3 males, 2 females, 14 juveniles (CUMZ), Mueang Phang Nga District, Phung Chang Cave, 8°26'34"N, 98°30'59"E, ca. 24 m a.s.l., 6 August 2014, leg. C. Sutcharit, R. Srisonchai and ASRU members. Many specimens (CUMZ), Mueang Phang Nga District, Phung Chang Cave, 8°26'34"N, 98°30'59"E, ca. 24 m a.s.l., 5 August 2015, leg. C. Sutcharit, R. Srisonchai and ASRU members. 5 males, 4 females (CUMZ), Mueang Phang Nga District, Phung Chang Cave, 8°26'34"N, 98°30'59"E, ca. 24 m a.s.l., 8 August 2016, leg. C. Sutcharit, R. Srisonchai, and ASRU members. 3 males, 2 juveniles (CUMZ), Takua Thung District, Wat Suwan Khuha (Monkey Cave), 8°25'42"N, 98°28'22"E, ca. 25 m a.s.l., 8 August 2016, leg. C. Sutcharit, R. Srisonchai, and ASRU members. Many specimens (CUMZ), Thap Put District, Wat Khiri Wong (Tham Kob), 8°31'57"N, 98°34'40"E, ca. 97 m a.s.l., 9 July 2017, leg. C. Sutcharit, R. Srisonchai, and ASRU members.

**Phuket Province**: 1 male of *Desmoxytes* sp. (ZMUC), Thalang District, Thepkrasattree Subdistrict, Tonsai Waterfall, 8°01'44"N, 98°21'45"E, ca. 67 m a.s.l., 12 October 1991, leg. M. Anderson, O. Martin, and N. Scharff. 1 female (CUMZ), Mueang Phuket District, Panwa Cave, 7°48'04"N, 98°24'34"E, ca. 25 m a.s.l., June 2007, leg. S. Panha and ASRU members. 1 female (CUMZ), Mueang Phuket District, Panwa Cave, 7°48'04"N, 98°24'34"E, ca. 25 m a.s.l., 5 November 2007, leg. S. Panha and ASRU members.

**Ranong Province**: 1 male (CUMZ), Kra Buri District, Tham Phra Khayang, 10°19'35"N, 98°45'54"E, ca. 51 m a.s.l., 21 November 2015, leg. S. Panha, P. Tongkerd, and A. Pholyotha. 1 female (CUMZ), Kra Buri District, Bok Krai Waterfall, 10°22'35"N, 98°51'22"E, ca. 106 m a.s.l., 3 January 2013, leg. S. Panha, P. Tongkerd, and A. Pholyotha.

**Surat Thani Province**: 2 broken and mixed females, 2 females remaining rings 13–20, 1 broken female (CUMZ), Phanom District, Khlong Phanom National Park, 8°52'44"N, 98°40'26"E, ca. 68 m a.s.l., 28 August 2007, leg. S. Panha and ASRU members. 1 male, 6 juveniles (CUMZ), Phanom District, Khlong Phanom National Park, Pha Daeng, 8°53'41"N, 98°33'12"E, ca. 67 m a.s.l., 7 August 2016, leg. C. Sutcharit, R. Srisonchai, and ASRU members. 8 males, 3 females (CUMZ), Phanom District, Khlong Phanom National Park, Pha Daeng, 8°53'41"N, 98°33'12"E, ca. 67 m a.s.l., 1 August 2017, leg. C. Sutcharit, R. Srisonchai, and ASRU members. 4 males (CUMZ), Phanom District, Ban Song Phi Nong, 8°50'51"N, 98°44'16"E, ca. 74 m a.s.l., 7 August 2016, leg. C. Sutcharit, R. Srisonchai, and ASRU members. 10 males, 3 females, 1 broken male missing gonopods (CUMZ), Ban Ta Khun District, Ratchaprapa Dam, 8°57'22"N, 98°48'22"E, ca. 53 m a.s.l., 8 October 2008, leg. S. Panha and ASRU members. Many specimens (CUMZ), Ban Ta Khun District, Ratchaprapa Dam, 8°57'22"N, 98°48'22"E, ca. 53 m a.s.l., 4 August 2014, leg. C. Sutcharit, R. Srisonchai, and ASRU members. Many specimens (CUMZ), Ban Ta Khun District, Ratchaprapa Dam, 8°57'22"N, 98°48'22"E, ca. 53 m a.s.l., 3 August 2015, leg. C. Sutcharit, R. Srisonchai, and ASRU members. 1 male (CUMZ), Ban Ta Khun District, Ratchaprapa Dam, 8°57'22"N, 98°48'22"E, ca. 53 m a.s.l., 5 August 2016, leg. C. Sutcharit, R. Srisonchai, and ASRU members. 1 male (CUMZ), Ban Ta Khun District, Khlong Hoi, 8 October 2008, leg. S. Panha and ASRU members. 2 males, 1 male missing gonopods, 1 juvenile, 1 broken juvenile (CUMZ), Ban Ta Khun District, Khao Wong Water Supply Station, 8°55'47"N, 98°56'25"E, ca. 97 m a.s.l., 9 October 2008, leg. S. Panha and ASRU members. 2 males (CUMZ), Ban Ta Khun District, Wat Khao Pang (Suspension Bridge), 8°56'54"N, 98°49'21"E, ca. 24 m a.s.l., 5 May 2017, leg. S. Panha and ASRU members. 1 male, 1 male missing gonopods, 2 females, 2 broken females, 1 juvenile (CUMZ), Khirirat Nikhom District, Wat Satit Khirirom, 9°01'48"N, 98°59'12"E, ca. 47 m a.s.l., 8 October 2008, leg. S. Panha and ASRU members. 1 male (CUMZ), Khirirat Nikhom District, Wat Satit Khirirom, 9°01'48"N, 98°59'12"E, ca. 47 m a.s.l., 5 September 2009, leg. S. Panha and ASRU members. 27 males, 10 females (CUMZ), 2 males, 1 female (ZMUC), 1 male, 1 female (ZMUM), 1 male, 1 female (NHMW), 1 male, 1 female (NHMUK), Khirirat Nikhom District, Wat Satit Khirirom, 9°01'48"N, 98°59'12"E, ca. 47 m a.s.l., 10 July 2017, leg. C. Sutcharit, R. Srisonchai, and ASRU members. 1 male missing right gonopod (CUMZ), Khirirat Nikhom District, km3 near Khirirat Nikhom City, 9 October 2008, leg. S. Panha and ASRU members. 1 male, 1 female (CUMZ), Khirirat Nikhom District, Tham Wang Badan Bureau of Monks, 8°56'09"N, 98°57'28"E, ca. 69 m a.s.l., 3 August 2017, leg. C. Sutcharit, R. Srisonchai, and ASRU members. 3 broken males (CUMZ), Ko Samui District, Na Muang Waterfall, 9°27'58"N, 99°59'02"E, ca. 53 m a.s.l., 27 January 2006, leg. S. Panha and ASRU members. 1 female (CUMZ), Ko Samui District, Na Muang Waterfall, 9°27'58"N, 99°59'02"E, ca. 53 m a.s.l., 4 December 2015, leg. S. Panha, P. Tongkerd and A. Pholyotha. 1 male remaining rings 1–11, 1 female (ZMUM), Ko Samui, Thailand, June 2013, leg. Korabushkin Daniil. 10 males, 5 females, 4 juveniles (CUMZ), Wiang Sa District, Khiri Rat Pattana Bureau of Monks (Wat Khao Poon), 8°31'37"N, 99°22'59"E, ca. 68 m a.s.l., 4 July 2017, leg. C. Sutcharit, R. Srisonchai, and ASRU members. 1 male, 1 female (CUMZ), Ban Na San District, Khao Kok Maharat Bureau of Monks, 8°41'33"N, 99°22'45"E, ca. 71 m a.s.l., 4 July 2017, leg. C. Sutcharit, R. Srisonchai, and ASRU members.

########### Diagnosis.

Differs from other *Desmoxytes* species by the combination of the following characters: body colour brownish red/brown/pale brown; paraterga brownish red/red/yellowish brown; metaterga 2–4 with 2+2 (anterior row) and 2+2 (posterior row) tubercles, metaterga 5–19 with 2(1)+2(1) (anterior row) and 2+2 (posterior row) tubercles; lamina lateralis (ll) stout; anterolaterally with a long, distinct, deep and wide furrow; ventral ridge (vrl) of lamina lateralis very long, wide, conspicuous; process (plm) of lamina medialis long, distinctly demarcated from distal lobe, irregularly shaped, directed mesodorsad; distal lobe (dlm) of lamina medialis distally with one distinct lamella; broad lobe (blm) slightly thick at the edge, distinctly demarcated from distal lobe (dlm) by a wide and shallow indentation.

########### Type locality.

MYANMAR, southern Myanmar, Tenasserim [Tanintharyi Region].

########### Redescription.

SIZE: Length 25–31 mm (male), 33–39 mm (female); width of midbody metazona ca. 2.1 mm (male), 3.6 mm (female). Width of head < collum < body ring 2 ≥ 3 = 4 < 5–16, thereafter body gradually tapering towards telson.

COLOUR (Figs 16, 17A–E): In life with two colour morphs. Brownish red morph – body brownish red (testaceous); head and antenna brownish black (except distal part of antennomere 7 and antennomere 8 whitish); collum, epiproct and legs brown; rings 2–3 brownish red or brown; metaterga, surface below paraterga and sterna brownish red; paraterga brownish red or red; a few basal podomeres reddish brown. Brown morph – body brown (female pale brown); head and antenna (except distal part of antennomere 7 and antennomere 8 whitish) brownish black or black; collum and rings 2–3 brownish black; metaterga, surface below paraterga, epiproct and legs brown; paraterga yellowish brown; sterna pale brown; a few basal podomeres brownish white. Colour in alcohol: after 100 years changed to greenish dark or greenish brown, after 5–10 years changed to pale brown.

ANTENNAE (Fig. 18D): Very long and slender, reaching to body ring 6 or 7 (male) and 5 or 6 (female) when stretched dorsally.

COLLUM (Fig. 18A): With 1 transverse row of setae, 3+3 anterior setae; paraterga of collum low, elevated at ca. 15°–20°, directed caudolaterad, with two inconspicuous notches on lateral margin.

TEGUMENT: Moderately shining; collum coarsely microgranulate; prozona finely shagreened; metaterga and surface below paraterga finely microgranulate; paraterga, sterna and epiproct smooth.

METATERGA (Fig. 18A–C): With 2 transverse rows of tubercles; metaterga 2–4 with 2+2 anterior tubercles and 2+2 posterior tubercles; metaterga 5–19 with 2(1)+2(1) anterior tubercles and 2+2 posterior tubercles (lateral setae of anterior row in some specimens very distinct, in some specimens poorly developed).

PARATERGA (Fig. 18E, F): Long, strongly developed; directed caudolaterad on body rings 2–17, elevated at ca. 45° (male) 40° (female); directed increasingly caudad on body rings 18 and 19; anterior margin with 2 distinct notches, on lateral margin of body rings 9, 10, 12, 13, 15–18 with tiny denticle near the tip.

TELSON (Fig. 19C–G): Epiproct: tip usually truncate (in some specimens slightly emarginate); lateral setiferous tubercles and apical setiferous tubercles conspicuous, coniform. Hypoproct subtrapeziform; caudal margin slightly round (in some specimens subtruncate), with inconspicuous setiferous tubercles.

STERNA (Fig. 20): Cross-impressions shallow (in some specimens slightly deep). Sternal lobe between male coxae 4 swollen, usually subtrapeziform (in some specimens subsemicircular), usually thick when seen in lateral view (in some specimens thin), slightly attenuated near tip, tip round/subtruncate/slightly emarginate.

LEGS (Fig. 19H–J): Very long and slender. Male femora 5 and 6 strongly humped ventrally in middle portion.

GONOPODS (Figs 21, 22): Coxa (cx) longer than prefemur. Cannula (ca) long and slender. Prefemur (pfe) ca. 2/3 as long as femur. Femur (fe) long and slender. Mesal sulcus (ms) and lateral sulcus (ls) conspicuous, very deep and narrow. Postfemur (pof) conspicuous, ventrally narrow. Solenophore (sph) well-developed: lamina lateralis (ll) stout; anterolaterally with a long, distinct, deep and wide furrow; with a long, wide and conspicuous ventral ridge (vrl): lamina medialis (lm) well-developed; process (plm) long, of irregular shape (varies within population), distinctly demarcated from distal lobe, tip blunt (in some specimens terminating in spines), directed mesodorsad; distal lobe (dlm) distally with one distinct lamella; broad lobe (blm) slightly thick at the edge, distinctly demarcated from distal lobe (dlm) by a wide and shallow indentation. Solenomere (sl) long.

########### Distribution and habitat.

*Desmoxytes cervina* is known from Myanmar (Lenya National Park) and Thailand (Chumphon, Krabi, Nakhon Si Thammarat, Phang Nga, Phuket, Ranong, and Surat Thani Provinces). The type locality is in south of Tenasserim, but we do not know the exact location, probably somewhere near Taninthayi township. The locality of the paralectotype (see below) “Malewoon, Tenasserim”, is currently known as Maliwan Village.

Almost all specimens were collected from limestone habitats, a few specimens were collected from granitic areas. Interestingly, *D. cervina* was also found in some islands in both the Andaman Sea and the Gulf of Thailand (Mu Koh Surin National Park, Phuket, and Ko Samui).

Decker (2010) classified “*D. pterygota*” as endemic for Thailand due to its narrow range near the type locality. After intensive collecting and synonymising of *D. cervina* and “*D. pterygota*”, the known distribution range has expanded to southern Myanmar and southern Thailand.

This species was found living together with *D. delfae* and *D. corythosaurus* sp. n. According to our observations, it is probable that they may even share microhabitat: humid rocks, branches of trees and rock walls.

########### Note on material.

This species was described based on two males, one of which was collected by L. Fea (Malewoon, Tenasserim – MSNG) and the other collected by E. W. Oates (south Tenasserim – NHMUK).

Jeekel (1964) revised this species by examining the male in MSNG; he stated that “in anticipation of the designation as holotype of the specimen collected by Oates in the British Museum, I have labelled this specimen as paratype”. However, the holotype has never been designated in the original description. R.L. Hoffman visited NHMUK and labelled the specimen collected by Oates as paratype, but he did not publish this designation, which is in conflict with Jeekel’s (1964) paratype designation. Therefore, we here designate the male collected by Oates and belonging to NHMUK as lectotype to stabilise the name. The male in MSNG (which we have not examined) is considered to be a paralectotype.

Some specimens kept in MHNG and identified as “*D. pterygota*” by Decker (2010), are probably *D. cervina* because the localities fall within the distribution range of this species.

########### Remarks.

This is the first report of the colour of living specimens for this species; the brownish red colour is apparently aposematic. Pocock (1895) and Golovatch and Enghoff (1994) did not mention the colour of living specimens. We found two colour morphs of *D. cervina*: brownish red and brown. The majority of specimens are brownish red, and the minority are brown; the latter colour can be found in a few specimens within a population.

Interestingly, brownish red and brown morphs occur in the same habitat in the valley behind Tiger Cave and Ban Song Phi Nong. The brown morph was found at Wat Tham Kanlaya Namit, Wat Tham Thong Phannara, Tham Nam Pud, Phung Chang Cave and Wat Suwan Khuha (Monkey Cave). Specimens from the remaining localities are of the brownish red morph. We examined the morphological characters of all specimens of both colour morphs; all specimens exhibit the same morphology, especially in the gonopods which are identical. Perhaps the difference in colour is caused by environmental factors and/or genetic variation.

We assume that the type material of both *D. cervina*, collected by Oates and Fea a hundred years ago, and “*D. pterygota*” collected by M. Andersen and A.R. Rasmussen 17 years ago, did probably exhibit brownish red colour because all specimens near the type localities are brownish red. We found additionally that the colour of some females is pale brownish red or pale brown, and the colour of juveniles is pale brown.

Jeekel (1964) wrote in his redescription that *D. cervina* shows collum without setae, metaterga 2–19 with 1+1 anterior tubercles and 2+2 posterior tubercles. Golovatch and Enghoff (1994) distinguished “*D. pterygota*” from *D. cervina* by having smooth metaterga. They also described “*D. pterygota*” as having no pleurosternal carinae, antenna reaching to ring 4 in male, collum with 2 rows of 3+3 anterior setae and 1+1 intermediate setae, metaterga 2–19 with 2+2 anterior cones and 2+2 posterior cones. After examination of all type material and newly collected specimens of *D. cervina* and “*D. pterygota*”, we found that:

– all specimens of *D. cervina* and “*D. pterygota*” display fine microgranulation on the metaterga.

– all specimens have pleurosternal carinae, in body ring 2 very distinct and crest-like, in ring 3 very small, thereafter absent.

– antenna reaches to ring 6–7 in male and to ring 5–6 in female of both *D. cervina* and “*D. pterygota*”.

– collum has 3+3 anterior setae in all specimens – because Jeekel studied old preserved specimens, the setae may have been lost over time.

– metaterga 5–19 varies within populations; metaterga 2–4 with 2+2 anterior tubercles and 2+2 posterior tubercles, metaterga 5–19 with 2+2/1+1 anterior and 2+2 posterior tubercles.

Several other characters show variability, as follows:

**I. variation within populations**

– size of tubercles on metaterga: tubercles conspicuous in some specimens, inconspicuous in the others (bigger and more obvious in the holotype of “*D. pterygota*” than in the lectotype of *D. cervina*).

– tip of process (plm) of lamina medialis: in some specimens terminating in one blunt process, in others terminating in a sharp spine.

– shape of sternal lobe between male coxae 4: in some individuals subtrapeziform, in others subsemicircular.

– tip of sternal lobe between male coxae 4: in some specimens round, in some subtruncate, in others emarginate.

– cross-impressions on sternum: in some individuals shallow and faint, in others slightly deep.

– tip of epiproct: in some individuals truncate, in others slightly emarginate.

– caudal margin of hypoproct: in some specimens slightly round, in others subtruncate.

– size of sternal lobe between male coxae 4 when seen in lateral view in specimens from Wat Satit Khirirom: in some specimens thick, in others thin.

**II. variation between populations**

– colour: all individuals in the same population usually have the same colour: brown or brownish red. However, in some populations (valley behind Tiger Cave and Ban Song Phi Nong) brownish red and brown individuals coexist.

Although the male paralectotype of *D. cervina* (in MSNG) has not been examined by us, the morphological characters for this specimen as redescribed by Jeekel (1964) perfectly match the morphology of the numerous other specimens we have seen. Based on our analysis of morphology and variation of these specimens we have synonymised “*D. pterygota*” under *D. cervina*.

Distribution data support the synonymisation: the type localities of “*D. pterygota*” (Ranong Province) and *D. cervina* (Tenasserim = Taninthayi) are very close to each other. Our intensive surveys prove that this species is distributed quite widely, but nevertheless is found in south Myanmar and south Thailand only.

During the field survey, we noticed several adult males of *D. cervina* which were infested with red mites. The mites are probably larvae of the genus *Leptus* Latreille, 1796 (Prostigmata, family Erythraeidae). Associations between mites and millipedes may be of a phoretic or a parasitic nature (Gerdeman et al. 2000, Swafford and Bond 2010, Farfan and Klompen 2012, Mwabvu 2014). In Fig. 17C and D, several engorged mites are seen along with a few small, non-engorged ones, and we therefore assume that the mite species found in *D. cervina* is parasitic, like other *Leptus* larvae. Southcott (1992) described *Leptus millipedius* from julid millipedes, but this is the first record of a parasitic prostigmatan mite from a paradoxosomatid millipede. The only record of a mite from Paradoxosomatidae concerns the widespread *Oxidus gracilis* (C. L. Koch, 1847) which was reported as associated with *Cosmolaelaps hortensis* Ishikawa, 1986 (Mesostigmata, family Laelapidae) (Ishikawa 1986).

Golovatch and Enghoff (1994) reported one broken male of “*Desmoxytes* sp.” from Phuket Province, Thailand. According to the remarks of Golovatch and Enghoff (1994), the gonopod characters of this specimen were similar to *D. delfae*, but the paraterga showed a higher degree of elevation. We examined this specimen again and found it to share gonopod and other characters with *D. cervina*. We therefore treat “*Desmoxytes* sp.” as *D. cervina*.

########### Coexisting species.

*D. delfae* at Wat Tham Sue (Tiger Cave), Krung Ching Waterfall, Khiri Rat Pattana Bureau of Monks (Wat Khao Poon); *D. corythosaurus* sp. n. at Ban Song Phi Nong.

########## Desmoxytes corythosaurus

Taxon classificationAnimaliaPolydesmidaParadoxosomatidae

Srisonchai, Enghoff & Panha sp. n.

http://zoobank.org/DA7B808F-B331-4568-8F4B-0356D78AD5E4

**Figure 23.**
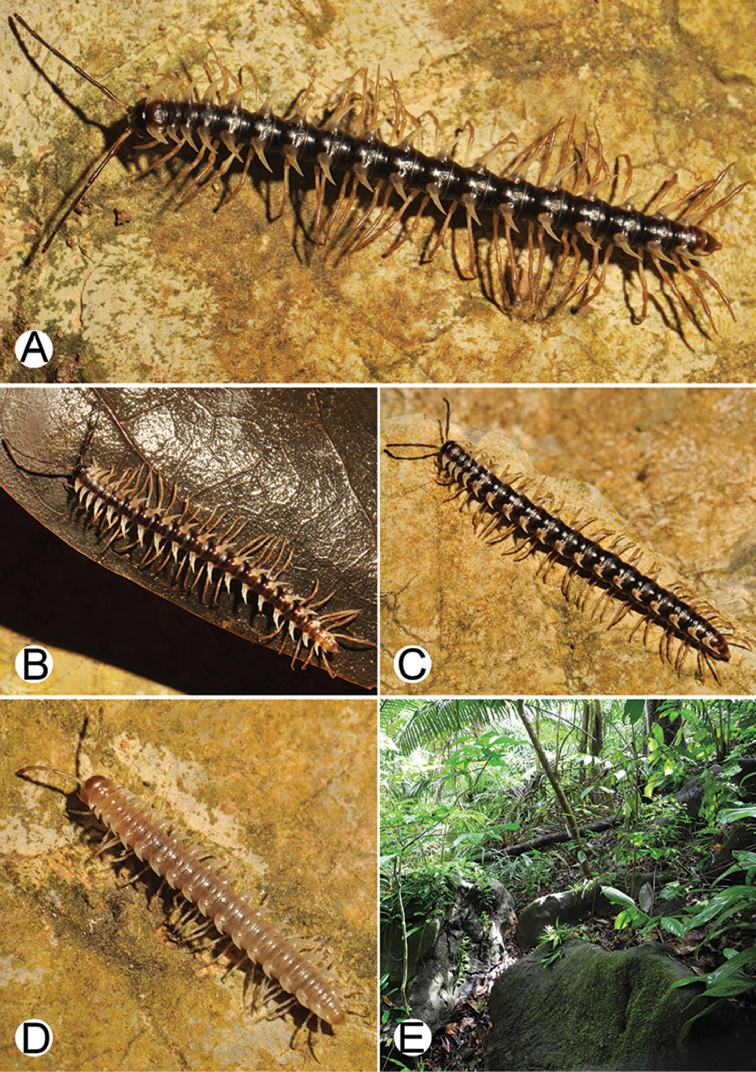
Photographs of live *Desmoxytes corythosaurus* sp. n. and habitat **A, B** male paratypes **C** female paratype **D** juvenile **E** habitat.

**Figure 24.**
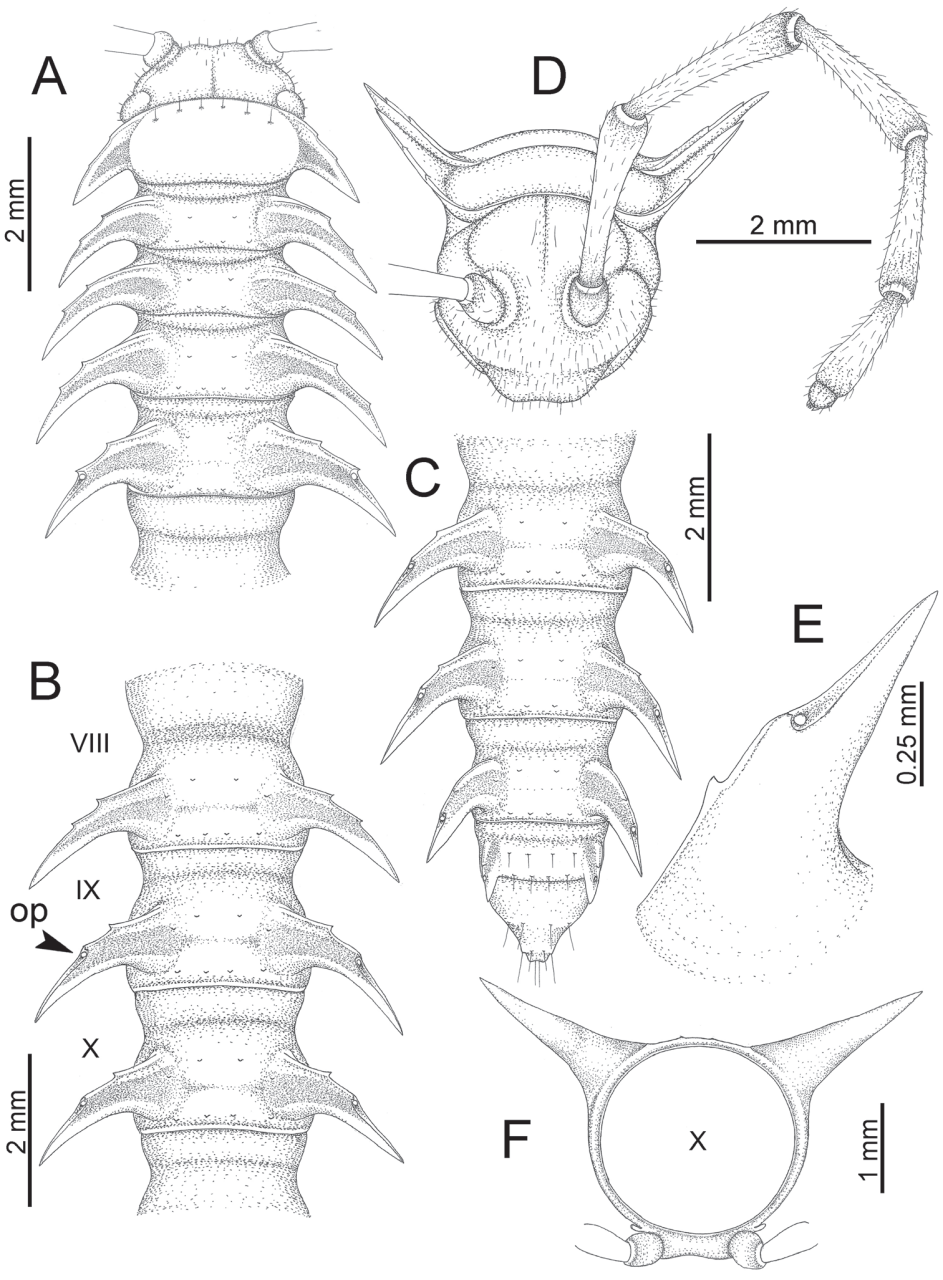
*Desmoxytes corythosaurus* sp. n. (male paratype). **A** anterior body part **B** body rings 8–10 (op = ozopore) **C** posteriormost body rings and telson **D** head and antenna **E** paraterga of ring 10 **F** body ring 10.

**Figure 25.**
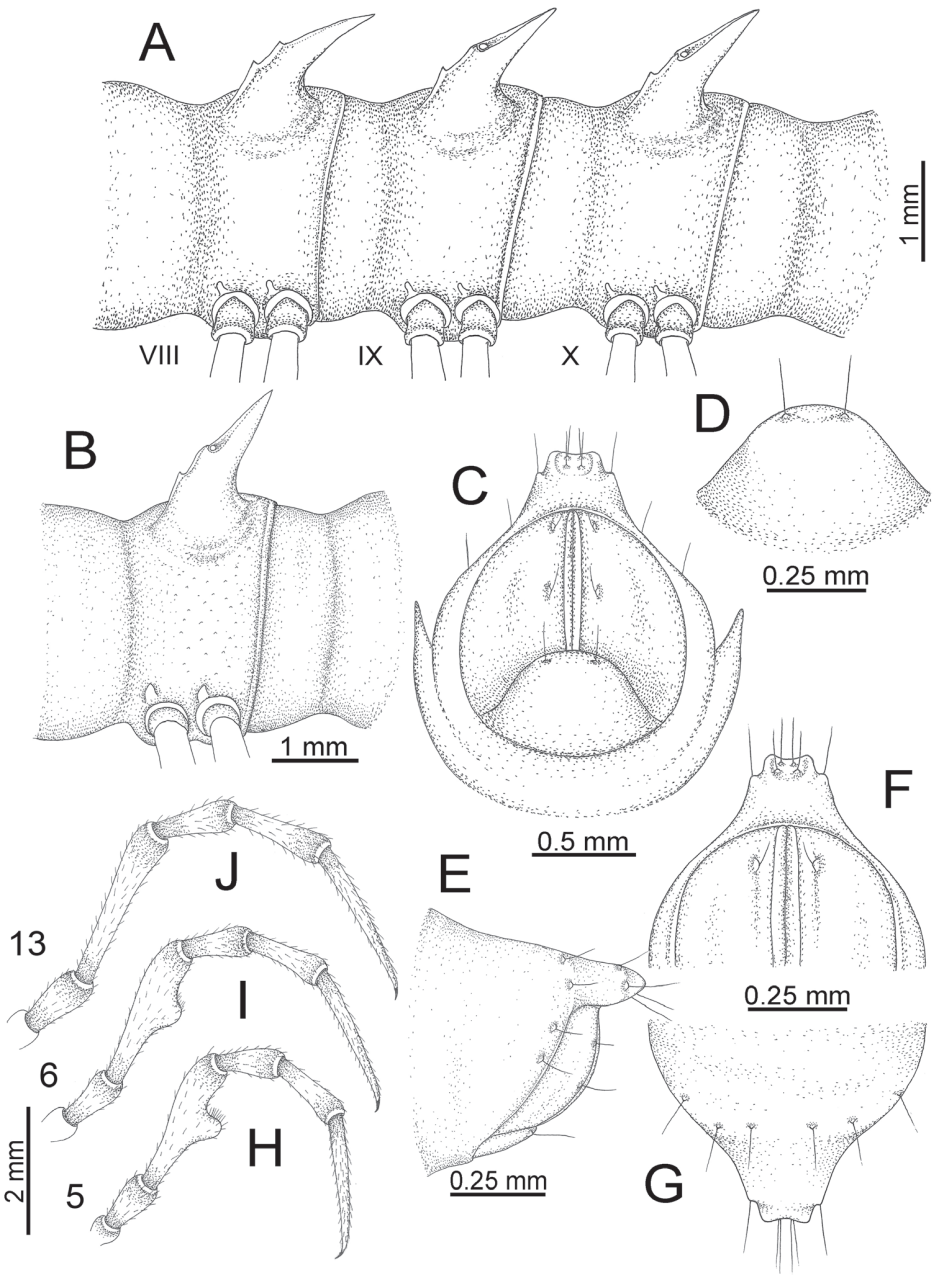
*Desmoxytes corythosaurus* sp. n. (male paratypes). **A** body rings 8–10 **B** sculpture of ring 10 **C, E** last ring and telson **D** hypoproct **F, G** epiproct **H** male leg 5 (right) **I** male leg 6 (right) **J** male leg 13 (right).

**Figure 26.**
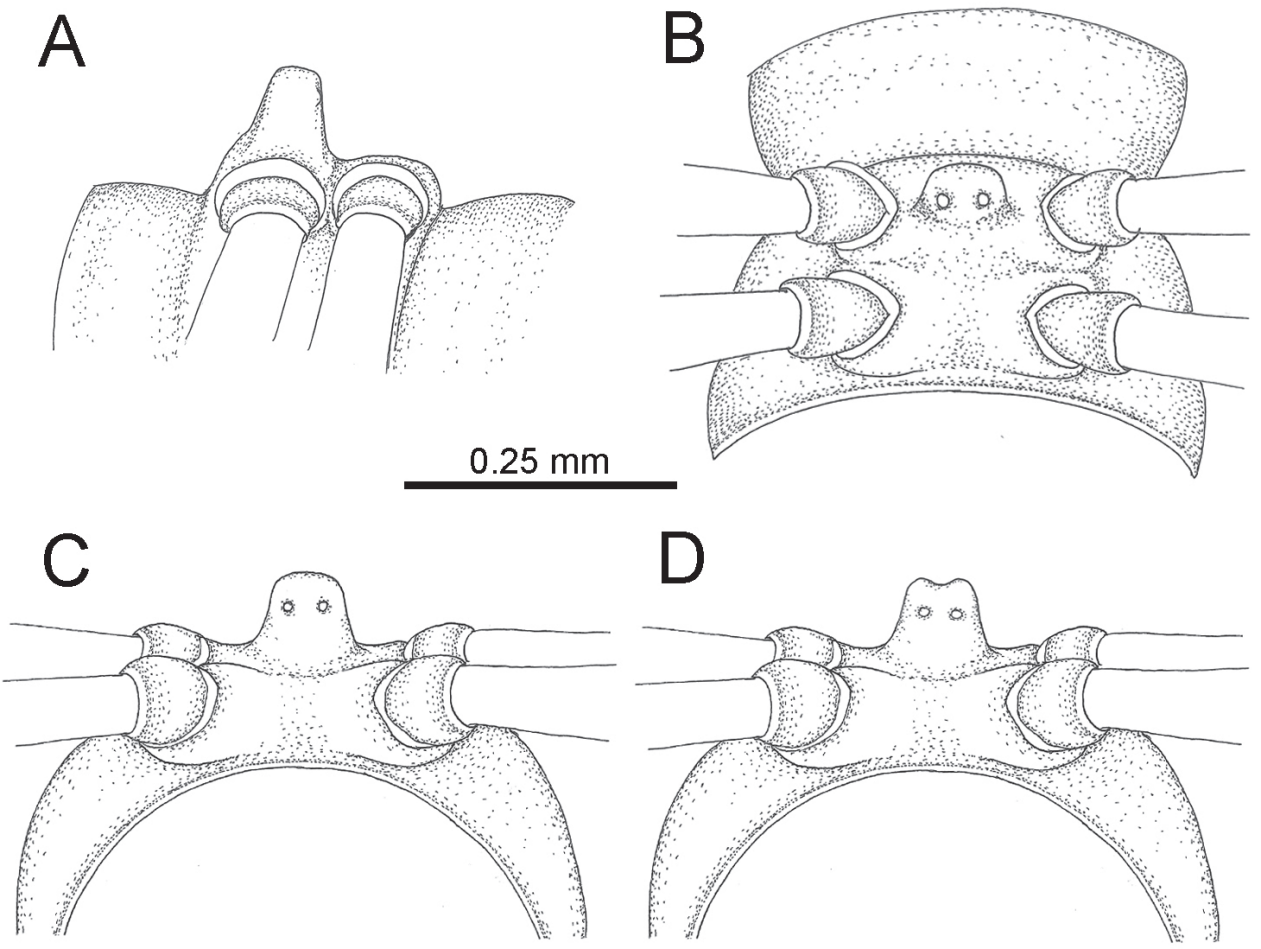
*Desmoxytes corythosaurus* sp. n. – sternal lobe between male coxae 4. **A** lateral view (male holotype) **B** ventral view (male holotype) **C** caudal view (male holotype) **D** caudal view (male paratype).

**Figure 27.**
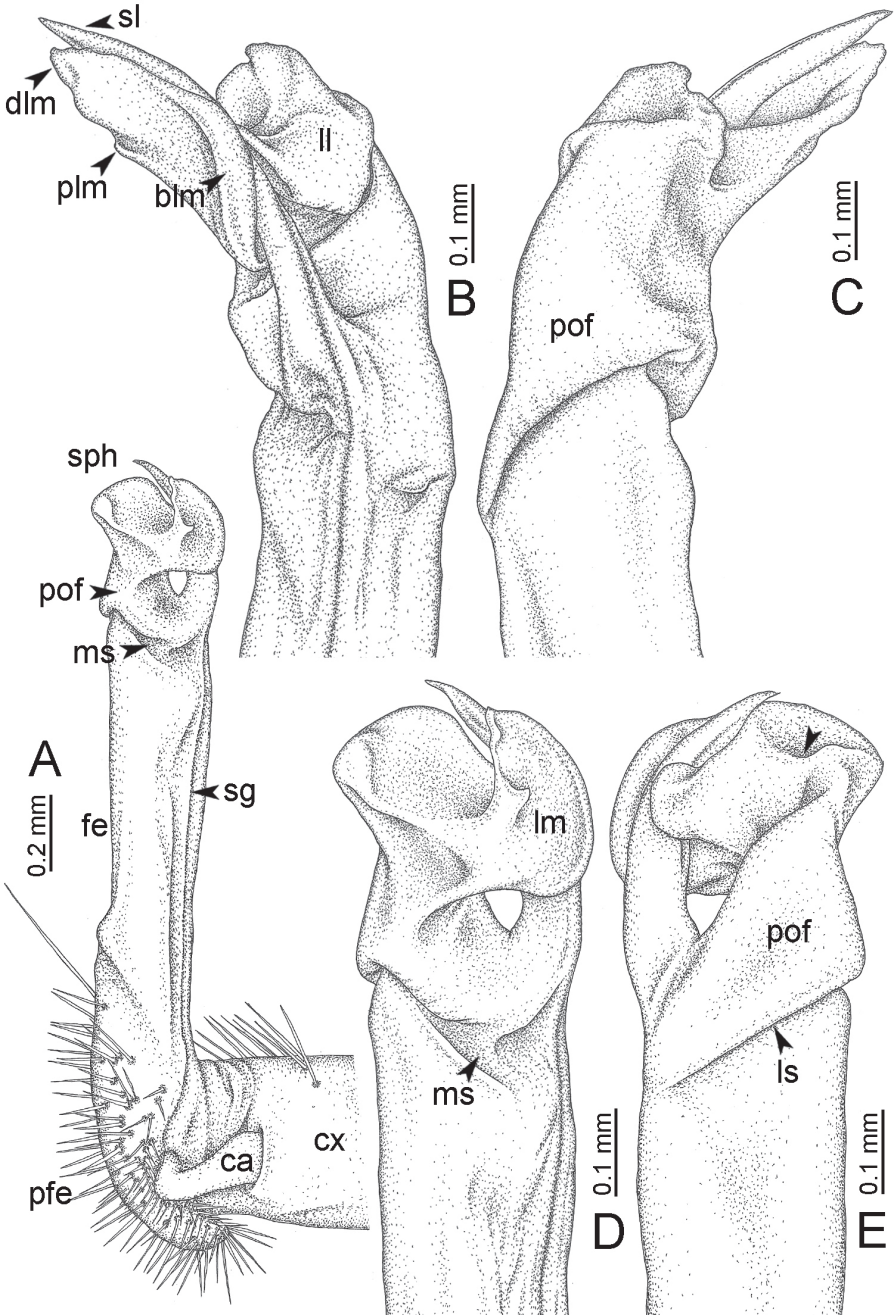
*Desmoxytes corythosaurus* sp. n. (paratype) – right gonopod. **A** mesal view **B** dorsal view **C** ventral view **D** submesal view **E** lateral view (arrow = furrow).

**Figure 28.**
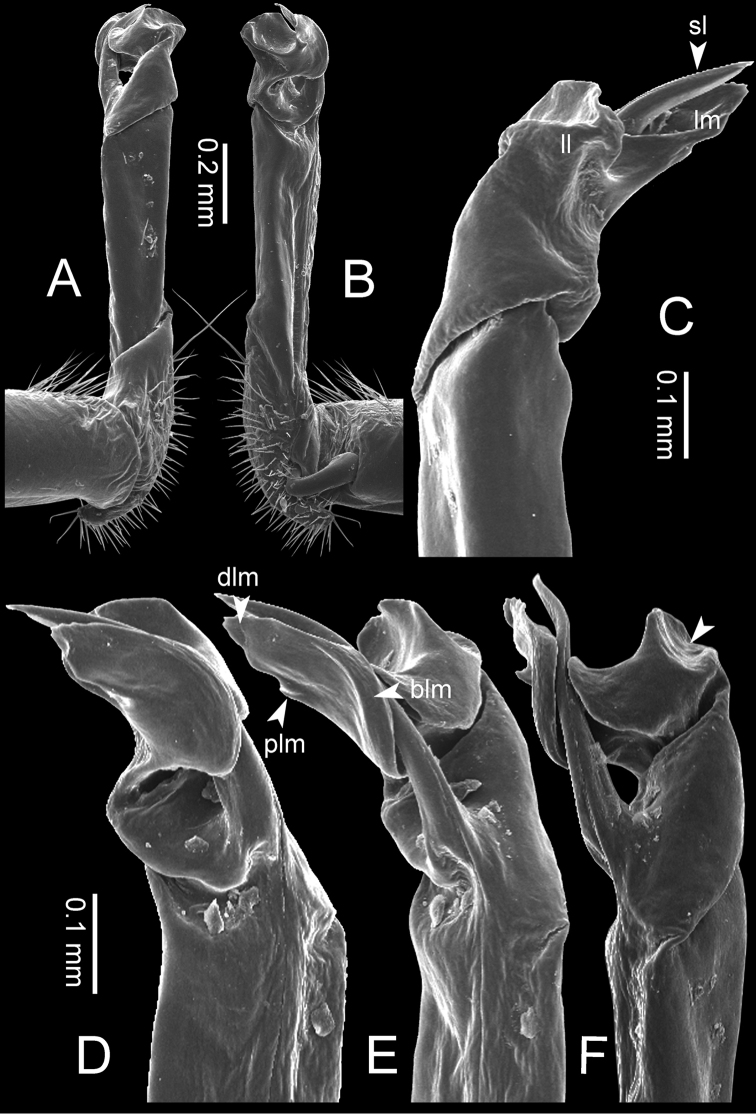
*Desmoxytes corythosaurus* sp. n. (paratype) – right gonopod. **A** lateral view **B** mesal view **C** ventral view **D** subdorsal view **E** dorsal view **F** subdorsal view (arrow = furrow).

Figs 23 , 24 , 25 , 26 , 27 , 28

########### Holotype.

Male (CUMZ), THAILAND, Surat Thani Province, Phanom District, Ban Song Phi Nong, huge limestone mountain, 8°50'51"N, 98°44'16"E, ca. 74 m a.s.l., 7 August 2014, leg. C. Sutcharit, R. Srisonchai and ASRU members.

########### Paratypes.

5 males, 1 female (CUMZ), 1 male (ZMUC), same data as holotype.

########### Further specimens, 

**not paratypes, all from THAILAND, Surat Thani Province, Phanom District**: 1 male, 2 females (CUMZ), Wat Tham Wararam, 8°53'07"N, 98°40'01"E, ca. 51 m a.s.l., 5 August 2014, leg. C. Sutcharit, R. Srisonchai and ASRU members. 1 male, 1 female (CUMZ), Wat Tham Wararam, 8°53'07"N, 98°40'01"E, ca. 51 m a.s.l., 6 August 2016, leg. C. Sutcharit, R. Srisonchai and ASRU members. 1 male, 1 broken female (CUMZ), Wat Tham Wararam, 8°53'07"N, 98°40'01"E, ca. 51 m a.s.l., 1 August 2017, leg. C. Sutcharit and A. Pholyotha and ASRU members. 2 males, 1 female (CUMZ), Tham Nam Lod, near Anurak Community Lodge Resort, big limestone mountain, 8°52'43"N, 98°40'50"E, ca. 51 m a.s.l., 7 August 2014, leg. C. Sutcharit, R. Srisonchai and ASRU members.

########### Etymology.

The name is a Latin noun in apposition, referring to the similarity of the lamina lateralis (ll) to the crest-liked structure on the head of the dinosaur genus *Corythosaurus*.

########### Diagnosis.

Body dark brown to black; paraterga with brown or black patches contrasting against whitish at base and along the edges; metaterga 2–18 with rows of 2+2 anterior and 2+2 posterior tubercles. Similar in these respects to *D. terae*, but differing by having paraterga much longer and higher; sternal lobe between male coxae 4 subtrapeziform; male femora 5 and 6 modified; lamina lateralis (ll) apically crest-like; distal lobe with one very long lamella; indentation between distal lobe (dlm) and broad lobe (blm) inconspicuous.

########### Description.

SIZE: Length 32–33 mm (male), 33–34 mm (female); width of midbody metazona ca. 2.3 mm (male), 3.0 mm (female). Width of head < collum < body ring 2 < 3 = 4 < 5–16, thereafter body gradually tapering towards telson.

COLOUR (Fig. 23A–D): In life with body brownish black; antenna and head dark brown (except distal part of antennomere 7 and antennomere 8 whitish); metaterga and surface below paraterga brownish black; paraterga whitish, dorsally with brown patches in the middle; legs, sterna and epiproct brown; a few basal podomeres sometimes whitish.

ANTENNAE (Fig. 24D): Moderately long and slender, reaching to the posterior end of body ring 6 (male) and 5 (female) when stretched dorsally.

COLLUM (Fig. 24A): With 1 transverse anterior row of 3+3 setae; paraterga of collum low, elevated at ca. 10°–15°, directed caudolaterad, with two distinct notches on lateral margin.

TEGUMENT: Moderately shining and smooth; prozona finely shagreened; collum, metaterga, surface below paraterga, sterna and epiproct smooth; lateral surface at base of paraterga with wrinkles.

METATERGA (Fig. 24A–C): With 2 transverse rows of setae and tubercles, mostly inconspicuous; metaterga 2–18 with 2+2 anterior and 2+2 posterior tubercles; metatergum 19 with 2+2 anterior and 2+2 posterior setae.

PARATERGA (Fig. 24E, F): Directed caudolaterad on body rings 2–17, elevated at ca. 30° (male) 25° (female); directed increasingly caudad on body rings 18 and 19; tip of paraterga in female slightlty curved caudad; anterior margin with 2 distinct notches, without a tiny denticle near the tip.

TELSON (Fig. 25C–G): Epiproct: tip subtruncate; lateral setiferous tubercles and apical tubercles inconspicuous. Hypoproct subtrapeziform; caudal margin round, with very small and inconspicuous setiferous tubercles.

STERNA (Fig. 26): Cross-impressions shallow. Sternal lobe between male coxae 4 subtrapeziform; tip usually slightly round (in some specimens subemarginate), base broad and stout.

LEGS (Fig. 25H–J): Long and slender. Male femora 5 and 6 strongly humped ventrally in middle portion (hump of male femora 6 more developed than 5).

GONOPODS (Figs 27, 28): Coxa (cx) longer than prefemur. Cannula (ca) somewhat stout. Telopodite erect. Prefemur (pfe) ca. 2/3 as long as femur. Femur (fe) quite long and slender. Mesal sulcus (ms) and lateral sulcus (ls) conspicuous, very deep. Postfemur (pof) conspicuous, ventrally narrow and short. Solenophore (sph) well-developed: lamina lateralis (ll) conspicuous, apically crest-like, quite thin; anterolaterally with a wide and conspicuous furrow: lamina medialis (lm) well-developed; process (plm) very short; distal lobe (dlm) very long and broad, indentation between distal lobe (dlm) and broad lobe (blm) inconspicuous; broad lobe (blm) moderately thick. Solenomere (sl) long.

########### Distribution and habitat.

This species is known only from a narrow distribution range in Phanom district. We consider *D. corythosaurus* sp. n. to be endemic for Surat Thani Province, Thailand.

########### Remarks.

The shape of the sternal lobe between male coxae 4 is variable: the tip is emarginate in the populations from Wat Tham Wararam and Tham Nam Lod, whereas specimens from the type locality have a truncate tip. The new species shares a similar shape of gonopodal solenophore with *D. terae*.

This species is almost impossible to find at first glance. We collected all specimens that were found on the humid rock walls by using flashlight. It blended in perfectly with the brown or black rock, this way probably avoiding being detected by predators.

########### Coexisting species.

*Desmoxytes cervina* (brown morph) at Ban Song Phi Nong, was collected from rock walls, same habitat as the new species.

########## Desmoxytes delfae

Taxon classificationAnimaliaPolydesmidaParadoxosomatidae

(Jeekel, 1964)

**Figure 29.**
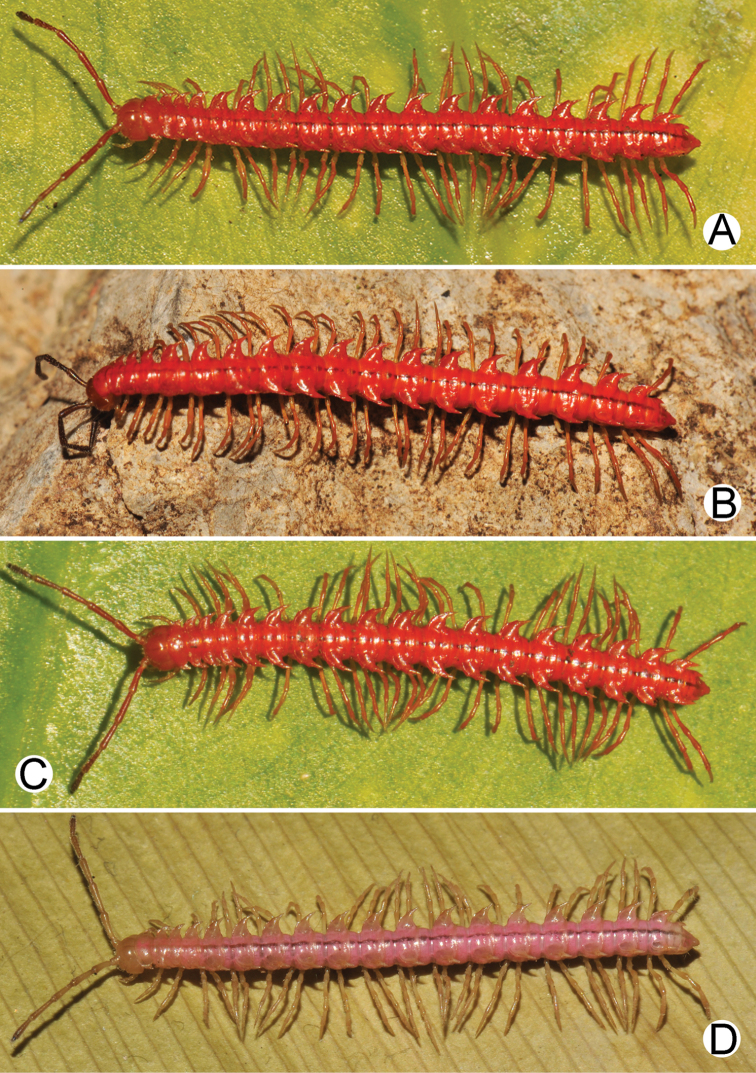
Photographs of live *Desmoxytes delfae* (Jeekel, 1964) – males. **A** specimen from Khao Chi Chan Bureau of Monks **B** specimen from Tham Tone Din **C** specimen from Wat Tham Sue (Tiger Cave) **D** early adult from La-ngu Subdistrict.

**Figure 30.**
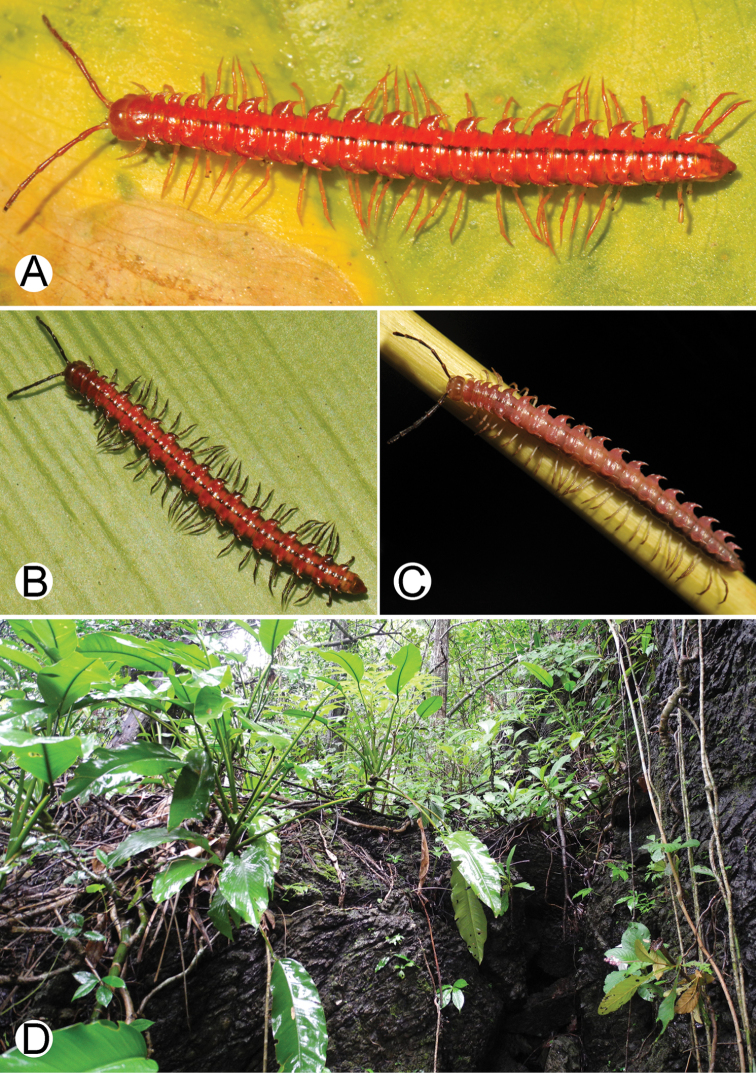
Photographs of live *Desmoxytes delfae* (Jeekel, 1964) and habitat. **A–C** specimens from Tham Khan Ti Phol **A** female **B** old female **C** newly moulted adult female **D** habitat.

**Figure 31.**
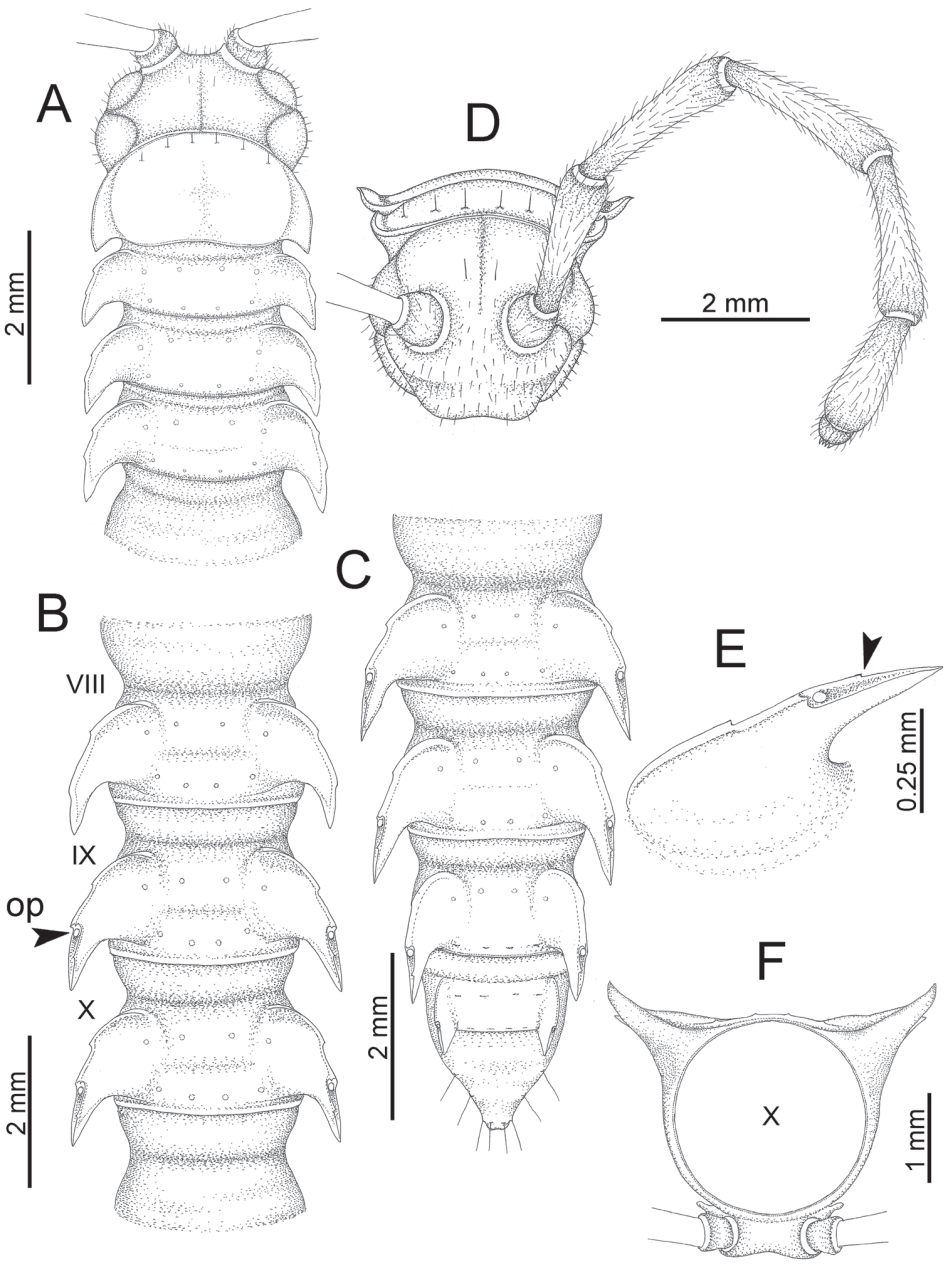
*Desmoxytes delfae* (Jeekel, 1964), specimen from Tham Khan Ti Phol. **A** anterior body part **B** body rings 8–10 (op = ozopore) **C** posteriormost body rings and telson **D** head and antenna **E** paraterga of ring 10 (arrow = tiny denticle) **F** body ring 10.

**Figure 32.**
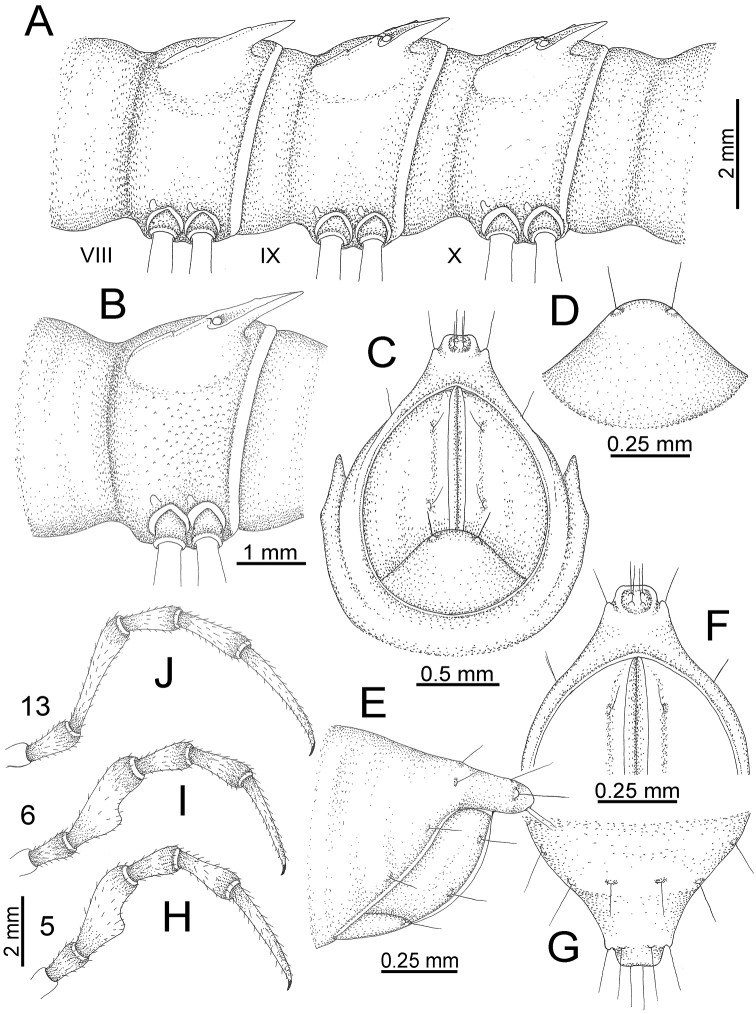
*Desmoxytes delfae* (Jeekel, 1964), specimens from Tham Khan Ti Phol. **A** body rings 8–10 **B** sculpture of ring 10 **C, E** last ring and telson **D** hypoproct **F, G** epiproct **H** male leg 5 (right) **I** male leg 6 (right) **J** male leg 13 (right).

**Figure 33.**
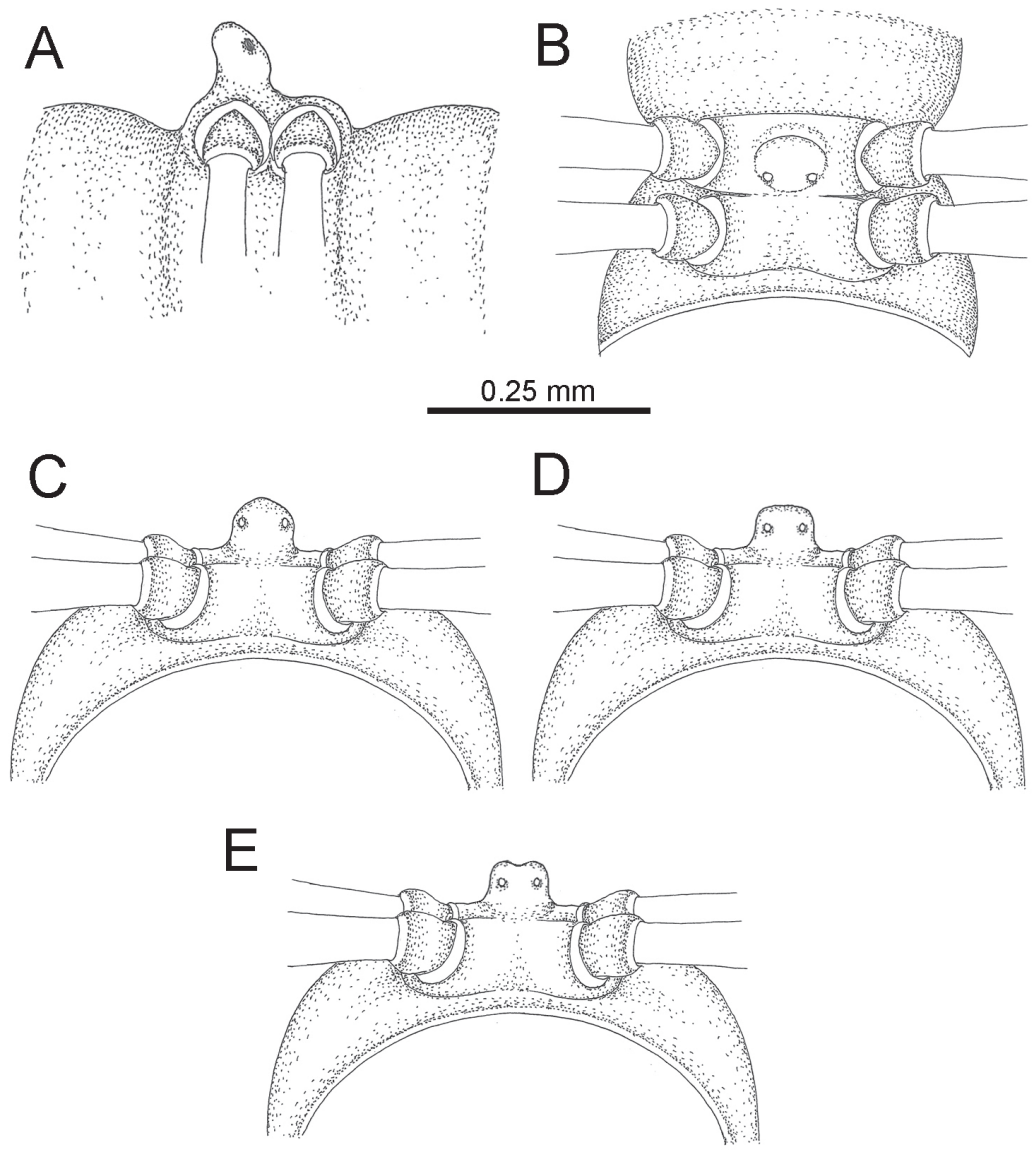
*Desmoxytes delfae* (Jeekel, 1964), specimens from Tham Khan Ti Phol – sternal lobe between male coxae 4. **A** lateral view **B** ventral view **C**, **D** caudal view **E** some specimens from Tham Khao Ting – caudal view.

**Figure 34.**
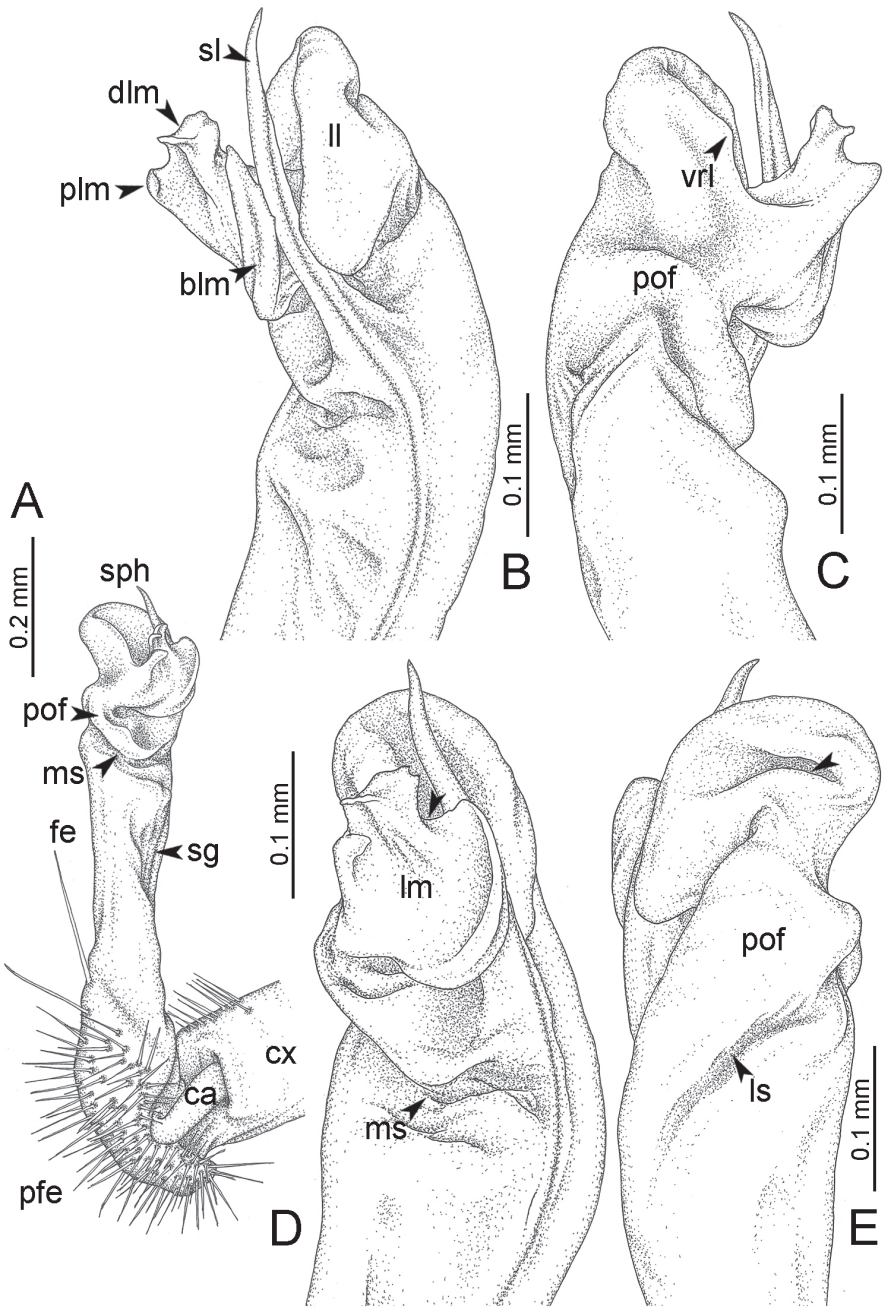
*Desmoxytes delfae* (Jeekel, 1964), specimen from Khao Chi Chan Bureau of Monks – right gonopod. **A** mesal view **B** dorsal view **C** ventral view **D** submesal view (arrow = indentation) **E** lateral view (arrow = furrow).

**Figure 35.**
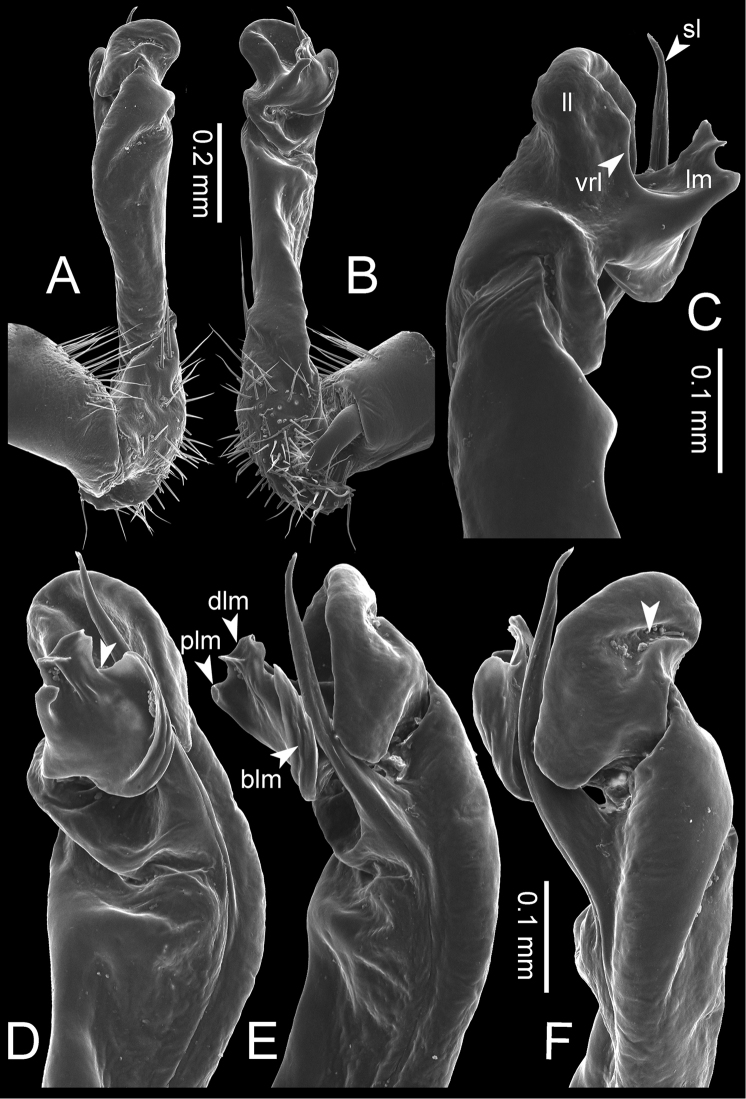
*Desmoxytes delfae* (Jeekel, 1964), specimen from Khao Chi Chan Bureau of Monks – right gonopod. **A** lateral view **B** mesal view **C** ventral view **D** subdorsal view (arrow = indentation) **E** dorsal view **F** subdorsal view (arrow = furrow).

Figs 29 , 30 , 31 , 32 , 33 , 34 , 35

Pratinus delfae Jeekel, 1964: 66; 1968: 51.
Desmoxytes delfae Jeekel, 1980a: 654. Golovatch and Enghoff 1994: 61. Enghoff 2005: 96. Decker 2010: 28. Nguyen and Sierwald 2013: 1241.
Desmoxytes rubra Golovatch & Enghoff, 1994: 54, syn. n. Enghoff 2005: 96. Decker 2010: 30. Nguyen and Sierwald 2013: 1242.

########### Material examined.

**Holotype.** Male (NHMUK), THAILAND, Bukit Besar, on leaves in clearing, late evening, 3 September 1901.

########### Paratypes.

1 male, 1 female (NHMUK), 1 male (NBC), THAILAND, Bukit Besar, Nawnchila, crawling on low foliage in clearing, 2500 feet a.s.l., 29 September 1901.

Holotype (*D. rubra*): Male (ZMUC), THAILAND, Satun Province, Thale Ban National Park, lowland rainforest, on vegetation and under bark, 6°42'N, 100°10'E, ca. 400 m a.s.l., 20 October 1991, leg. M. Andersen, O. Martin, N. Scharff.

Paratypes (*D. rubra*): 6 males, 5 females (ZMUC), same data as holotype.

########### Further specimens, 

**all from THAILAND, Krabi Province**: 1 female (CUMZ), Mueang Krabi District, Huai To Waterfall, 8°14'27"N, 98°54'51"E, ca. 110 m a.s.l., 16 January 2006, leg. S. Panha and ASRU members. 1 male missing right gonopod (CUMZ), Mueang Krabi District, Wat Tham Sue (Tiger Cave), valley behind Tiger Cave, 8°07'39"N, 98°55'27"E, ca. 85 m a.s.l., 7 October 2009, leg. S. Panha and ASRU members. 1 male (CUMZ), Mueang Krabi District, Wat Tham Sue (Tiger Cave), valley behind Tiger Cave, 8°07'39"N, 98°55'27"E, ca. 85 m a.s.l., 28 April 2014, leg. C. Sutcharit, R. Srisonchai and ASRU members. 5 males, 2 females (CUMZ), Mueang Krabi District, Wat Tham Sue (Tiger Cave), valley behind Tiger Cave, 8°07'39"N, 98°55'27"E, ca. 85 m a.s.l., 9 July 2017, leg. C. Sutcharit, R. Srisonchai and ASRU members. 1 male, 1 female (CUMZ), Mueang Krabi District, Wat Tham Sue (Tiger Cave), valley behind Tiger Cave, 8°07'39"N, 98°55'27"E, ca. 85 m a.s.l., 25 July 2017, leg. C. Sutcharit, R. Srisonchai and ASRU members.

**Nakhon Si Thammarat Province**: 2 broken males (CUMZ), Nopphitam District, Krung Ching Waterfall, 8°43'27"N, 99°40'07"E, ca. 171 m a.s.l., 28 October 2006, leg. S. Panha and ASRU members. 1 female (CUMZ), Thung Song District, Talod Cave Park (Talod Cave), 8°09'32"N, 99°40'42"E, ca. 73 m a.s.l., 5 July 2017, leg. C. Sutcharit, R. Srisonchai and ASRU members. 2 males, 1 female (CUMZ), Thung Song District, Weruwan Bureau of Monks (Tham Rad), 8°02'48"N, 99°43'43"E, ca. 82 m a.s.l., 5 July 2017, leg. C. Sutcharit, R. Srisonchai and ASRU members.

**Phatthalung Province**: 2 males,1 female (CUMZ), Khao Chaison District, Khao Chaison Hot Spring, 7°26'59"N, 100°07'48"E, ca. 37 m a.s.l., 12 January 2009, leg. S. Panha and ASRU members. 1 broken male missing gonopods, 2 females (CUMZ), Khao Chaison District, Khao Chaison Hot Spring, 7°26'59"N, 100°07'48"E, ca. 37 m a.s.l., 13 January 2009, leg. C. Sutcharit and ASRU members. 1 female (CUMZ), Khuan Khanun District, Tham Wang Thong, 7°40'57"N, 100°00'58"E, ca. 45 m a.s.l., 12 January 2009, leg. C. Sutcharit and ASRU members. 3 males, 1 female (CUMZ), Khuan Khanun District, Tham Wang Thong, 7°40'57"N, 100°00'58"E, ca. 45 m a.s.l., 6 July 2017, leg. C. Sutcharit and ASRU members. 3 males, 3 females (CUMZ), Kong Ra District, Khao Phaya Hong, 7°27'46"N, 99°57'50"E, ca. 55 m a.s.l., 6 July 2017, leg. C. Sutcharit, R. Srisonchai and ASRU members.

**Satun Province**: 1 male (ZMUC), Thale Ban National Park, lowland rainforest, 6°42'37"N, 100°10'09"E, ca. 93 m a.s.l., 15–18 October 2003, leg. ATOL expedition (ZMUC staff). 7 males, 2 females (CUMZ), Thung Wa District, Tham Khan Ti Phol, 7°05'11"N, 99°47'53"E, ca. 82 m a.s.l., 8 July 2017, leg. C. Sutcharit, R. Srisonchai and ASRU members. 26 males, 4 females (CUMZ), 2 males, 1 female (ZMUC), 1 male (ZMUM), 1 male (NHMW) ,1 male (NHMUK), Thung Wa District, Tham Khan Ti Phol, 7°05'11"N, 99°47'53"E, ca. 82 m a.s.l., 7 July 2017, leg. C. Sutcharit, R. Srisonchai and ASRU members. 2 males, 2 females (CUMZ), Khuan Don District, Thale Ban National Park, Tham Tone Din (Tone Din Cave), 6°43'35"N, 100°09'45"E, ca. 154 m a.s.l., 7 July 2017, leg. S. Sutcharit, R. Srisonchai and ASRU members. 1 female (CUMZ), La-ngu District, limestone mountain near Ao Noon (Mu Ko Petra National Park), 6°50'17"N, 99°45'41"E, ca. 37 m a.s.l., 31 August 2015, leg. C. Sutcharit, R. Srisonchai and ASRU members. 2 males (CUMZ), La-ngu District, limestone mountain near La-ngu Subdistrict, 6°53'41"N, 99°46'49"E, ca. 18 m a.s.l., 17 July 2017, leg. P. Danaisawadi.

**Songkhla Province**: 1 male missing gonopods (CUMZ), Hat Yai District, Ton Nga Chang Waterfall, 6°56'53"N, 100°14'03"E, ca. 157 m a.s.l., 12 January 2009, leg. S. Panha and ASRU members. Many broken and mixed specimens (CUMZ), Hat Yai District, Ton Nga Chang Waterfall, 6°56'53"N, 100°14'03"E, ca. 157 m a.s.l., 13 December 2011, leg. S. Panha and ASRU members. 4 males, 4 females (CUMZ), Sa Dao District, Khao Wong Pra Chan Bureau of Monks, 6°42'38"N, 100°16'34"E, ca. 100 m a.s.l., 7–8 July 2017, leg. C. Sutcharit, R. Srisonchai and ASRU members. 2 females (CUMZ), Sa Dao District, Tham Nang Phaya Lued Kao Bureau of Monks, 6°44'26"N, 100°15'27"E, ca. 124 m a.s.l., 7 July 2017, leg. C. Sutcharit, R. Srisonchai and ASRU members.

**Surat Thani Province**: 2 males, 1 female (CUMZ), Wiang Sa District, Khiri Rat Pattana Bureau of Monks (Wat Khao Poon), 8°31'38"N, 99°22'59"E, ca. 49 m a.s.l., 4 July 2017, leg. C. Sutcharit, R. Srisonchai and ASRU members. 2 males, 1 female (CUMZ), Tham Phannara District, Wat Tham Kanlaya Namit, 8°30'49"N, 99°22'53"E, ca. 62 m a.s.l., 4 July 2017, leg. C. Sutcharit, R. Srisonchai and ASRU members.

**Trang Province**: 3 males, 3 females, 2 juveniles (CUMZ), Palian District, Tham Khao Ting, 7°09'31"N, 99°48'10"E, ca. 42 m a.s.l., 31 August 2015, leg. C. Sutcharit, R. Srisonchai and ASRU members. 9 males, 2 females (CUMZ), Palian District, Tham Khao Ting, 7°09'31"N, 99°48'10"E, ca. 42 m a.s.l., 8 July 2017, leg. C. Sutcharit, R. Srisonchai and ASRU members.

**Yala Province**: 1 male (ZMUC), Bang Lang National Park, lowland rainforest, 6°4'N, 101°11'E, ca. 400 m a.s.l., 20 October 1991, leg. M. Andersen, O. Martin, N. Scharff.

########### Diagnosis.

Differs from congeners in the combination of the following characters; sternal lobe between male coxae 4 thick and stout, round/ subtrapeziform/ subrectangular; lamina lateralis (ll) swollen, round crest-like, laterally with a distinct and wide furrow, mesally with deep subsided surface; process (plm) of lamina medialis short, distally curving dorsad, tip blunt.

########### Type locality.

Thailand, Bukit Besar [Thale Ban National Park, Khuan Don District, Satun Province].

The redescription hereunder is modified from Jeekel (1964), and Golovatch and Enghoff (1994). We ‘harmonised’ descriptions of all morphological characters and added some morphological characteristics from additional collected specimens.

########### Redescription.

SIZE: Length 21–24 mm (male), 23–27 mm (female); width of midbody metazona ca. 1.8 mm (male), 2.3 mm (female). Width of head ≥ collum < body ring 2 < 3 < 4 < 5–16, thereafter body gradually tapering towards telson.

COLOUR (Figs 29, 30A–C): In life body bright orange or brownish orange (newly moulted adults pale pink); head, paraterga, surface below paraterga and epiproct yellowish orange; metaterga bright orange; antenna brownish orange or blackish orange (except antennomere 7 blackish, distal part of antennomere 7 and antennomere 8 whitish); legs brownish orange or brownish black; a few basal podomeres whitish yellow; sterna whitish yellow or orange; prozona and metazona (metaterga) with wide black stripe, conspicuous on rings 4–19. Colour in alcohol: after 116 years changed to white, after 26 years changed to brownish white.

ANTENNAE (Fig. 31D): Moderately long and slender, reaching to body ring 6 (male) and 4–5 (female) when stretched dorsally.

COLLUM (Fig. 31A): With 1 transverse anterior row of 3+3 setae; paraterga of collum low, almost horizontal, directed almost caudad, with two setiferous notches on lateral margin.

TEGUMENT: Strongly shining and smooth; prozona finely shagreened; collum, metaterga, paraterga, sterna and epiproct smooth; surface below paraterga coarsely microgranulate.

METATERGA (Fig. 31A–C): With 2 transverse rows of small tubercles; metaterga 2–19 with 2+2 anterior and 2+2 posterior tubercles.

PARATERGA (Fig. 31E, F): Directed caudolaterad on body rings 2–17, elevated ca. 10°–20° above the horizontal plane (ca. 20° in male, ca. 10° in female), directed increasingly caudad on body rings 18 and 19; anterior margin with 2 distinct notches, on lateral margin of body rings 9, 10, 12, 13, 15–18 with tiny denticle near the tip.

TELSON (Fig. 32C–G): Epiproct: tip truncate; lateral setiferous tubercles usually conspicuous (in some specimens inconspicuous); apical tubercles inconspicuous. Hypoproct subtriangular; caudal margin round and narrow, with inconspicuous setiferous tubercles.

STERNA (Fig. 33): Cross-impressions shallow. Sternal lobe between male coxae 4 round/ subtrapeziform/ subrectangular (varies within population); erect, stout, thick, and broad when seen in ventral view; tip subtruncate/ round/ emarginate (varies within population).

LEGS (Fig. 32H–J): Long and slender. Male femora 5 and 6 strongly humped ventrally in middle portion.

GONOPODS (Figs 34, 35): Coxa (cx) longer than prefemur. Cannula (ca) long and slender. Prefemur (pfe) ca. 2/3 as long as femur. Femur (fe) quite long. Mesal sulcus (ms) and lateral sulcus (ls) conspicuous, deep and wide. Postfemur (pof) conspicuous, ventrally narrow, and short. Solenophore (sph) well-developed: lamina lateralis (ll) swollen, round crest-like, laterally with a distinct furrow, mesally with deep subsided surface; ventral ridge (vrl) conspicuous: lamina medialis (lm) well-developed; process (plm) short, distally curving dorsad, tip blunt; distal lobe (dlm) distally with two lamellae, mesal lamella thin and smaller than lateral one; broad lobe (blm) somewhat thick, distinctly separated from distal lobe (dlm) by a deep and wide indentation. Solenomere (sl) long.

########### Distribution and habitat.

*Desmoxytes delfae* is known from several provinces in southern Thailand. All new specimens were collected by us from limestone habitats (the recorded locations in previous papers are also limestone); most specimens were seen crawling on leaf litter and climbing on branches of trees (Fig. 30D).

The type locality, Bukit Besar, may be part of what is now Thale Ban National Park in Khuan Don District, Satun Province, and refers to the big mountain which is probably Khao Chin (ca. 2500 ft. or 756 m). Specimens collected by us from Thale Ban National Park may thus be topotypes. The other paratype locality (Bukit Besar, Nawnchila) has not been exactly located, but it is probably near Thale Ban National Park (Thailand–Malaysia border), possibly close to the type locality.

We assume that *D. delfae* is distributed mainly in Thailand and possibly south to Malaysia near the Thailand–Malaysia border.

This species was reported as endemic for Thailand (Decker 2010), and we would agree that it should be regarded as endemic for the southern part of Thailand due to its narrow distribution. However, two males of *D. delfae* were reported from Khaosok National Park in west of Surat Thani Province by Decker (2010); it is probable that the specimens from this location belong to another species because all the specimens of *D. delfae* which we have seen are from an area in south Thailand (Krabi, east of Surat Thaini, Nakhon Si Thammarat, Phatthalung, Satun, Songkhla, and Yala Provinces). Furthermore, Decker (2010) also identified one male, which was collected from Nakhon Si Thammarat Province (Lan Saka District, Khao Luang National Park, near Karom Waterfall) as *D. rubra*. Re-examination of those specimens in MHNG is necessary to evaluate the distribution of this species.

########### Remarks.

The remarkable and vivid bright orange colouration is clearly aposematic.

Golovatch and Enghoff (1994) distinguished *D. rubra* from *D. delfae* based on *D. rubra* having 1+1 anterior and 2+2 posterior metatergal tubercles, the mid-dorsal (axial) line traceable, the sternal lobe between male coxae 4 roundly subtriangular and lamina medialis (lm) more strongly produced mesally than dorsally. After examination of all type material and new specimens of both *D. delfae* and “*D. rubra*” collected by us, we found that:

– There is a variation in the size of tubercles on metaterga (metaterga with two rows of 2+2 anterior and 2+2 posterior tubercles, lateral tubercles of anterior row in some specimens (tiny and very inconspicuous).

– All specimens are without mid-dorsal line.

– The sternal lobe between male coxae 4 is highly variable in shape, even within populations, as round/ subtrapeziform/ subrectangular; we found this variation in both *D. delfae* and “*D. rubra*” specimens. Its tip also varies as subtruncate/ round/ emarginate.

– SEM images clearly show that specimens of these two nominal species have identical gonopods, especially in details of lamina lateralis and lamina medialis.

Due to this variation, we have synonymised *D. rubra* under *D. delfae*.

Jeekel (1964) described this species as lacking a tiny denticle near the tip of paraterga on the lateral margin of rings 9, 10, 12, 13, 15–18, and collum as having 2 rows of 3+3 conspicuous setae (anterior row) and 1?+1? inconspicuous tubercles (posterior row). All specimens studied by us have a tiny denticle near the tip (conspicuous in some specimens, inconspicuous in others), and we regard this character as variable within populations.

For *D. rubra*, Golovatch and Enghoff (1994) described the colour of alcohol-preserved specimens as bright pinkish, that of living specimens as bright red, collum with 3 rows of setae (anterior conspicuous, intermediate and posterior inconspicuous), mid-dorsal line traceable. Based on the re-examination of type material of *D. rubra* and examination of newly collected specimens we have found that *D. rubra* (= *D. delfae*) exhibits:

– Specimens in life with bright orange colouration, newly moulted adult stage pinkish or pinkish orange, late adult stage reddish orange or dark orange. As Golovatch and Enghoff (1994) reported that living specimens have a bright red colour, it is possible that the type specimens of *D. rubra* were collected at late adult stage (red = reddish orange?).

– Collum with one row of setae (3+3 anterior setae), intermediate and posterior rows absent. Therefore, we here report collum with only one row of setae (3+3 anterior setae).

– All specimens without mid-dorsal line.

– Sternal lobe between male coxae 4 varies within population, round/ subtrapeziform/ subrectangular.

As we mentioned above, this species shows high variability in morphology, e.g., colour, rows of setae on collum, size of metatergal tubercles, occurrence of a tiny denticle near tip of paraterga, shape of sternal lobe between male coxae 4. All variations are typically present within a population. Although there are deviations in several morphological characters, interestingly, gonopod characters of all specimens are quite stable, looking exactly the same in details.

########### Coexisting species.

*Desmoxytes cervina* in several places, *D. terae* at Tham Tone Din, *D. flabella* sp. n. at Tham Khao Ting and Tham Khan Ti Phol.

########## Desmoxytes des

Taxon classificationAnimaliaPolydesmidaParadoxosomatidae

Srisonchai, Enghoff & Panha, 2016

Fig. 36

Desmoxytes des Srisonchai, Enghoff & Panha, 2016: 94.

########### Material examined. Holotype.

Male (CUMZ), THAILAND, Chiang Mai Province, Fang District, Doi Angkhang, near Royal Agricultural Station, 19°54'26"N, 99°02'26"E, ca. 1426 m a.s.l., 12 August 2014, leg. N. Likhitrakarn.

########### Paratypes.

2 males, 2 females (CUMZ), same data as holotype. 1 male, 2 females (CUMZ), THAILAND, Chiang Mai Province, Chiang Dao District, Wat Tham Krab, 19°33'32"N, 99°03'47"E, ca. 622 m a.s.l., 25 October 2015, leg. C. Sutcharit, R. Srisonchai, T. Seesamut and A. Pholyotha.

########### Type locality.

THAILAND, Chiang Mai Province, Fang District, Doi Angkhang, near Royal Agricultural Station.

########### Diagnosis.

Differs from all other *Desmoxytes* species by the combination of the following characters: paraterga knife-like; lateral sulcus (ls) of gonopod shallow; lamina lateralis (ll) separated into two ridges by a deep and wide furrow; process (plm) of lamina medialis long and thin, lamellar, tip dentate or crenate; distal lobe (dlm) of lamina medialis quite long, tip directed ventroanteriad; caudal margin of hypoproct concave or truncate.

########### Redescription

**(updated from Srisonchai et al. 2016).** SIZE: Length 26–34 mm (male), 30–34 mm (female); width of midbody metazona ca. 2.5 mm (male), 3.0 mm (female). Width of head < collum < body ring 2 = 3 < 4 ≤ 5 < 6–16, thereafter body gradually tapering toward telson.

COLOUR: In life with body dark brown; paraterga bright pink; head, metaterga and surface below paraterga dark brown; antenna (except distal part of antennomere 7 and antennomere 8 whitish), leg, sterna and epiproct brown; a few basal podomeres pinkish brown or brown. Colour in alcohol: after two years changed to dark brown or pale brown.

ANTENNAE: Quite short, reaching to body ring 3 or 4 (male), and 3 (female) when stretched dorsally.

COLLUM: With 3 transverse rows of setiferous tubercles, 3+3 anterior, 1+1 posterior setae (posterior setae inconspicuous); paraterga of collum low, elevated at ca. 20°–30°, directed almost caudolaterad, with two inconspicuous setiferous notches on lateral margin.

TEGUMENT: Quite dull, but slightly shining; prozona finely shagreened; metaterga and surface below paraterga coarsely microgranulate; collum, paraterga, sterna and epiproct smooth.

METATERGA: With 2 transverse rows of setiferous tubercles; metaterga 2–18 with 2+2 anterior and 2+2 posterior tubercles; metatergum 19 with 2+2 anterior and 2+2 posterior setae.

PARATERGA: Directed caudolaterad on body rings 2–16, elevated at ca. 45° (male) 40° (female); directed increasingly caudad on body rings 17, 18 and 19; anterior margin with 2 distinct notches, on lateral margin of body rings 9, 10, 12, 13, 15–18 with tiny denticle near the tip.

TELSON: Epiproct: tip truncate; lateral setiferous tubercles conspicuous, small; apical tubercles inconspicuous. Hypoproct subtrapeziform; caudal margin quite concave or truncate, with inconspicuous setiferous tubercles.

STERNA: Cross-impressions shallow. Sternal lobe between male coxae 4 swollen, subrectangular, flat when seen in lateral view, tip truncate.

LEGS: Long and slender. Male femora 5 and 6 slightly stout, moderately humped ventrally in middle part.

**Figure 36.**
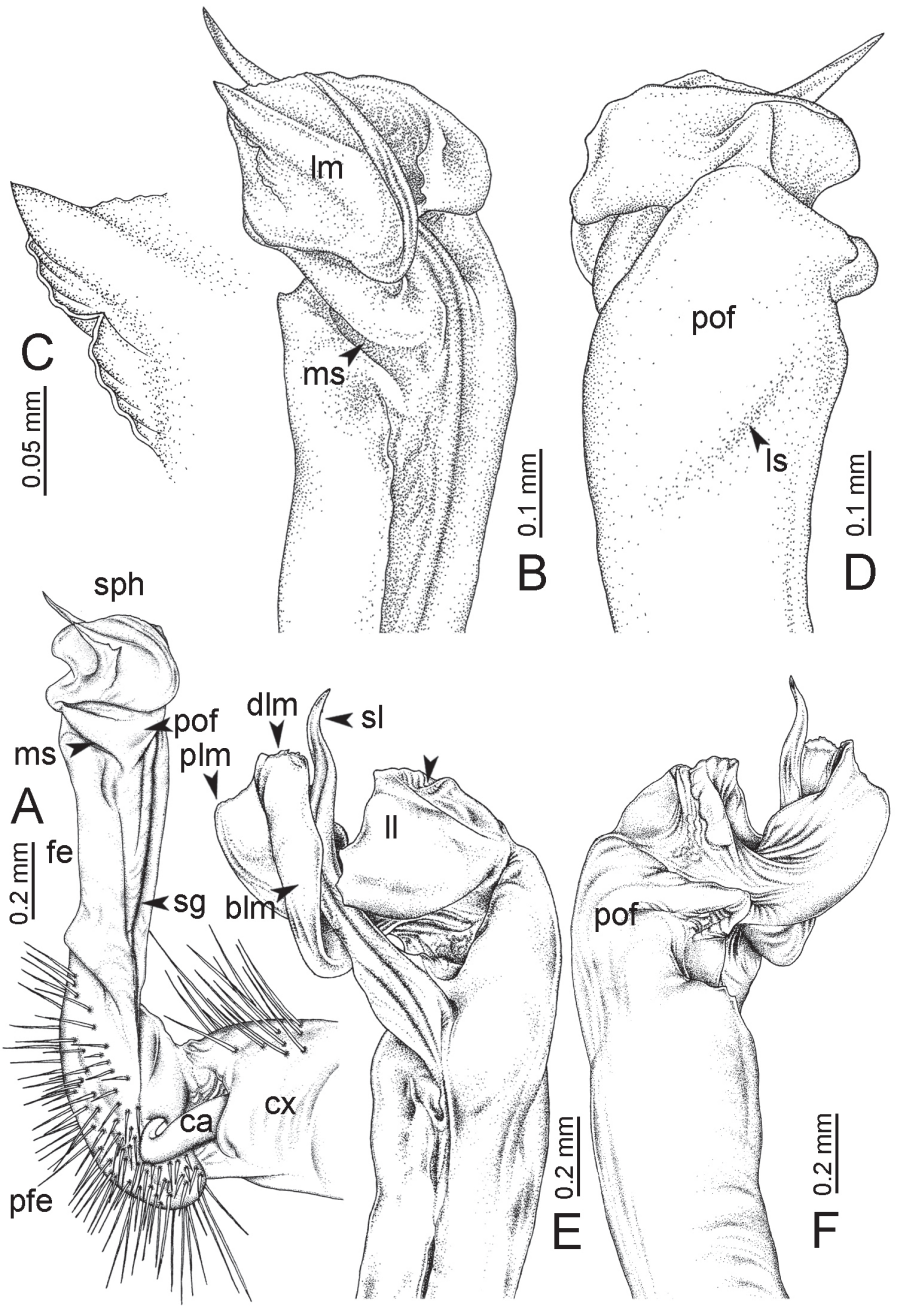
*Desmoxytes des* Srisonchai et al. 2016 (paratype) – right gonopod (modified from Srisonchai et al. 2016). **A** mesal view **B** submesal view **C** process (plm) of lamina medialis and distal lobe (dlm) of lamina medialis **D** lateral view **E** dorsal view (arrow = indentation) **F** ventral view.

GONOPODS (Fig. 36): Coxa (cx) longer than prefemur. Cannula (ca) slender. Telopodite quite stout. Prefemur (pfe) half to 2/3 as long as femur. Femur (fe) slightly stout. Mesal sulcus (ms) very deep and narrow, lateral sulcus (ls) shallow (= “poorly developed” in Srisonchai et al. 2016). Postfemur (pof) conspicuous, ventrally narrow. Solenophore (sph) well-developed: lamina lateralis (ll) swollen, laterally with a conspicuous, deep and wide furrow (= sulcus) separating ll into two ridges (inner ridge larger than outer one): lamina medialis (lm) well-developed; process (plm) lamellar, slightly short and thin, base long, tip dentate or crenate, indistinctly separated from distal lobe; distal lobe (dlm) quite long, tip directed ventroanteriad; broad lobe (blm) thick, indentation between broad lobe (blm) and distal lobe (dlm) inconspicuous. Solenomere (sl) quite long, distally twisted.

########### Distribution and habitat.

Known only from the type locality and nearby areas. This species seems to be rare because we made intensive surveys again in 2015 and 2016, no further specimens were found. As mentioned by Srisonchai et al. (2016), *D. des* is known only from two localities (Doi Angkhang and Wat Tham Krab), and we consider it to be endemic to Thailand.

########### Remarks.

This species exhibits some variation in the gonopods: the tip of process (plm) of lamina lateralis is dentate in some specimens, crenate in others. *Desmoxytes des* is easy to discriminate from other dragon millipedes by the distinct shape of paraterga and unique gonopod characters.

########### Coexisting species.

None known.

**Corrections to Srisonchai et al. (2016).** Srisonchai et al. (2016, pp. 99–103) wrote in the description of this species that paraterga (including paraterga of collum) are directed dorsolaterad at ca. 30°. They are in fact directed caudolaterad and are elevated at ca. 45° as stated in the updated redescription above. Moreover, Srisonchai et al. also described the surface of metaterga as finely shagreened, but we now regard it as being coarsely microgranulate.

########## Desmoxytes euros

Taxon classificationAnimaliaPolydesmidaParadoxosomatidae

Srisonchai, Enghoff & Panha sp. n.

http://zoobank.org/74AB93DF-E308-40C8-8E29-1F8AE6377651

**Figure 37.**
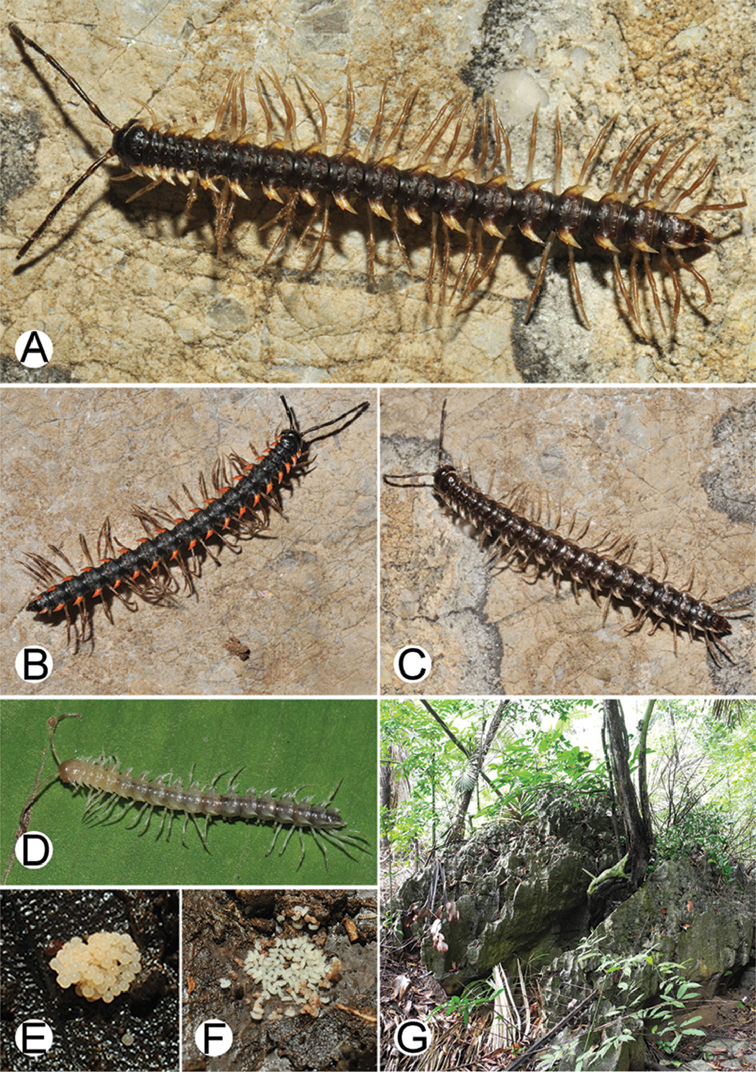
Photographs of live *Desmoxytes euros* sp. n. and habitat. **A, B** male paratypes **C** female paratype **D** juvenile **E** egg cluster **F** cluster of stadium 1 juveniles **G** habitat.

**Figure 38.**
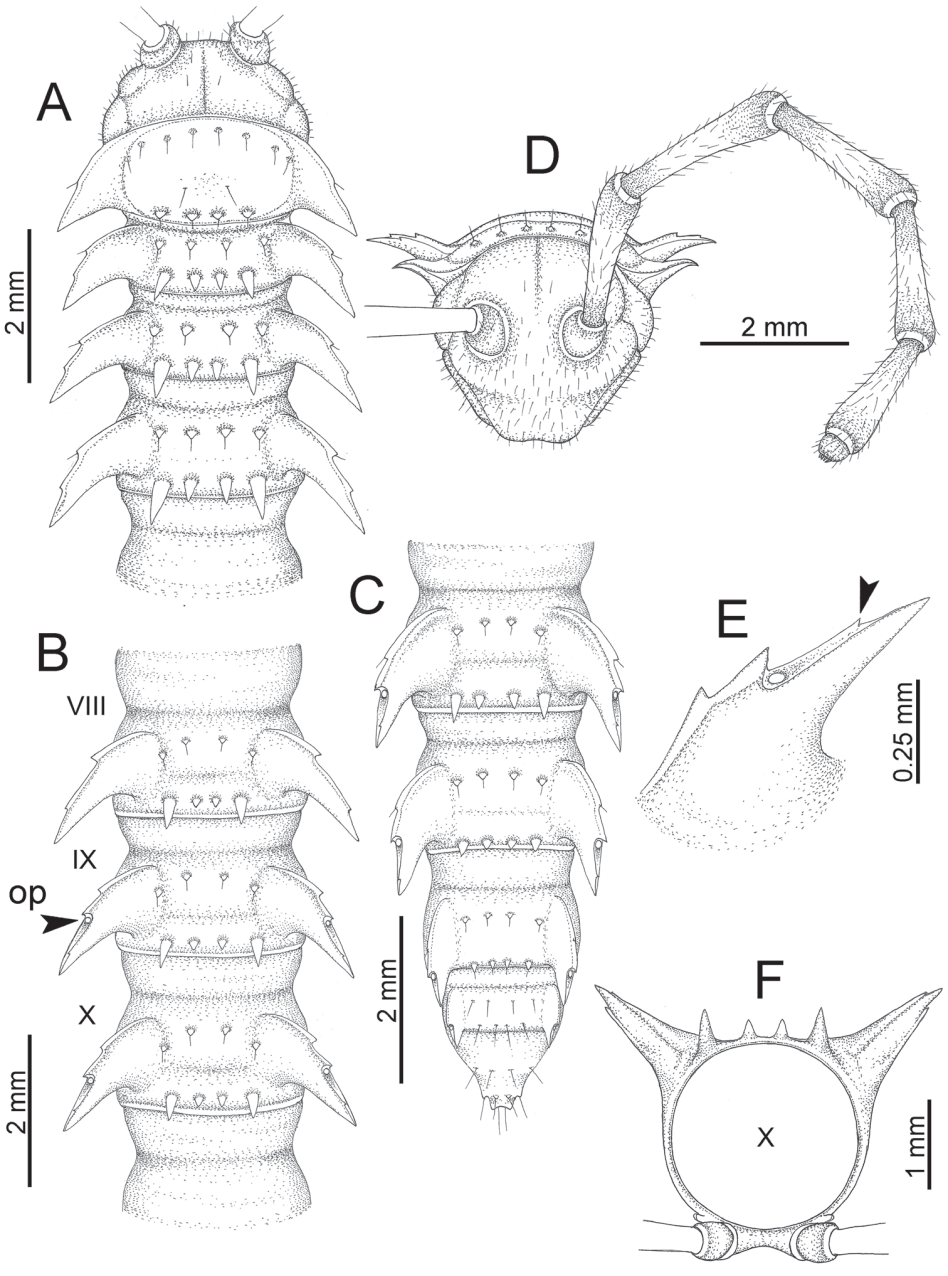
*Desmoxytes euros* sp. n. (male paratype). **A** anterior body part **B** body rings 8–10 (op = ozopore) **C** posteriormost body rings and telson **D** head and antenna **E** paraterga of ring 10 (arrow = tiny denticle) **F** body ring 10.

**Figure 39.**
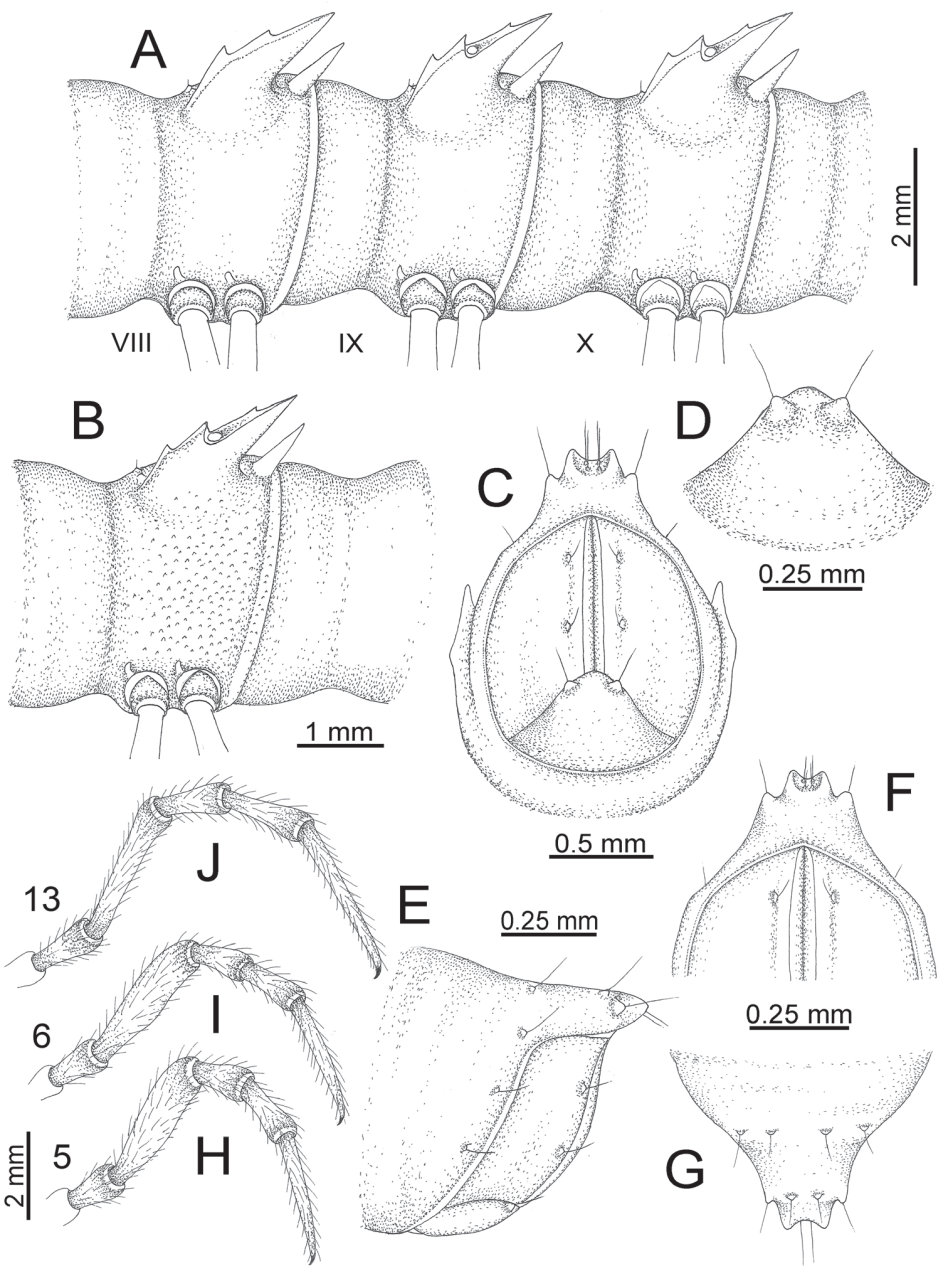
*Desmoxytes euros* sp. n. (male paratype). **A** body rings 8–10 **B** sculpture of ring 10 **C, E** last ring and telson **D** hypoproct **F, G** epiproct **H** male leg 5 (right) **I** male leg 6 (right) **J** male leg 13 (right).

**Figure 40.**
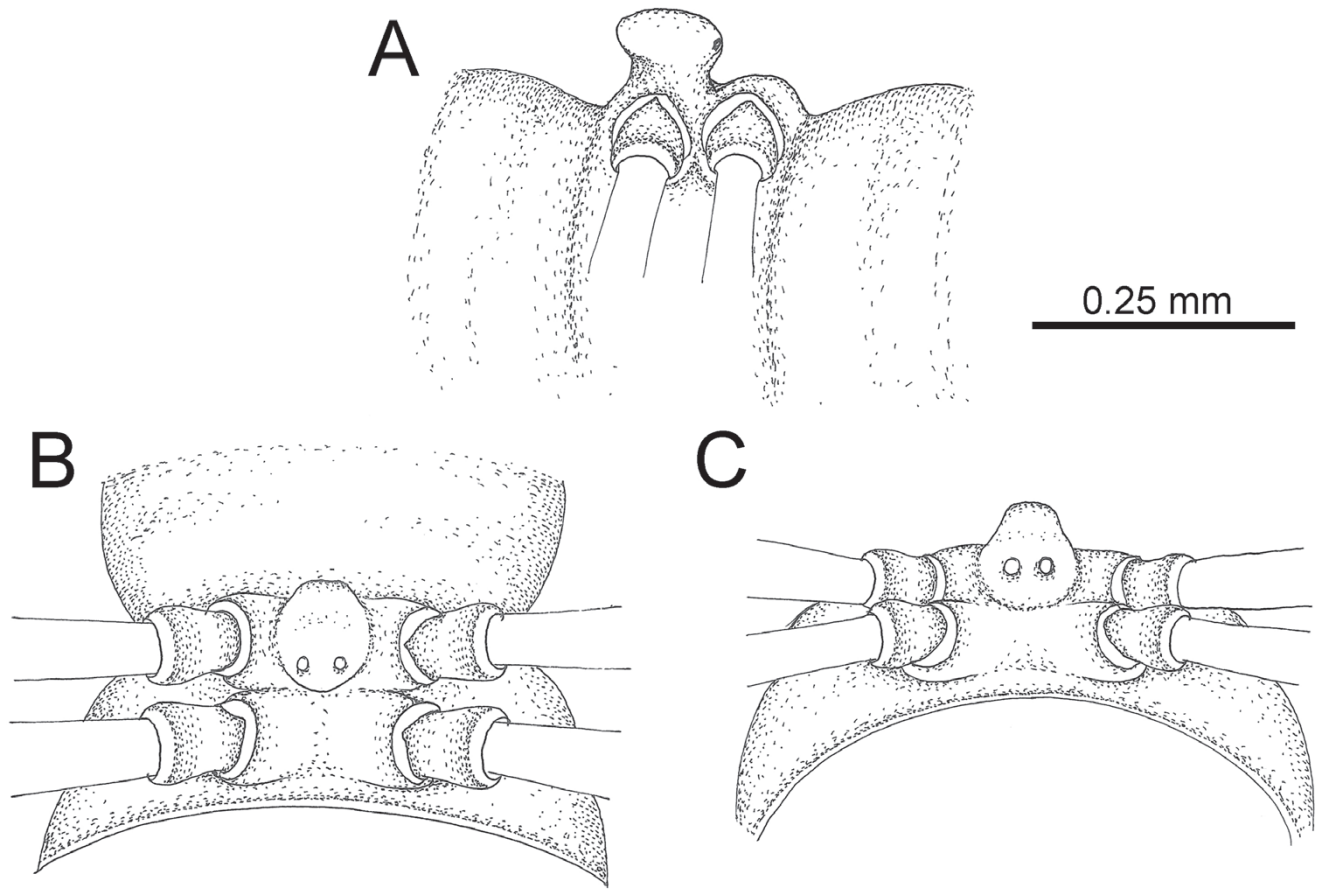
*Desmoxytes euros* sp. n. (male paratype) – sternal lobe between male coxae 4. **A** lateral view **B** ventral view **C** caudal view.

**Figure 41.**
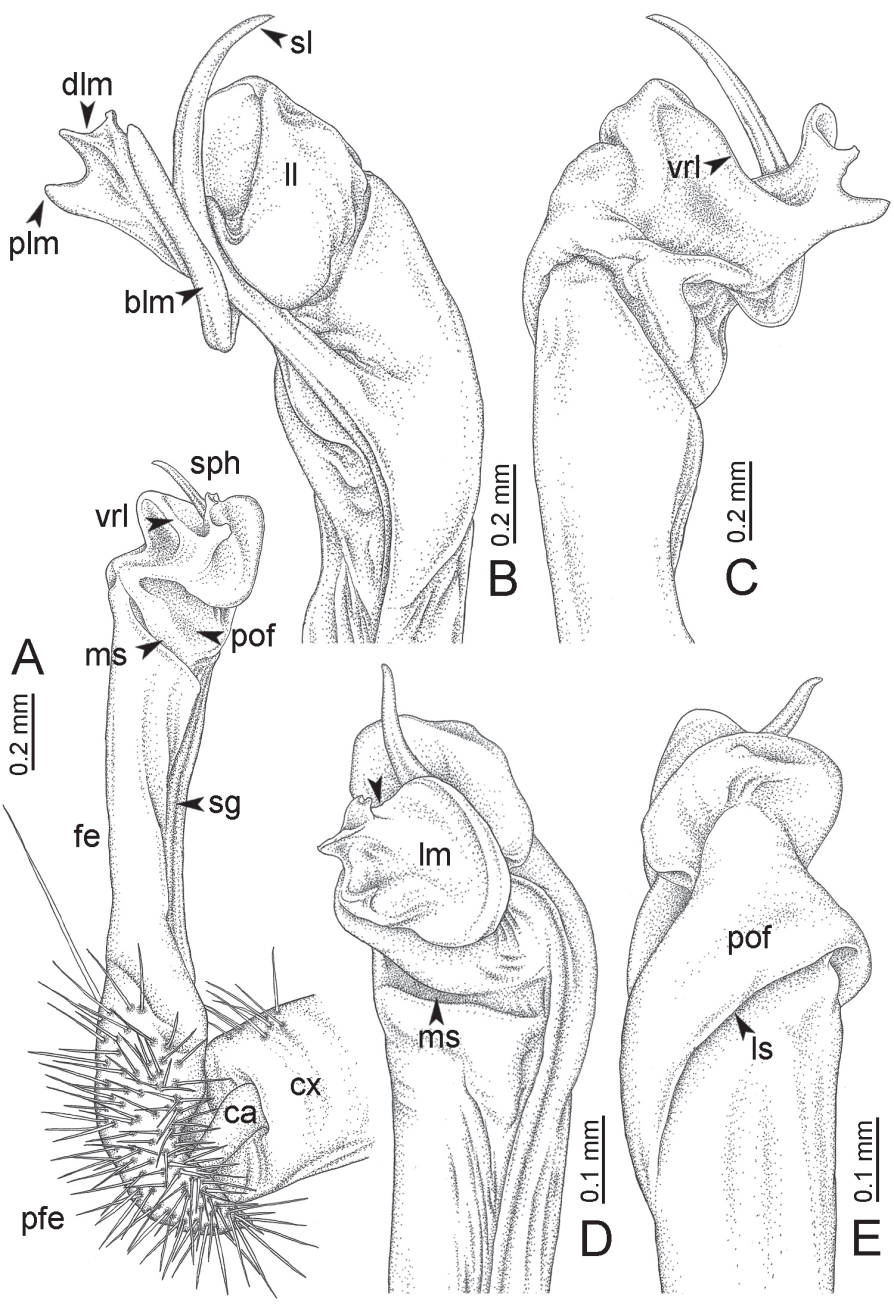
*Desmoxytes euros* sp. n. (paratype) – right gonopod. **A** mesal view **B** dorsal view **C** ventral view **D** submesal view (arrow = indentation) **E** lateral view.

**Figure 42.**
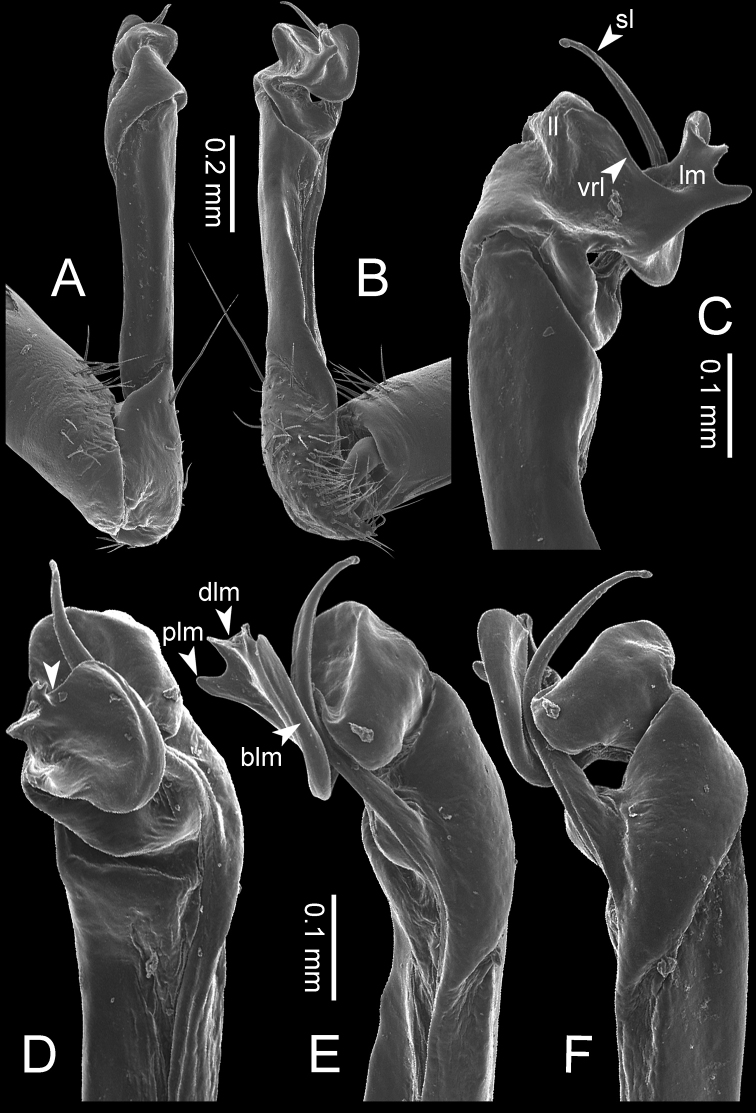
*Desmoxytes euros* sp. n. (paratype) – right gonopod. **A** lateral view **B** mesal view **C** ventral view **D** subdorsal view (arrow = indentation) **E** dorsal view **F** subdorsal view.

Figs 37 , 38 , 39 , 40 , 41 , 42

########### Holotype.

Male (CUMZ), THAILAND, Chanthaburi Province, Kaeng Hang Maeo District, Khao Wong Kot Cave, 12°53'53"N, 101°49'00"E, ca. 53 m a.s.l., 4 August 2016, leg. S. Panha, P. Tongkerd ans ASRU members.

########### Paratypes.

13 males, 5 females (CUMZ), same data as holotype. 3 males, 4 females (CUMZ), 2 males, 1 female (ZMUC), 1 male (NHMW), THALAND, Rayong Province, Khao Chamao District, Wat Tham Suwan Phupha, 12°59'16"N, 101°39'32"E, ca. 64 m a.s.l., 8 August 2017, leg. P. Tongkerd and ASRU members.

########### Further specimens, 

**not paratypes, all from THAILAND, Chantaburi Province**: 1 male, 1 female (CUMZ), Kaeng Hang Maeo District, Khao Wong Kot Cave, 12°53'53"N, 101°49'00"E, ca. 53 m a.s.l., 15 September 2009, leg. S. Panha and ASRU members. 1 male, 1 female (CUMZ), Kaeng Hang Maeo District, Khao Wong Kot Cave, 12°53'53"N, 101°49'00"E, ca. 53 m a.s.l., 1 October 2009, leg. S. Panha and ASRU members. 2 males, 1 female (CUMZ), Kaeng Hang Maeo District, near Khao Wong Kot Cave, 12°53'53"N, 101°49'00"E, ca. 53 m a.s.l., 15 October 2010, leg. S. Panha and ASRU members. 1 male, 2 females (CUMZ), Kaeng Hang Maeo District, near Khao Wong Kot Cave, 12°53'53"N, 101°49'00"E, ca. 53 m a.s.l., 24 October 2010, leg. S. Panha and ASRU members. 4 males, 4 females, 2 juveniles (CUMZ), Kaeng Hang Maeo District, Khao Wong Kot Cave, 12°53'53"N, 101°49'00"E, ca. 53 m a.s.l., 17 October 2015, leg. C. Sutcharit and R. Srisonchai. 1 female (CUMZ), Kaeng Hang Maeo District, Khao Wong Kot Cave, 12°53'53"N, 101°49'00"E, ca. 53 m a.s.l., 8 August 2017, leg. S. Panha, P. Tongkerd ans ASRU members. 5 males, 3 females (CUMZ), Kaeng Hang Maeo District, Khao Sip Ha Chan National Park, 13°10'33"N, 102°00'09"E, ca. 118 m a.s.l., 7 October 2010, leg. S. Panha and ASRU members. 2 males, 1 female (CUMZ), Khlung District, Makok Waterfall, 12°35'14"N, 102°15'21"E, ca. 58 m a.s.l., 3 September 2007, leg. ASRU members. 1 male, 1 female (CUMZ), Khlung District, Makok Waterfall, 12°35'14"N, 102°15'21"E, ca. 59 m a.s.l., 3 September 2007, leg. ASRU members. 3 males, 3 females, 4 females (CUMZ), Khlung District, Makok Waterfall, 12°35'14"N, 102°15'21"E, ca. 58 m a.s.l., 10 August 2014, leg. ASRU members. 5 males, 3 females (CUMZ), Mueang Chantaburi District, Phlio Waterfall, 12°31'44"N 102°10'57"E, ca. 104 m a.s.l., 19 October 2015, leg. S. Panha and ASRU members. 2 males, 2 females (CUMZ), Tha Mai District, Wat Khao Su Kim (Khao Su Kim Temple), 12°45'47"N, 102°01'56"E, ca. 148 m a.s.l., 29 September 2009, leg. S. Panha and ASRU members. 3 males, 5 females (CUMZ), Tha Mai District, Wat Khao Su Kim (Khao Su Kim Temple), 12°45'47"N, 102°01'56"E, ca. 148 m a.s.l., 14 October 2010, leg. S. Panha and ASRU members. 2 males, 2 females (CUMZ), Tha Mai District, Wat Khao Su Kim (Khao Su Kim Temple), 12°45'47"N, 102°01'56"E, ca. 148 m a.s.l., 6 August 2011, leg. S. Panha and ASRU members. 2 males, 1 female, 1 juvenile (CUMZ), Tha Mai District, Wat Khao Su Kim (Khao Su Kim Temple), 12°45'47"N, 102°01'56"E, ca. 148 m a.s.l., 8 August 2017, leg. S. Panha, P. Tongkerd and ASRU members.

**Chonburi Province**: 2 males, 3 females (CUMZ), Bo Thong District, Wat Tham Khao Cha-ang Oune, 13°12'35"N, 101°39'09"E, ca. 151 m a.s.l., 15 September 2009, leg. ASRU members. 2 males, 3 females (CUMZ), Bo Thong District, Wat Tham Khao Cha-ang Oune, 13°12'35"N, 101°39'09"E, ca. 151 m a.s.l., 15 October 2010, leg. ASRU members. 2 males (CUMZ), Bo Thong District, Wat Tham Khao Cha-ang Oune, 13°12'35"N, 101°39'09"E, ca. 151 m a.s.l., 24 September 2012, leg. S. Panha and ASRU members. 2 males, 2 females (CUMZ), Bo Thong District, Wat Tham Khao Cha-ang Oune, 13°12'35"N, 101°39'09"E, ca. 151 m a.s.l., 6 July 2016, leg. A. Pholyotha and ASRU members. 1 male, 1 female (CUMZ), Bo Thong District, Wat Tham Khao Cha-ang Oune, 13°12'35"N, 101°39'09"E, ca. 151 m a.s.l., 25 October, leg. ASRU members.

**Rayong Province**: 1 male, 1 female (CUMZ), Khao Chamao District, Wat Tham Watana Monkhol, 13°05'45"N, 101°36'28"E, ca. 76 m a.s.l., Unknown date, leg. S. Panha and ASRU members. 3 males (CUMZ), Khao Chamao District, Wat Tham Khao Loy (Wat Ma Duae), 13°03'26"N, 101°36'28"E, ca. 71 m a.s.l., 5 September 2008, leg. S. Panha and ASRU members. 1 male, 1 female (CUMZ), Khao Chamao District, Wat Tham Khao Loy (Wat Ma Duae), 13°03'26"N, 101°36'28"E, ca. 71 m a.s.l., 23 October 2010, leg. S. Panha and ASRU members. 1 male, 3 females (CUMZ), Khao Chamao District, Wat Tham Khao Loy (Wat Ma Duae), 13°03'26"N, 101°36'28"E, ca. 71 m a.s.l., 9 November 2013, leg. S. Panha and ASRU members. 1 male, 1 female (CUMZ), Khao Chamao District, Tham Khao Pratun Monastery, 13°07'26"N, 101°35'52"E, ca. 107 m a.s.l., 24 October 2010, leg. S. Panha and ASRU members. 1 male, 1 female (CUMZ), Khao Chamao District, Khlong Pra Kang Waterfall, 12°55'59"N, 101°42'58"E, ca. 69 m a.s.l., 15 September 2009, leg. S. Panha and ASRU members. 1 male, 1 female (CUMZ), Khao Chamao District, Tham Khao Boath Bureau of Monks, 13°02'13"N, 101°38'08"E, ca. 102 m a.s.l., 23 October 2010, leg. S. Panha and ASRU members. 2 males, 2 females (CUMZ), Khao Chamao District, Wat Nong Tha Khian, 12°57'49"N, 101°40'24"E, ca. 64 m a.s.l., 5 September 2008, leg. C. Sutcharit and ASRU members. 1 male, 1 female (CUMZ), Khao Chamao District, Wat Tham Khao Chamao, 23 October 2008, leg. C. Sutcharit and ASRU members.

**Sa Kaeo Province**: 1 male, 1 female (CUMZ), Khlong Hat District, Phet Pho Thong Cave, 13°24'50"N, 102°19'33"E, ca. 240 m a.s.l., 28 October 2010, leg. C. Sutcharit and ASRU members. 1 male, 1 female (CUMZ), Khao Chakan District, near Wat Tham Khao Chakan, 13°39'40"N, 102°05'11"E, ca. 75 m a.s.l., 28 August 2014, leg. S. Panha and ASRU members. 2 males, 1 female (CUMZ), Khlong Hat District, Phet Pho Thong Cave, 13°24'50"N, 102°19'33"E, ca. 240 m a.s.l., 28 August 2014, leg. S. Panha and ASRU members. 1 male, 3 females (CUMZ), Khao Chakan District, near Wat Tham Khao Chakan, 13°39'40"N, 102°05'11"E, ca. 75 m a.s.l., 3 September 2015, leg. S. Panha and ASRU members.

########### Diagnosis.

Body black or brownish black; collum with 3 transverse rows of setae and setiferous tubercles (4+4 anterior setae, 1+1 intermediate setae and 2+2 posterior tubercles); metaterga 2–16 with two rows of 2+2 (anterior) setiferous cones and 2+2 (posterior) setiferous spines; ventral ridge (vrl) of lamina lateralis conspicuous; process (plm) of lamina medialis long, directed almost mesad; distal lobe (dlm) distally with two distinct lamellae. Similar in these respects to *D. planata*, but differs from that species by having paraterga yellow to orange and hypoproct subtriangular with conspicuous setiferous tubercles.

########### Etymology.

“*Euros*” (noun in apposition) is the name of the ancient Greek god of the east wind; the name refers to the occurrence of this species in the eastern part of Thailand.

########### Description.

SIZE: Length 25–27 mm (male), 28–29 mm (female); width of midbody metazona ca. 1.6 mm (male), 2 mm (female). Width of head < collum = body ring 2 < 3 = 4 < 5–16, thereafter body gradually tapering towards telson.

COLOUR (Fig. 37A–D): In life with body dark or brownish black; paraterga yellow to orange; head, antenna, metaterga and surface below paraterga dark brown (except distal part of antennomere 7 and antennomere 8 whitish); epiproct, sterna and a few basal podomeres brown to whitish; legs otherwise brown.

ANTENNAE (Fig. 38D): Moderately long and slender, reaching to body ring 6 (male) and 5 (female) when stretched dorsally.

COLLUM (Fig. 38A): With 3 transverse rows of setae and setiferous tubercles, 4+4 anterior setae, 1+1 intermediate setae and 2+2 posterior tubercles (lateral seta in anterior row located almost at base of paraterga in some specimens); paraterga of collum low, elevated at ca. 15°–20°, directed caudolaterad, with one inconspicuous notch on lateral margin.

TEGUMENT: Quite dull, slightly shining; collum and metaterga microgranulate; prozona finely shagreened; surface below paraterga finely microgranulate; sterna and epiproct smooth.

METATERGA (Fig. 38A–C): With 2 transverse rows of setiferous tubercles, cones and spines; metaterga 2–8 with 2+2 anterior cones and 2+2 posterior spines; metaterga 9–16 with 2+2 anterior cones and 2+2 posterior spines; metaterga 17–18 with 2+2 anterior and 2+2 posterior cones; metatergum 19 with 2+2 anterior and 2+2 posterior tubercles.

PARATERGA (Fig. 38E, F): Directed caudolaterad on body rings 2–17, elevated at ca. 45° (male) 40° (female); directed increasingly caudad on body rings 18 and 19; anterior margin with 2 distinct notches, on lateral margin of body rings 9, 10, 12, 13, 15–18 with tiny denticle near the tip.

TELSON (Fig. 39C–G): Epiproct: tip emarginate; lateral setiferous tubercles and apical tubercles conspicuous. Hypoproct subtriangular; caudal margin subtriangular, with conspicuous setiferous tubercles.

STERNA (Fig. 40): Cross-impressions shallow. Sternal lobe between male coxae 4 swollen, base stout, slightly attenuated near tip, knob-like when seen in lateral view, tip subtruncate.

LEGS (Fig. 39H–J): Long and slender. Male femora 5 and 6 moderately humped ventrally in middle portion.

GONOPODS (Figs 41, 42): Coxa (cx) longer than prefemur. Cannula (ca) long and slender. Prefemur (pfe) ca. 2/3 as long as femur. Femur (fe) long and slender. Mesal sulcus (ms) and lateral sulcus (ls) conspicuous, very deep. Postfemur (pof) conspicuous, ventrally narrow and short. Solenophore (sph) well-developed: lamina lateralis (ll) well-developed, inner surface subsided, anterolaterally with an inconspicuous furrow; with conspicuous ventral ridge (vrl): lamina medialis (lm) well-developed; process (plm) long, tip blunt, directed mesad; distal lobe (dlm) distally with two distinct lamellae (equal in size); broad lobe (blm) dorsally thick at the edge, with a wide and conspicuous indentation between broad lobe (blm) and distal lobe (dlm). Solenomere (sl) very long.

########### Distribution and habitat.

Known only from east Thailand (Chantaburi, Chonburi, Sa Kaeo and Rayong Provinces). Interestingly, this species exists in both limestone areas and granitic mountains. It was seen crawling on rocks during the rainy season and occurs only in natural habitat inside primary forest. On the basis of current data, the distribution area is quite narrow, and the species seems to be restricted to the eastern part of Thailand. We thus regard *D. euros* sp. n. to be an endemic for the Thai fauna.

########### Remarks.

The observation of all living specimens reveals variation on colour of paraterga within a population; yellow in some specimens, yellowish orange to orange in others.

*Desmoxytes euros* sp. n. strongly resembles *D. planata* in several morphological characters (except for the characters mentioned in the diagnosis); notably the gonopod characters are identical. However, our initial study on DNA barcoding gene (COI) revealed that *D. planata* and the new species are separated enough to support the suggestion that *D. planata* and the new species are indeed different species (paper in preparation).

Because of the similarity in gonopod morphology, it is difficult to discriminate old material of *D. planata* and *D. euros* sp. n. However, they can be distinguished by colour of paraterga (yellow or yellow orange in *D. euros* sp. n., pink in *D. planata*), and by characters of hypoproct (subtriangular in *D. euros* sp. n., subtrapeziform in *D. planata*).

We kept several adults in an acrylic box with litter at room temperature. Two weeks later, we found a nest with eggs and a cluster of stadium 1 juveniles at ca. 2 cm depth in the soil and leaf litter (Fig. 37E, F).

########### Coexisting species.

None known.

########## Desmoxytes flabella

Taxon classificationAnimaliaPolydesmidaParadoxosomatidae

Srisonchai, Enghoff & Panha sp. n.

http://zoobank.org/FA63C81E-F8B9-450F-A771-7CE8EFA098D4

**Figure 43.**
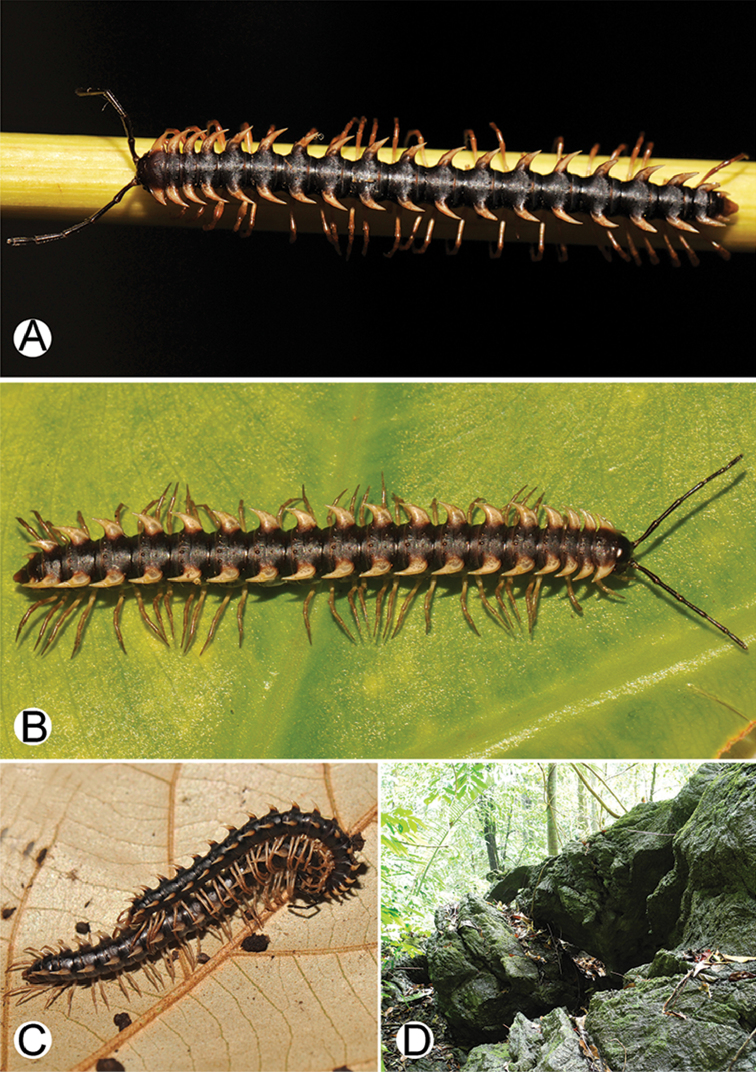
Photographs of live *Desmoxytes flabella* sp. n. and habitat. **A** male paratype **B** female paratype **C** mating couple **D** habitat.

**Figure 44.**
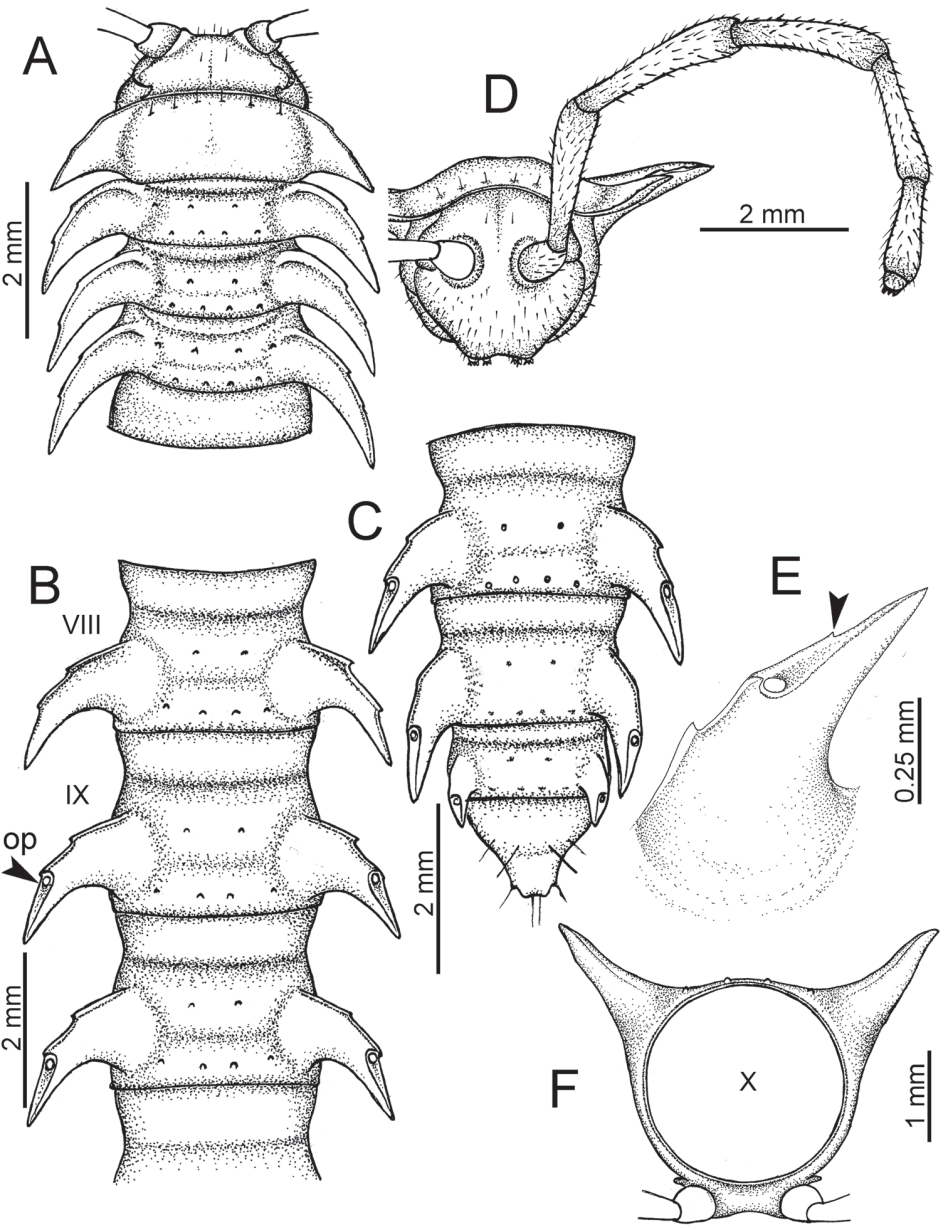
*Desmoxytes flabella* sp. n. (male paratype). **A** anterior body part **B** body rings 8–10 (op = ozopore) **C** posteriormost body rings and telson **D** head and antenna **E** paraterga of ring 10 (arrow = tiny denticle) **F** body ring 10.

**Figure 45.**
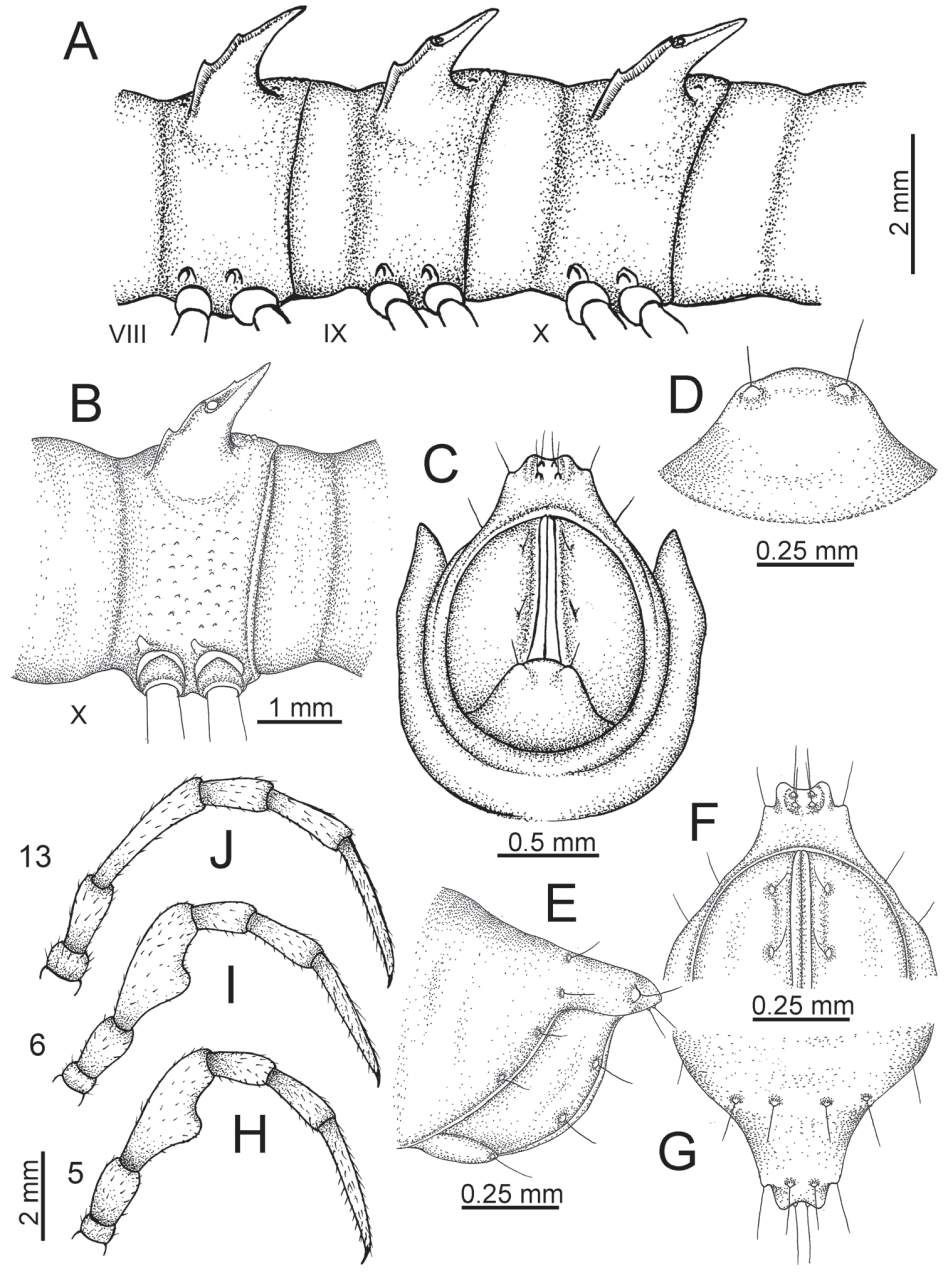
*Desmoxytes flabella* sp. n. (male paratype) **A** body rings 8–10 **B** sculpture of ring 10 **C, E** last ring and telson **D** hypoproct **F, G** epiproct **H** male leg 5 (right) **I** male leg 6 (right) **J** male leg 13 (right).

**Figure 46.**
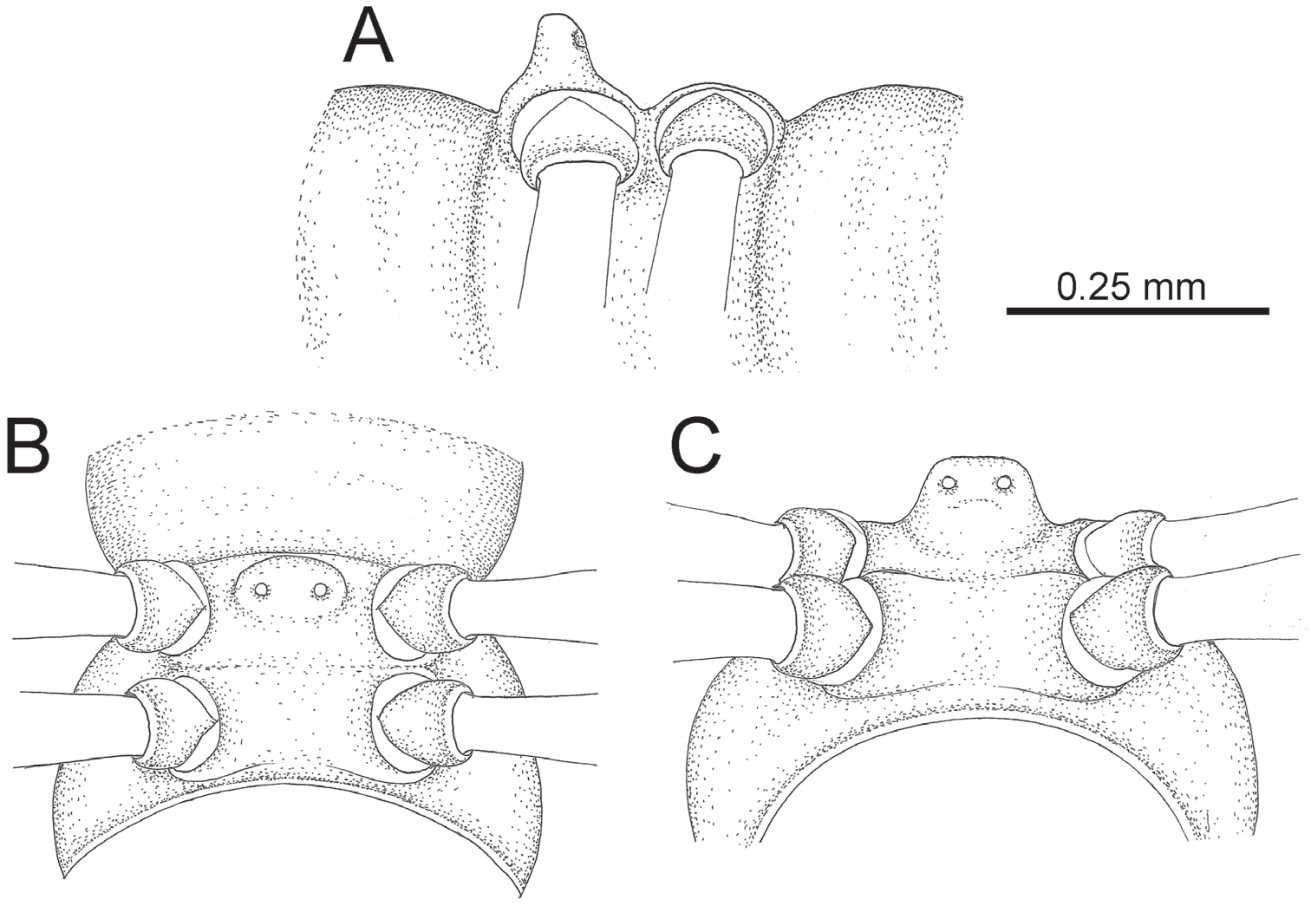
*Desmoxytes flabella* sp. n. (male paratype) – sternal lobe between male coxae 4. **A** lateral view **B** ventral view **C** caudal view.

**Figure 47.**
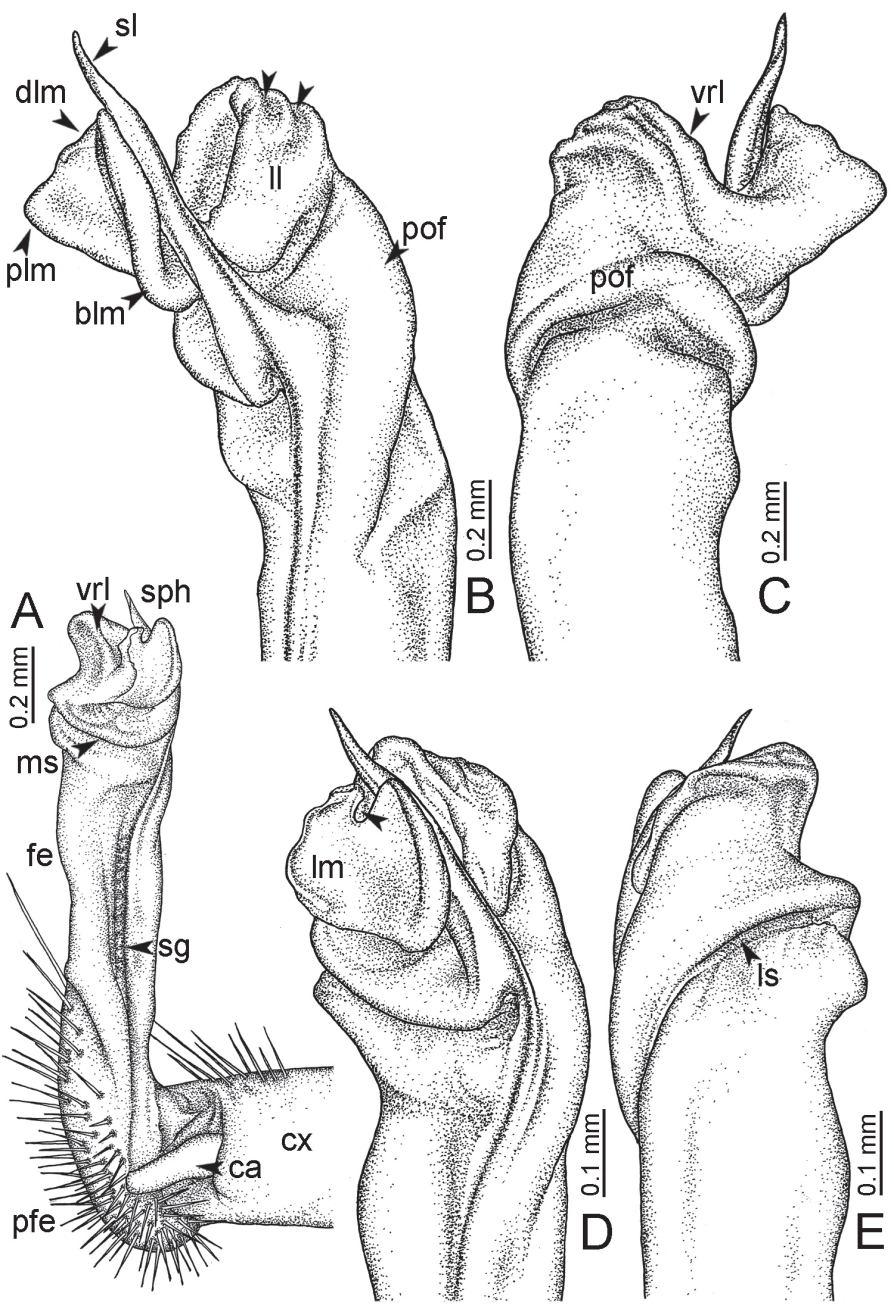
*Desmoxytes flabella* sp. n. (paratype) – right gonopod. **A** mesal view **B** dorsal view (arrows = furrows) **C** ventral view **D** submesal view (arrow = indentation) **E** lateral view.

**Figure 48.**
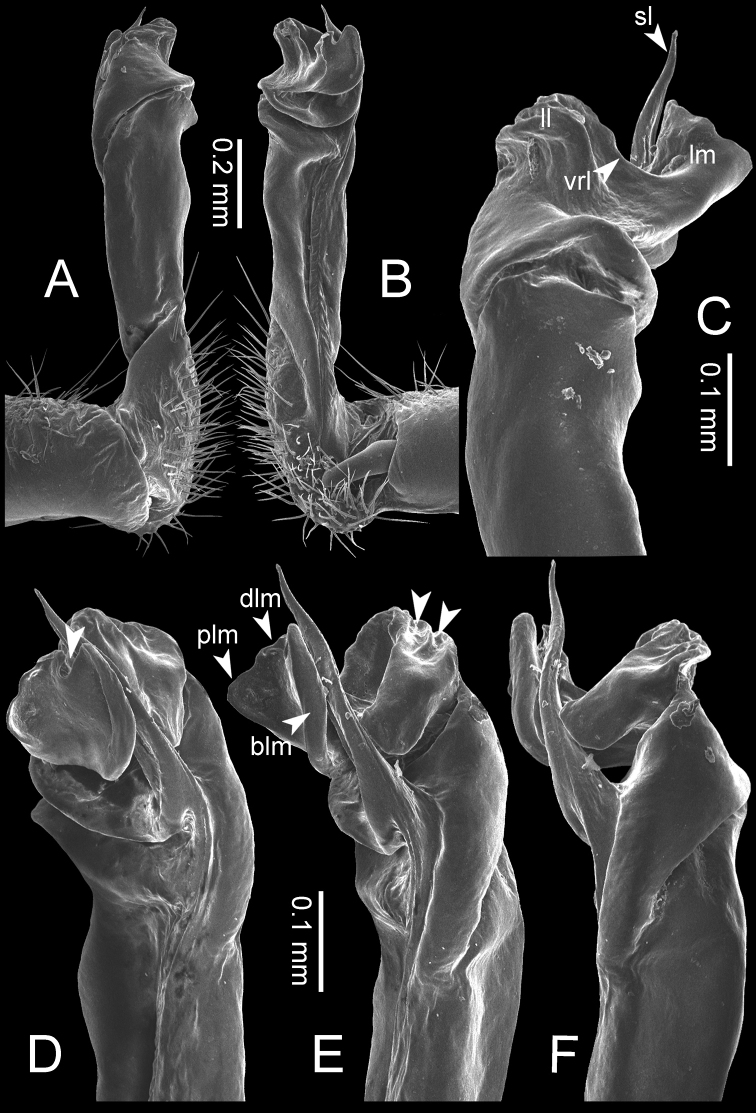
*Desmoxytes flabella* sp. n. (paratype) – right gonopod. **A** lateral view **B** mesal view **C** ventral view **D** subdorsal view (arrows = indentations) **E** dorsal view (arrows = furrows) **F** subdorsal view.

Figs 43 , 44 , 45 , 46 , 47 , 48

########### Holotype.

Male (CUMZ), THAILAND, Trang Province, Palian District, Tham Khao Ting, 7°09'31"N, 99°48'10"E, ca. 42 m a.s.l., 8 July 2017, leg. C. Sutcharit, R. Srisonchai and ASRU members.

########### Paratypes.

13 males, 4 females (CUMZ), 1 male, 1 female (ZMUC), 1 male (ZMUM), 1 male (NHMW), 1 male (NHMUK), same data as holotype. 3 males, 3 females, 2 juveniles (CUMZ), THAILAND, Trang Province, Palian District, Tham Khao Ting, 7°09'31"N, 99°48'10"E, ca. 42 m a.s.l., 31 August 2015, leg. C. Sutcharit, R. Srisonchai and ASRU members.

########### Further specimens, not paratypes.

7 males, 3 females (CUMZ), THAILAND, Satun Province, Thung Wa District, Tham Khan Ti Phol, 7°05'08"N, 99°47'54"E, ca. 80 m a.s.l., 8 July 2017, leg. C. Sutcharit, R. Srisonchai and ASRU members.

########### Diagnosis.

Collum with one row of 3+3 setae (anterior row); paraterga wing-like (not knife-shaped); metaterga 2–19 with 2+2 tubercles in anterior row and 2+2 tubercles in posterior row; male femora 5 and 6 modified; lamina lateralis (ll) distally rough, anterolaterally with 2–3 distinct furrows; process (plm) of lamina medialis very short. Similar in these respects to *D. perakensis* sp. n. Differs from that species by having body black or brownish black contrasting with yellowish brown paraterga; paraterga narrower; process (plm) of lamina medialis indistinctly demarcated from distal lobe (dlm); distal lobe (dlm) of lamina medialis with one lamella.

########### Etymology.

The name is a Latin noun, referring to the shape of process (plm) and the distal lobe (dlm) on lamina medialis which somewhat resemble a handheld fan or flyswatter.

########### Description.

SIZE: Length 32–35 mm (male), 34–36 mm (female); width of midbody metazona ca. 2.2 mm (male), 2.5 mm (female). Width of head < collum = body ring 2 = 3 = 4 < 5–16, thereafter body gradually tapering toward telson.

COLOUR (Fig. 43A–C): In life with body brownish black or black; metaterga and antenna black (except distal part of antennomere 7 and antennomere 8 whitish); surface below paraterga, head and epiproct brownish black; paraterga yellowish brown; sterna and legs brown; a few basal podomeres brownish white.

ANTENNAE (Fig. 44D): Moderately long and slender, reaching to body ring 6 (male) and 4–5 (female).

COLLUM (Fig. 44A): With 1 transverse anterior row of 3+3 setae; paraterga of collum low, elevated at ca. 10°–15°, directed caudolaterad, with 2 distinct notches on lateral margin.

TEGUMENT: Moderately shining; collum and metaterga coarsely microgranulate; prozona finely shagreened; surface below paraterga coarsely microgranulate; sterna and epiproct smooth.

METATERGA (Fig. 44A–C): With 2 transverse rows of conspicuous tubercles; metaterga 2–19 with 2+2 anterior and 2+2 posterior tubercles.

PARATERGA (Fig. 44E, F): Directed caudolaterad on body rings 2–17, elevated at ca. 45° (male) 45° (female); directed increasingly caudad on body rings 18 and 19; anterior margin with 2 distinct notches, on lateral margin of body rings 9, 10, 12, 13, 15–18 with tiny denticle near the tip.

TELSON (Fig. 45C–G): Epiproct: tip emarginate; lateral setiferous tubercles inconspicuous; apical tubercles conspicuous, small, knob-like. Hypoproct subtrapeziform, broad; caudal margin round, with two conspicuous setiferous tubercles.

STERNA (Fig. 46): Cross-impressions shallow. Sternal lobe between male coxae 4 swollen, suberect, subtrapeziform when seen in posterior view, tip usually truncate (in some specimens slightly round).

LEGS (Fig. 45H–J): Long and slender. Male femora 5 and 6 strongly humped ventrally in middle portion.

GONOPODS (Figs 47, 48): Coxa (cx) subequal in length to prefemur. Cannula (ca) quite long and slender. Telopodite quite stout. Prefemur (pfe) ca. 2/3 as long as femur. Femur (fe) somewhat stout, slightly enlarged distally. Mesal sulcus (ms) and lateral sulcus (ls) conspicuous, very deep. Postfemur (pof) conspicuous, ventrally narrow and short. Solenophore (sph) well-developed: lamina lateralis (ll) rough, anterolaterally with 2–3 distinct furrows, deep; ventral ridges (vrl) conspicuous: lamina medialis (lm) well-developed; process (plm) very short, indistinctly demarcated from distal lobe; distal lobe (dlm) distally with one lamella; broad lobe (blm) thick, clearly demarcated from distal lobe (dlm) by a narrow and deep indentation. Solenomere (sl) long, apically twisted.

########### Distribution and habitat.

Known only from the type locality and a few nearby localities. The new species is restricted to limestone habitats (Fig. 43D), and it is sympatric with *D. delfae* at Tham Khao Ting and Tham Khan Ti Phol. Unlike the bright orange *D. delfae*, which was easily spotted crawling on branches of shrubs and on rocks, the new species, blackish brown, was found on rocks where it was quite hard to see. This suggests that these two species, although sharing the same habitat, may show microhabitat differences, but this has not yet been studied in detail. We assume that the new species is distributed along limestone mountain ranges in a narrow area at the border between Trang and Satun Provinces. This species should be regarded as endemic to the Thai fauna.

########### Coexisting species.

*Desmoxytes delfae* co-occurs at the same localities.

########## Desmoxytes golovatchi

Taxon classificationAnimaliaPolydesmidaParadoxosomatidae

Srisonchai, Enghoff & Panha sp. n.

http://zoobank.org/D91567EA-E62F-4183-AF96-A4EBF12B895E

**Figure 49.**
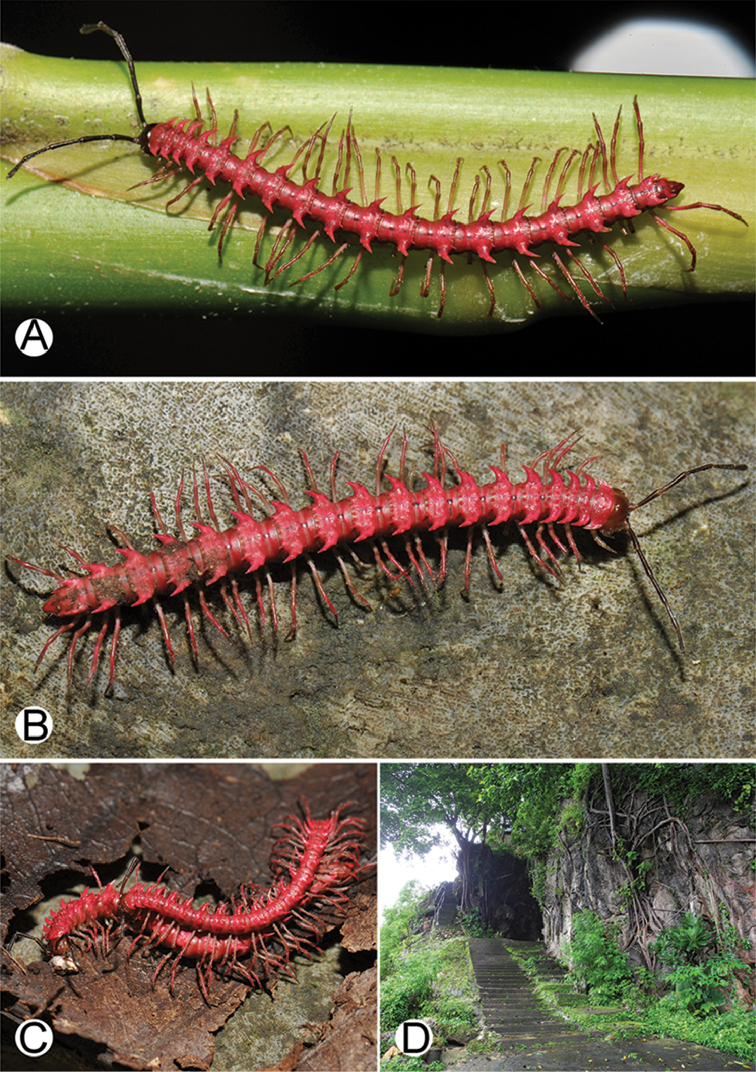
Photographs of live *Desmoxytes golovatchi* sp. n. and habitat. **A** male paratype **B** female paratype **C** mating couple **D** habitat.

**Figure 50.**
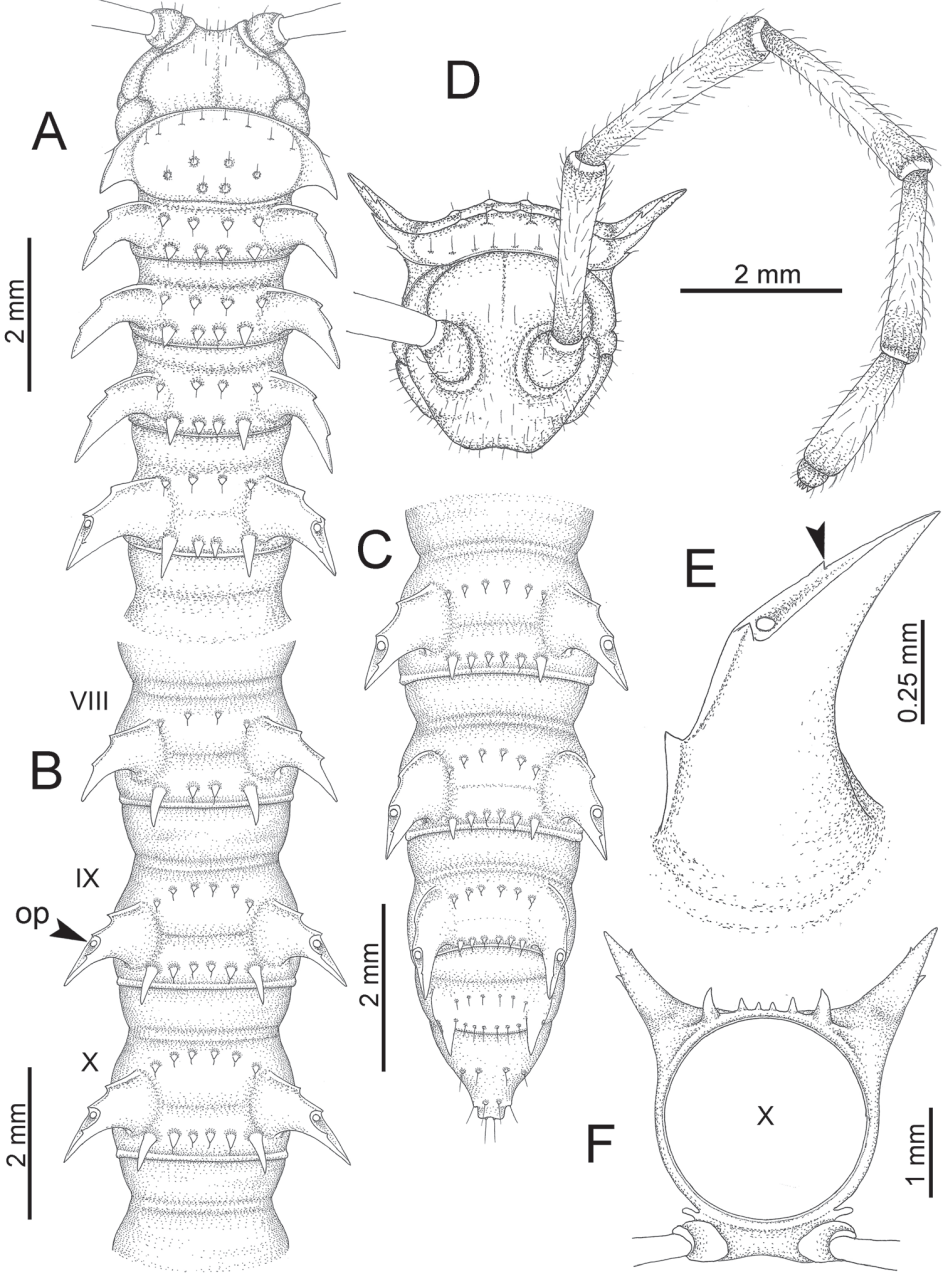
*Desmoxytes golovatchi* sp. n. (male paratype). **A** anterior body part **B** body rings 8–10 (op = ozopore) **C** posteriormost body rings and telson **D** head and antenna **E** paraterga of ring 10 (arrow = tiny denticle) **F** body ring 10.

**Figure 51.**
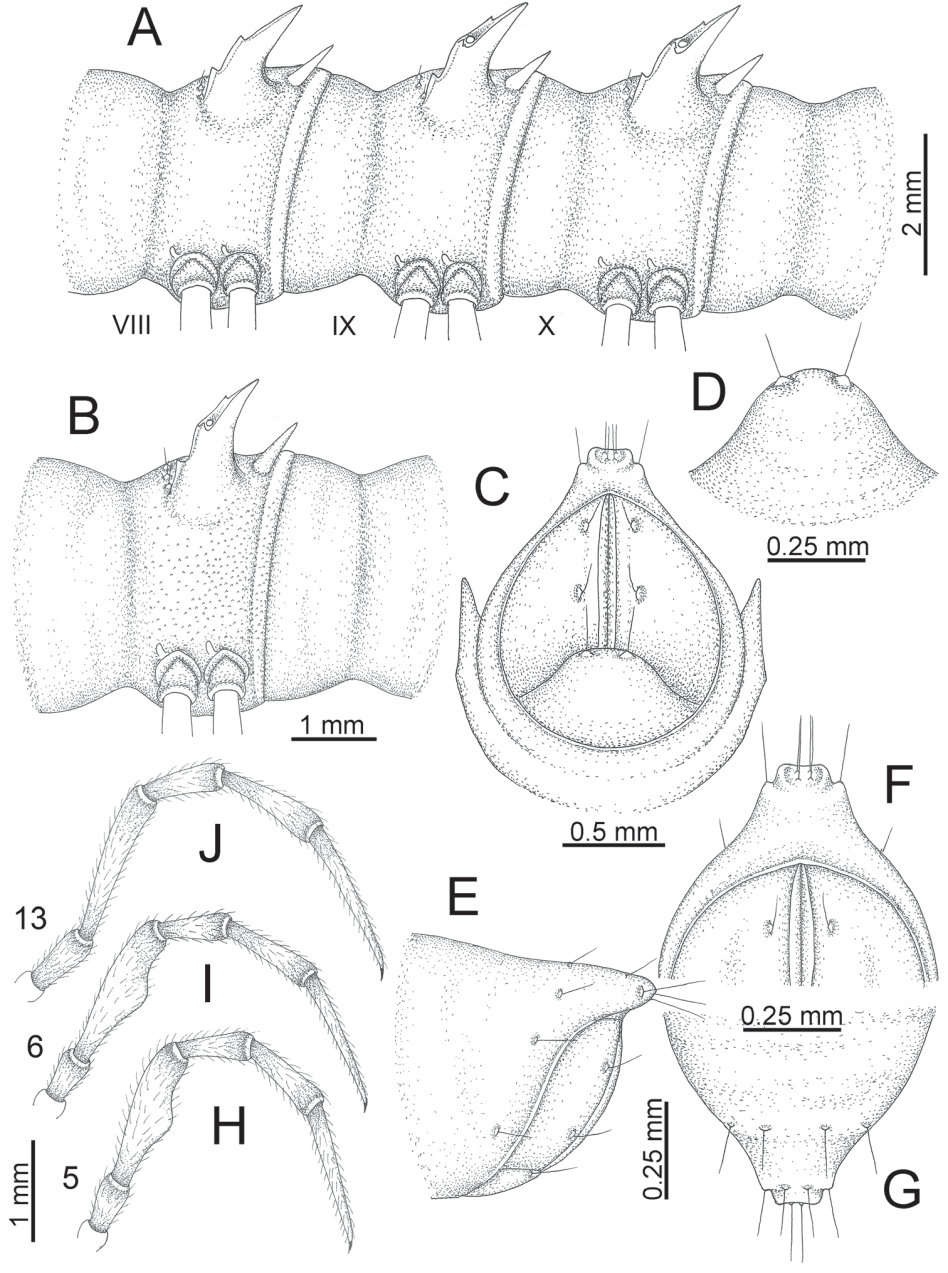
*Desmoxytes golovatchi* sp. n. (male paratype). **A** body rings 8–10 **B** sculpture of ring 10 **C, E** last ring and telson **D** hypoproct **F, G** epiproct **H** male leg 5 (right) **I** male leg 6 (right) **J** male leg 13 (right).

**Figure 52.**
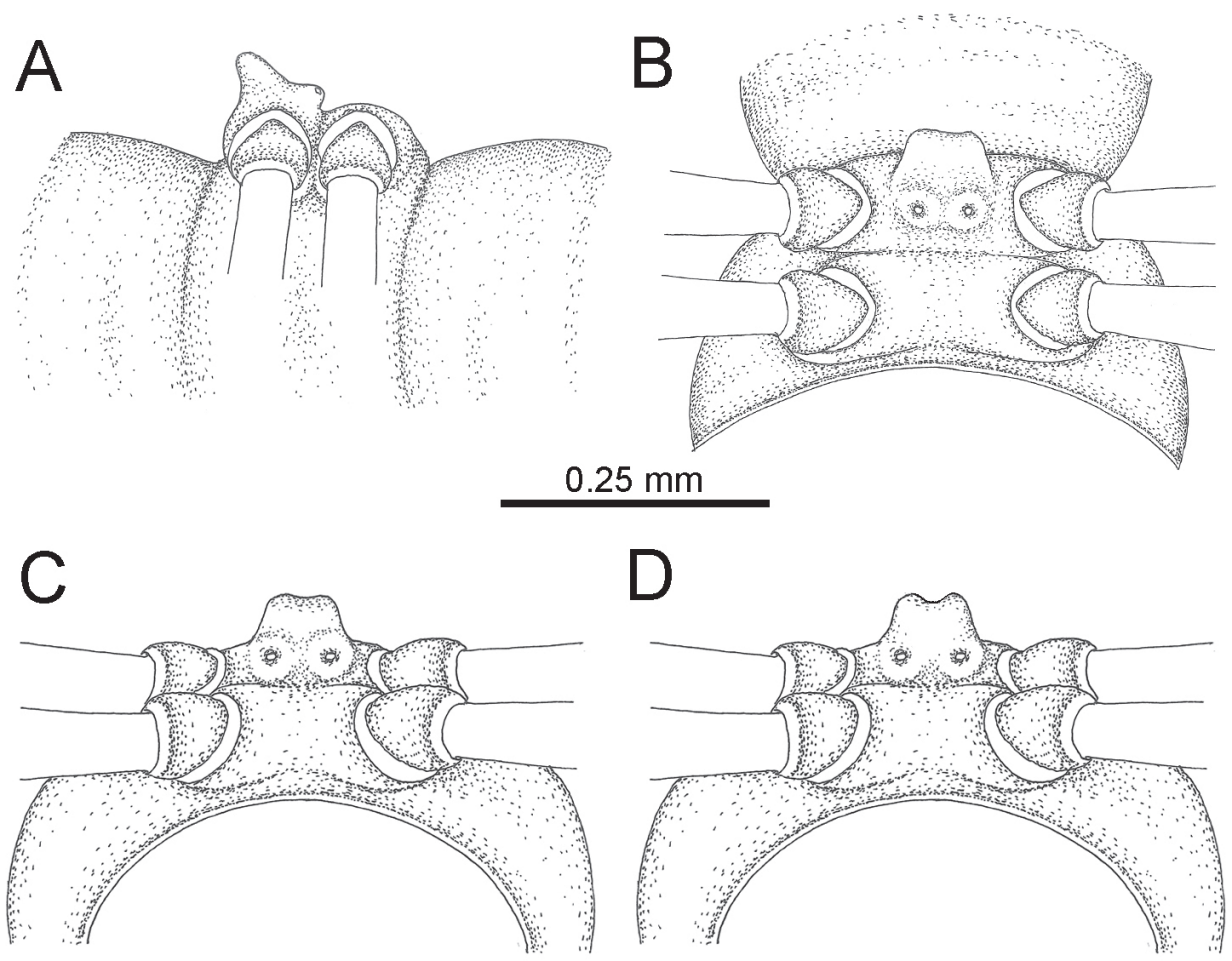
*Desmoxytes golovatchi* sp. n. (male paratype) – sternal lobe between male coxae 4. **A** lateral view **B** ventral view **C, D** caudal view.

**Figure 53.**
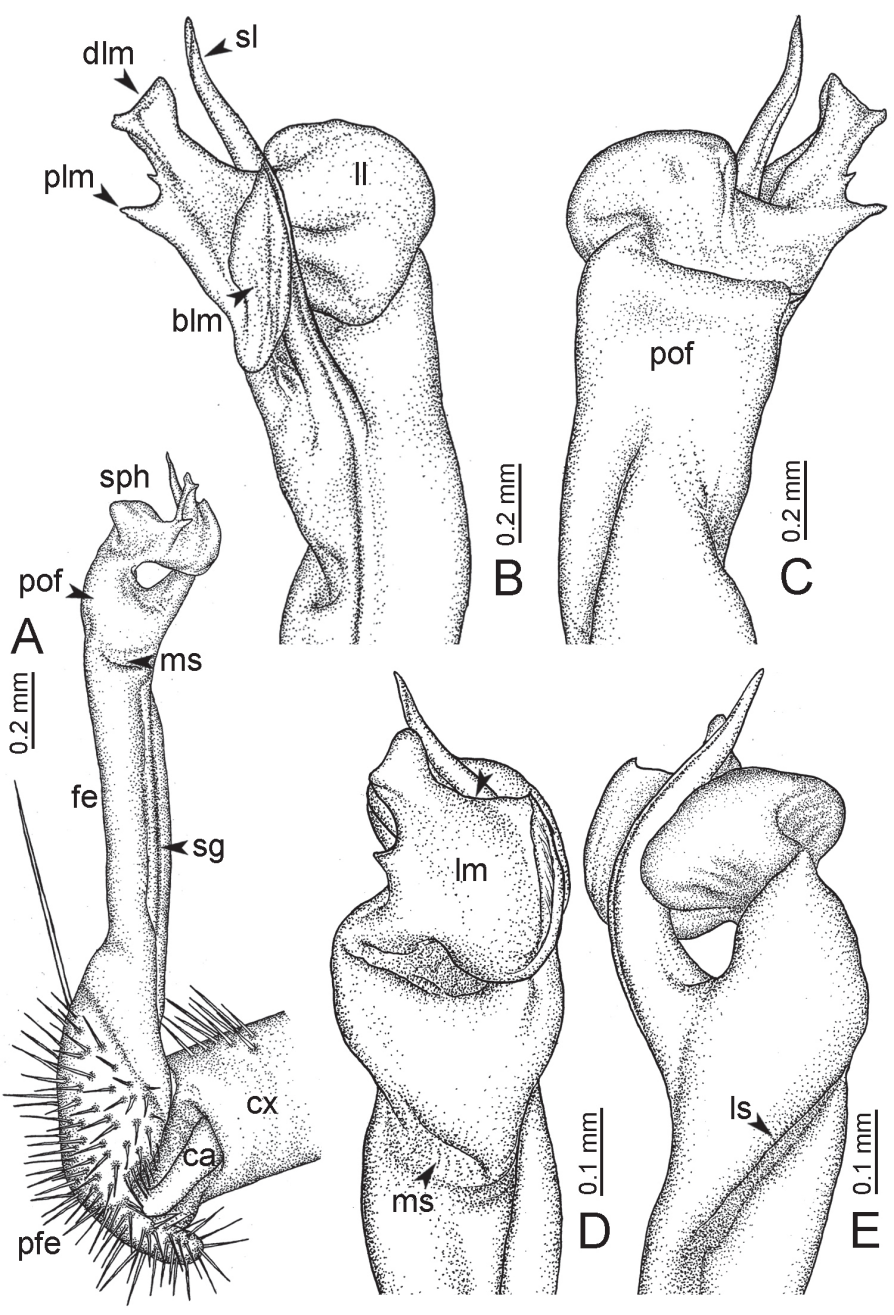
*Desmoxytes golovatchi* sp. n. (paratype) – right gonopod. **A** mesal view **B** dorsal view **C** ventral view **D** submesal view (arrow = indentation) **E** lateral view.

**Figure 54.**
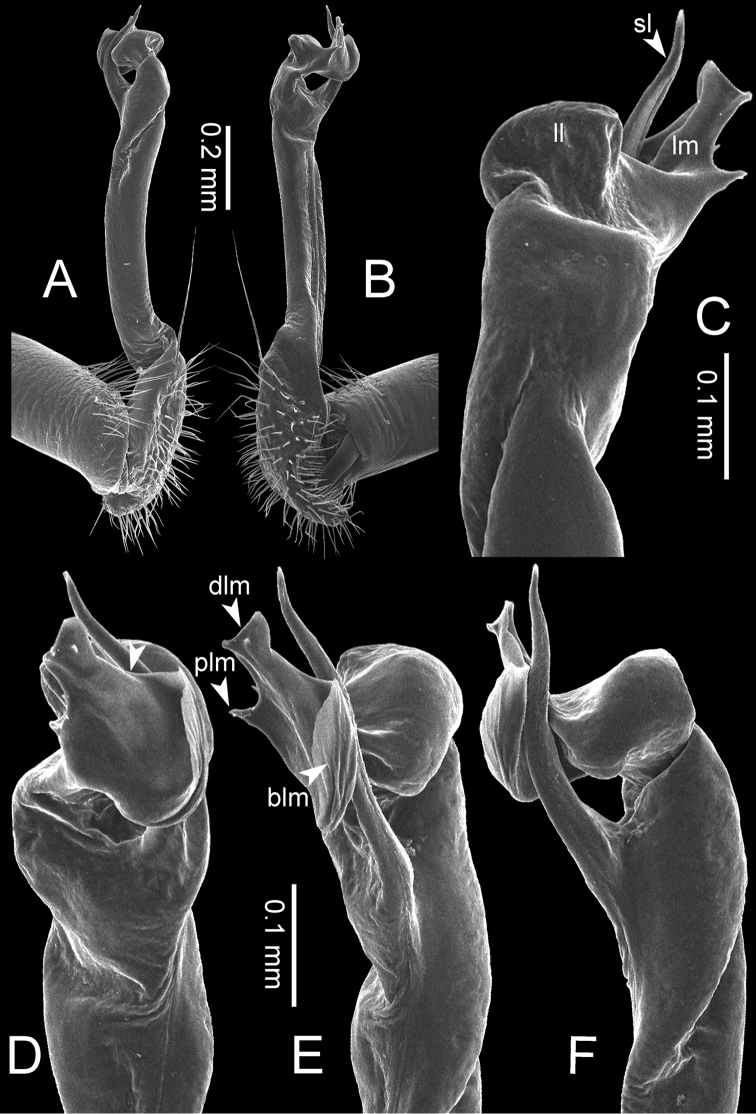
*Desmoxytes golovatchi* sp. n. (paratype) – right gonopod. **A** lateral view **B** mesal view **C** ventral view **D** subdorsal view (arrow = indentation) **E** dorsal view **F** subdorsal view.

Figs 49 , 50 , 51 , 52 , 53 , 54

########### Holotype.

Male (CUMZ), THAILAND, Kanchanaburi Province, Thong Pha Phum District, Prang Ka Sri Temple, 14°39'05"N, 98°40'08"E, ca. 107 m a.s.l., 15 August 2016, leg. C. Sutcharit, R. Srisonchai and ASRU members.

########### Paratypes.

19 males (CUMZ), 2 males (ZMUC), 1 male (ZMUM), 1 male (NHMW), 1 male (NHMUK), same data as holotype.

########### Further specimens, not paratypes

, **all from THAILAND, Kanchanaburi Province, Thong Pha Phum District**: 4 males (CUMZ), Tham Khao Noi Bureau of Monks (Wat Tham Khao Noi), 14°41'55"N, 98°31'33"E, ca. 225 m a.s.l., 21 August 2015, leg. E. Jeratthitikul and R. Srisonchai. 3 males, 1 female (CUMZ), Tham Khao Noi Bureau of Monks (Wat Tham Khao Noi), 14°41'55"N, 98°31'33"E, ca. 225 m a.s.l., 15 August 2016, leg. C. Sutcharit, R. Srisonchai and ASRU members. 6 males, 1 female (CUMZ), Wat Huay Charoen Srattha Tham, 14°39'27"N, 98°31'38"E, ca. 202 m a.s.l., 11 October 2015, leg. C. Sutcharit and R. Srisonchai. 1 female (CUMZ), Huay Kayeng Subdistrict, Tham Pong Chang Monastery, 14°44'38"N, 98°30'26"E, ca. 209 m a.s.l., 11 October 2015, leg. C. Sutcharit and R. Srisonchai. 3 males, 5 females (CUMZ), Wat Pak Lam Philok, 14°37'39"N, 98°34'27"E, ca. 280 m a.s.l., 11 October 2015, leg. C. Sutcharit and R. Srisonchai. 4 males, 1 female (CUMZ), Prang Ka Sri temple, 14°39'05"N, 98°40'08"E, ca. 107 m a.s.l., 24 July 2016, leg. P. Pimvichai and P. Prasankok.

**Sai Yok District**: 2 males, 2 females (CUMZ), Daowadueng Cave, 14°28'23"N, 98°50'04"E, ca. 132 m a.s.l., 15 August 2016, leg. C. Sutcharit, R. Srisonchai, and ASRU members.

########### Diagnosis.

Metaterga 2–8 with 2+2 tubercles/cones/spines in anterior row and 2+2 tubercles/cones/spines in posterior row; metaterga 9–19 with 3+3 tubercles/cones/spines in posterior row. Similar in these respects to *D. breviverpa*, *D. purpurosea*, *D. takensis*, and *D. taurina*. Differs from those by having: metaterga 9–19 with two rows of 3(2)+3(2) tubercles/cones/spines in anterior row; lamina lateralis (ll) round and compact; tip of process (plm) of lamina medialis terminating in a sharp spine; distal lobe (dlm) of lamina medialis long; broad lobe (blm) dorsally expanded.

########### Etymology.

The name honours Sergei I. Golovatch, a myriapodologist at the Institute for Problems of Ecology and Evolution, Russian Academy of Sciences, who has enthusiastically encouraged millipede research in Thailand, in recognition of his extensive work on the taxonomy of millipedes – especially in family Paradoxosomatidae.

########### Description.

SIZE: Length 27–31 mm (male), 32 mm (female); width of midbody metazona ca. 1.9 mm (male), 2.3 mm (female). Width of head < collum < body ring 2 = 3 = 4 < 5–16, thereafter body gradually tapering towards telson.

COLOUR (Fig. 49A–C): In life with body vivid pink; paraterga, metaterga, surface below paraterga and epiproct pink; head brown; antenna black, except distal part of antennomere 7 and antennomere 8 whitish; sterna and legs pinkish brown; a few basal podomeres brown to whitish.

ANTENNAE (Fig. 50D): Very long and slender, reaching to body ring 7 (male) and 5 (female) when stretched dorsally.

COLLUM (Fig. 50A): With 3 transverse rows of setae and setiferous tubercles, 4+4 anterior setae, 1+1 intermediate setae and 2+2 posterior tubercles (lateral seta in anterior row located almost at base of paraterga in some specimens; lateral tubercles in posterior row located almost halfway to intermediate row); paraterga of collum low, elevated at ca. 15°–20°, directed caudolaterad, with one conspicuous notch on lateral margin.

TEGUMENT: Slightly shining; collum and metaterga microgranulate; prozona finely shagreened; surface below paraterga finely microgranulate; sterna and epiproct relatively smooth.

METATERGA (Fig. 50A–C): With 2 transverse rows of setiferous tubercles, cones and spines; metaterga 2–8 with 2+2 anterior cones and 2(3)+2(3) posterior spines; metaterga 9–17 with 3(2)+3(2) anterior cones and 3(4)+3(4) posterior spines; metatergum 18 with with 3+3 anterior cones and 3+3 posterior cones; metatergum 19 with with 3+3 anterior tubercles and 3+3 posterior tubercles.

PARATERGA (Fig. 50E, F): Directed caudolaterad on body rings 2–17, elevated at ca. 45° (male) 40° (female); directed increasingly caudad on body rings 18 and 19; anterior margin with 2 distinct notches, on lateral margin of body rings 9, 10, 12, 13, 15–18 with tiny denticle near the tip.

TELSON (Fig. 51C–G): Tip subtruncate; lateral setiferous tubercles inconspicuous, apical tubercles small and inconspicuous. Hypoproct subtrapeziform; caudal margin round, with small inconspicuous setiferous tubercles.

STERNA (Fig. 52): Cross-impressions shallow. Sternal lobe between male coxae 4 subtrapeziform; base slightly enlarged; tip emarginate; swollen near pores.

LEGS (Fig. 51H–J): Long and slender. Male femora 5 and 6 strongly humped ventrally in middle portion.

GONOPODS (Figs 53, 54): Coxa (cx) longer than prefemur. Cannula (ca) slender. Prefemur (pfe) ca. 2/3 as long as femur. Femur (fe) long, slightly curved. Mesal sulcus (ms) and lateral sulcus (ls) conspicuous, deep. Postfemur (pof) conspicuous, ventrally wide. Solenophore (sph) well-developed: lamina lateralis (ll) round and compact (obvious when seen in ventral view): lamina medialis (lm) well-developed; process (plm) somewhat short, tip terminating in a sharp spine (in some specimens with a tiny spine-like process situated between process (plm) and distal lobe (dlm)); distal lobe (dlm) quite long, distally with two indistinctly separated lamellae; broad lobe (blm) dorsally expanded, indentation between broad lobe (blm) and distal love (dlm) very wide and shallow. Solenomere (sl) relatively long.

########### Distribution and habitat.

Known from the type locality and nearby areas in Kanchanaburi Province only. The type locality is situated on a small, isolated limestone mountain near Khwae Noi River. All specimens were found in limestone habitats (Fig. 49D).

This species is distributed along the limestone mountain ranges in Sai Yok and Thong Pha Phum districts. Based on many intensive surveys, the current distribution of the new species is evidently quite narrow, ca. 100 km^2^. Thus, *D. golovatchi* sp. n. should be regarded as a Thai endemic.

########### Remarks.

*Desmoxytes golovatchi* sp. n. is aposematic in its vivid pink body. During the field trips, this animal was noticeable by the contrast of its bright colour to green leaves or brown rocks, it thus was easy to see and collect after rain.

This species is morphologically similar to *D. breviverpa*, *D. purpurosea*, *D. takensis*, and *D. waepyanensis* sp. n. with which it shares colourful pink or red body colour, as well as further characters, viz., the same patterns of row of cones on metaterga (metaterga 2–8 with 2+2 cones in anterior row).

########### Coexisting species.

The new species was found in one place together with *D. octoconigera* sp. n. (see detail in *D. octoconigera* sp. n.), with *D. planata* at Wat Huay Charoen Srattha Tham and Tham Khao Noi Bureau of Monks, and with *D. purpurosea* at Daowadueng Cave. This species and *D. purpurosea* were hand-collected after rain when lots of them were climbing on vegetation and limestone rocks. Microhabitat differences have not yet been observed. Moreover, *D. planata* also occurs near the new species, but the habitats of these two species are clearly different: *D. planata* was found on humid cement and on construction materials whereas *D. golovatchi* sp. n. was seen crawling on limestone rock.

########## Desmoxytes octoconigera

Taxon classificationAnimaliaPolydesmidaParadoxosomatidae

Srisonchai, Enghoff & Panha sp. n.

http://zoobank.org/CC25ABC1-2675-4D84-BB8F-2A7D82EA1D25

**Figure 55.**
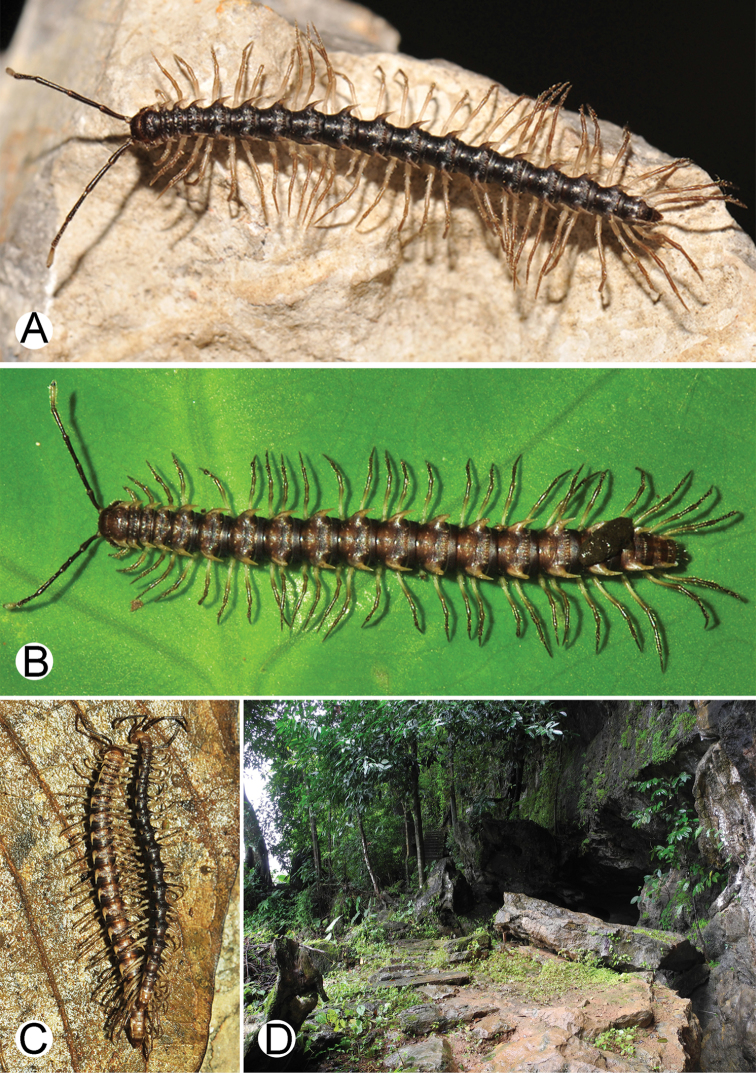
Photographs of live *Desmoxytes octoconigera* sp. n. and habitat. **A** male paratype **B** female paratype **C** mating couple **D** habitat.

**Figure 56.**
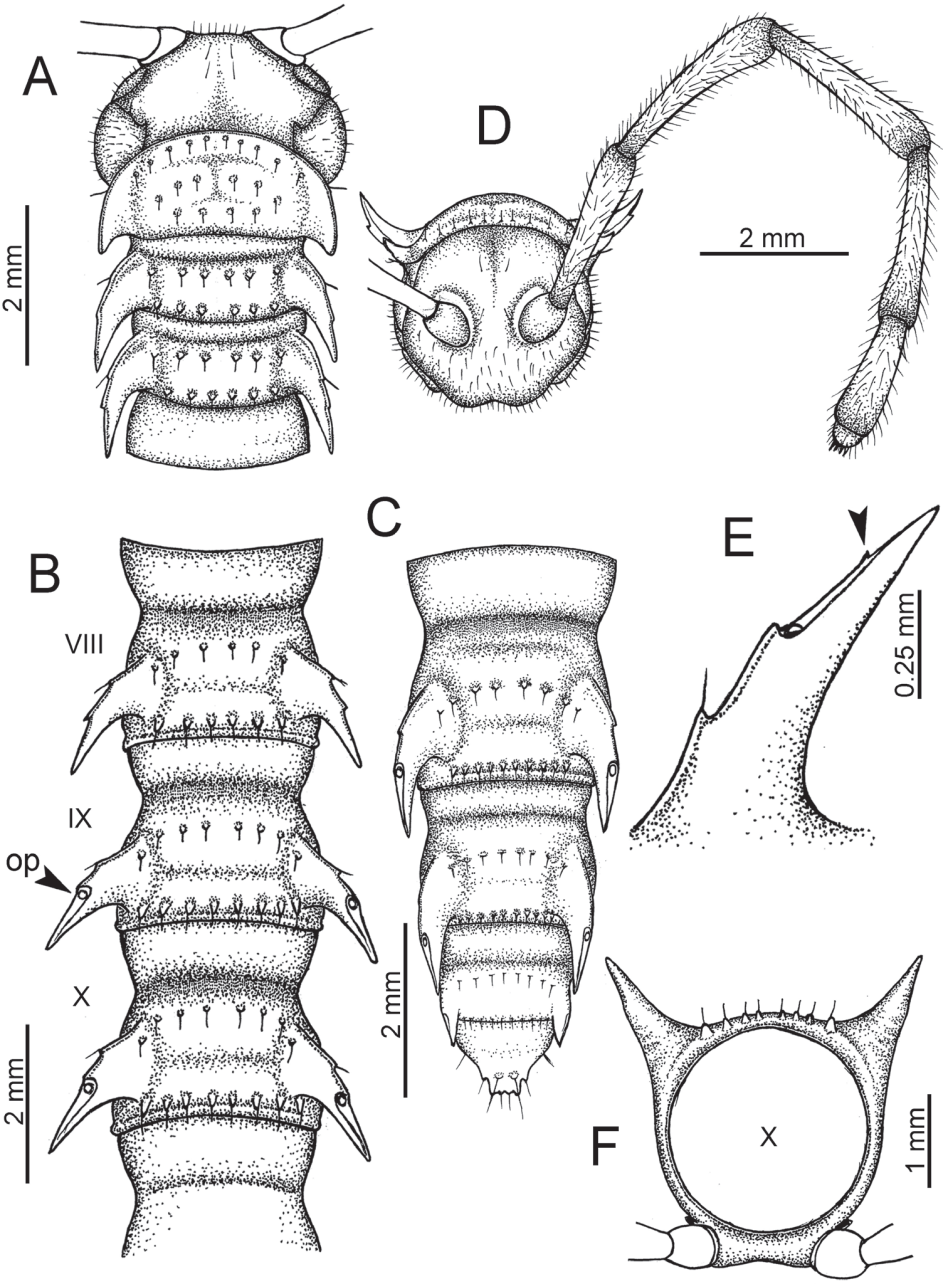
*Desmoxytes octoconigera* sp. n. (male paratype). **A** anterior body part **B** body rings 8–10 (op = ozopore) **C** posteriormost body rings and telson **D** head and antenna **E** paraterga of ring 10 (arrow = tiny denticle) **F** body ring 10.

**Figure 57.**
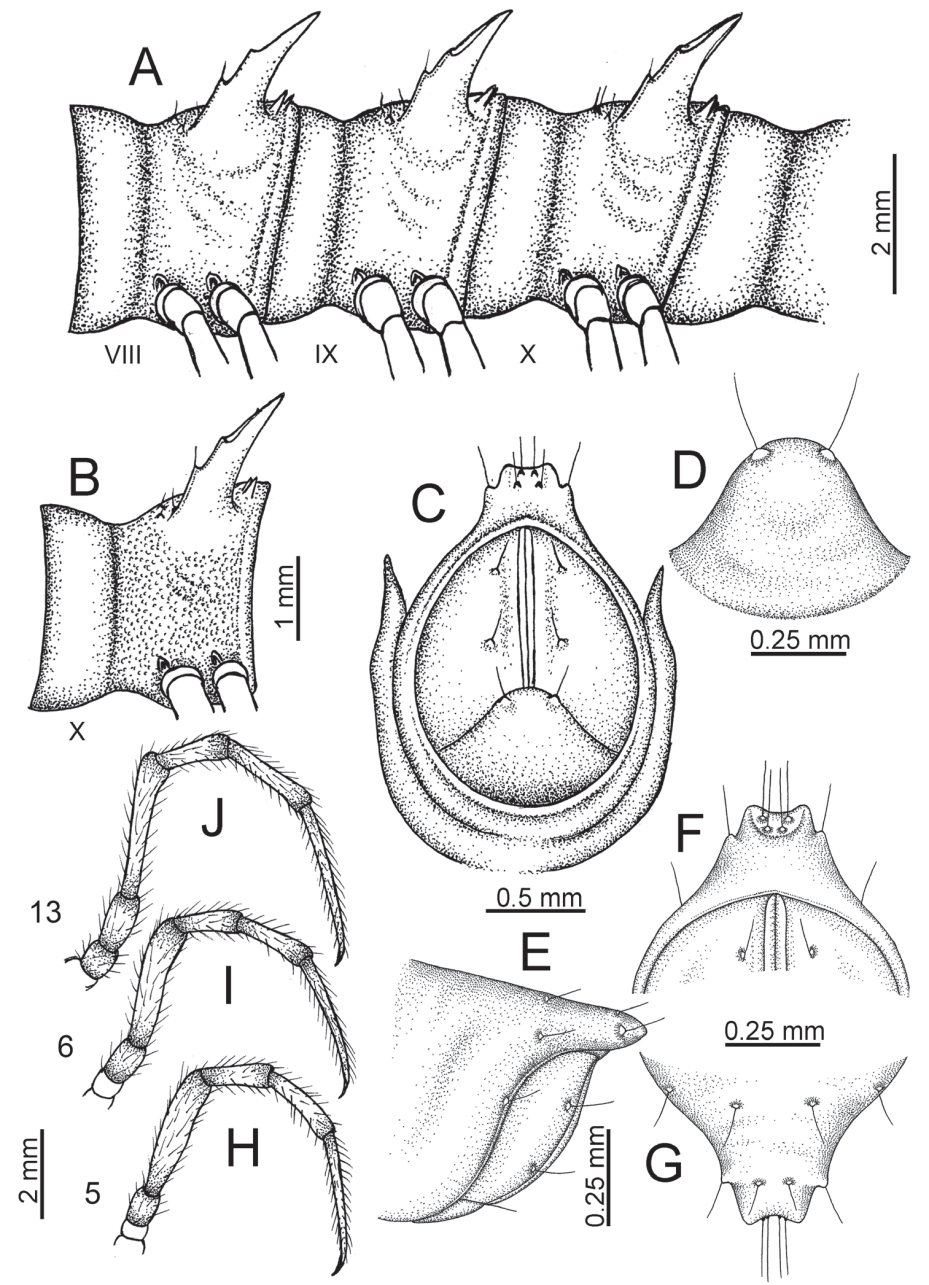
*Desmoxytes octoconigera* sp. n. (male paratype). **A** body rings 8–10 **B** sculpture of ring 10 **C, E** last ring and telson **D** hypoproct **F, G** epiproct **H** male leg 5 (right) **I** male leg 6 (right) **J** male leg 13 (right).

**Figure 58.**
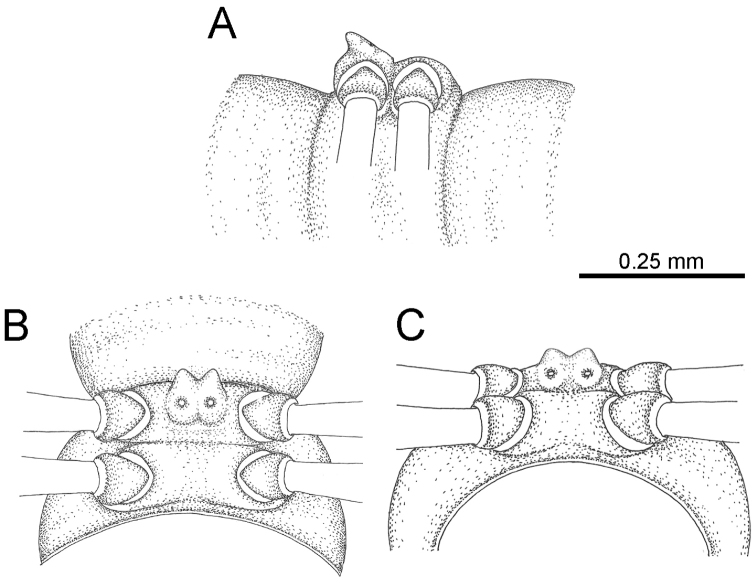
*Desmoxytes octoconigera* sp. n. (male paratype) – sternal lobe between male coxae 4. **A** lateral view **B** ventral view **C** caudal view.

**Figure 59.**
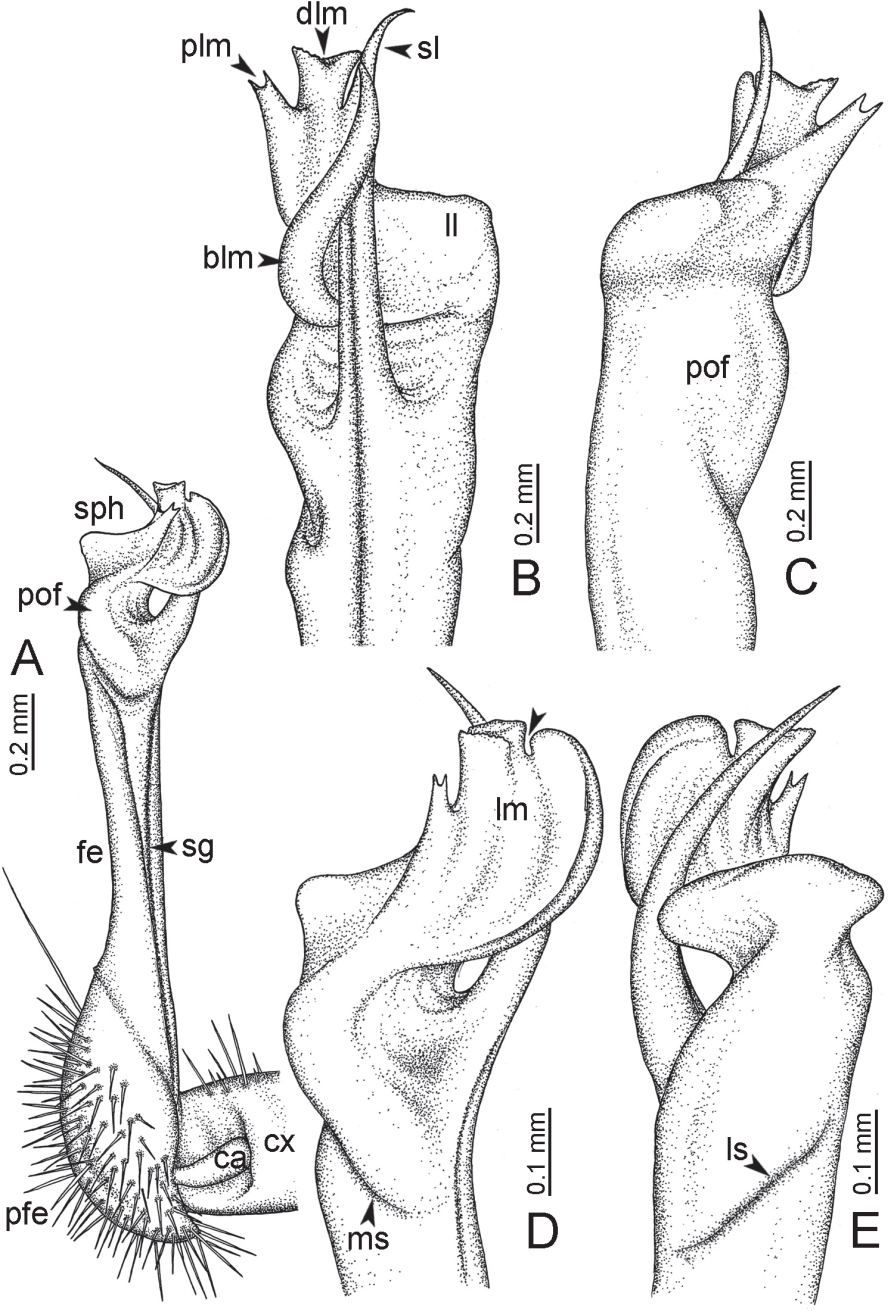
*Desmoxytes octoconigera* sp. n. (paratype) – right gonopod. **A** mesal view **B** dorsal view **C** ventral view **D** submesal view (arrow = indentation) **E** lateral view.

**Figure 60.**
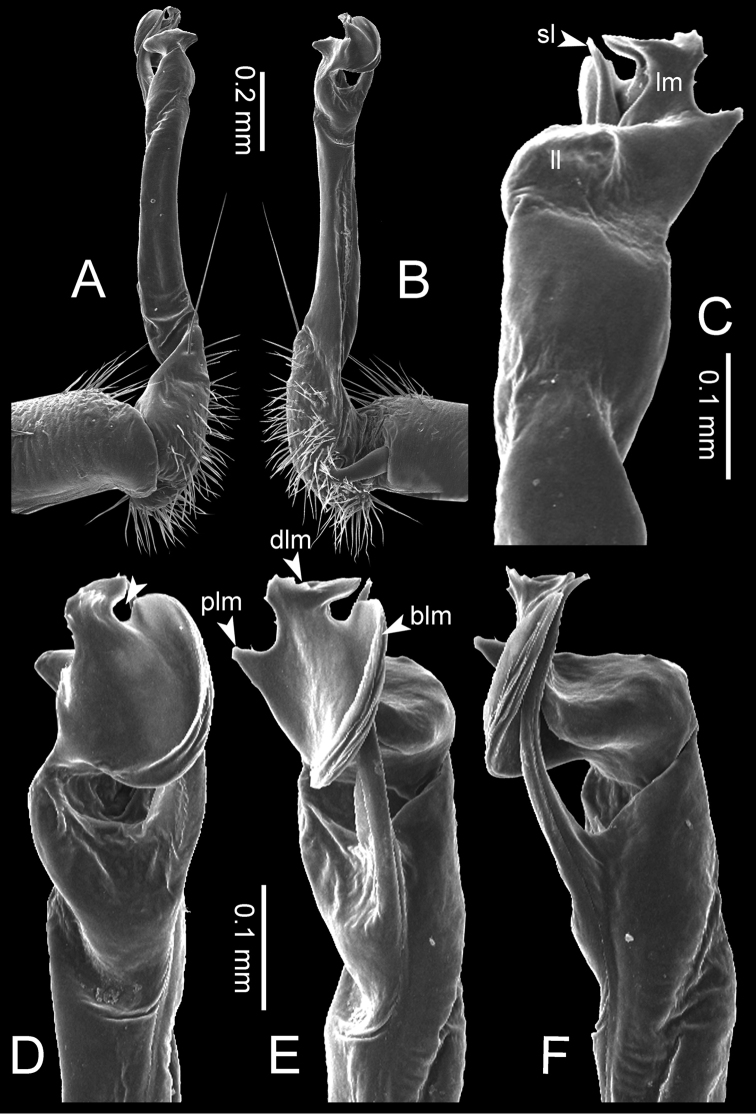
*Desmoxytes octoconigera* sp. n. (paratype) – right gonopod. **A** lateral view **B** mesal view **C** ventral view **D** subdorsal view (arrow = indentation) **E** dorsal view **F** subdorsal view.

Figs 55 , 56 , 57 , 58 , 59 , 60

########### Holotype.

Male (CUMZ), THAILAND, Kanchanaburi Province, Sangkhla Buri District, Wat Tham Kaeo Sawan Bandal, 15°18'18"N, 98°24'57"E, ca. 334 m a.s.l., 16 August 2016, leg. C. Sutcharit, R. Srisonchai and ASRU members.

########### Paratypes.

22 males, 11 females (CUMZ), 2 males (ZMUC), same data as holotype; 1 female; 1 broken female (CUMZ), same locality as holotype, 10 July 2009, leg. S. Panha and ASRU members.

########### Further specimens, not paratypes

, **all from THAILAND, Kanchanaburi Province, Sangkhla Buri District**: 3 broken males, 1 female, 2 broken and mixed specimens, 2 broken and mixed females? (CUMZ), Kra Teng Cheng Waterfall, 15°01'30"N, 98°36'05"E, ca. 208 m a.s.l., 10 July 2009, leg. C. Sutcharit, R. Srisonchai and ASRU members. 1 broken male missing gonopods, 5 males, 2 females (CUMZ), Kroeng Krawia Waterfall, 14°58'53"N, 98°37'54"E, ca. 255 m a.s.l., 10 July 2009, leg. S. Panha and ASRU members. 2 males, 3 juveniles (CUMZ), Takhian Thong Waterfall, 15°17'58"N, 98°26'56"E, ca. 241 m a.s.l., 10 July 2009, leg. S. Panha and ASRU members. 1 broken male (CUMZ), mountain near the Three Pagodas Pass, 15°18'20"N, 98°24'01"E, ca. 368 m a.s.l., 19 December 2010, leg. S. Panha and ASRU members. 3 males (CUMZ), Kra Teng Cheng Waterfall, 15°01'30"N, 98°36'05"E, ca. 208 m a.s.l., 16 August 2016, leg. C. Sutcharit, R. Srisonchai and ASRU members. 2 males, 2 females (CUMZ), near Ban Songkaria (Songkaria Village), limestone mountain, 15°13'01"N, 98°27'06"E, ca. 206 m a.s.l., 16 August 2016, leg. C. Sutcharit, R. Srisonchai and ASRU members. 7 males (CUMZ), Wat Tham Sukhlo, 15°02'14"N, 98°34'59"E, ca. 196 m a.s.l., 16 August 2016, leg. C. Sutcharit, R. Srisonchai and ASRU members.

**Thong Pha Phum District**: 4 males (CUMZ), Kroeng Krawia Checkpoint, 14°56'32"N, 98°40'11"E, ca. 347 m a.s.l., 16 August 2016, leg. C. Sutcharit, R. Srisonchai and ASRU members. 2 males, 1 female (CUMZ), Tham Khao Noi Bureau of Monks (Wat Tham Khao Noi), 14°41'55"N, 98°31'33"E, ca. 225 m a.s.l., 21 August 2015, leg. E. Jeratthitikul and R. Srisonchai. 2 males (CUMZ), Tham Khao Noi Bureau of Monks (Wat Tham Khao Noi), 14°41'55"N, 98°31'33"E, ca. 225 m a.s.l., 15 August 2016, leg. C. Sutcharit, R. Srisonchai and ASRU members.

########### Diagnosis.

Differs from all other *Desmoxytes* species by the combination of the following characters; collum with three rows of 5+5 anterior, 1(2)+1(2) intermediate, and 3+3 posterior setiferous tubercles; metaterga 2–8 with two rows of 3+3 (anterior row) and 3+3 (posterior row) setiferous cones; metaterga 9–17 with two rows of 4(3)+4(3) (anterior row) and 4(5)+4(5) (posterior row) setiferous cones; sternal cone between male coxae 4 incompletely bilobed, cordiform; lateral lamella of distal lobe (dlm) on lamina medialis broad and thin, demarcated from broad lobe (blm) by a deep indentation.

########### Etymology.

The name is a Latin adjective, referring to the two rows each with eight setiferous cones on metaterga 9–17.

########### Description.

SIZE: Length 24–30 mm (male), 30–32 mm (female); width of midbody metazona ca. 1.75 mm (male), 2.0 mm (female). Width of head > collum = body ring 2 = 3 = 4 < 5–16, thereafter body gradually tapering toward telson.

COLOUR (Fig. 55A–C): In life with body dark brown (male), brown (female); paraterga and sterna brown to whitish; metaterga, surface below paraterga and antenna dark brown (except antennomeres 6–8 whitish); head and epiproct brown; a few basal podomeres brown to whitish.

ANTENNAE (Fig. 56D): Very long and slender, reaching to body ring 7–8 (male) and 6 (female) when stretched dorsally.

COLLUM (Fig. 56A): With 3 transverse rows of setiferous tubercles, 5+5 anterior, 1(2)+1(2) intermediate and 3+3 posterior tubercles (lateral tubercles of anterior row located almost at base of paraterga in some specimens); paraterga of collum low, elevated at ca. 20°, directed caudad, with one distinct notch on lateral margin.

TEGUMENT: Quite dull, slightly shining; collum and metaterga coarsely microgranulate; prozona finely shagreened; surface below paraterga finely microgranulate; sterna and epiproct smooth.

METATERGA (Fig. 56A–C): With 2 transverse rows of setae, setiferous tubercles and setiferous cones; metaterga 2–8 with 3+3 anterior and 3+3 posterior cones; metaterga 9–17 with 4(3)+4(3) anterior and 4(5)+4(5) posterior cones; metatergum 18 with 4+4 anterior and 4+4 posterior tubercles; metatergum 19 with 4+4 anterior and 4+4 posterior setae.

PARATERGA (Fig. 56E, F): Directed caudolaterad on body rings 2–17, elevated at ca. 50° (male) 45° (female); directed increasingly caudad on body rings 18 and 19; anterior margin with 2 distinct notches, on lateral margin of body rings 9, 10, 12, 13, 15–18 with tiny denticle near the tip.

TELSON (Fig. 57C–G): Epiproct: tip subemarginate; lateral setiferous tubercles small but conspicuous; apical tubercles conspicuous. Hypoproct subtrapeziform; caudal margin round, with inconspicuous setiferous tubercles.

STERNA (Fig. 58): Cross-impressions shallow. Sternal lobe between male coxae 4 swollen, tip emarginate to incompletely bilobed, cordiform when seen in ventral view.

LEGS (Fig. 57H–J): Very long and slender. Male femora 5 and 6 swollen in middle part.

GONOPODS (Figs 59, 60): Coxa (cx) longer than prefemur. Cannula (cx) slender. Prefemur (pfe) ca. 2/3 as long as femur. Femur (fe) long and slender. Mesal sulcus (ms) and lateral sulcus (ls) very deep and wide. Postfemur (pof) conspicuous, ventrally wide. Solenophore (sph) well-developed: lamina lateralis (ll) swollen, lobe-like when seen in ventral view, conspicuous: lamina medialis (lm) well-developed; process (plm) long, directed mesoanteriad, tip almost blunt (in some specimens tip bifurcating into two sharp small spines); distal lobe (dlm) well-developed, distally with two lamellae (lateral lamella broad and thin, projecting laterad, *in situ* terminating close to tip of solenomere (sl); mesal lamella shorter than lateral one, apical margin serrate); broad lobe (blm) thick, distinctly demarcated from distal lobe (dlm) by a very deep and narrow indentation. Solenomere (sl) quite long.

########### Distribution and habitat.

*D. octoconigera* sp. n. is known only from Kanchanaburi Province. At the type locality where the holotype and the majority of the paratypes were collected, the animals were crawling on litter inside rock holes; some specimens were seen crawling on rock walls near the cave.

Interestingly, some specimens of *D. planata* were also found near the cave, however, according to our surveys this species is usually found in places with human activity. We assume that it was probably accidentally introduced to the cave by human actions. Therefore, *D. planata* and the new species might not share microhabitat, although they live syntopically.

The new species and *D. golovatchi* sp. n. are sympatric in one location at Tham Khao Noi Bureau of Monks (Wat Tham Khao Noi), and they both have narrow distribution ranges (<100 km^2^). Currently, the type locality of *D. octoconigera* sp. n. is situated in an area with considerable human activity (Bureau of Monks and tourist cave), where the forest habitat is cut every year.

We have tried in vain to find this species in another area nearby. Given the narrow distribution range, the new species is probably endemic to Thailand.

########### Remarks.

There is considerable variation in tip of process (plm) of lamina medialis within populations. The process tip in some specimens is bifurcate as two small spines whereas in other specimens it may be relatively blunt.

########### Coexisting species.

*Desmoxytes golovatchi* sp. n. and *D. planata*.

########## Desmoxytes perakensis

Taxon classificationAnimaliaPolydesmidaParadoxosomatidae

Srisonchai, Enghoff & Panha sp. n.

http://zoobank.org/AE9D6B74-2471-41E9-82C2-8E5AE25596C8

**Figure 61.**
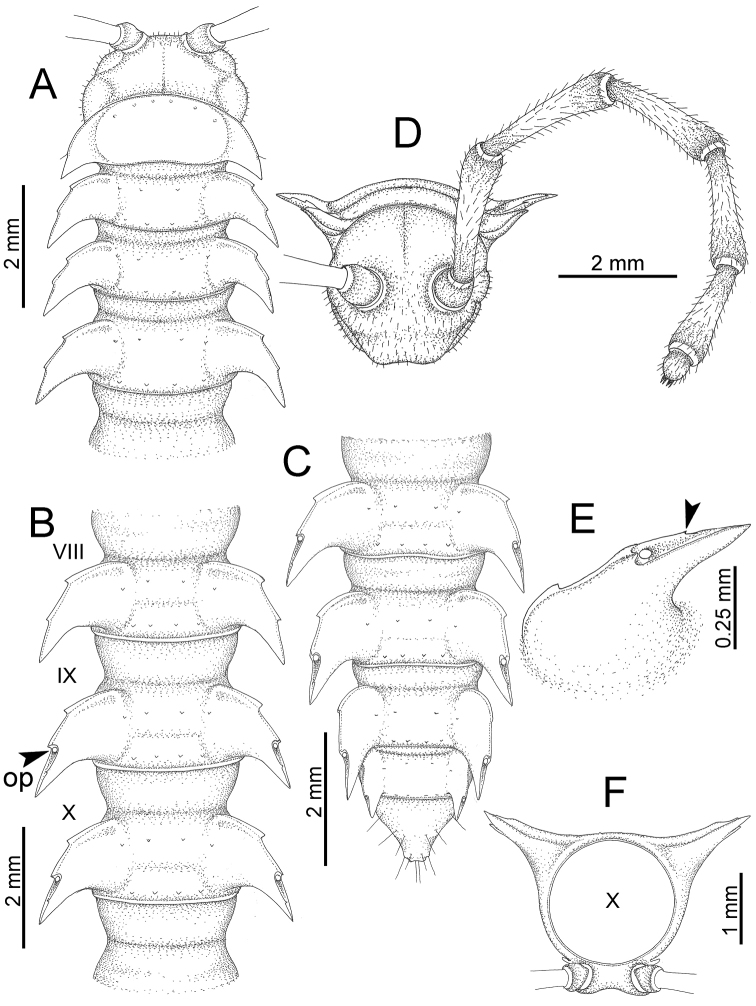
*Desmoxytes perakensis* sp. n. (male paratype). **A** anterior body part **B** body rings 8–10 (op = ozopore) **C** posteriormost body rings and telson **D** head and antenna **E** paraterga of ring 10 (arrow = tiny denticle) **F** body ring 10.

**Figure 62.**
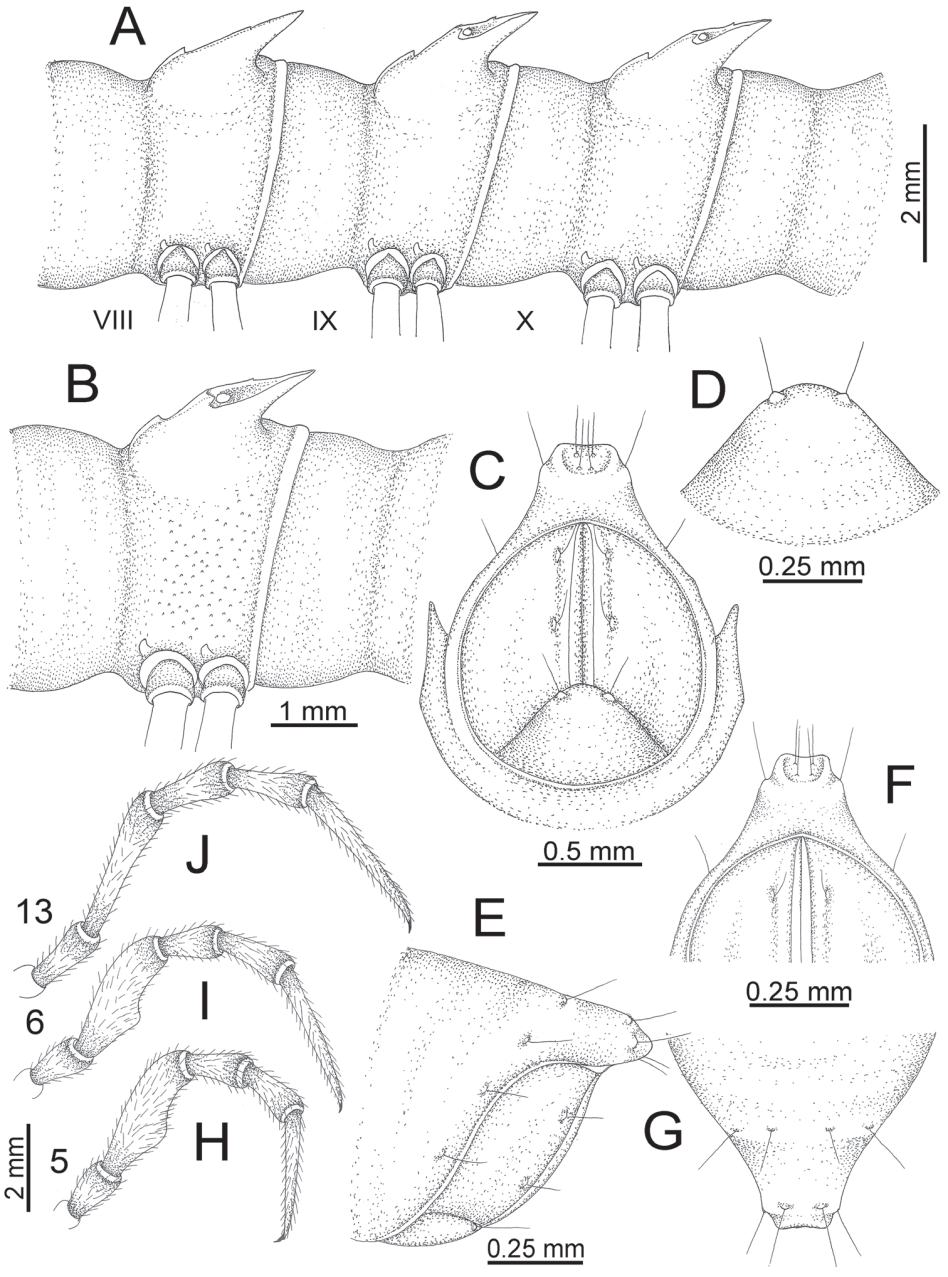
*Desmoxytes perakensis* sp. n. (male paratype). **A** body rings 8–10 **B** sculpture of ring 10 **C, E** last ring and telson **D** hypoproct **F, G** epiproct **H** male leg 5 (right) **I** male leg 6 (right) **J** male leg 13 (right).

**Figure 63.**
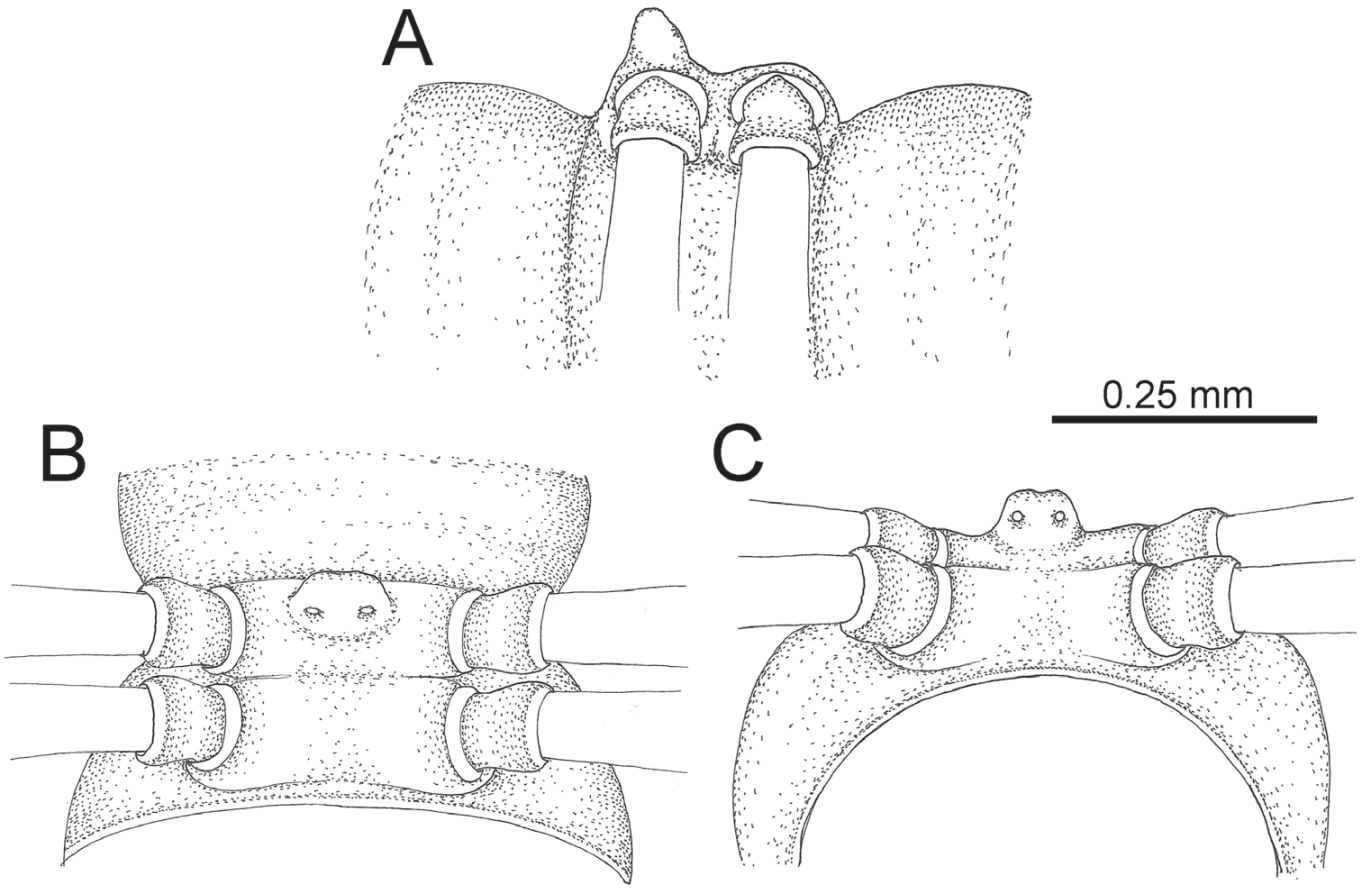
*Desmoxytes perakensis* sp. n. (male paratype) – sternal lobe between male coxae 4. **A** lateral view **B** ventral view **C** caudal view.

**Figure 64.**
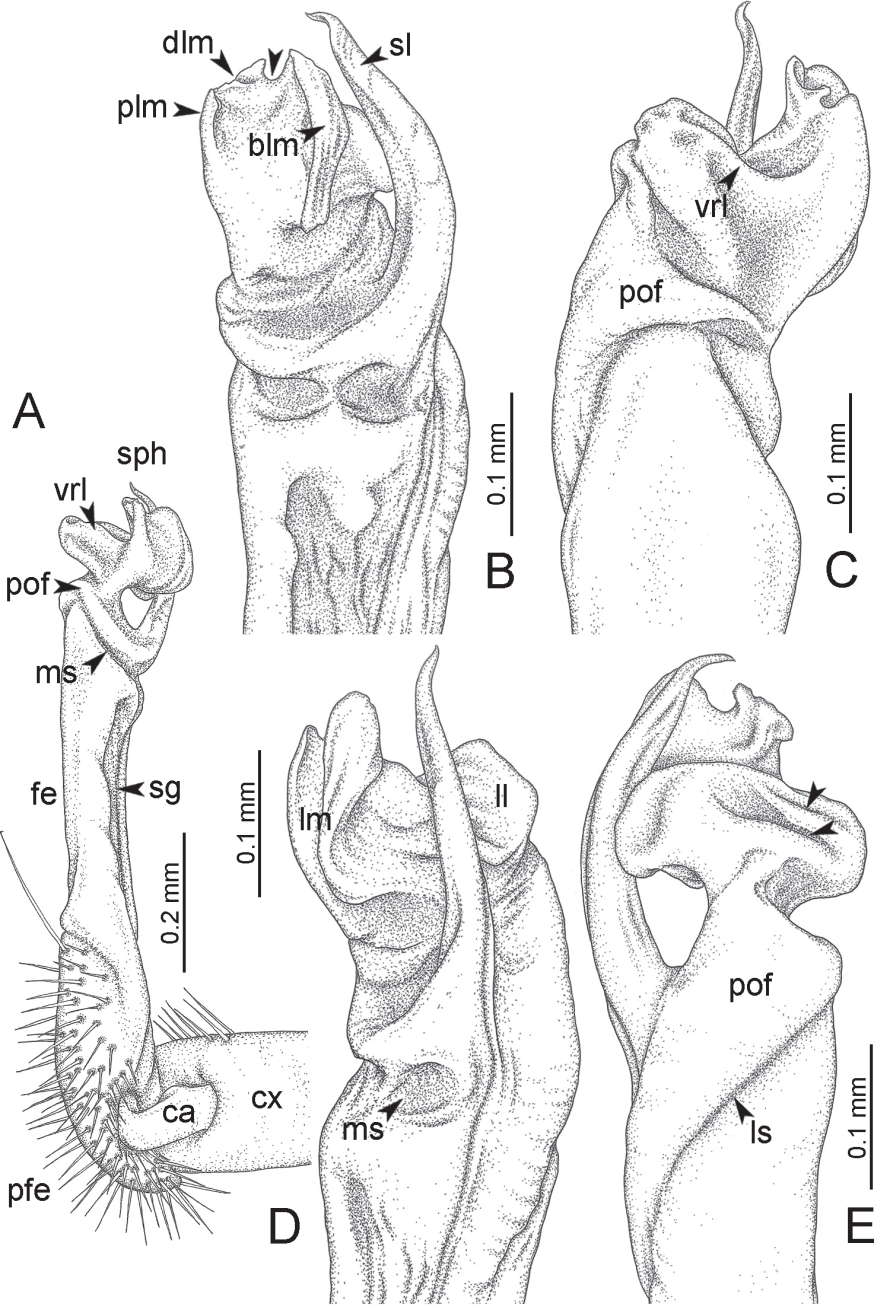
*Desmoxytes perakensis* sp. n. (paratype) – right gonopod. **A** mesal view **B** submesal view (arrow = indentation) **C** ventral view **D** dorsal view **E** lateral view (arrows = furrows).

**Figure 65.**
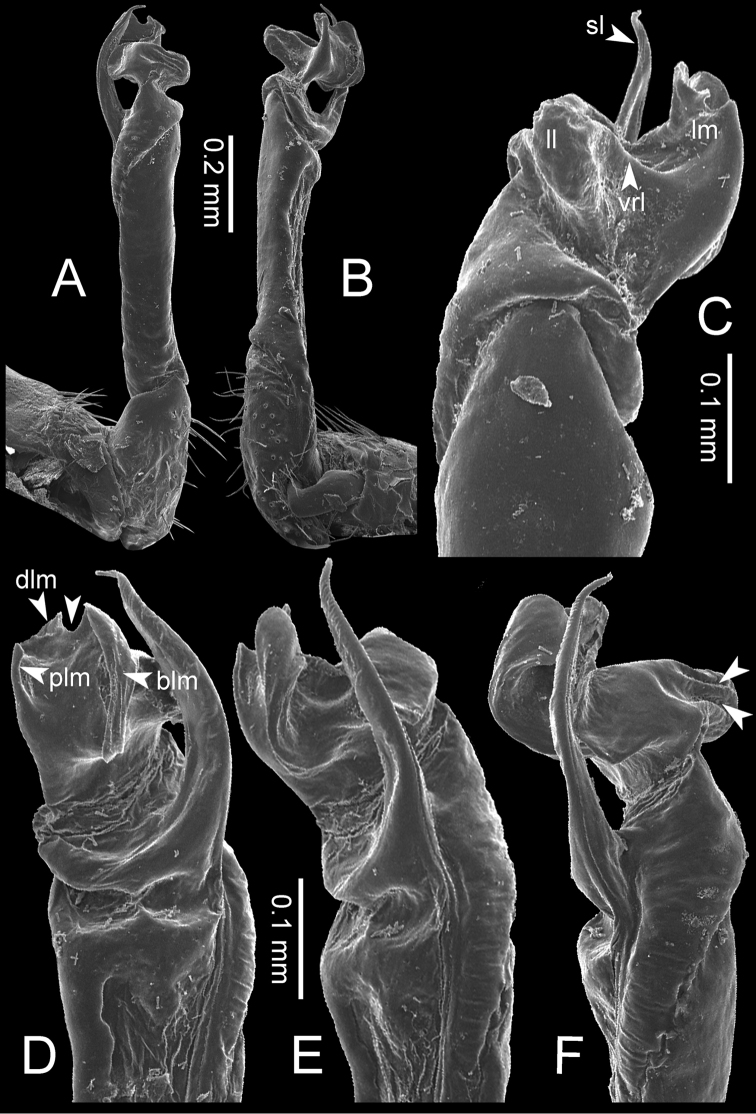
*Desmoxytes perakensis* sp. n. (paratype) – right gonopod. **A** lateral view **B** mesal view **C** ventral view **D** subdorsal view (arrow = indentation) **E** dorsal view **F** subdorsal view (arrows = furrows).

Figs 61 , 62 , 63 , 64 , 65

########### Holotype.

Male (CUMZ), MALAYSIA, Perak, Ipoh, Ulu Kinta, near The Lost World Tambun Theme Park, limestone mountain, 4°37'45"N, 101°09'21"E, ca. 73 m a.s.l., 27 September 2007, leg. B. W. Ng, S. Panha and ASRU members.

########### Paratypes.

4 males (CUMZ), same data as holotype.

########### Diagnosis.

Differing from all other species, except *D. delfae* and *D. aurata* sp. n., by having a low degree of elevation of paraterga, femora 5 and 6 strongly humped ventrally in middle part, collum with a row of 3+3 anterior setae and metaterga 2–18 with rows of 2+2 anterior and 2+2 posterior small tubercles. Differs from *D. delfae* and *D. aurata* sp. n. by having paraterga wider than those species; lamina lateralis (ll) with two distinct furrows ventrolaterally; process (plm) of lamina medialis lamellar, tip blunt.

########### Etymology.

The name is a Latin adjective referring to the type locality.

########### Description.

SIZE: Length 24–26 mm (male), 27–29 mm (female); width of midbody metazona ca. 1.9 mm (male), 2.2 mm (female). Width of head < collum < body ring 2 = 3 < 4 < 5–16, thereafter body gradually tapering towards telson.

COLOUR: In life with bright orange. Colour in alcohol: after 10 years in alcohol pale yellow to whitish.

ANTENNAE (Fig. 61D): Moderately long and slender, reaching to body ring 6 (male) and 5 (female) when stretched dorsally.

COLLUM (Fig. 61A): With 1 transverse anterior row of 3+3 setae; paraterga of collum low, almost horizontal, directed caudolaterad, with two inconspicuous setiferous notches on lateral margin.

TEGUMENT: Slightly shining and smooth; collum, metaterga, sterna and epiproct smooth; prozona finely shagreened; surface below paraterga finely microgranulate.

METATERGA (Fig. 61A–C): With 2 transverse rows of small, inconspicuous setae and tubercles; metaterga 2–18 with 2+2 anterior and 2+2 posterior tubercles; metatergum 19 with 2+2 anterior and 2+2 posterior setae.

PARATERGA (Fig. 61E, F): Directed caudolaterad on body rings 2–17, elevated at ca. 25°–30° (male) 25° (female); directed increasingly caudad on body rings 18 and 19; anterior margin with 2 distinct notches, on lateral margin of body rings 9, 10, 12, 13, 15–18 with tiny denticle near the tip.

TELSON (Fig. 62C–G): Epiproct: tip truncate; lateral setiferous tubercles and apical tubercles inconspicuous. Hypoproct subtriangular; caudal margin round, with very small and inconspicuous setiferous tubercles.

STERNA (Fig. 63): Cross-impressions shallow. Sternal lobe between male coxae 4 subtrapeziform, tip subtruncate, base slightly enlarged.

LEGS (Fig. 62H–J): Long and slender. Male femora 5 and 6 strongly humped ventrally in middle portion.

GONOPODS (Figs 64, 65): Coxa (cx) longer than prefemur. Cannula (ca) somewhat stout. Prefemur (pfe) ca. 2/3 as long as femur. Femur (fe) quite long and slender. Mesal sulcus (ms) and lateral sulcus (ls) conspicuous, very deep. Postfemur (pof) conspicuous, ventrally narrow and short. Solenophore (sph) well-developed: lamina lateralis (ll) swollen, quite thin, anterolaterally with two distinct furrows, dorsolaterally subsided, ventral ridge (vrl) conspicuous: lamina medialis (lm) well-developed; process (plm) short, lamellar, tip blunt; distal lobe (dlm) distally with two lamellae (mesal lamella smaller than lateral one); broad lobe (blm) dorsally somewhat thick, demarcated from distal lobe (dlm) by conspicuous indentation. Solenomere (sl) long.

########### Distribution and habitat.

Known only from the type locality. Currently, the habitats at this site are being destroyed and disturbed by human activities, e.g. deforestation for tourist attractions. Our extensive surveys in 2007 allow us to consider this species to be endemic to Malaysia.

########### Remarks.

*D. perakensis* sp. n. is morphologically similar to *D. delfae* and *D. aurata* sp. n.

Unfortunately, we did not photograph a living specimen during the field trip; however, one collector noticed orange colouration similar to *D. aurata* sp. n. and *D. delfae*.

########### Coexisting species.

None known.

########## Desmoxytes pinnasquali

Taxon classificationAnimaliaPolydesmidaParadoxosomatidae

Srisonchai, Enghoff & Panha, 2016

Fig. 66

Desmoxytes pinnasquali Srisonchai, Enghoff & Panha, 2016: 107.

########### Material examined. Holotype.

Male (CUMZ), THAILAND, Phitsanulok Province, Noen Maprang District, near Pra Tham Mans Monastery (Tham Wangdaeng), 16°41'40"N, 100°40'42"E, ca. 76 m a.s.l., 22 August 2014, leg. S. Panha, C. Sutcharit and U. Banthaowong.

########### Paratypes.

22 males, 5 females (CUMZ), 2 males, 1 female (ZMUC), same data as holotype. 10 males, 17 females (CUMZ), THAILAND, Phitsanulok Province, Noen Maprang District, near Pra Tham Mans Monastery (Tham Wangdaeng), 16°41'40"N, 100°40'42"E, ca. 76 m a.s.l., 23 July 2008, leg. N. Likhitrakarn. 2 females (CUMZ), THAILAND, Phitsanulok Province, Noen Maprang District, near Pra Tham Mans Monastery (Tham Wangdaeng), 16°41'40"N, 100°40'42"E, ca. 76 m a.s.l., 8 September 2009, leg. U. Bantaowong and R. Chanabun. 3 males, 6 females (CUMZ), THAILAND, Phitsanulok Province, Noen Maprang District, near Pa Ma Muang monastery, 16°34'00"N, 100°41'41"E, ca. 113 m a.s.l., 23 July 2008, leg. C. Sutcharit and P. Tongkerd.

########### Further specimens, 

**all from THAILAND, Phitsanulok Province, Noen Maprang District**: 1 male, 2 females (CUMZ), Pra Tham Mans Monastery (Tham Wangdaeng), 16°41'40"N, 100°40'42"E, ca. 76 m a.s.l., 29 July 2016, leg. P. Pimvichai, T. Backeljau, P. Prasankok and N. Nantarat. 3 males, 4 females (CUMZ), Pa Ma Muang Bureau of Monks (= Pa Ma Muang Monastery), 16°34'00"N, 100°41'41"E, ca. 113 m a.s.l., 29 July 2016, leg. P. Pimvichai, T. Backeljau, P. Prasankok and N. Nantarat.

########### Type locality.

THAILAND, Phitsanulok Province, Noen Maprang District, Pra Tham Mans Monastery (Tham Wangdaeng).

########### Diagnosis.

Differs from all other *Desmoxytes* species by the combination of the following characters; sternal lobe between male coxae 4 subrectangular or subtrapeziform, flattened when seen in lateral view; apical tubercles of epiproct conspicuous, long, digitiform; process (plm) of lamina medialis sharkfin-like, long.

########### Redescription (updated from Srisonchai et al. 2016).

SIZE: Length 24–30 mm (male), 28–33 mm (female); width of midbody metazona ca. 2.0–2.2 mm (male), 2.4 mm (female). Width of head < collum < body ring 2 < 3 ≤ 4 < 5–16, thereafter body gradually tapering toward telson.

COLOUR: In life with body vivid pink or brownish pink; paraterga vivid pink; metaterga and surface below paraterga brownish pink; head and antenna blackish brown (except distal part of antennomere 7 and antennomere 8 whitish); legs pink or brownish pink; a few basal podomeres whitish pink; sterna brown or pinkish brown; epiproct pink. Colour in alcohol: after two years changed to pale brown.

ANTENNAE: Long and slender, reaching to body ring 5 or end of 5 (male), and 4 (female) when stretched dorsally.

COLLUM: With 3 transverse rows of setiferous tubercles, 3(4)+3(4) anterior, 1+1 intermediate and 2+2 posterior tubercles (excluding small setiferous notches at base of collum paraterga); paraterga of collum low, elevated at ca. 10°–15°, directed almost laterad, with two setiferous notches on lateral margin (first notch located at the base of paratergum, second one conspicuous).

TEGUMENT: Moderately shining; collum and metaterga coarsely microgranulate; prozona finely shagreened; surface below paraterga finely microgranulate; paraterga, sterna and epiproct smooth.

METATERGA: With 2 transverse rows of setiferous tubercles, setiferous cones and setiferous spines; metaterga 2–17 with 2+2 anterior cones and 2+2 posterior spines; metaterga 18 and 19 with 2+2 anterior tubercles and 2+2 posterior tubercles.

PARATERGA: Directed caudolaterad on body rings 2–17, elevated at ca. 45° (male) 40° (female); directed increasingly caudad on body rings 18 and 19; anterior margin with 2 distinct notches, on lateral margin of body rings 9, 10, 12, 13, 15–18 with tiny denticle near the tip.

TELSON: Epiproct: tip extremely concave; lateral setiferous tubercles inconspicuous, very short; apical tubercles conspicuous, very long, digitiform. Hypoproct subsemicircular; caudal margin round, with big and conspicuous setiferous tubercles.

STERNA: Cross-impressions shallow. Sternal lobe between male coxae 4 swollen, usually subrectangular (in some specimens subtrapeziform), flat when seen in lateral view, tip subtruncate.

LEGS: Very long and slender. Male femora 5 and 6 moderately humped ventrally in middle part (hump of femora 6 bigger than 5).

**Figure 66.**
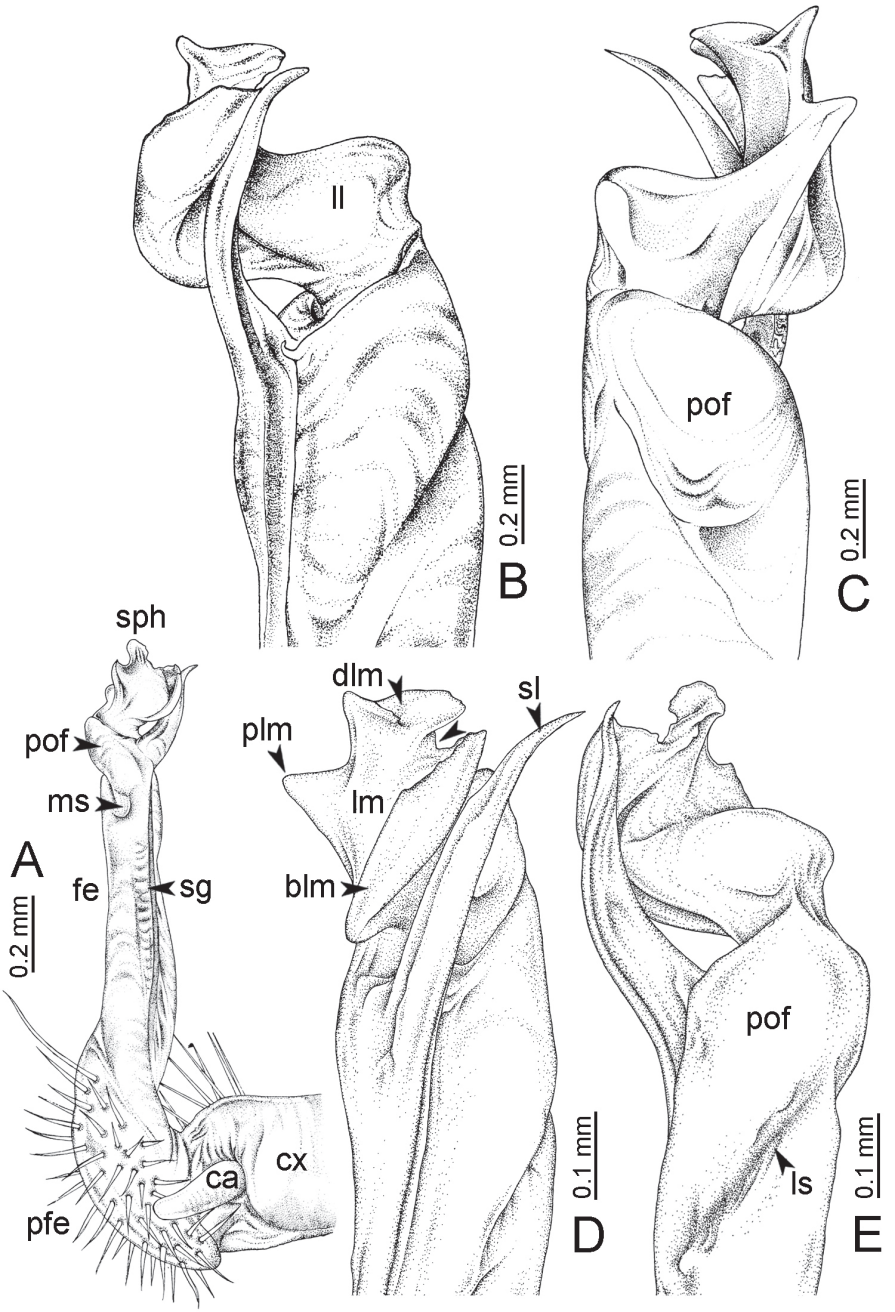
*Desmoxytes pinnasquali* Srisonchai et al., 2016 (paratype) – right gonopod (modified from Srisonchai et al. 2016). **A** mesal view **B** dorsal view **C** ventral view **D** subdorsal view (arrow = indentation) **E** lateral view.

GONOPODS (Fig. 66): Coxa (cx) longer than prefemur. Cannula (ca) slender. Prefemur (pfe) ca. 2/3 as long as femur. Femur (fe) long and slender. Mesal sulcus (ms) and lateral sulcus (ls) very deep and wide. Postfemur (pof) conspicuous, ventrally quite wide. Solenophore (sph) well-developed: lamina lateralis (ll) swollen: lamina medialis (lm) well-developed; process (plm) long, sharkfin-like, tip slightly blunt (in some specimens slightly sharp), directed mesad; distal lobe (dlm) distally with two lamellae (mesal lamella slightly smaller than second one); broad lobe (blm) thick, obviously demarcated from distal lobe (dlm) by a wide and shallow indentation. Solenomere (sl) quite long.

########### Distribution and habitat.

Known only from the type locality and nearby area. It was found in limestone habitats. Given the narrow distribution in the small limestone area in the west of Thung Salaeng Luang National Park (Srisonchai et al. 2016), we regard this species as endemic for Thailand.

########### Remarks.

We collected additional topotypes during the rainy season. Some morphological variation was found. Thus, in some individuals, the sternal lobe between male coxae 4 is subrectangular, in others subtrapeziform. Gonopod variation is also found in this species; the tip of process (plm) of lamina medialis seems to be slightly blunt in some specimens, slightly sharp in others.

########### Coexisting species.

None known.

########### Corrections to Srisonchai et al. (2016)

Srisonchai et al. (2016, pp. 99–103) wrote in the description of this species that the paraterga (including paraterga of collum) are directed dorsolaterad at ca. 30°. They are in fact directed caudolaterad and elevated at ca. as 45°. Moreover, Srisonchai et al. also described the type of tubercles on metaterga as spines in body rings 2–19. We prefer to change terms, from spine to tubercle and cone – thus, metaterga 2–17 with rows of 2+2 anterior cones and 2+2 posterior spines, metaterga 18–19 with rows of 2+2 anterior tubercles and 2+2 posterior tubercles.

########## Desmoxytes planata

Taxon classificationAnimaliaPolydesmidaParadoxosomatidae

(Pocock, 1895)

Figs 5 , 6 , 7 , 8 , 67 , 68 , 69 , 70 , 71 , 72 , 73 , 74

Prionopeltis planatus Pocock, 1895: 829. Attems 1914: 204; 1936: 215; 1937: 114.
Pratinus planatus – Jeekel 1964: 63 (in key); 1968: 61. Mauriès 1980: 161.
Desmoxytes planata – Jeekel 1980a: 652; 1980b: 124. Golovatch and Korsós 1992: 25. Golovatch and Enghoff 1994: 60. Shelley and Lehtinen 1998: 88. Enghoff 2005: 96. Decker 2010: 29. Nguyen and Sierwald 2013: 1240. Zoysa et al. 2016: 474. Golovatch and Wesener 2016: 45. Likhitrakarn et al. 2017: 20.
Desmoxytes coniger Chamberlin, 1923: 165. Jeekel 1980a: 652 (synonymised).
Euphyodesmus greeni (Silv.) Attems, 1936: 213. Jeekel 1980a: 652 (synonymised).
Euphyodesmus greeni – Attems 1937: 128.
Pratinus greeni – Jeekel 1964: 63 (in key).
Euphyodesmus (Ceylonesmus) vector Chamberlin, 1941: 33. Jeekel 1980a: 652 (synonymised).
Pratinus vector – Jeekel 1964: 63 (in key).
Pratinus rastrituberus Zhang, 1986: 255. Golovatch and Enghoff 1994: 60 (synonymised).

########### Material examined.

**Lectotype.** Male (NHMUK, 1892.5.4.64–74), MYANMAR, Andaman Sea, Great Cocos Island, leg. E. W. Oates; designated by Mauriès (1980).

**Paralectotypes.** 1 male, 2 females pinned through body (>10 males and >15 females broken and mixed) (NHMUK), MYANMAR, Andaman Sea, Great Cocos Island, leg. E. W. Oates.

########### Other material examined.

**CHINA**: 8 males, 2 females (CUMZ), Yunnan, Xishuangbanna, Mengla, 213 national road near Menglunzhen, Munlun Village, 21°56'40"N, 101°13'45"E, ca. 576 m a.s.l., 26 November 2016, leg. S. Panha, C. Sutcharit and E. Jeratthitikul.

**FIJI**: 1 female, 1 juvenile (ZMUC), Viti Levu Suva, in garden, 2–3 September 1995, leg. A. Van Harten. 1 male (ZMUC), Upper Sigataka Valley, 6 March 1995, leg. A. Van Harten. 1 female (ZMUC), Colo-i-Suva Forest Park, 10 January 1995, leg. A. Van Harten. 3 females (ZMUC), Viti Levu, Colo-i-Suva Forest Park, 23–27 August 1995, leg. A. Van Harten. 1 male (ZMUC), Viti Levu, Colo-i-Suva Forest Park, 3 March 1995, leg. A. Van Harten. 4 males (ZMUC), Viti Levu, Colo-i-Suva Forest Park, 22 October 1995, leg. A. Van Harten. 1 male (ZMUC), Viti Levu, Colo-i-Suva Forest Park, 1995, leg. A. Van Harten. 1 male (ZMUC), Colo-i-Suva Forest Park, 29 March–6 April 1995, leg. A. Van Harten.

**MYANMAR**: 4 males (CUMZ), Tanintharyi Region, Myeik, 20 km northeast of Monoron, near Lenya National Park, Lampane Village, Ngawun Chaung River, 11°40'18"N, 99°13'29"E, ca. 44 m a.s.l., 9 June 2015, leg. ASRU members and FFI staff.

**SEYCHELLES**: 2 females (ZMUC), Mahé, ca. 50 m above Hotel Reef (near airport), under dead banana leaves, 8 October 1997, leg. H. Sturm.

**THAILAND**:

**Chiang Mai Province**: 1 female (ZMUC), Mae Rim District, Queen Sirikit Botanical Garden, June 1998. leg. R. Meier. 2 males, 6 females (ZMUC), Chiang Mai Province, Chiang Mai, in garden, ca. 300 m a.s.l., 26 September 1981, leg. ZMUC staff. 1 male, 1 female (ZMUC), Chiang Mai, in garden, ca. 300 m a.s.l., 2 October 1981, leg. ZMUC staff. 1 female (ZMUC), Doi Inthanon National Park, Siripum Waterfall, ca. 1200–1300 m a.s.l., 2 October 1981, leg. ZMUC staff. 1 female (ZMUC), Fang Horticultural Station, ca. 1200–1300 m a.s.l., 21 October 1981, leg. ZMUC staff. 2 females, 2 juveniles (CUMZ), Chiang Dao District, Wat Tham Chiang Dao, 19°23'46"N, 98°55'45"E, ca. 442 m a.s.l., 20 July 2008, leg. ASRU members. 1 female (CUMZ), Mueang Chiang Mai District, 700 years house of Chiang Mai, 2 October 2009, leg. S. Panha and ASRU members. 1 female (CUMZ), Mueang Chiang Mai District, 700 years house of Chiang Mai, 2 October 2009, leg. S. Panha and ASRU members. 1 female (CUMZ), Mae On District, Mae Kampong Waterfall, 18°51'56"N, 99°21'18"E, ca. 1069 m a.s.l., 27 September 2009, leg. S. Panha and ASRU members.

**Chiang Rai Province**: 7 males, 4 females (CUMZ), Mae Lao District, Dong Mada Subdistrict, Huai Kon Kom, beside road no.118, 19°44'55"N, 99°39'33"E, ca. 472 m a.s.l., 27 November 2009, leg. ASRU members.

**Chumphon Province**: 1 male, 1 female (CUMZ), Pathio District, Phitsadarn Cave (Tham Phitsadarn), 10°45'36"N, 99°13'46"E, ca. 106 m a.s.l., 29 August 2015, leg. ASRU members. 2 females (CUMZ), Pathio District, Phitsadarn Cave (Tham Phitsadarn), 10°45'36"N, 99°13'46"E, ca. 106 m a.s.l., 5 January 2017, leg. ASRU members.

**Kanchanaburi Province**: 14 males, 11 females, 2 juveniles (CUMZ), Thong Pha Phum District, Thong Pha Phum Forest Garden, 14°40'06"N, 98°35'42"E, ca. 171 m a.s.l., 8 May 2014, leg. ASRU members. 1 male, 5 females (CUMZ), Thong Pha Phum District, Thong Pha Phum Forest Garden, 14°40'06"N, 98°35'42"E, ca. 171 m a.s.l., 24 July 2016, leg. P. Pimvichai, T. Backeljau, P. Prasankok and N. Nantarat. 2 males, 2 females, (CUMZ), Thong Pha Phum District, Huai Ka Yeng Hot Spring, 14°38'57"N, 98°31'28"E, ca. 202 m a.s.l., 9 May 2014, leg. ASRU members. 1 male (CUMZ), Thong Pha Phum District, Wat Huay Charoen Srattha Tham, 14°39'27"N, 98°31'38"E, ca. 202 m a.s.l., 9 May 2014, leg. ASRU members. 2 males, 2 females (CUMZ), Sangkhla Buri District, Kroeng Krawia Waterfall, 14°58'55"N, 98°37'54"E, ca. 258 m a.s.l., 10 July 2009, leg. ASRU members. 2 females, 1 juvenile (CUMZ), Sri Sawat District, Erawan Waterfall National Park, 14°22'09"N, 99°08'41"E, ca. 102 m a.s.l., 28 August 2011, leg. ASRU members. 1 male, 1 broken fragment (CUMZ), Thong Pha Phum District, Tham Khao Noi Bureau of Monks, 14°41'55"N, 98°31'34"E, ca. 233 m a.s.l., 21 August 2015, leg. R. Srisonchai and C. Sutcharit. 3 females, 6 juvenile (CUMZ), Thong Pha Phum District, Kroeng Krawia Check Point, 14°56'32"N, 98°40'10"E, ca. 343 m a.s.l., 12 October 2015, leg. C. Sutcharit and ASRU members. 8 males, 9 females (CUMZ), Sangkhla Buri District, Wat Tham Su Kho, 15°02'14"N, 98°34'58"E, ca. 194 m a.s.l., 16 August 2016, leg. C. Sutcharit and ASRU members. 1 female (CUMZ), Sangkhla Buri District, Kra Teng Jeng Waterfall, 15°01'30"N, 98°36'03"E, ca. 202 m a.s.l., 16 August 2016, leg. C. Sutcharit and ASRU members. 7 males, 1 female (CUMZ), Sangkhla Buri District, Ban Song Karia, beside road no. 323, 15°13'01"N, 98°27'06"E, ca. 204 m a.s.l., 16 August 2016, leg. C. Sutcharit and ASRU members. 19 males, 26 females (CUMZ), Sangkhla Buri District, Tham Thep Prachao Praya Nak Kharach, 16 August 2016, leg. C. Sutcharit and ASRU members. 1 female (CUMZ), Sangkhla Buri District, Tham Kaeo Sawan Bandhan, 15°18'18"N, 98°24'57"E, ca. 339 m a.s.l., 16 August 2016, leg. C. Sutcharit and ASRU members. 2 females (CUMZ), Thong Pha Phum District, Wat Tha Kha-nun, 14°44'36"N, 98°38'19"E, ca. 113 m a.s.l., 16 August 2016, leg. C. Sutcharit and ASRU members. 4 males, 9 females (CUMZ), Thong Pha Phum District, Phuphrai Thannam Resort, 14°44'07"N, 98°38'39"E, ca. 110 m a.s.l., 16 August 2016, leg. C. Sutcharit and ASRU members. 3 males (CUMZ), Mueang Kanchanaburi District, Lat Ya Subdistrict, Klong Ta Ploen, 19 December 2016, leg. E. Jeratthitikul.

**Lampang Province**: 1 male, 5 male fragments, 5 females (CUMZ), Mae Mo District, Chao Por Pra Thu Pha Shrine, 18°30'48"N, 99°49'13"E, ca. 587 m a.s.l., 23 October 2008, leg. ASRU members. 1 female (CUMZ), Mae Mo District, Chao Por Pra Thu Pha Shrine, 18°30'48"N, 99°49'13"E, ca. 587 m a.s.l., 22 October 2015, leg. ASRU members.

**Mae Hong Son Province**: 12 males, 12 females (CUMZ), Mueang Mae Hong Son District, Tham Pla-Namtok Pha Suea National Park, 19°30'07"N, 98°00'23"E, ca. 398 m a.s.l., 19 July 2008, leg. ASRU members. 1 male, 5 females, 6 juveniles (CUMZ), Mae Sariang District, Mae Sariang Highway Division, 18°12'37"N, 97°56'15"E, ca. 296 m a.s.l., 16 January 2015, leg. ASRU members. 9 males, 14 females (CUMZ), Mueang Mae Hong Son District, Tham Phadaeng, 19°25'23"N, 97°59'04"E, ca. 288 m a.s.l., 19 September 2008, leg. ASRU members.

**Phayao Province**: 5 males, 6 females (CUMZ), Phusang District, Phu Sang Waterfall, 19°39'49"N, 100°22'36"E, ca. 472 m a.s.l., 24 October 2008, leg. ASRU members. 1 male (CUMZ), Phusang District, Phu Sang Waterfall, 19°39'49"N, 100°22'36"E, ca. 472 m a.s.l., 11 October 2014, leg. ASRU members. 1 broken male, 3 females (CUMZ), Phusang District, Phu Sang Waterfall, 19°39'49"N, 100°22'36"E, ca. 472 m a.s.l., 19 November 2012, leg. ASRU members. 1 male, 3 females (CUMZ), Chiang Kham District, Tham Phadaeng, 19°30'00"N, 100°27'10"E, ca. 495 m a.s.l., 24 October 2008, leg. ASRU members.

**Phetchaburi Province**: Mixed rings – probably 2 males (CUMZ), Kaeng Krachan District, Huai Mae Priang Subdistrict, Ban Dan Ngo, 12°48'41"N, 99°33'46"E, ca. 252 m a.s.l., 1 September 2007, leg. ASRU members. 1 male, 4 females, 1 juvenile (CUMZ), Khao Yoi District, Wat Paung Malai (Wat Tham Khao Iko), 13°18'42"N, 99°47'03"E, ca. 42 m a.s.l., 24 August 2016, leg. ASRU members. 17 males, 16 females (CUMZ), Khao Yoi District, Wat Paung Malai (Wat Tham Khao Iko), 13°18'42"N, 99°47'03"E, ca. 42 m a.s.l., 24 October 2016, leg. ASRU members.

**Phrae Province**: 3 males, 3 females (CUMZ), Rong Kwang District, Huai Rong Waterfall, 18°26'28"N, 100°26'60"E, ca. 441 m a.s.l., 31 August 2014, leg. ASRU members. 2 females (CUMZ), Den Chai District, Suan Sai Thong Restaurant, 17°58'17"N, 100°04'22"E, ca. 170 m a.s.l., 9 October 2008, leg. ASRU members. 1 male, 2 females (CUMZ), Den Chai District, Suan Sai Thong Restaurant, 17°58'17"N, 100°04'22"E, ca. 170 m a.s.l., 24 August 2014, leg. ASRU members. 31 males, 30 females (CUMZ), Den Chai District, Suan Sai Thong Restaurant, 17°58'17"N, 100°04'22"E, ca. 170 m a.s.l., 31 August 2014, leg. ASRU members.

**Prachuap Khiri Khan Province**: 1 male, 1 female, broken and mixed rings (CUMZ), Bang Saphan District, Wat Khao Tham Ma Rong, 11°12'05"N, 99°29'52"E, ca. 22 m a.s.l., 12 October 2008, leg. ASRU members. 8 males, 4 females (CUMZ), Kui Buri District, Hat Kham Subdistrict, near Kui Buri National Park, 12°03'13"N, 99°37'53"E, ca. 141 m a.s.l., 7 August 2014, leg. ASRU members. 7 males, 4 females (CUMZ), Kui Buri District, Hat Kham Subdistrict, near Kui Buri National Park, 12°03'13"N, 99°37'53"E, ca. 141 m a.s.l., 9 August 2016, leg. ASRU members. 4 males, 1 female (CUMZ), Kui Buri District, Hat Kham Subdistrict, near Kui Buri National Park, 12°03'13"N, 99°37'53"E, ca. 141 m a.s.l., 11 October 2010, leg. ASRU members. 9 males, 12 females (CUMZ), Mueang Prachuap Khiri Khan District, Khao Ta Mong Lai Forest Park, 11°50'49"N, 99°49'35"E, ca. 14 m a.s.l., 24 October 2016, leg. ASRU members.

**Ratchaburi Province**: 1 female (CUMZ), Mueang District, Khao Bin Cave, 13°35'37"N, 99°40'00"E, ca. 73 m a.s.l., 8 September 1973, leg. CUMZ staff.

**Sa Kaeo Province**: 2 males, 2 females (CUMZ), Mueang Sa Kaeo District, Tayak Subdistrict, near Pang Sida National Park, 13°58'26"N, 102°12'15"E, ca. 68 m a.s.l., 17 July 2016, leg. A. Pholyotha.

**Saraburi Province**: 1 male (CUMZ), Muak Lek District, Chet Sao Noi Waterfall, 14°44'07"N, 101°11'30"E, ca. 158 m a.s.l., 11 October 2014, leg. S. Panha, P. Tongkerd and A. Pholyotha.

**Tak Province**: 2 males, 3 females, 1 fragment of male (CUMZ), Phop Phra District, Pha Charoen Waterfall, 16°30'04"N, 98°45'06"E, ca. 649 m a.s.l., 5 July 2009, leg. ASRU members. 3 males, 2 females, 1 juvenile, broken and mixed fragments (CUMZ), Umphang District, Khun Danai Restaurant, 16°02'32"N, 98°50'55"E, ca. 466 m a.s.l., 6 July 2009, leg. ASRU members. 1 broken male (CUMZ), Mae Sot District, PK House Hotel, 16°43'39"N, 98°34'13"E, ca. 217 m a.s.l., 5 July 2009, leg. ASRU members. 2 males, 4 females, 6 juveniles (CUMZ), Phop Phra District, Um Piam Village, 16°24'33"N, 99°00'22"E, ca. 1088 m a.s.l., 1 July 2015, leg. ASRU members. 7 males, 1 female, 1 juvenile (CUMZ), Umphang District, Thi Lo Su Riverside Resort, 16°02'47"N, 98°51'09"E, ca. 470 m a.s.l., 1 July 2015, leg. ASRU members. 20 specimens (CUMZ), Phop Phra District, Chao Por Phawo Phop Phra Shrine, near Ban Ja Dee Kho, 16°34'10"N, 98°40'03"E, ca. 554 m a.s.l., 2 July 2015, leg. ASRU members. 1 male (CUMZ), Umphang District, Mae Klong Kee Bureau of Monks, 16°13'46"N, 98°55'12"E, ca. 586 m a.s.l., 30 June 2015, leg. ASRU members.

########### Diagnosis.

Body black or brownish black; collum with three transverse rows of setae and setiferous tubercles (4+4 anterior setae, 1+1 intermediate and 2+2 posterior tubercles); metaterga 2–16 with two rows of 2+2 (anterior) setiferous cones and 2+2 (posterior) setiferous spines; ventral ridge (vrl) of lamina lateralis conspicuous; process (plm) of lamina medialis long, directed almost mesad; distal lobe (dlm) distally with two distinct lamellae. Similar in these respects to *D. euros* sp. n., but differs from that species by having paraterga vivid pink and hypoproct subtrapeziform with inconspicuous setiferous tubercles.

########### Type locality.

Great Cocos Island, Andaman Sea [Myanmar, Yangon Region].

########### Redescription.

SIZE: Length 16–26 mm (male), 20–28 mm (female); width of midbody metazona ca. 1.8 mm (male), 2.1 mm (female). Width of head ≤ collum ≥ body ring 2 ≥ 3 = 4 < 5–16, thereafter body gradually tapering towards telson.

**Figure 67.**
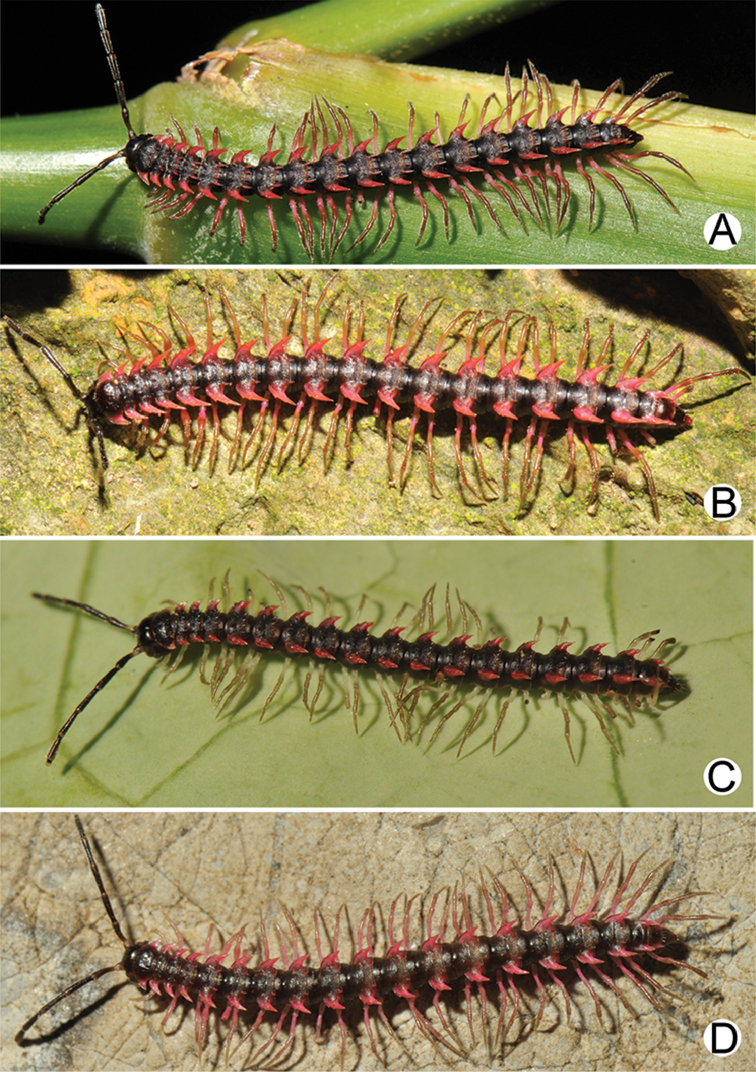
Photographs of live *Desmoxytes planata* (Pocock, 1895) – males. **A** specimen from Xishuangbanna, Mengla (China) **B** specimen from Suan Sai Thong Restaurant, Phrae (Thailand) **C** specimen from Lampane Village, Nguwun Chuang River (Myanmar) **D** specimen from Wat Khao Tham Ma Rong, Prachuap Khiri Khan (Thailand).

**Figure 68.**
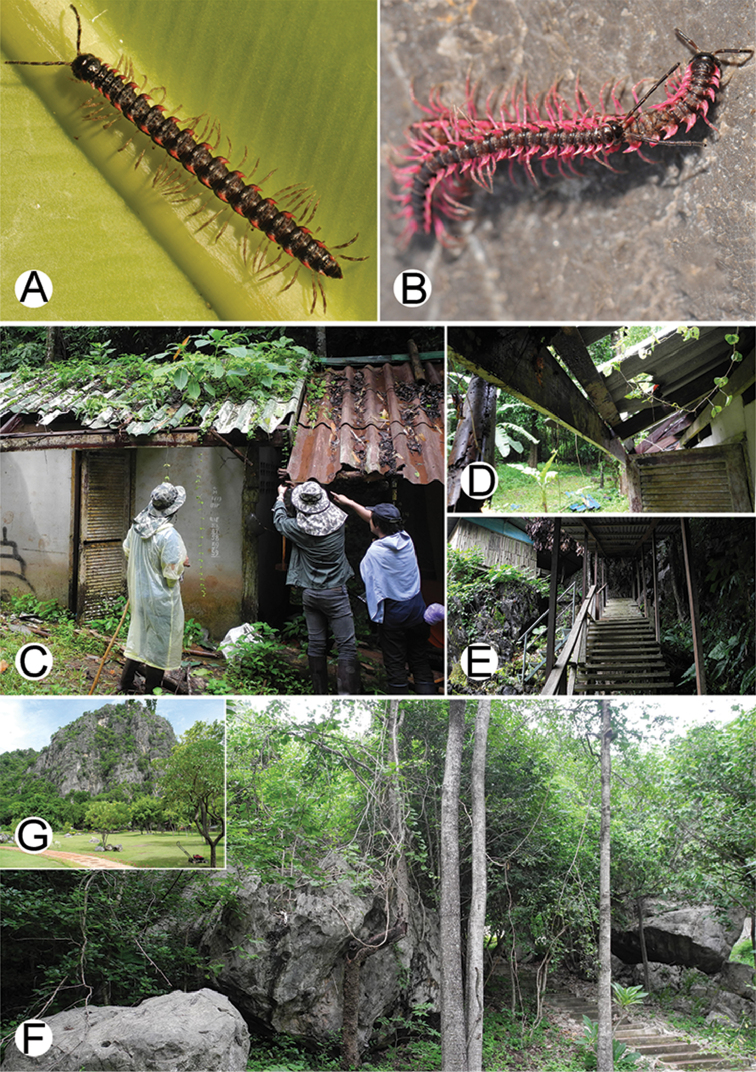
Photographs of live *Desmoxytes planata* (Pocock, 1895) and habitats. **A** female **B** mating couple **C–E** synanthropic areas **F, G** limestone area near synanthropic area.

COLOUR (Figs 67, 68A, B): In life with body black or brownish black; head, antenna (except distal part of antennomere 7 and antennomere 8 whitish), metaterga, and surface below paraterga brownish black; paraterga vivid pink; sterna pinkish brown; epiproct brown; legs brownish pink; a few basal podomeres pinkish white. Colour in alcohol: after 120 years changed to pale brown, after 2–10 years changed to brown or pale brown.

**Figure 69.**
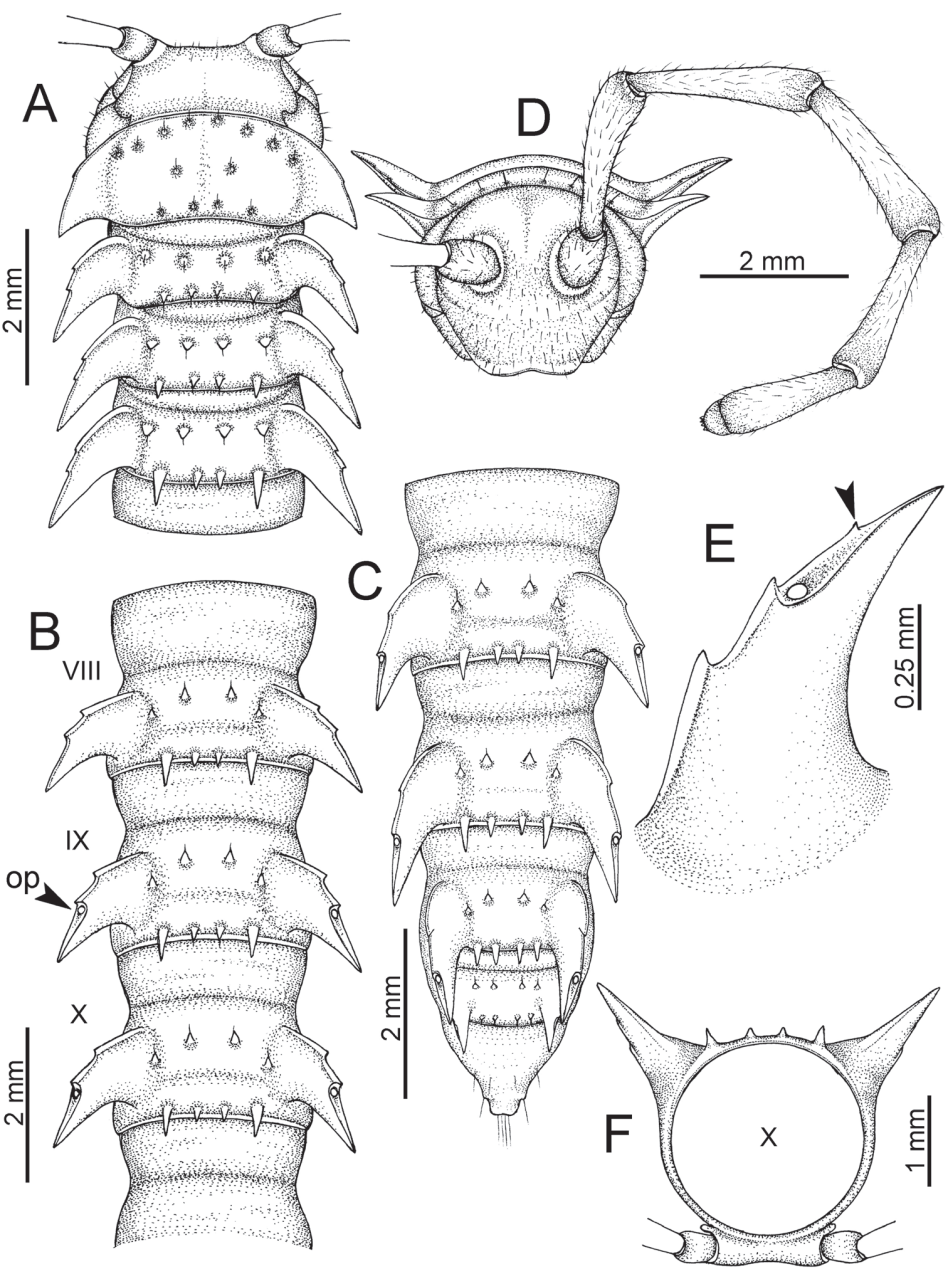
*Desmoxytes planata* (Pocock, 1895), specimen from Suan Sai Thong Restaurant. **A** anterior body part **B** body rings 8–10 (op = ozopore) **C** posteriormost body rings and telson **D** head and antenna **E** paraterga of ring 10 (arrow = tiny denticle) **F** body ring 10.

ANTENNAE (Fig. 69D): Moderately long and slender, reaching to body ring 5 or 6 (male) and 4 or 5 (female) when stretched dorsally.

COLLUM (Fig. 69A): With 3 transverse rows of setae and setiferous tubercles, 4+4 anterior setae, 1+1 intermediate and 2+2 posterior tubercles (lateral seta in anterior row located almost at base of paraterga in some specimens, posterior tubercles in posterior row bigger than others); paraterga of collum low, elevated at ca. 10°–15°, directed caudolaterad, with one inconspicuous notch on lateral margin.

TEGUMENT: Slightly shining; prozona finely shagreened; metaterga and surface below paraterga finely microgranulate; collum, sterna and epiproct smooth.

METATERGA (Fig. 69A–C): With 2 transverse rows of setiferous tubercles/cones/spines; metaterga 2–17 with 2+2 anterior tubercles/cones and 2+2 posterior cones/spines (lectotype and paralectotypes all with 2+2 anterior tubercles and 2+2 posterior tubercles/cones on metaterga 2–17); metaterga 18 and 19 with 2+2 anterior and 2+2 posterior tubercles.

PARATERGA (Fig. 69E, F): Directed caudolaterad on body rings 2–17, elevated at ca. 45° (male) 40° (female), in lectotype and paralectotypes less elevated than in others: at ca. 40° in male and 35° in female; directed increasingly caudad on body rings 18 and 19; anterior margin with 2 distinct notches, on lateral margin of body rings 9, 10, 12, 13, 15–18 with tiny denticle near the tip (tiny denticle of lectotype and paralectotypes poorly developed).

**Figure 70.**
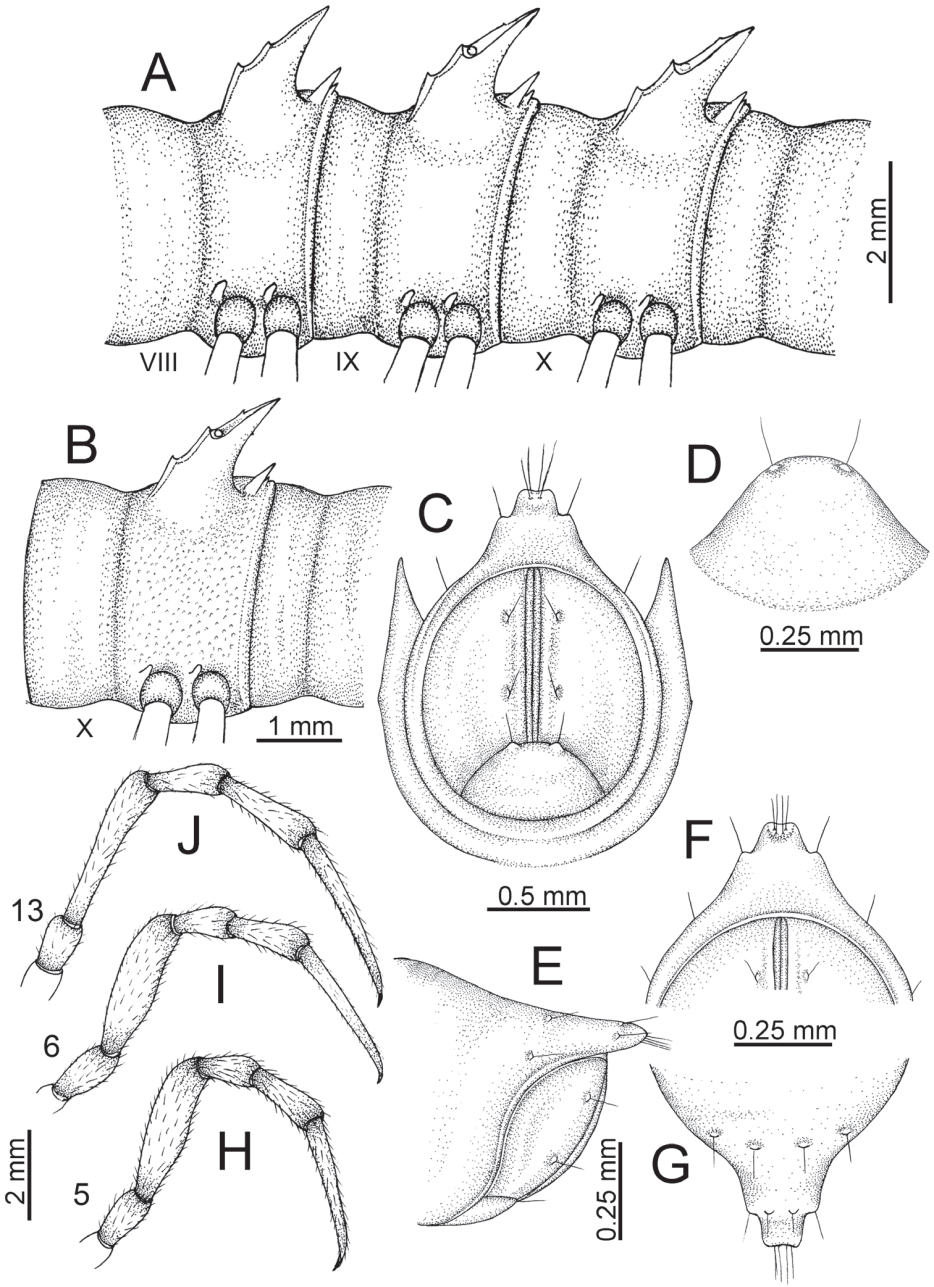
*Desmoxytes planata* (Pocock, 1895), specimen from Suan Sai Thong Restaurant. **A** body rings 8–10 **B** sculpture of ring 10 **C, E** last ring and telson **D** hypoproct **F, G** epiproct **H** male leg 5 (right) **I** male leg 6 (right) **J** male leg 13 (right).

TELSON (Fig. 70C–G): Epiproct: tip usually subtruncate (in some specimens slightly emarginate); lateral setiferous tubercles and apical tubercles usually conspicuous (in some specimens inconspicuous). Hypoproct subtrapeziform; caudal margin round, with inconspicuous setiferous tubercles.

**Figure 71.**
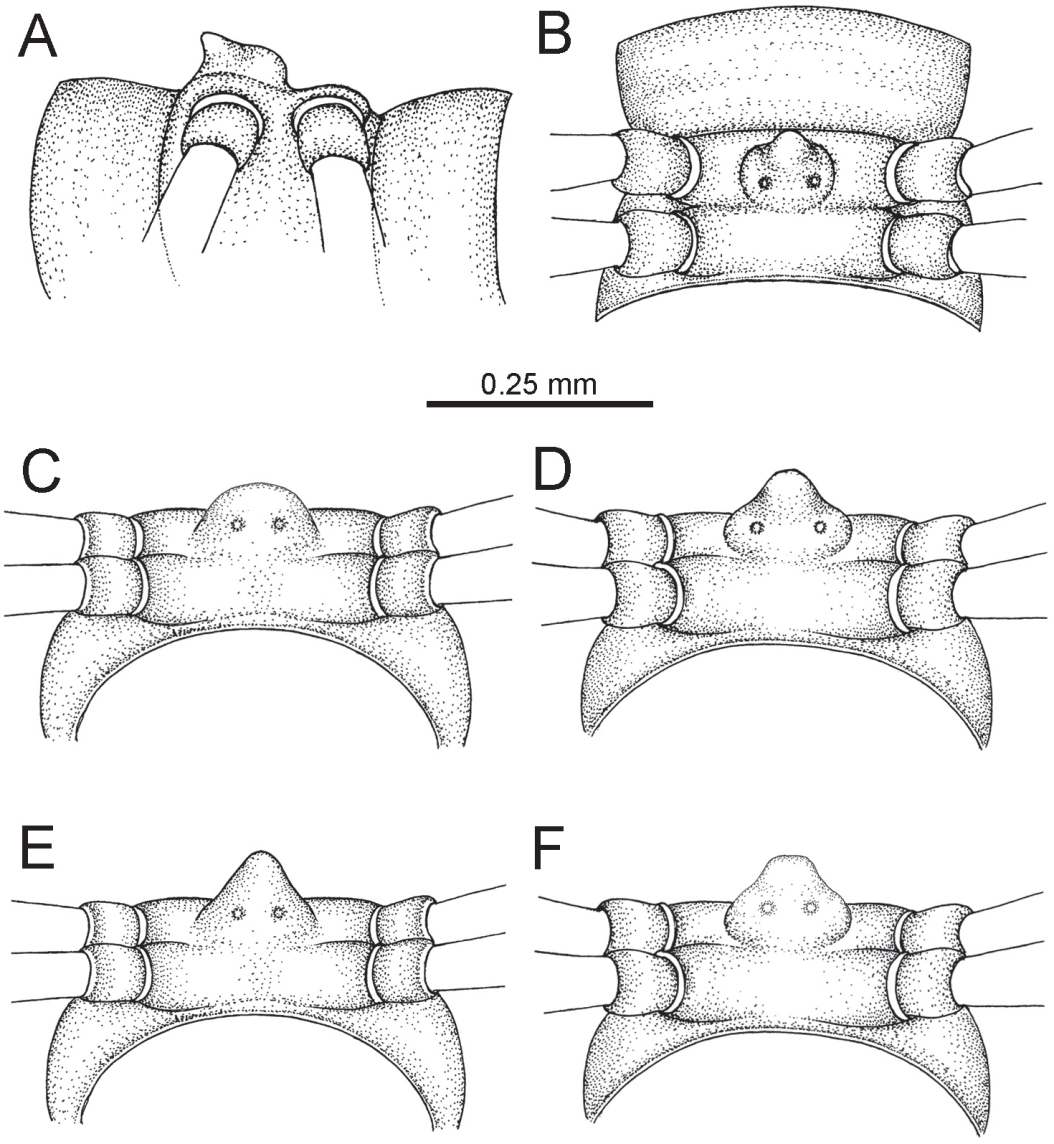
*Desmoxytes planata* (Pocock, 1895), population from Suan Sai Thong Restaurant – sternal lobe between male coxae 4. **A** lateral view **B** ventral view **C** caudal view (specimen from Fiji only) **D–F** caudal view.

STERNA (Fig. 71): Cross-impressions shallow. Sternal lobe between male coxae 4 swollen subtrapeziform/subsemicircular when seen in caudal view (round in lectotype and paralectotypes); base stout; slightly attenuated near tip; tip usually subtruncate (in some specimens round).

LEGS (Fig. 70H–J): Long and slender. Male femora 5 and 6 slightly humped ventrally in middle portion.

**Figure 72.**
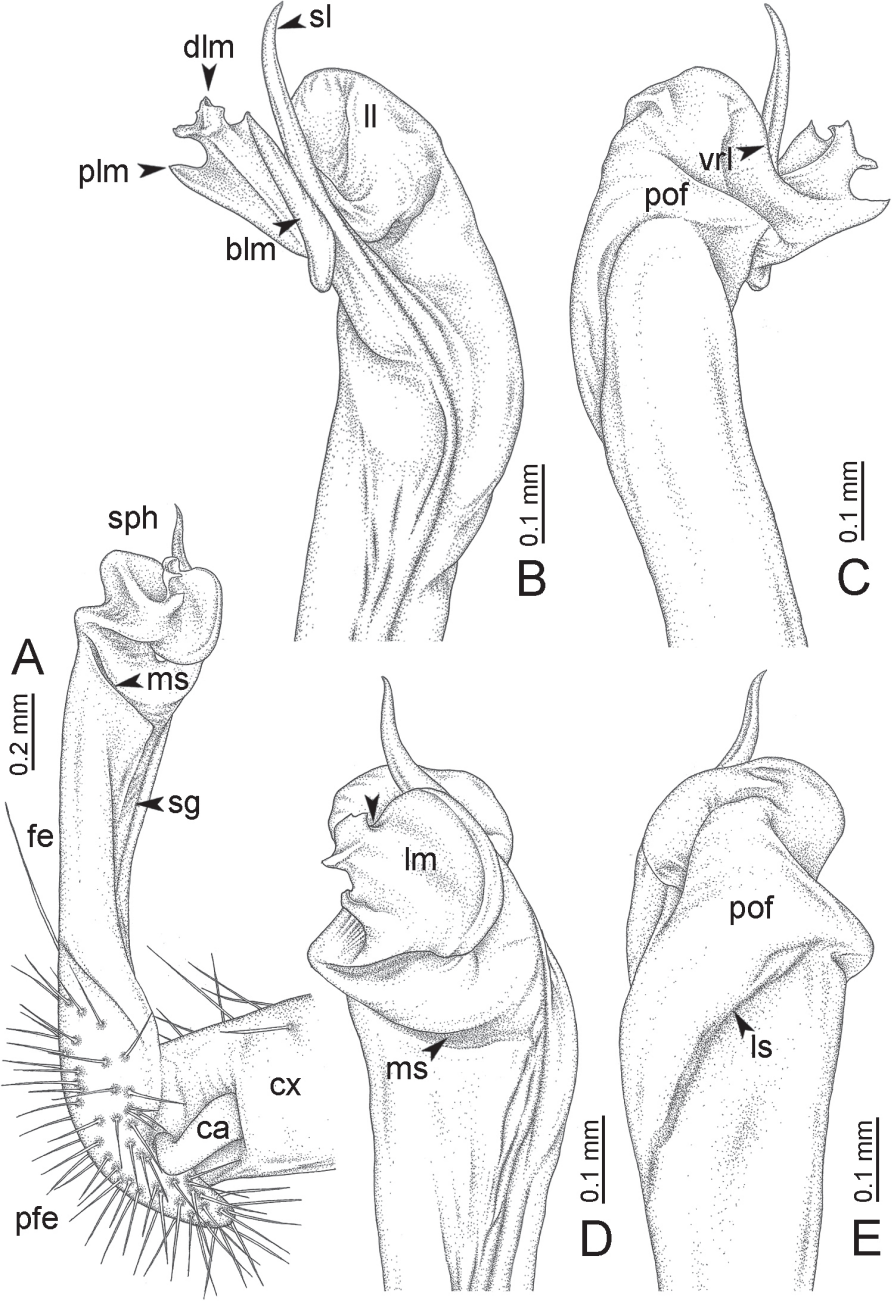
*Desmoxytes planata* (Pocock, 1895), specimen from Wat Puang Malai – right gonopod. **A** mesal view **B** dorsal view **C** ventral view **D** submesal view (arrow = indentation) **E** lateral view.

**Figure 73.**
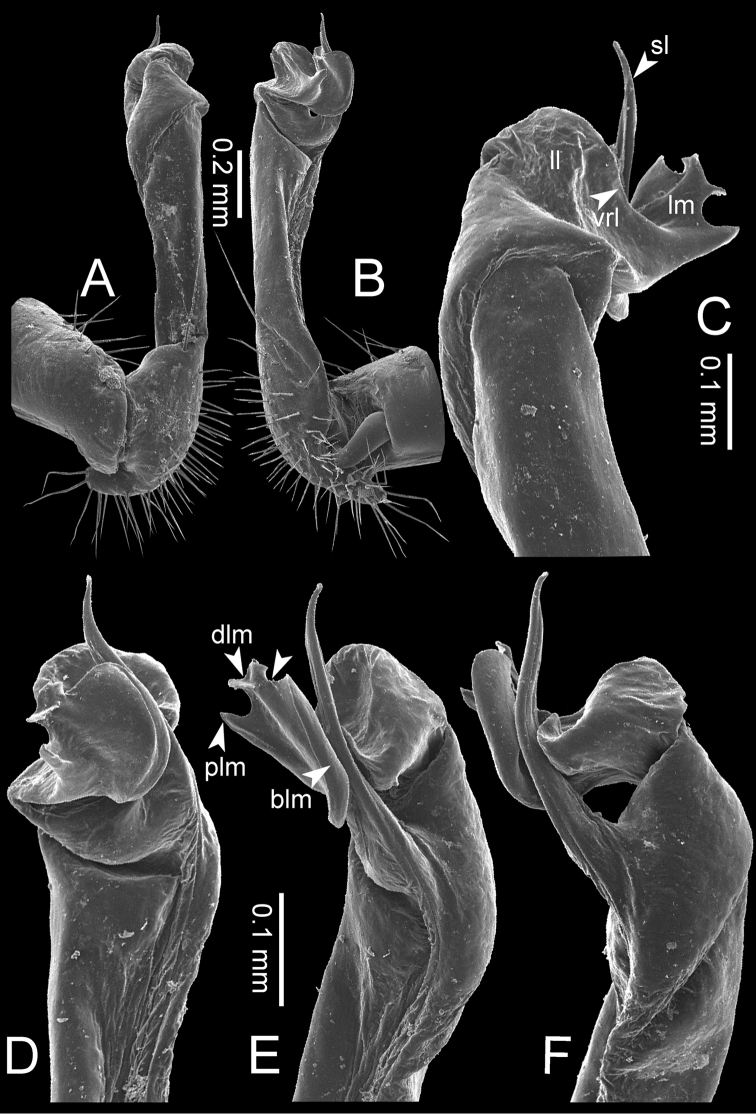
*Desmoxytes planata* (Pocock, 1895), specimen from Wat Puang Malai – right gonopod. **A** lateral view **B** mesal view **C** ventral view **D** subdorsal view **E** dorsal view (unlabeled arrow = indentation) **F** subdorsal view.

**Figure 74.**
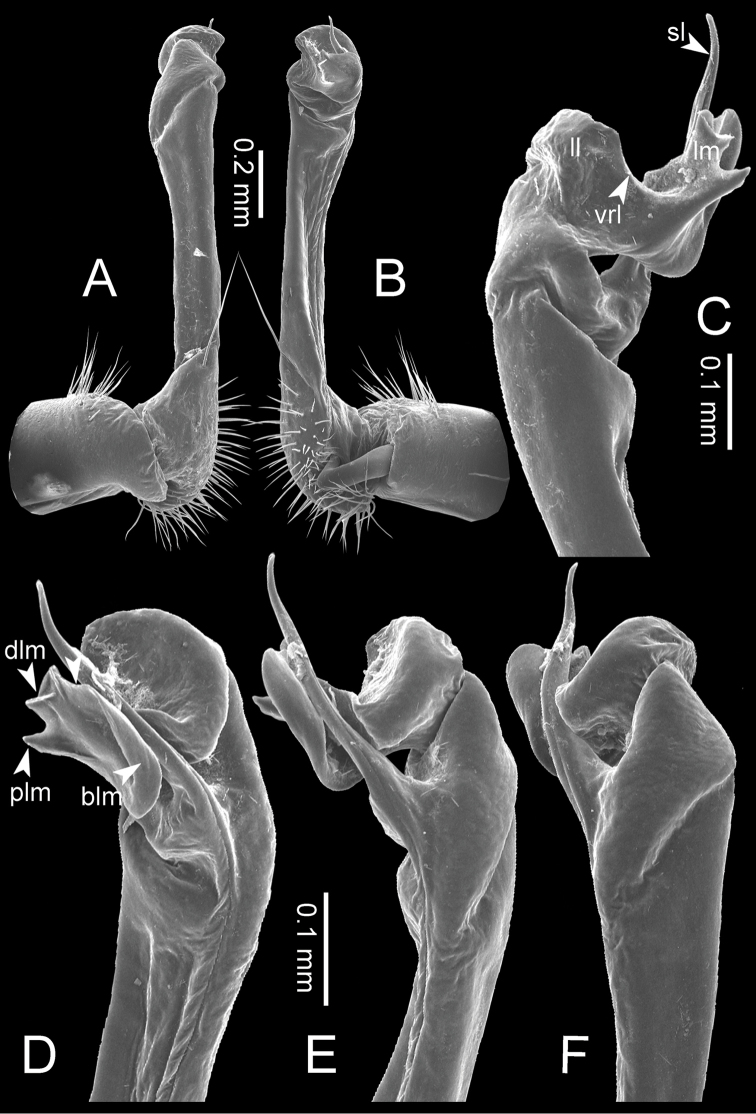
*Desmoxytes planata* (Pocock, 1895), specimen from Fiji, Viti Levu – right gonopod. **A** lateral view **B** mesal view **C** ventral view **D** subdorsal view (arrow = indentation) **E** dorsal view **F** subdorsal view.

GONOPODS (Figs 7, 8, 72, 73, 74): Coxa (cx) longer than prefemur. Cannula (ca) long and slender. Prefemur (pfe) ca. 2/3 as long as femur. Femur (fe) long and slender. Mesal sulcus (ms) and lateral sulcus (ls) conspicuous, very deep. Postfemur (pof) conspicuous, ventrally narrow, and short. Solenophore (sph) well-developed: lamina lateralis (ll) swollen, anterolaterally with an inconspicuous furrow; with conspicuous ventral ridge (vrl): lamina medialis (lm) well-developed; process (plm) long, spear-like, tip sharp (blunt in some specimens), directed mesad; distal lobe (dlm) distally with two distinct lamellae (mesal lamella smaller than second one); broad lobe (blm) dorsally thick at the edge, distinctly demarcated from distal lobe (dlm) by a conspicuous indentation. Solenomere (sl) very long.

########### Distribution and habitat.

This species was collected at several places together with *D. octoconigera* sp. n., *D. golovatchi* sp. n. and *D. purpurosea*, but these species apparently occupy different microhabitats. *D. planata* can be found all year round in humid places in environments strongly influenced by humans. It was very easy to find, mostly in plant farms or shaded gardens. Although *D. planata* was sometimes seen in areas close to another *Desmoxytes* species, the habitats where it was collected are clearly different: *D. planata* is often found in human-influenced habitats while the other ones live in natural habitats.

However, some specimens from Tham Khoa Ma Rong, Khoa Ta Mong Lai, Ban Yang Chum (all in Prachuap Khiri Khan Province) seem to be indigenous as they were found in limestone habitats, although not too far from human-influenced habitats. Certain introduced paradoxosomatid species seem to have penetrated natural habitats and have become more dominant and abundant than the native ones (Jeekel 1980b), and *D. planata* at the above-mentioned locations may similarly have dispersed into the forest.

This species has been reported from Hawaii (Chamberlin 1923, 1941). Because the only reliable records concern specimens taken from quarantine in Honolulu, it seems reasonable to delete *D. planata* from the Hawaii fauna. We believe that this species is transported easily by global commerce, most probably in soil-containing or plant-associated materials.

The origin of *D. planata* was assumed to be Burma or Malaya by Jeekel (1980a). Later, Shelley and Lehtinen (1998) regarded it more probable that this species occurs naturally in Thailand and China. Based on all recent data analysed by us, *D. planata* may originally be native to Thailand or Myanmar. Surprisingly, however, no specimen of *D. planata* has yet been found in Cambodia, Laos, or Malaysia, possibly owing to insufficient surveys in these areas.

Decker (2010) reported *D. planata* from eastern Thailand, viz., Namtok Phliu (Chantaburi Province), Khao Chamoa National Park (Rayong Province) and Ko Chang National Park (Trat Province). However, these localities all lie in the distribution range of the very similar *D. euros* sp. n., and Decker’s specimens may well belong to the latter species.

########### Note on type material.

The lectotype was designated by Mauriès (1980). In the jar which contains lectotype and paralectotypes in NHMUK, there are three small vials:

– one vial contains the male lectotype with a label “*Pratinus planatus* (Poc.) male lectotype vid. Mauriès (Paris)”, pinned through the body; collum and telson were broken off.

– a second vial with one male and two female paralectotypes (one female with only rings 10–20), all specimens pinned through the body.

– a third vial with broken specimens (>5 specimens).

There is also one more vial containing many broken and mixed specimens (>15 specimens).

########### Remarks.

The vivid pink paraterga are obviously aposematic.

According to many previous works, as well as our own results, this species seems to be almost pantropical. In order to assess morphological variability, we compared the gonopods of several specimens (including illustrations) reported from different localities: *D. planata* from Myanmar (lectotype and paralectotypes); *D. coniger* – (Jeekel 1980b) from Hawaii taken from Java (Bogor); *D. planata* – (Mauries 1980) from Seychelles; *D. planata* – (Shelley and Lehtinen 1998) and (Golovatch and Enghoff 1994) from Fiji; *Pratinus rastrituberus* – (Zhang, 1986) from China. Combining the examination of previous works and the newly collected specimens from China, Myanmar, and Thailand, variation in morphological characters was as follows.

**I. Variation within populations** (Fig. 71)

– anterior row of tubercles on collum (usually with 4+4 anterior setae): in some specimens lateral setae located in anterior margin (conspicuous tubercles), in others lateral setae located almost at base of paraterga (inconspicuous tubercles).

– type of metatergal projections (anterior row and posterior row) on body rings 2–17: anterior tubercles/cones in some specimens, posterior cones/spines in others.

– tip of sternal lobe between male coxae 4: truncate in some specimens, quite round in others.

– process (plm) of lamina medialis: short in some individuals, long in others.

– tip of process (plm) of lamina medialis of specimens from Great Cocos Island: slightly emarginate in some, sharp in others.

– epiproct tip: in some specimens subtruncate, in others slightly emarginate.

**II. Variation between populations**

– Fiji population: posterior tubercles on collum seem to be bigger than in other populations.

– Fiji population: sternal lobe between male coxae 4 more round than in others.

– Great Cocos Island population: size seems to be smaller than in others (16–20 mm in male, 20-23 mm in female).

– Great Cocos Island population: metatergal tubercles shorter than in others.

On this basis, we strongly agree with Jeekel (1980a) and Golovatch and Enghoff (1994) with the synonymy of *D. coniger, E. greeni, E.vector* and *P. rastrituberus* under *Desmoxytes planata*. Although the type material of *D. coniger* in MCZ has not been examined by us, its identity with *D. planata* is clear from the photo and remarks given by Jeekel (1980a). The morphological characters of this specimen match perfectly with the others.

*Desmoxytes planata* is morphologically similar to *D. euros* sp. n. with which it shares the metaterga with 2+2 anterior tubercles/cones and 2+2 posterior cones/spines. Moreover, gonopod characters of these species are very similar in shape and processes. Based on morphological characters only, they could be supposed to be the same species. However, the colour of living specimens (paraterga) and shape of hypoproct are totally different, as well as ongoing COI analysis supports to separate them as different species.

########### Coexisting species.

*D. golovatchi* sp. n. and *D. octoconigera* sp. n. (see details under these species).

########## Desmoxytes purpurosea

Taxon classificationAnimaliaPolydesmidaParadoxosomatidae

Enghoff, Sutcharit & Panha, 2007

**Figure 75.**
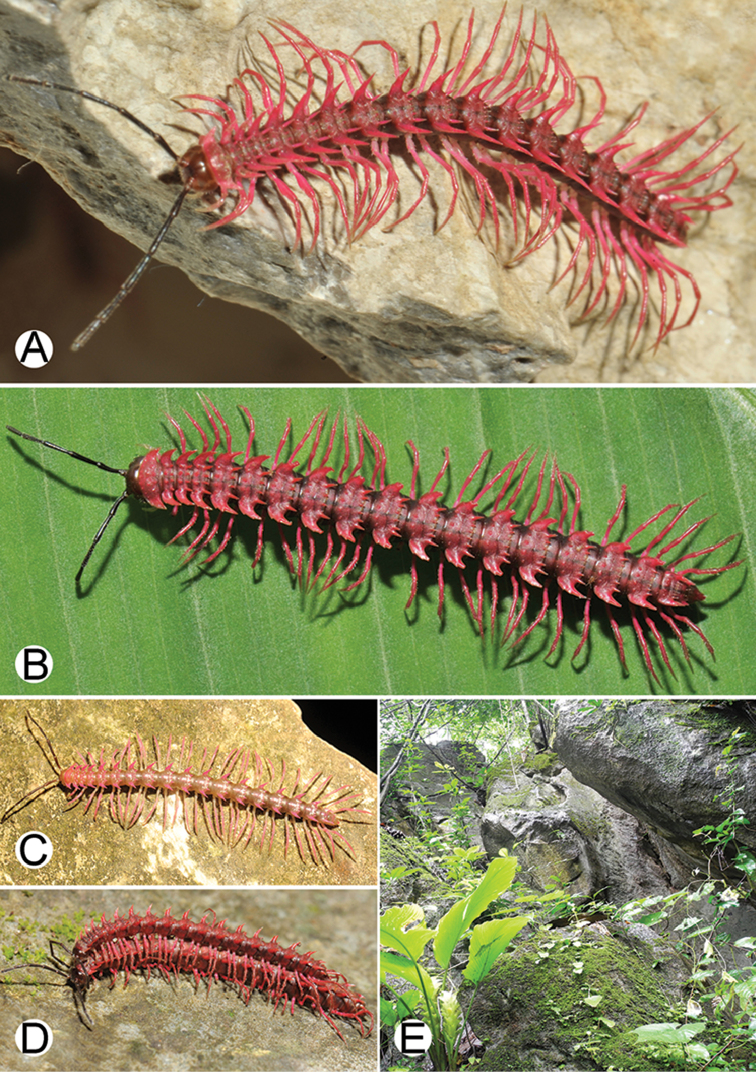
Photographs of live *Desmoxytes purpurosea* Enghoff et al., 2007 (specimens from Hup Pa Tard) and habitat. **A** male **B** female **C** early adult male **D** mating couple **E** habitat.

**Figure 76.**
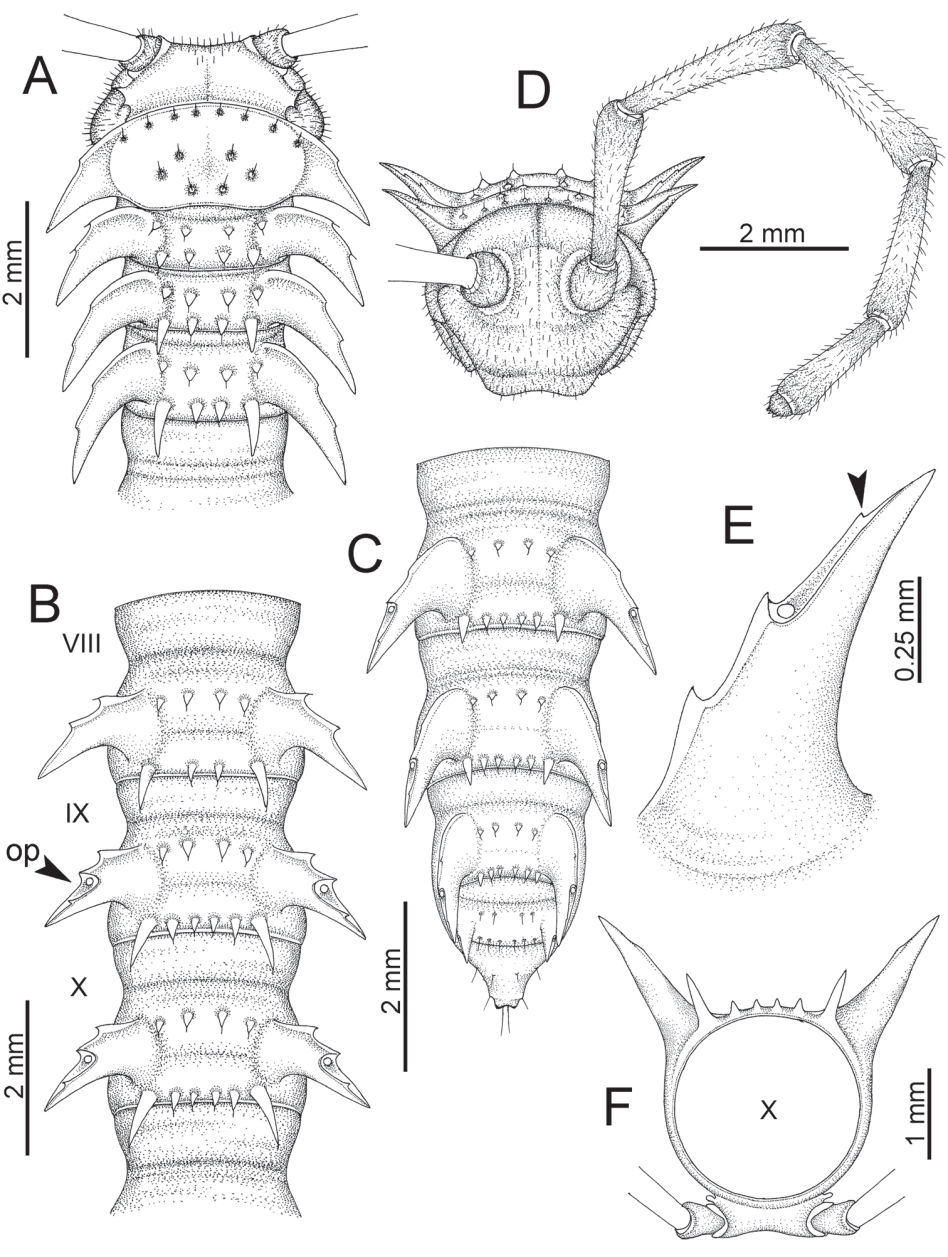
*Desmoxytes purpurosea* Enghoff et al., 2007 (male paratype). **A** anterior body part **B** body rings 8–10 (op = ozopore) **C** posteriormost body rings and telson **D** head and antenna **E** paraterga of ring 10 (arrow = tiny denticle) **F** body ring 10.

**Figure 77.**
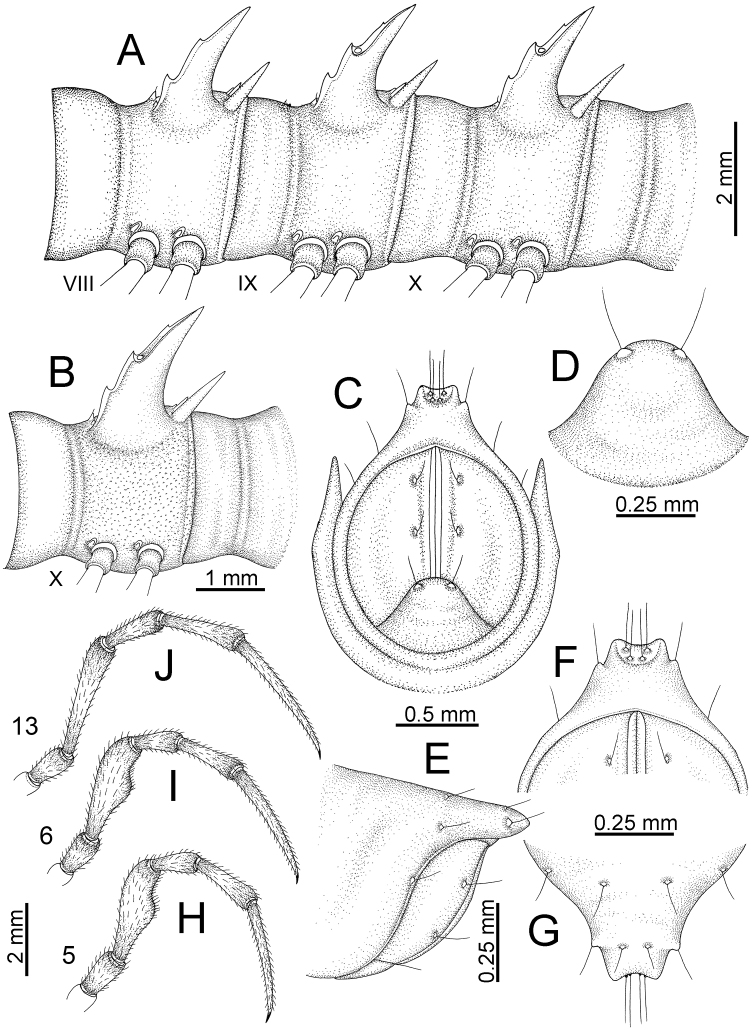
*Desmoxytes purpurosea* Enghoff et al., 2007 (male paratype). **A** body rings 8–10 **B** sculpture of ring 10 **C, E** last ring and telson **D** hypoproct **F, G** epiproct **H** male leg 5 (right) **I** male leg 6 (right) **J** male leg 13 (right).

**Figure 78.**
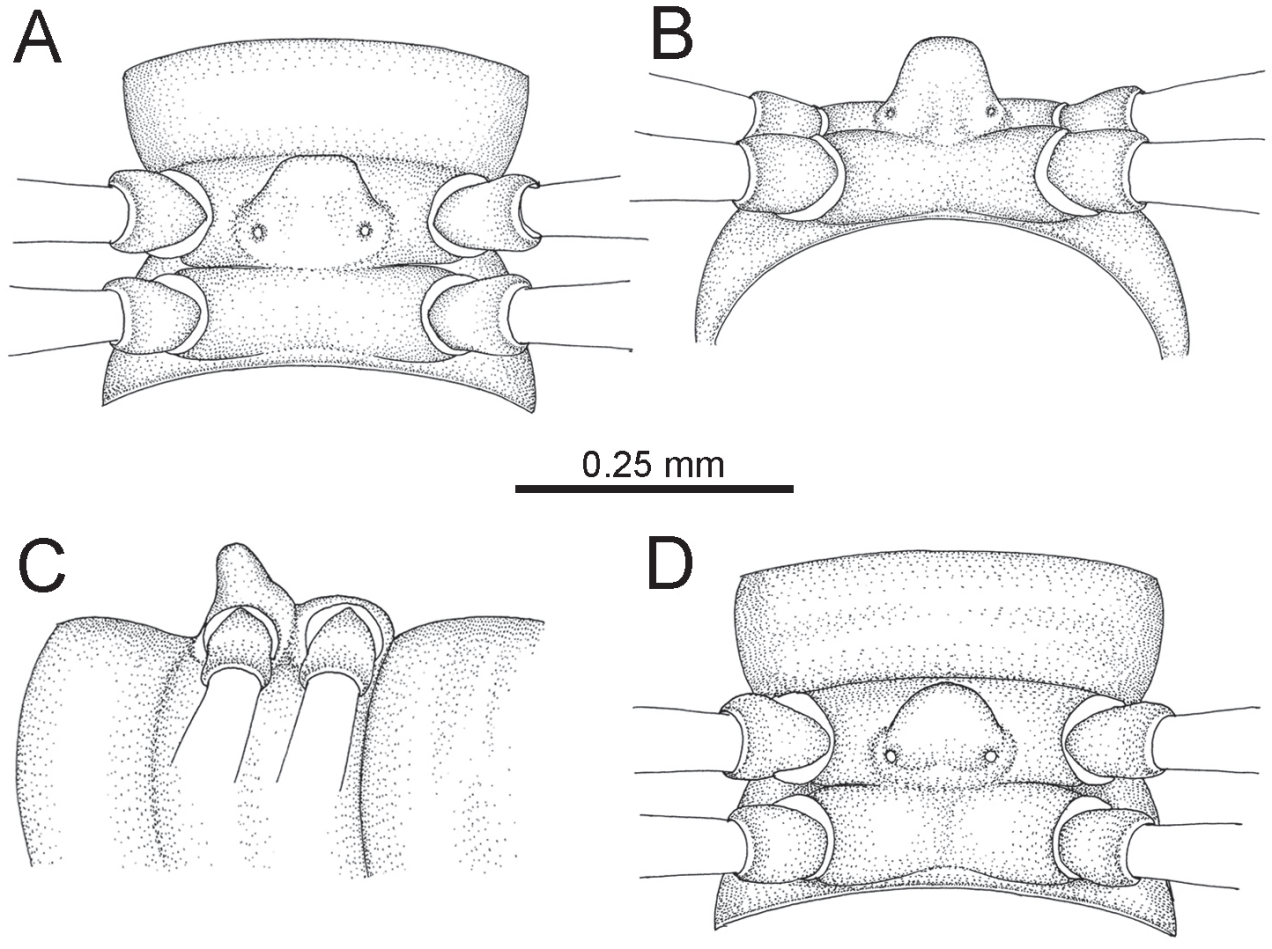
*Desmoxytes purpurosea* Enghoff et al., 2007 (male paratypes) – sternal lobe between male coxae 4. **A, D** ventral view **B** caudal view **C** lateral view.

**Figure 79.**
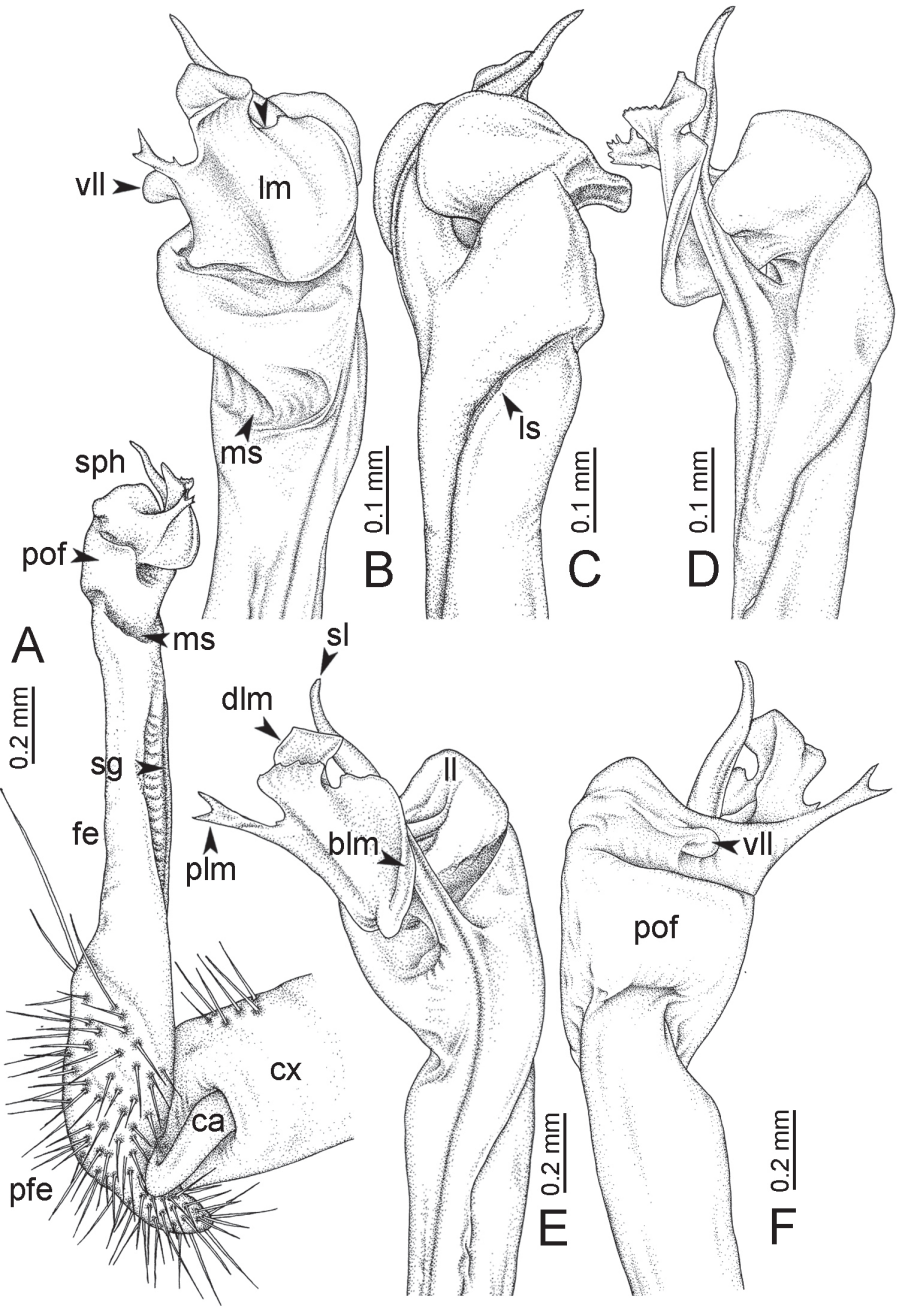
*Desmoxytes purpurosea* Enghoff et al., 2007 (specimen from Hup Pa Tard) – right gonopod. **A** mesal view **B** submesal view (arrow = indentation) **C** lateral view **D** dorsal view (Kanchababuri A and B populations) **E** subdorsal view **F** ventral view.

**Figure 80.**
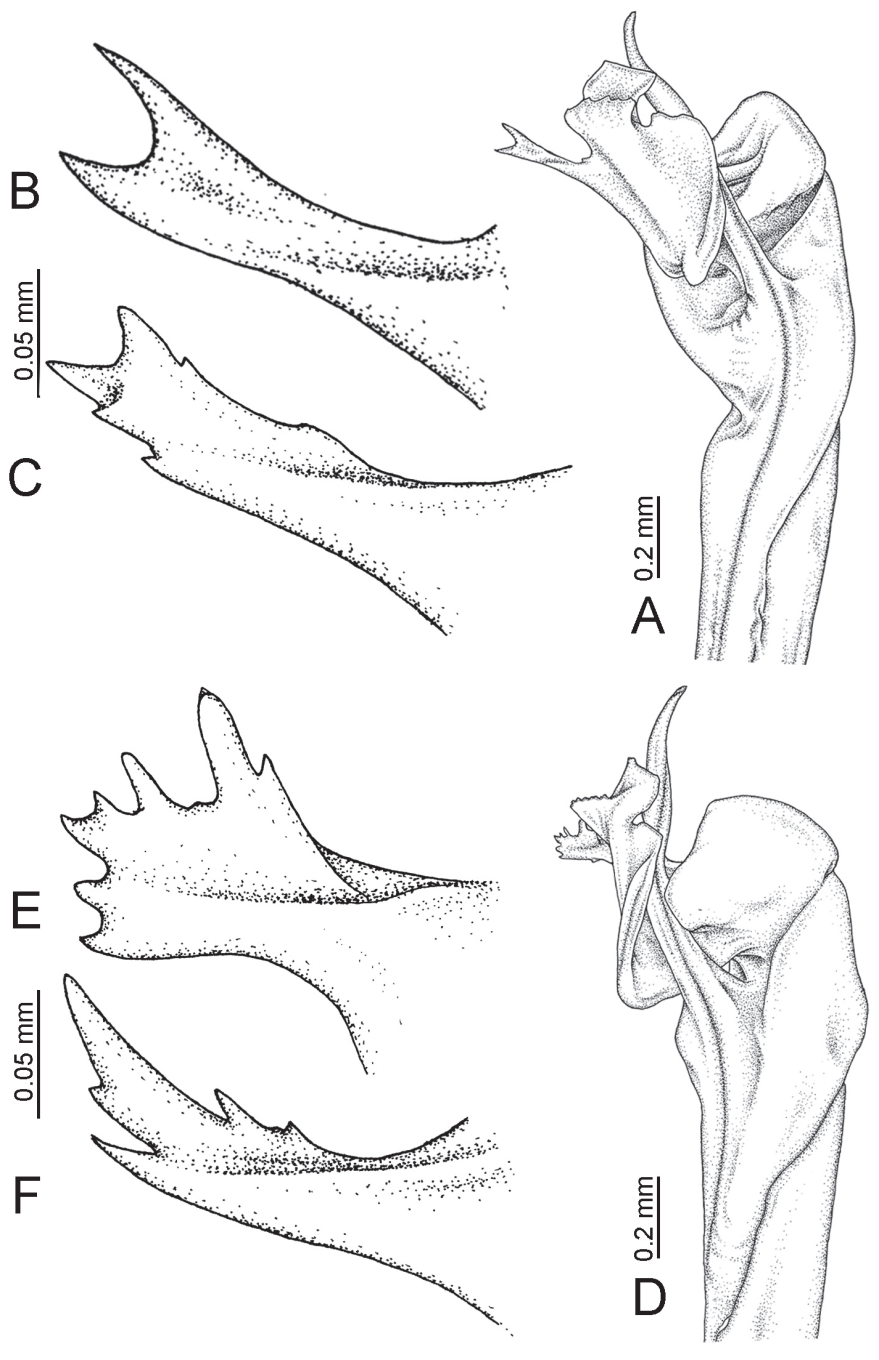
*Desmoxytes purpurosea* Enghoff et al., 2007 – right gonopod. **A** mesal view (specimen from Hup Pa Tard) **B, C** process (plm) of lamina medialis (Lamphun and Uthai Thani populations) **D** dorsal view (specimen from Tham Than Lod cave) **E, F** process (plm) of lamina medialis (Kanchababuri A and B populations).

**Figure 81.**
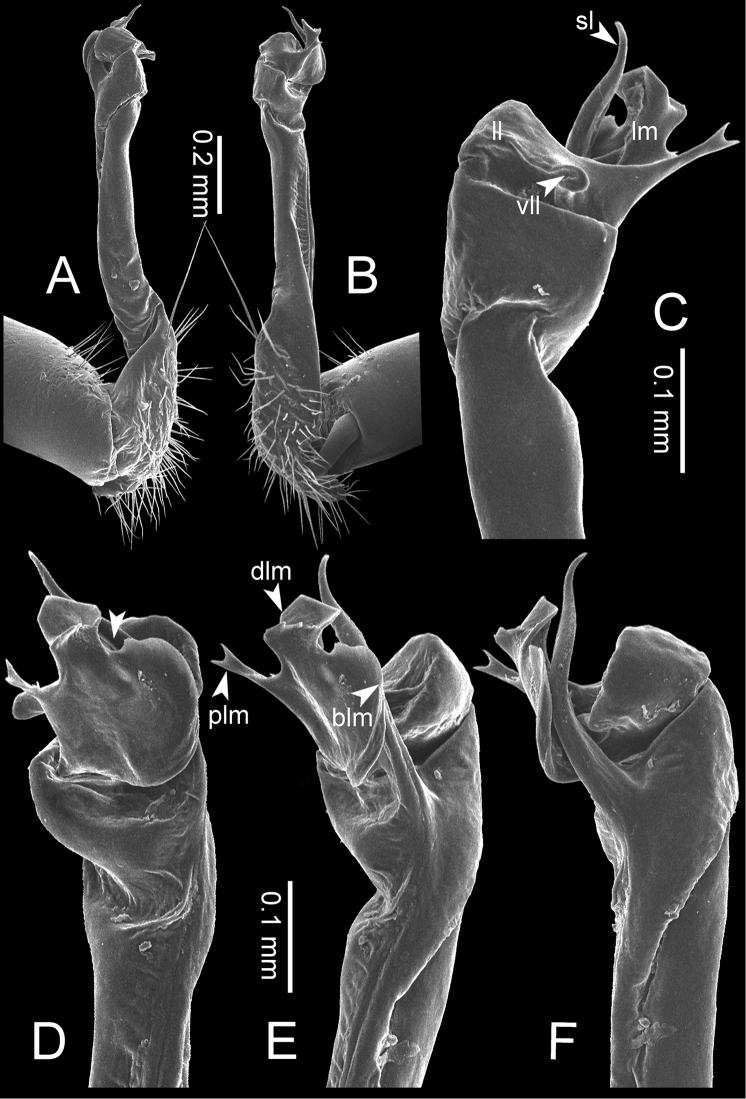
*Desmoxytes purpurosea* Enghoff et al., 2007 (specimen from Hup Pa Tard) – right gonopod. **A** lateral view **B** mesal view **C** ventral view **D** subdorsal view (arrow = indentation) **E** dorsal view **F** subdorsal view.

Figs 75 , 76 , 77 , 78 , 79 , 80 , 81

Desmoxytes purpurosea Enghoff, Sutcharit & Panha, 2007: 32. Nguyen and Sierwald 2013: 1242.

########### Material examined.

**Hototype**: Male (CUMZ), THAILAND, Uthai Thani Province, Department of National Parks, Tam Pratun Non-Hunting Area, Hup Pa Tard. 15°22'38"N, 99°37'50"E, ca. 113 m a.s.l., 28 August 2006, leg. S. Panha, H. Enghoff, P. Pimwichai, and C. Sutcharit.

########### Paratypes.

16 males, 30 females (CUMZ), 4 males, 3 females (ZMUC), same data as holotype.

########### Further specimens, 

**all from THAILAND, Kanchanaburi Province**: 8 males, 5 females, 1 broken male, 1 male missing gonopods, 1 male missing left gonopod, 1 fragment, 1 juvenile (CUMZ), Sai Yok District, Daowadueng Cave, 14°28'23"N, 98°50'04"E, ca. 132 m a.s.l., 11 July 2009, leg. S. Panha and ASRU members. 1 male (CUMZ), Sai Yok District, Daowadueng Cave, 14°28'23"N, 98°50'04"E, ca. 132 m a.s.l., 12 October 2015, leg. C. Sutcharit and R. Srisonchai. 14 males, 7 females (CUMZ), Sai Yok District, Daowadueng Cave, 14°28'23"N, 98°50'04"E, ca. 132 m a.s.l., 15 August 2016, leg. C. Sutcharit, R. Srisonchai, and ASRU members. 3 males, 1 female (CUMZ), Sai Yok District, Ban Thung Kang Yang, 14°24'17"N, 98°55'04"E, ca. 264 m a.s.l., 15 August 2016, leg. C. Sutcharit, R. Srisonchai, and ASRU members. 38 males, 13 females (CUMZ), Sai Yok District, Wat Sunantha Wanaram, 14°32'11"N, 98°49'51"E, ca. 161 m a.s.l., 17 August 2016, leg. C. Sutcharit, R. Srisonchai, and ASRU members. 1 male (CUMZ), Si Sawat District, Chalermrattanakosin National Park, Tham Than Lod Cave, 14°40'06"N, 99°19'02"E, ca. 255 m a.s.l., 9 September 1973, leg. CUMZ staff. Many specimens (CUMZ), Si Sawat District, Chalermrattanakosin National Park, Tham Than Lod Cave, 14°40'06"N, 99°19'02"E, ca. 255 m a.s.l., 10 July 2006, leg. CUMZ staff. 6 males, 4 females (CUMZ), Si Sawat District, Chalermrattanakosin National Park, Tham Than Lod Cave, 14°40'06"N, 99°19'02"E, ca. 255 m a.s.l., 13 October 2015, leg. C. Sutcharit and R. Srisonchai. 1 male missing gonopods, 2 females (CUMZ), Sangkhla Buri District, Kroeng Krawia Waterfall, 14°58'56"N, 98°37'55"E, ca. 264 m a.s.l., 10 July 2009, leg. S. Panha and ASRU members. 7 males, 12 females (CUMZ), Thong Pha Phum District, Kroeng Krawia Checkpoint, 14°56'32"N, 98°40'11"E, ca. 347 m a.s.l., 16 August 2016, leg. C. Sutcharit, R. Srisonchai, and ASRU members.

**Lamphun Province**: 1 male (CUMZ), Pasang District, Tham Erawan (Erawan Cave), 18°19'35"N, 98°52'24"E, ca. 551 m a.s.l., 26 October 2015, leg. C. Sutcharit, R. Srisonchai, and ASRU members.

**Suphan Buri Province**: 3 juveniles (CUMZ), Dan Chang District, Tham Weruwan, 14°57'17"N, 99°38'49"E, ca. 121 m a.s.l., 5 June 2017, leg. C. Sutcharit, A. Pholyotha, and ASRU members.

**Uthai Thani Province**: 1 male missing gonopods (CUMZ), Lansak District, Hup Pa Tard, 15°22'26"N, 99°37'58"E, ca. 113 m a.s.l., 8 June 2008, leg. S. Panha and ASRU members. 7 males, 9 females, 1 broken female, 1 male missing gonopods (CUMZ), Lansak District, Hup Pa Tard, 15°22'26"N, 99°37'58"E, ca. 113 m a.s.l., 15 May 2009, leg. S. Panha and ASRU members. 5 females (CUMZ), Lansak District, Hup Pa Tard, 15°22'26"N, 99°37'58"E, ca. 113 m a.s.l., April 2009, leg. S. Panha and ASRU members. 1 male, 3 broken and mixed males (CUMZ), Lansak District, Hup Pa Tard, 15°22'26"N, 99°37'58"E, ca. 113 m a.s.l., 27 October 2013, leg. S. Panha and ASRU members. Many specimens (CUMZ), Lansak District, Hup Pa Tard, 15°22'26"N, 99°37'58"E, ca. 113 m a.s.l., 31 May 2009, leg. S. Panha and ASRU members. Many specimens (CUMZ), Lansak District, Hup Pa Tard, 15°22'26"N, 99°37'58"E, ca. 113 m a.s.l., 7 June 2008, leg. S. Panha and ASRU members. Many specimens (CUMZ), Lansak District, Hup Pa Tard, 15°22'26"N, 99°37'58"E, ca. 113 m a.s.l., 10 September 2006, leg. S. Panha and ASRU members. Many specimens (CUMZ), Lansak District, Hup Pa Tard, 15°22'26"N, 99°37'58"E, ca. 113 m a.s.l., unknown date, unknown collector. 1 female (CUMZ), Lansak District, Hup Pa Tard, 15°22'26"N, 99°37'58"E, ca. 113 m a.s.l., 27 October 2013, leg. S. Panha and ASRU members. 2 males, 1 female (CUMZ), Lansak District, Hup Pa Tard, 15°22'26"N, 99°37'58"E, ca. 113 m a.s.l., 1 August 2014, leg. S. Panha and ASRU members. 34 specimens (CUMZ), Lansak District, Hup Pa Tard, 15°22'26"N, 99°37'58"E, ca. 113 m a.s.l., 20 October 2015, leg. C. Sutcharit and R. Srisonchai. 4 males, 1 female (CUMZ), Lansak District, Hup Pa Tard, 15°22'26"N, 99°37'58"E, ca. 113 m a.s.l., 27 July 2006, leg. C. Sutcharit, R. Srisonchai, and ASRU members. 1 male, 1 female (ZMUC), 1 male, 1 female (ZMUM), 1 male, 1 female (NHMW), 1 male, 1 female (NHMUK), Lansak District, Hup Pa Tard, 15°22'26"N, 99°37'58"E, ca. 113 m a.s.l., 27 July 2016, leg. C. Sutcharit, R. Srisonchai, and ASRU members. 9 males, 1 female, 6 broken males and 4 broken females mixed together (CUMZ), Lansak District, Hup Pa Tard, 15°22'26"N, 99°37'58"E, ca. 113 m a.s.l., 19 August 2017, leg. R. Srisonchai and ASRU members. Many specimens (CUMZ), Lansak District, Tham Pha Nam Thip Bureau of Monks, 15°26'03"N, 99°35'24"E, ca. 245 m a.s.l., 27 July 2016, leg. C. Sutcharit, R. Srisonchai, and ASRU members. 17 males, 6 females (CUMZ), Lansak District, Tham Pha Nam Thip Bureau of Monks, 15°26'03"N, 99°35'24"E, ca. 245 m a.s.l., 19 August 2017, leg. R. Srisonchai and ASRU members. 4 males, 6 females, 1 broken male, 2 broken females (CUMZ), Lansak District, Wat Wang Pong (Wat Tham Khoa Chong Lom), 15°16'50"N, 99°43'11"E, ca. 90 m a.s.l., 28 July 2016, leg. C. Sutcharit, R. Srisonchai, and ASRU members. Many specimens (CUMZ), Ban Rai District, Wat Khao Chuak Charoen Tham, 15°16'19"N, 99°41'43"E, ca. 86 m a.s.l., 8 July 2009, leg. S. Panha and ASRU members. 3 males (CUMZ), Ban Rai District, Wat Tham Khao Wong, 15°01'57"N, 99°27'21"E, ca. 259 m a.s.l., 27 October 2013, leg. S. Sutcharit, R. Chanabun, and S. Siriwut.

########### Diagnosis.

Differs from all other *Desmoxytes* species by the combination of the following characters; body purple pink; collum with rows of 4+4 anterior, 1+1 intermediate and 2+2 posterior setiferous tubercles; tip of epiproct slightly or moderately concave; lateral sulcus (ls) deep, long and narrow; lamina lateralis (ll) swollen, surface smooth; ventral lobe (vll) of lamina lateralis long and slender, digitiform, tip round; process (plm) of lamina medialis very long, spine-like, tip usually bifurcating into two distinct spines (in some populations tip terminating in 3–5 spines); distal lobe (dlm) of lamina medialis quite long, distally with two distinct lamellae (mesal lamella smaller and thinner than lateral lamella, tip crenate; lateral lamella broad).

########### Type locality.

THAILAND, Uthai Thani Province, Lansak District, Department of National Parks, Tam Pratun Non–Hunting Area, Hup Pa Tard.

The updated redescription hereunder is modified from Enghoff et al. 2007; some morphological characteristics have been added which were extracted from the type material and all recently collected specimens.

########### Redescription.

SIZE: Length 22–34 mm (male), 26–38 mm (female); width of midbody metazona ca. 2 mm (male), 3.4 mm (female). Width of head < collum = body ring 2 < 3 = 4 < 5–17, thereafter body gradually tapering toward telson.

COLOUR (Fig. 75A–D): In life with body purple to vivid pink; paraterga and metaterga vivid pink to purple; surface below paraterga brownish pink or pink; head brown or blackish brown; antenna black or blackish brown (except distal part of antennomere 7 and antennomere 8 whitish); leg, sterna and epiproct pink; a few basal podomeres pale pink. Colour in alcohol: after 10–11 years changed brownish white, after 2–5 years changed to pale brown.

ANTENNAE (Fig. 76D): Very long and slender, reaching to body ring 7 or 8 (male), and 6 (female) when stretched dorsally.

COLLUM (Fig. 76A): With 3 transverse rows of setiferous tubercles, 4+4 anterior, 1+1 intermediate and 2+2 posterior tubercles, lateral tubercles of posterior row located almost halfway anteriad to intermediate row; paraterga with one conspicuous setiferous notch on lateral margin, elevated at 15°–20°.

TEGUMENT: Slightly shining; collum and metaterga coarsely microgranulate; surface below paraterga finely microgranulate; prozona finely shagreened; paraterga, sterna and epiproct smooth.

METATERGA (Fig. 76A–C): With 2 transverse rows of setiferous rose thorn-like spines; metaterga 2–18 with 2+2 anterior and 2+2 posterior spines; metatergum 19 with 2+2 anterior and 2+2 posterior spines/tubercles.

PARATERGA (Fig. 76E, F): Directed caudolaterad on body rings 2–17, elevated at ca. 45° (male) 40° (female); directed increasingly caudad on body rings 18 and 19; anterior margin with 2 distinct notches, on lateral margin of body rings 9, 10, 12, 13, 15–18 with tiny denticle near the tip.

TELSON (Fig. 77C–G): Epiproct: tip slightly or moderately concave; lateral setiferous tubercles inconspicuous, short; apical tubercles conspicuous, usually short (very long and distinct in Kanchanaburi B population). Hypoproct trapeziform; caudal margin round, with inconspicuous setiferous tubercles.

STERNA (Fig. 78): Cross-impressions shallow. Sternal lobe between male coxae 4 swollen and stout, usually trapeziform (in specimen from Lamphun Province subsemicircular), tip usually round or truncate (in some specimens slightly emarginate).

LEGS (Fig. 77H–J): Very long and slender. Male femora 5 and 6 strongly humped ventrally in middle part.

GONOPODS (Figs 79, 80, 81): Coxa (cx) longer than prefemur. Cannula (ca) long and slender. Prefemur (pfe) ca. 2/3 as long as femur. Femur (fe) long and slender. Mesal sulcus (ms) very deep and wide, lateral sulcus (ls) very deep, long and narrow. Postfemur (pof) conspicuous, ventrally very wide and stout. Solenophore (sph) well-developed: lamina lateralis (ll) swollen, surface smooth; ventral lobe (vll) long and slender, digitiform, tip round, directed ventrad: lamina medialis (lm) well-developed; process (plm) very long, spine-like, tip usually bifurcating into two distinct spines (in some populations tip terminating in 3–5 spines), directed mesoanteriad; distal lobe (dlm) quite long, distally with two distinct lamellae (mesal lamella slightly smaller than lateral one, tip crenate; lateral lamella broad); broad lobe (blm) thick, obviously demarcated from distal lobe (dlm) by conspicuous indentation. Solenomere (sl) long, twisted distally.

########### Distribution and habitat.

Known from several places in many provinces (Kampaeng Phet, Kanchanaburi, Lamphun, Suphan Buri, and Uthai Thani). All specimens were collected from limestone habitats (Fig. 75E); they were very easy to collect because of their aposematic colouration. We noticed that August–September is an annual peak period for adult swarming.

Although this species has been found in several places, it is distributed only in central, west, and north Thailand. Hence, *D. purpurosea* is regarded as endemic for the Thai fauna.

A specimen from Nakhon Sawan Province (Mae Wong National Park, near the type locality of *D. purpurosea*) which really looks very much like *D. purpurosea* is shown on YouTube (“shocking pink dragon millipede - living treasure of the forest at Mae Wong National Park” (https://youtu.be/jQsn6rOrlA8 - in Thai)). Although we cannot confirm this record because we did not examine specimens from this location, according to the known distribution of *D. purpurosea*, the specimen from Nakhon Sawan Province may possibly be this species.

########### Remarks.

Interestingly, all adult specimens in all populations show exactly the same colour as reported in the original description: vivid pink to purple. However, we found morphological variation between four main populations delimited as follows:

1. Lamphun – Tham Erawan.

2. Uthai Thani – Tham Pha Nam Thip, Wat Wang Pong, Wat Khao Chuak Charoen Tham, Wat Tham Khao Wong and Tham Weruwan.

3. Kanchanaburi A – Tham Than Lod Cave.

4. Kanchanaburi B – Daowadueng Cave, Ban Thung Kang Yang, Wat Sunantha Wanaram, Kroeng Krawia Waterfall and Kroeng Krawia Checkpoint.

– Size: Specimens from the Lamphun and Uthai Thani populations are larger than others (length 28–34 mm in male, 32–38 mm in female), whereas specimens from the Kanchanaburi B population seems to be smaller than others (length 22–26 mm in male, 26–28 mm).

– Sternal lobe between male coxae 4: The only studied specimen from the Lamphun population has a subsemicircular and quite short lobe, while in others the lobe is trapeziform.

– Apical tubercles of epiproct: Distinctly longer in the Kanchanaburi B population than in others.

– Process (plm) of lamina lateralis: A bifurcate tip, as two conspicuous spines, in the Lamphun and Uthai Thani populations, but terminating in several spines in the Kanchanaburi A and B populations.

The shape of the hypoproct varies within populations: in some specimens it is trapeziform, in others it is subsemicircular.

*D. purpurosea* shares some morphological characters with *D. breviverpa* and *D. takensis*, viz., metaterga 9–19 with rows of 2+2 (anterior) and 3+3 (posterior) setiferous tubercles/cones/spines. However, the differences in gonopod characters are sufficient for separating these as different species.

########### Coexisting species.

*Desmoxytes octoconigera* sp. n. and *D. golovatchi* sp. n. (see details under these species).

########## Desmoxytes takensis

Taxon classificationAnimaliaPolydesmidaParadoxosomatidae

Srisonchai, Enghoff & Panha, 2016

Figs 82 , 83

Desmoxytes takensis Srisonchai, Enghoff & Panha, 2016: 103.

########### Material examined. Holotype.

Male (CUMZ), THAILAND, Tak Province, Phobphra District, Nangkruen Waterfall, on litters and under decaying bark, 16°24'36.0"N, 98°41'21.0"E, ca. 383 m a.s.l., 15 January 2015, leg. R. Srisonchai, T. Seesamut, and P. Jirapatrasilp.

########### Paratypes.

12 males, 10 females, 1 juvenile (CUMZ), 2 males, 1 female (ZMUC), same data as holotype. 2 males, 1 female (CUMZ), same locality as holotype, 18 January 2011, leg. C. Sutcharit, R. Chanabun, N. Likhitrakarn and T. Krutchuen.

########### Further specimens, 

**all from THAILAND, Kamphaeng Phet Province**: 2 males missing gonopods, 1 female, 2 males (CUMZ), Khlong Lan District, Khlong Lan Waterfall, 16°07'40"N, 99°17'11"E, ca. 189 m a.s.l., 31 May 2009, leg. S. Panha and ASRU members.

**Tak Province, Tha Song Yang District**: 1 male, 8 females, 2 broken males, 1 male missing gonopods, 1 male missing right gonopod (CUMZ), Km 89 on road no. 105 from Mae Sot to Mae Hong Son, limestone mountain, 18 July 2008, leg. S. Panha and ASRU members. 13 juveniles (CUMZ), Km 131 on road no. 105 from Mae Sot to Mae Hong Son, limestone moutain, 30 June 2015, leg. C. Sutcharit, R. Srisonchai and ASRU members.

**Tak Province, Mae Sot District**: 3 males (CUMZ), Chao Por Phawo Shrine, 16°46'19"N, 98°41'11"E, ca. 668 m a.s.l., 17 July 2010, leg. S. Panha and ASRU members. 4 males, 14 females, 1 broken male and missing gonopods, 6 broken males, (12 males, all remaining rings 1–8), many broken and mixed specimens (CUMZ), Chao Por Phawo Shrine, 16°46'19"N, 98°41'11"E, ca. 668 m a.s.l., 17 July 2010, leg. S. Panha and ASRU members. 1 male missing gonopods, 1 male, 1 broken male (CUMZ), Chao Por Phawo Shrine, 16°46'19"N, 98°41'11"E, ca. 668 m a.s.l., 26 September 2010, leg. S. Panha and ASRU members. 10 males, 8 females (CUMZ), Chao Por Phawo Shrine, 16°46'19"N, 98°41'11"E, ca. 668 m a.s.l., 29 June 2015, leg. C. Sutcharit and ASRU members. 15 mixed specimens (CUMZ), Chao Por Phawo Shrine, 16°46'19"N, 98°41'11"E, ca. 668 m a.s.l., 19 October 2015, leg. C. Sutcharit and R. Srisonchai. 3 females (CUMZ), Chao Por Phawo Shrine, 16°46'19"N, 98°41'11"E, ca. 668 m a.s.l., 27 July 2016, leg. P. Pimvichai, P. Prasankok, and N. Nantarat. 54 males, 16 females (CUMZ) , 2 males, 1 female (ZMUC), 1 male (NHMW), 1 male (NHMUK), Chao Por Phawo Shrine, 16°46'19"N, 98°41'11"E, ca. 668 m a.s.l., 29 August 2016, leg. S. Panha and ASRU members. 15 specimens (CUMZ), Wat Tham Inthanin, 16°45'59"N, 98°40'21"E, ca. 671 m a.s.l., 19 October 2015, leg. C. Sutcharit and R. Srisonchai. 13 males, 11 females (CUMZ), Wat Pho Thi Khun (Wat Huai Toey), 16°45'42"N, 98°38'49"E, ca. 432 m a.s.l., 29 August 2016, leg. C. Sutcharit and R. Srisonchai.

**Tak Province, Umphang District**: 15 males, 13 females, 1 male missing right gonopod, 1 male missing left gonopod, 4 males missing gonopods (CUMZ), Tham Takhobi (Takhobi Cave), 16°03'15"N, 98°49'14"E, ca. 511 m a.s.l., 5 July 2009, leg. S. Panha and ASRU members. 7 males, 11 females (CUMZ), Tham Takhobi (Takhobi Cave), 16°03'15"N, 98°49'14"E, ca. 511 m a.s.l., 5 July 2009, leg. S. Panha and ASRU members. 2 males, 5 females, 1 male missing right gonopod, 8 males missing gonopods, 2 broken males, many broken and mixed specimens (CUMZ), Doi Hua Mod, 15°57'30"N, 98°51'13"E, ca. 893 m a.s.l., 5 July 2009, leg. S. Panha and ASRU members. 6 males, 7 females (CUMZ) Doi Hua Mod, 15°57'30"N, 98°51'13"E, ca. 893 m a.s.l., 1 July 2015, leg. S. Panha and ASRU members. 1 male, 1 female (CUMZ), Mae Klong Kee Bureau of Monks, 16°13'46"N, 98°55'12"E, ca. 586 m a.s.l., 5 July 2009, leg. C. Sutcharit, R. Srisonchai, and ASRU members. 1 male, 1 female (CUMZ), Ban Ta Per Pru – Wa Krue Kro, 16°10'49"N, 98°52'48"E, ca. 523 m a.s.l., 30 June 2015, leg. C. Sutcharit, R. Srisonchai, and ASRU members. 29 males, 47 females, 5 broken males, 4 broken females; 1 male missing gonopods, 1 broken male and missing gonopods, 1 male remaining rings 7–20, 1 female remaining rings 1–10 (CUMZ), Km 162 on road no.1090 from Mae Sot to Umphang (near Chao Por Phawo Shrine Umphang), 16°02'23"N, 98°50'60"E, ca. 483 m a.s.l., 6 July 2009, leg. S. Panha and ASRU members. 3 males, 7 females, 1 juvenile (CUMZ), Ban Ta Per Pru – Wa Krue Kro Village, 16°10'49"N, 98°52'48"E, ca. 523 m a.s.l., 30 June 2015, leg. C. Sutcharit and ASRU members. 3 males, 1 female, 4 juveniles (CUMZ), Ban Kra Per Pru, 16°12'15"N, 98°52'04"E, ca. 628 m a.s.l., 2 July 2015, leg. C. Sutcharit and ASRU members.

########### Type locality.

THAILAND, Tak Province, Phobphra District, Nangkruen Waterfall.

########### Diagnosis.

Differs from all other *Desmoxytes* species by the combination of the following characters; lamina lateralis (ll) subtriangular; ventral lobe (vll) of lamina lateralis thumb-like, large and long; broad lobe (blm) of lamina medialis indistinctly demarcated from distal lobe (dlm) of lamina medialis by very shallow or slightly deep indentation.

########### Redescription (updated from Srisonchai et al. 2016).

SIZE: Population A (see Remarks): Length 24–26 mm (male), 25–27 mm (female); width of midbody metazona ca. 1.9 mm (male), 2.2 mm (female). Width of head = collum = body ring 2 = 3 = 4 < 5 < 6–16, thereafter body gradually tapering toward telson. Population B (see Remarks): Length 29–31 mm (male), 32–35 mm (female); width of midbody metazona ca. 1.9 mm (male), 2.3 mm (female). Width of head < collum > body ring 2 > 3 > 4 < 5–16, thereafter body gradually tapering toward telson.

**Figure 82.**
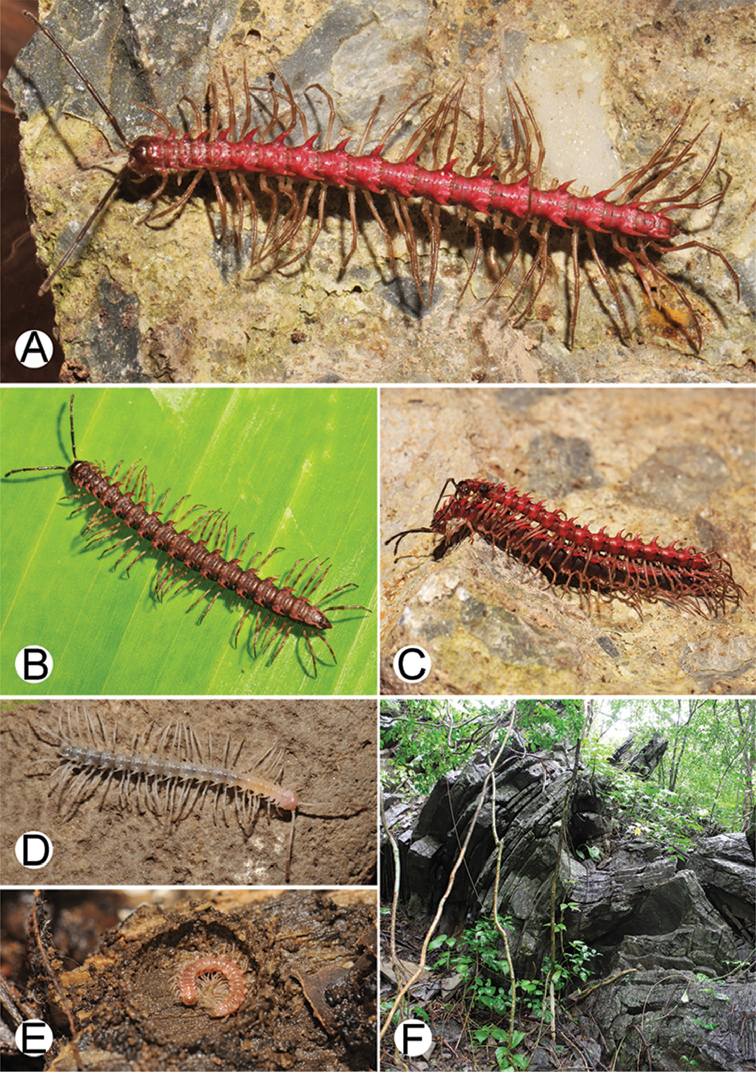
*Desmoxytes takensis* Srisonchai et al., 2016 (specimen from Chao Por Phawo Shrine) and habitat. **A** male **B** female **C** mating couple **D** early adult **E** moulting chamber **F** habitat.

COLOUR (Fig. 82A–D): Population A (see below): In life with body bright red; paraterga, metaterga and surface below paraterga red; head, antennae (distal part of antennomere 7 and antennomere 8 whitish), a few basal podomeres, sterna and epiproct brownish red. Population B (see below): In life with body bright pink; paraterga, metaterga and surface below paraterga bright pink; head brown; antenna blackish brown (except distal part of antennomere 7 and antennomere 8 whitish); legs brownish pink to brown; a few basal podomeres pale brown to whitish; sterna and epiproct brownish pink. Colour in alcohol: after one year changed to pale brown or almost whitish in some specimens.

ANTENNAE: Very long and slender, reaching to body ring 6 or beginning of 7 (male) and 5 (female) when stretched dorsally.

COLLUM: With 3 transverse rows of setiferous tubercles, 4+4 anterior, 1+1 intermediate and 2+2 posterior tubercles (lateral tubercles of posterior row located almost halfway to intermediate row); paraterga of collum low, elevated at ca. 10°–15°, directed caudolaterad, with one distinct notch on lateral margin.

TEGUMENT: Slightly shining; collum and metaterga coarsely microgranulate; prozona finely shagreened; surface below paraterga finely microgranulate; paraterga, sterna and epiproct smooth.

METATERGA: With 2 transverse rows of setae, setiferous tubercles and setiferous spines; metaterga 2–8 with 2+2 anterior and 2+2 posterior spines; metaterga 9–17 with 2+2 anterior and 3+3 posterior spines; metatergum 18 with 2+2 anterior spines and 3+3 posterior tubercles; metatergum 19 with 2+2 anterior and 3+3 posterior setae or tubercles.

PARATERGA: Directed caudolaterad on body rings 2–17, elevated at ca. 45° (male) 40° (female); directed increasingly caudad on body rings 18 and 19; anterior margin with 2 distinct notches, on lateral margin of body rings 9, 10, 12, 13, 15–18 with tiny denticle near the tip.

TELSON: Epiproct: tip subtruncate; lateral setiferous tubercles conspicuous, short; apical tubercles inconspicuous. Hypoproct subsemicircular (population B subtrapeziform); caudal margin round, with inconspicuous setiferous tubercles.

STERNA: Cross-impressions shallow. Sternal lobe between male coxae 4 swollen, subtrapeziform when seen in caudal view; base enlarged, slightly attenuated near tip; tip round (in population B subtruncate).

LEGS: Very long and slender. Male femora 5 and 6 strongly humped ventrally in middle part.

**Figure 83.**
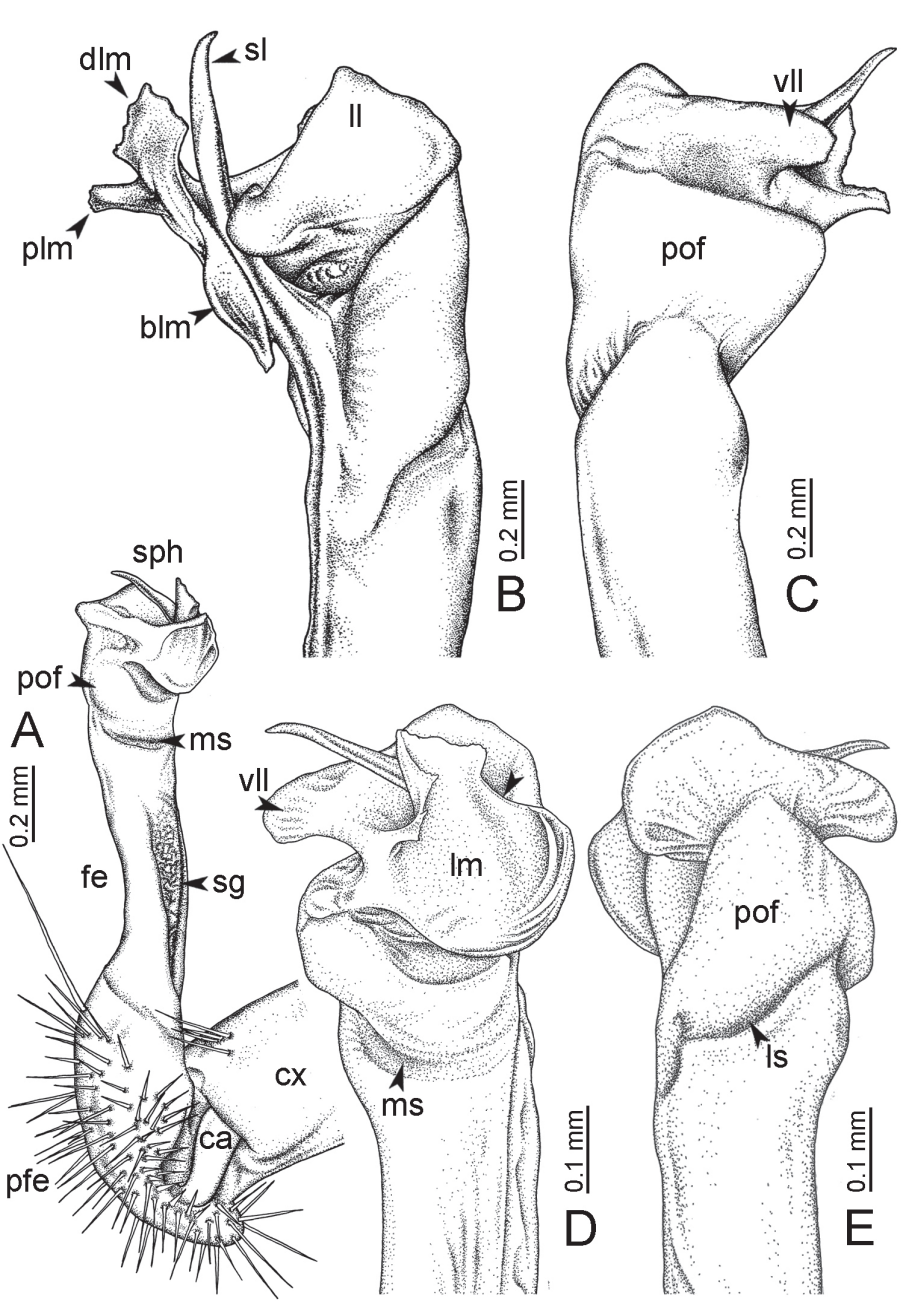
*Desmoxytes takensis* Srisonchai et al., 2016 (paratype) – right gonopod (modified from Srisonchai et al. 2016). **A** mesal view **B** dorsal view **C** ventral view **D** submesal view (arrow = indentation) **E** lateral view.

GONOPODS (Fig. 83): Coxa (cx) longer than prefemur. Cannula (ca) slender. Prefemur (pfe) ca. 2/3 as long as femur. Femur (fe) long and slender. Mesal sulcus (ms) and lateral sulcus (ls) very deep and wide. Postfemur (pof) conspicuous, ventrally wide. Solenophore (sph) well-developed: lamina lateralis (ll) swollen, like a triangular lobe when seen in lateral view; ventral lobe (vll) thumb-like, large (in population B thumb-like, longer and more slender than in population A), directed ventrad: lamina medialis (lm) well-developed; process (plm) long, directed mesad, tip usually sharp (in some specimens almost blunt); distal lobe (dlm) well-developed, distally with one broad and thin lamella (in population B distally with two lamellae; mesal lamella very small, crest-like; lateral lamella broad and thin, *in situ* terminating close to tip of solenomere); broad lobe (blm) thick, indistinctly demarcated from distal lobe (dlm) by a usually wide and shallow indentation (in some specimens demarcated from distal lobe (dlm) by a deep indentation). Solenomere (sl) quite long.

########### Distribution and habitat.

Known only from Tak and Kamphaeng Phet Provinces. This species is restricted to limestone habitats and was seen crawling on litter and decaying bark (Fig. 82F). Srisonchai et al. (2016) reported that *D. takensis* was found on humid plastic garbage, a sign that the type locality is clearly under human influence. There is a long, broad concrete natural trail into the waterfall, and lots of garbage littered the type locality. However, the species has also been found in other natural habitats.

During our intensive surveys in western Thailand, we found this species in many places. However, it has a narrow range and occurs in only two provinces. Thus, *D. takensis* should be regarded to be endemic to the Thai fauna.

########### Remarks.

Based on morphology, we divided our material specimens into two main populations: Population A includes specimens from Nangkruen Waterfall (type locality), Tham Takhobi, Doi Hua Mod, Mae Klong Kee Bureau of Monks, Ban Ta Per Pru – Wa Krue Kro, Km 162 on road no.1090 near Chao Por Phawo Shrine Umphang and Ban Kra Per Pru. Population B includes specimens from Khlong Lan Waterfall, Km 89 on road no. 105 from Mae Sot to Mae Hong Son, Km 131 on road no. 105 from Mae Sot to Mae Hong Son, Chao Por Phawo Shrine (Mae Sot), Wat Tham Inthanin and Wat Pho Thi Khun.

The two populations differ in some characters as follows:

– Colour: The remarkable body colour of the two populations apparently differs: bright red in population A, vivid pink in population B (some old females strongly pinkish to reddish).

– Size: Population B individuals seem to be bigger than population A ones in both width and length (see size description).

– Hypoproct: The shape of the hypoproct in population A is subsemicircular whereas it is subtrapeziform in population B.

– Gonopods: The ventral lobe (vll) of lamina lateralis of population B specimens is large, thumb-like, longer, and more slender than that of population A ones. The distal lobe (dlm) of lamina medialis in population A specimens consists of one lamella while population B specimens have two lamellae distally.

Although the two populations vary in some morphological characters, they show an overall gonopodal resemblance. According to the differences in morphology of the two populations, this might an example of ongoing speciation in allopatry, supported by the confinement of the two populations to two large isolated limestone regions located in the northern (Population B) and southern (Population A) parts of the distribution area.

We collected some juveniles during the field trip and kept them with litter until they moulted. Interestingly, the juveniles made a moulting chamber which was apparently produced by fecal material and silk; it is probable that the building process is the same as in the families Polydesmidae, Pyrgodesmidae and in order Callipodida (Adis et al. 2000, Youngsteadt 2009, Reboleira and Enghoff 2016). This is the first observation of moulting in dragon millipedes; however, we did not keep an eye on them in detail. After moulting and emerging from the chamber, the specimens were in an early adult stage showing a pale whitish colouration. Nearly 2 weeks later, they became vivid pink (Fig. 82D, E).

########### Coexisting species.

None known.

########### Corrections to Srisonchai et al. (2016)

Srisonchai et al. (2016: 99–103) wrote in the description of this species that the paraterga (including paraterga of collum) are directed dorsolaterad at ca. 30°. They are in fact directed caudolaterad and are elevated at ca. as 45°.

########## Desmoxytes taurina

Taxon classificationAnimaliaPolydesmidaParadoxosomatidae

(Pocock, 1895)

**Figure 84.**
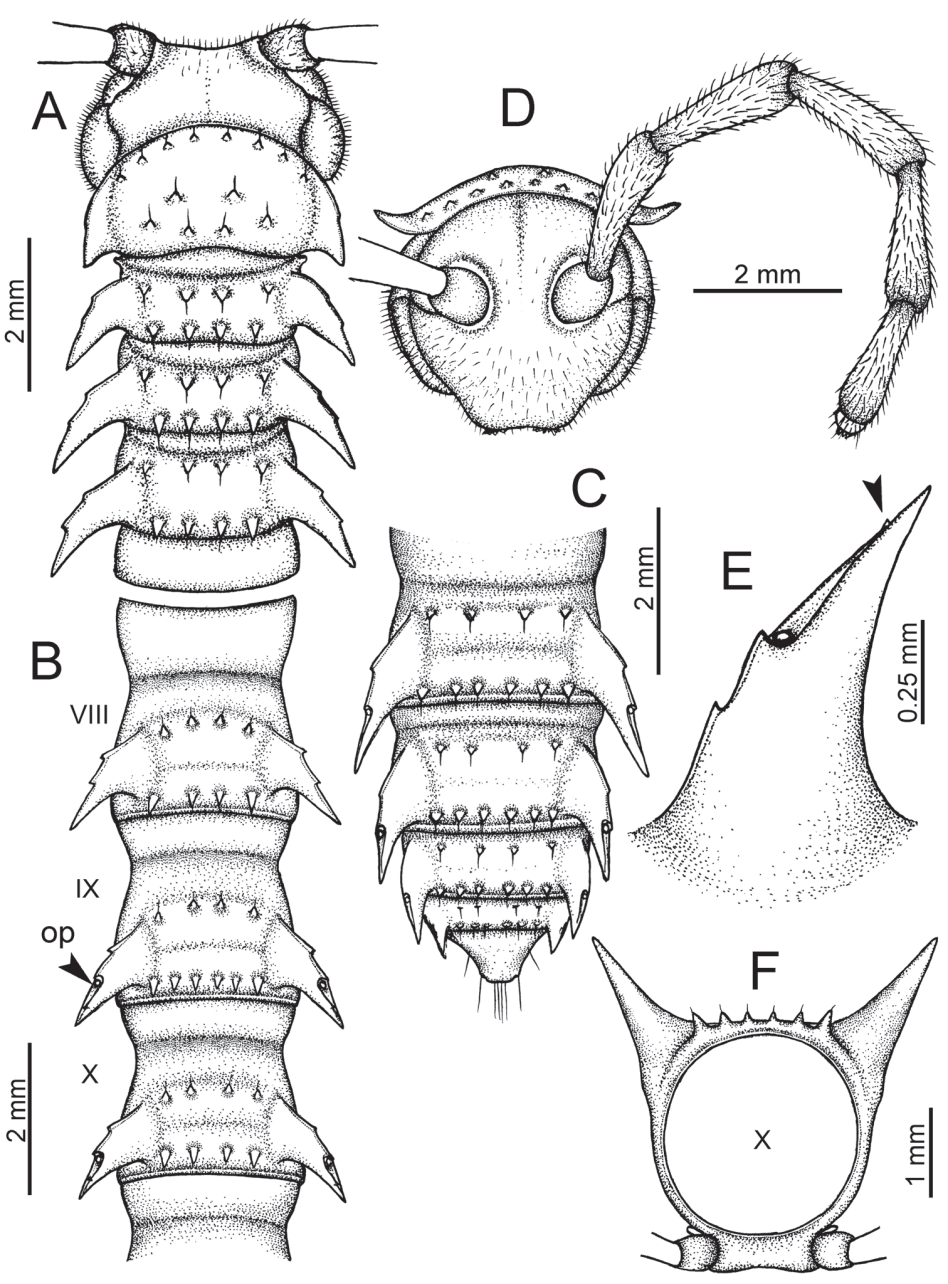
*Desmoxytes taurina* (Pocock, 1895), lectotype. **A** anterior body part **B** body rings 8–10 (op = ozopore) **C** posteriormost body rings and telson **D** head and antenna **E** paraterga of ring 10 (arrow = tinydenticle) **F** body ring 10.

**Figure 85.**
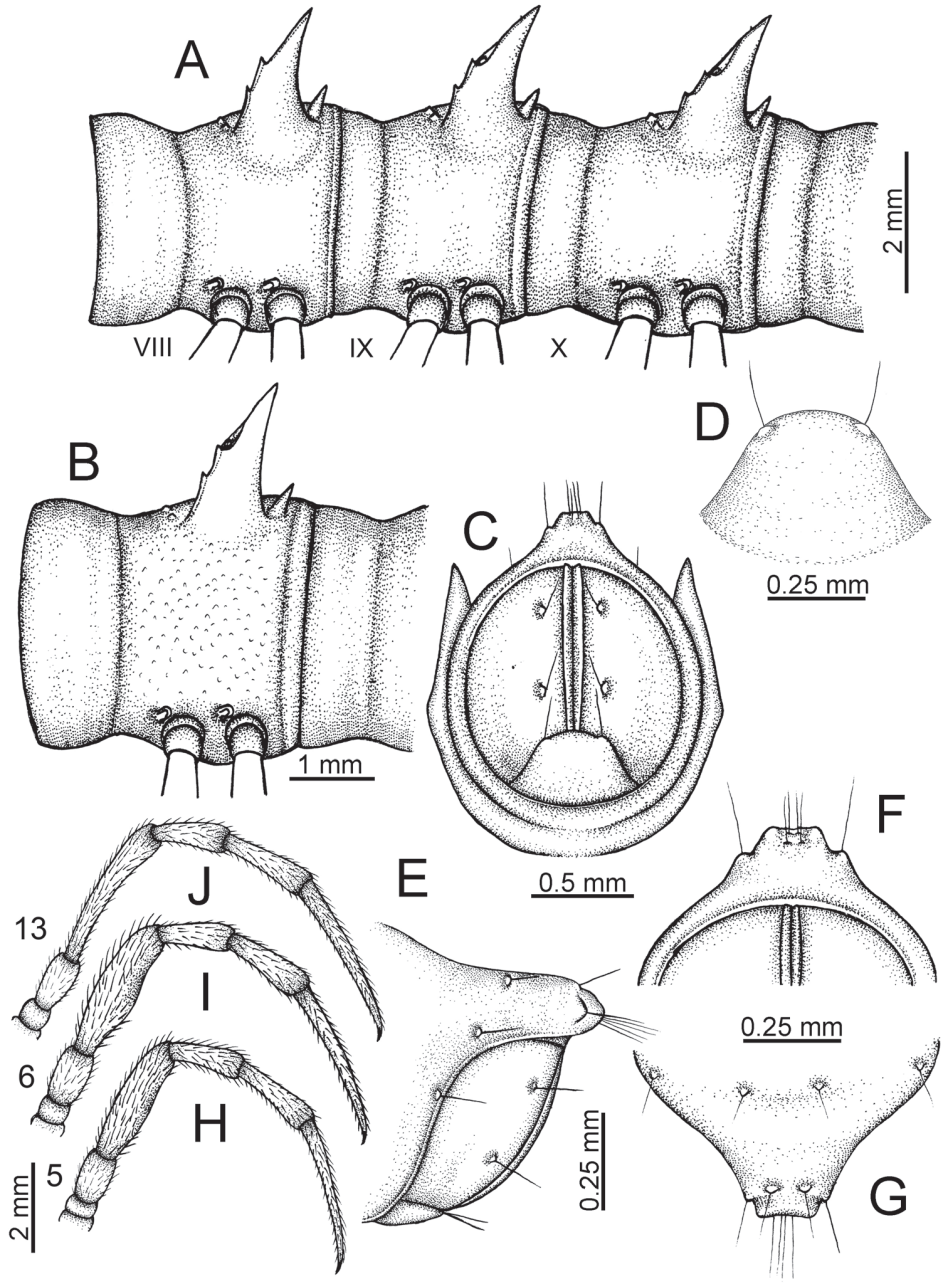
*Desmoxytes taurina* (Pocock, 1895), lectotype. **A** body rings 8–10 **B** sculpture of ring 10 **C, E** last ring and telson **D** hypoproct **F, G** epiproct **H** male leg 5 (right) **I** male leg 6 (right) **J** male leg 13 (right).

**Figure 86.**
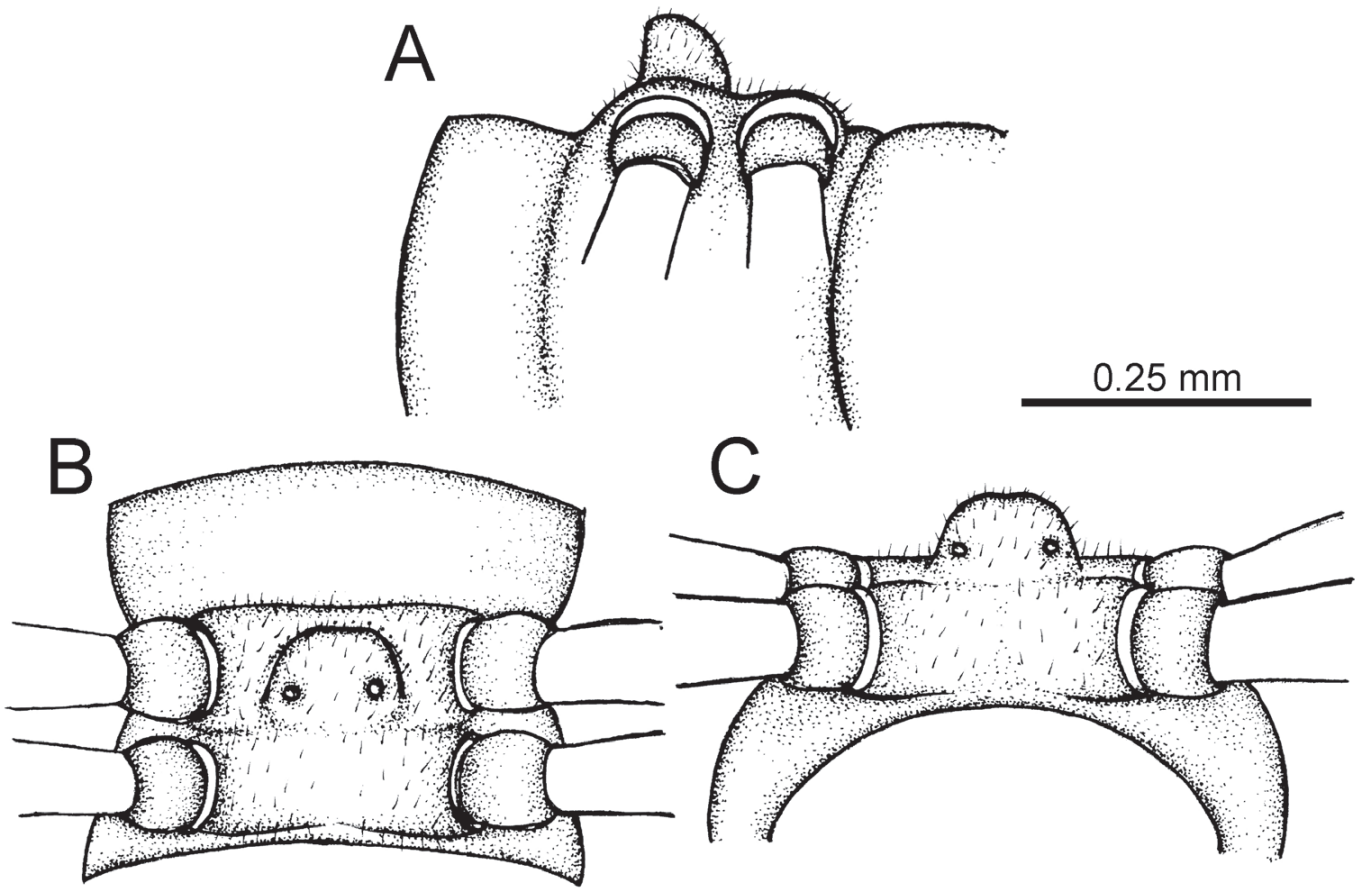
*Desmoxytes taurina* (Pocock, 1895), lectotype – sternal lobe between male coxae 4. **A** lateral view **B** ventral view **C** caudal view.

**Figure 87.**
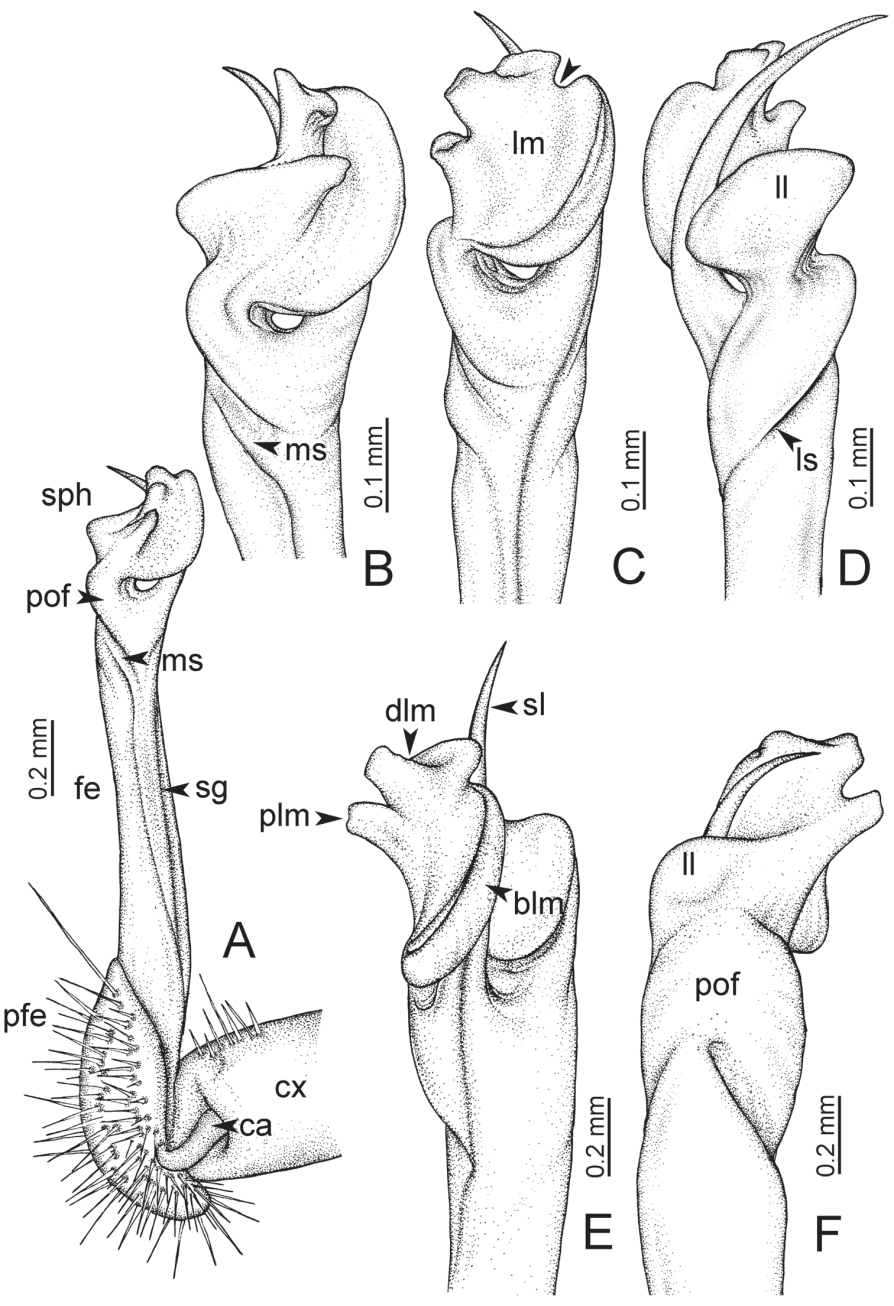
*Desmoxytes taurina* (Pocock, 1895), lectotype – right gonopod. **A** mesal view **B** mesal view **C** submesal view (arrow = indentation) **D** lateral view **E** dorsal view **F** ventral view.

Figs 84 , 85 , 86 , 87

Prionopeltis taurinus Pocock, 1895: 830. Attems 1914: 204. Weidner 1960: 89. Jeekel 1965: 124.
Pratinus taurinus – Attems 1937: 121. Jeekel 1964: 63; 1968: 61.
Desmoxytes taurina – Jeekel 1980a: 655. Golovatch and Enghoff 1994: 57. Nguyen and Sierwald 2013: 1243. Likhitrakarn et al. 2017: 20.

########### Material examined.

**Lectotype.** Male (rings 1–11 only, with gonopod – pinned through body) (ZMUC), MYANMAR, Pegu (Taikkyii and Palon), leg. Fea. Lectotype here designated.

**Paralectotypes.** 1 male (2–3 broken rings in very poor condition, without gonopods) (ZMUC), MYANMAR, Pegu (Taikkyii and Palon), leg. Fea. 2 females (1 female, complete – pinned through body; 1 female, remaining rings 11–20 – pinned through body) (NHMUK), MYANMAR, Rangoon, leg. E. W. Oates [Yangon].

########### Diagnosis.

Metaterga 9–19 usually with 2+2 cones/spines (anterior row) and 3+3 cones/spines (posterior row). Similar in this respect to *D. breviverpa, D. purpurosea* and *D. takensis*. Differs from these species by the following combination of characters; process (plm) of lamina medialis short, thick and broad, directed mesad, tip blunt; distal lobe (dlm) apically with two distinct lamellae, mesal and lateral lamellae equal in size, very broad and thick; epiproct short; male femora 5 and 6 slightly humped ventrally.

########### Type locality.

Myanmar, Pegu (Taikkyii and Palon).

########### Redescription.

SIZE: Length ca. 23 mm (male), ca. 27 mm (female) width of midbody metazona ca. 1.7 mm (male), 2.3 mm (female). Width of head < collum < body ring 2 < 3 = 4 < 5–16, thereafter body gradually tapering toward telson.

COLOUR: In life with body probably brownish black (Pocock, 1895) or castaneous brown? (Golovatch and Enghoff, 1994). Colour in alcohol: after ca. 100 years changed to pale brown (lectotype) or rusty brown (paralectotypes).

ANTENNAE (Fig. 84D): Long and slender, probably reaching to body ring 5 (male) and 4–5 (female) when stretched dorsally.

COLLUM (Fig. 84A): With 3 transverse rows of setiferous tubercles, 4+4 anterior, 1+1 intermediate and 2+2 posterior tubercles (lateral tubercles of anterior row located near base of paraterga); paraterga of collum low, elevated almost in horizontal plane, directed caudolaterad, with one inconspicuous notch on lateral margin.

TEGUMENT: Quite dull; collum and metaterga coarsely microgranulate; prozona finely shagreened; surface below paraterga and sterna finely microgranulate; paraterga and epiproct smooth.

METATERGA (Fig. 84A–C): With 2 transverse rows of setiferous tubercles and setiferous cones; metaterga 2–8 with 2+2 anterior and 2(3)+2(3) posterior cones; metaterga 9–18 with 2+2 anterior and 3(2)+3(2) posterior cones (anterior cones shorter than posterior ones); metatergum 19 with 2+2 anterior and 3+3 posterior tubercles.

PARATERGA (Fig. 84E, F): Directed caudolaterad on body rings 2–17, elevated at ca. 50° (male) 45° (female); directed increasingly caudad on body rings 18 and 19; anterior margin with 2 distinct notches, on lateral margin of body rings 9, 10, 12, 13, 15–18 with tiny denticle near the tip.

TELSON (Fig. 85C–G): Epiproct short; tip truncate; lateral setiferous tubercles and apical tubercles inconspicuous. Hypoproct subtrapeziform; caudal margin round, with inconspicuous setiferous tubercles.

STERNA (Fig. 86): Cross-impressions shallow. Sternal lobe between male coxae 4 swollen, stout, subquadrate when seen in caudal view; base enlarged, slightly attenuated near tip; tip slightly emarginate.

LEGS (Fig. 85H–J): Very long and slender. Male femora 5 and 6 slightly humped ventrally in middle part.

GONOPODS (Fig. 87): Coxa (cx) longer than prefemur. Cannula (ca) slender. Prefemur (pfe) ca. 2/3 as long as femur. Femur (fe) long and slender. Mesal sulcus (ms) and lateral sulcus (ls) very deep. Postfemur (pof) conspicuous, ventrally wide. Solenophore (sph) well-developed: lamina lateralis (ll) swollen, stout: lamina medialis (lm) well-developed; process (plm) slightly short, thick and broad, directed mesad, tip blunt; distal lobe (dlm) well-developed, distally with two distinct lamellae (mesal lamella and lateral lamella equal in size, very broad and thick); broad lobe (blm) very thick, distinctly demarcated from distal lobe (dlm) by a conspicuous, shallow indentation. Solenomere (sl) quite long.

########### Distribution and habitat.

This species is known from Myanmar – Rangoon and Pegu (Taikkyii and Palon). [Rangoon is currently Yangon. Pegu is presently Bago township (Hanthawaddy), Bago region. Taikkyii is Taikkyi township, Yangon region. Palon is probably a small village located in the area north to Taikkyii in the west of Pegu, Yangon region]. Taikkyii and Palon were formerly parts of Pegu region, now they belong to Yangon region. Therefore, the label of specimens collected by Fea gives the locality as Pegu (Taikkyii and Palon).

Habitat details for this species have never been reported; however, all locations are supposed to be granitic and limestone mountain ranges based on geological data, and the two locations were approximately 20 km apart.

We assume that *D. taurina* is distributed in a narrow range. A field survey near Yangon in 2015 revealed no further specimens of *D. taurina*. Therefore, we regard this species as endemic to Myanmar.

########### Note on material.

In the original description Pocock (1895), wrote that all specimens were collected from Rangoon by Oates and from Pegu (Taikkyii and Palon) by Fea. Two females collected by Oates are now in NHMUK, one specimen collected by Fea in ZMH and two males collected by Fea in ZMUC.

The two males in ZMUC collected by Fea are labelled “cotypes”, and only “Palon” is given as locality whereas in the original description Pocock gave “Pegu (Taikkyii and Palon)”. We assume that these two males were probably collected from different locations, one from Taikkyii and one from Palon.

Weidner (1960) classified a specimen (unknown sex, not studied by us) of *Prionopeltis taurinus* (= *Desmoxytes taurina*) in ZMH as a “paratypoid”. However, Pocock (1895) and the following authors did not designate a holotype or lectotype for this species, thus, all specimens are syntypes.

The lectotype chosen is the ZMUC male with one remaining gonopod. The other ZMUC, ZMH and NHMUK specimens are designated here as paralectotypes.

Colour of type specimens: the lectotype is brown without metallic oxidation of the pin while the paralectotypes in NHMUK have become greenish black with metallic oxidation of the pin.

########### Remarks.

This species has not been revised since Golovatch and Enghoff gave a good description in 1994. Golovatch and Enghoff (1994) described the collum with rows of 3(4?)+3(4?) anterior tubercles, suture between prozona and metazona distinctly beaded, pleurosternal carinae absent. After examining all known specimens except the one in ZMH, we found:

– collum with rows of 4+4 anterior tubercles (lateral tubercles near base of paraterga).

– suture between prozona and metazona not beaded, but with very small ridges of irregular shape.

– pleurosternal carinae of all specimens conspicuously present on body ring 2, very small ridges on body ring 3, thereafter missing.

We noticed that the number of cones (posterior row) on metaterga varies between individuals. Most specimens have metaterga 8 with 2+2 tubercles in the posterior row, but some have 3+2 tubercles. Metaterga 9–19 usually have 3+3 tubercles in the posterior row, whereas some individuals have with 3+4 or 4+3 tubercles.

The length of antenna in male could not be examined (antennae missing in both males), but the antennae are supposed to reach to ring 5 (Pocock 1895).

########### Coexisting species.

None known.

########## Desmoxytes terae

Taxon classificationAnimaliaPolydesmidaParadoxosomatidae

(Jeekel, 1964)

**Figure 88.**
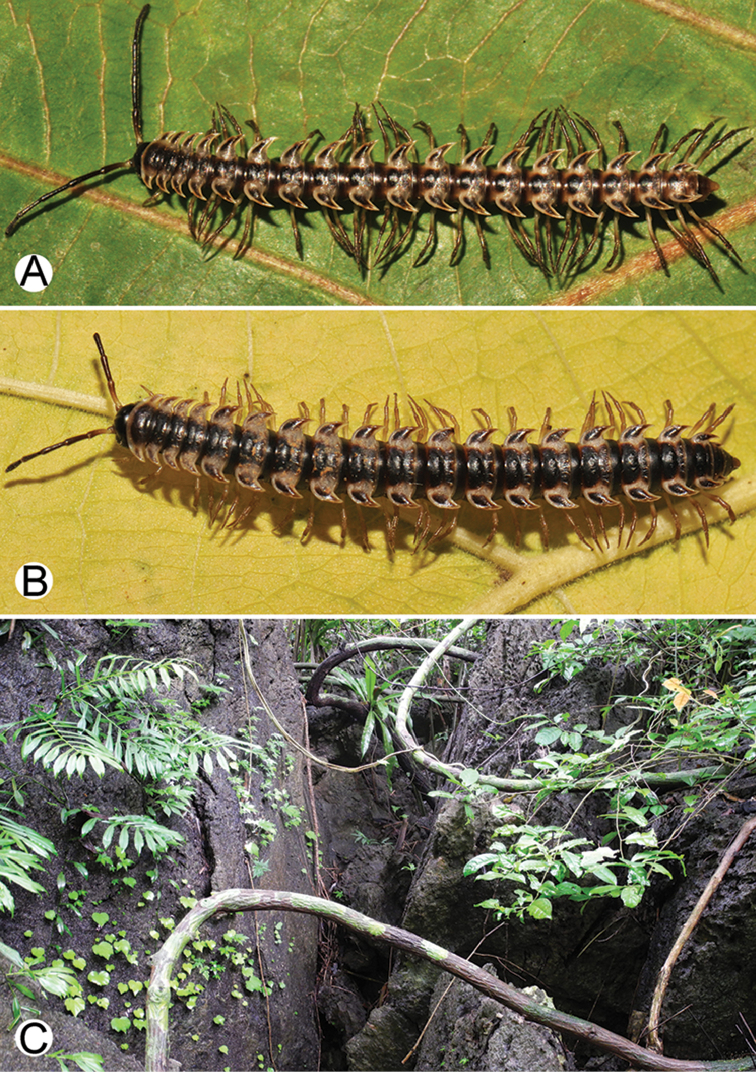
Photographs of live *Desmoxytes terae* (Jeekel, 1964) (specimens from Tham Tone Din) and habitat. **A** male **B** female **C** habitat.

**Figure 89.**
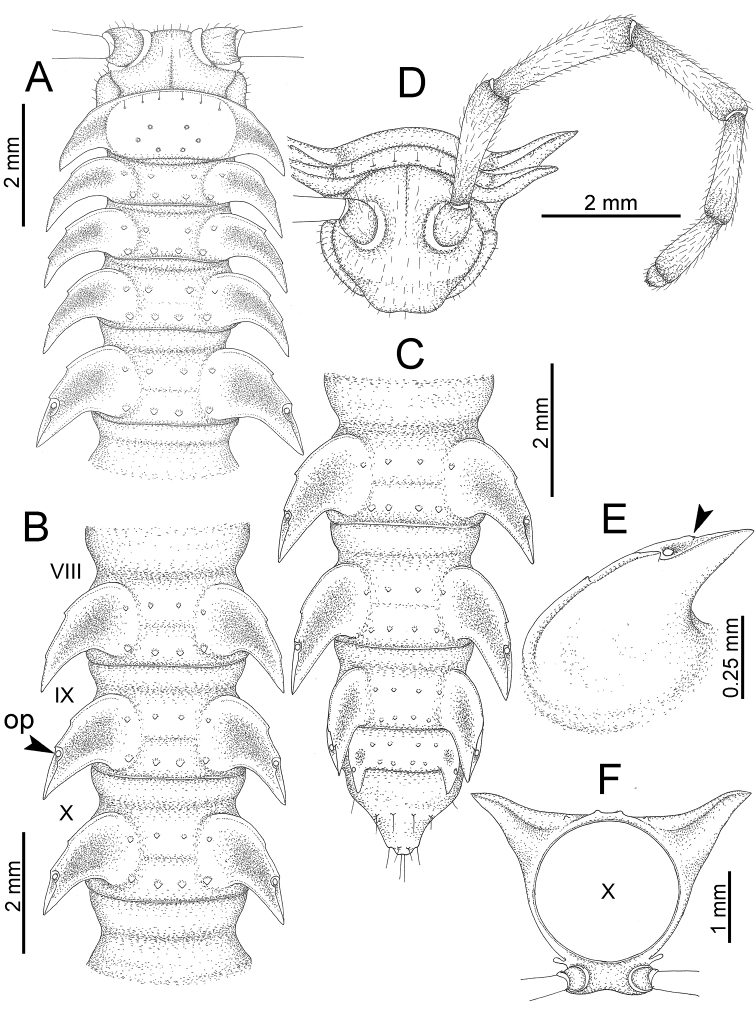
*Desmoxytes terae* (Jeekel, 1964), specimen from Tham Tone Din. **A** anterior body part **B** body rings 8–10 **C** posteriormost body rings and telson **D** head and antenna **E** paraterga of ring 10 **F** body ring 10.

**Figure 90.**
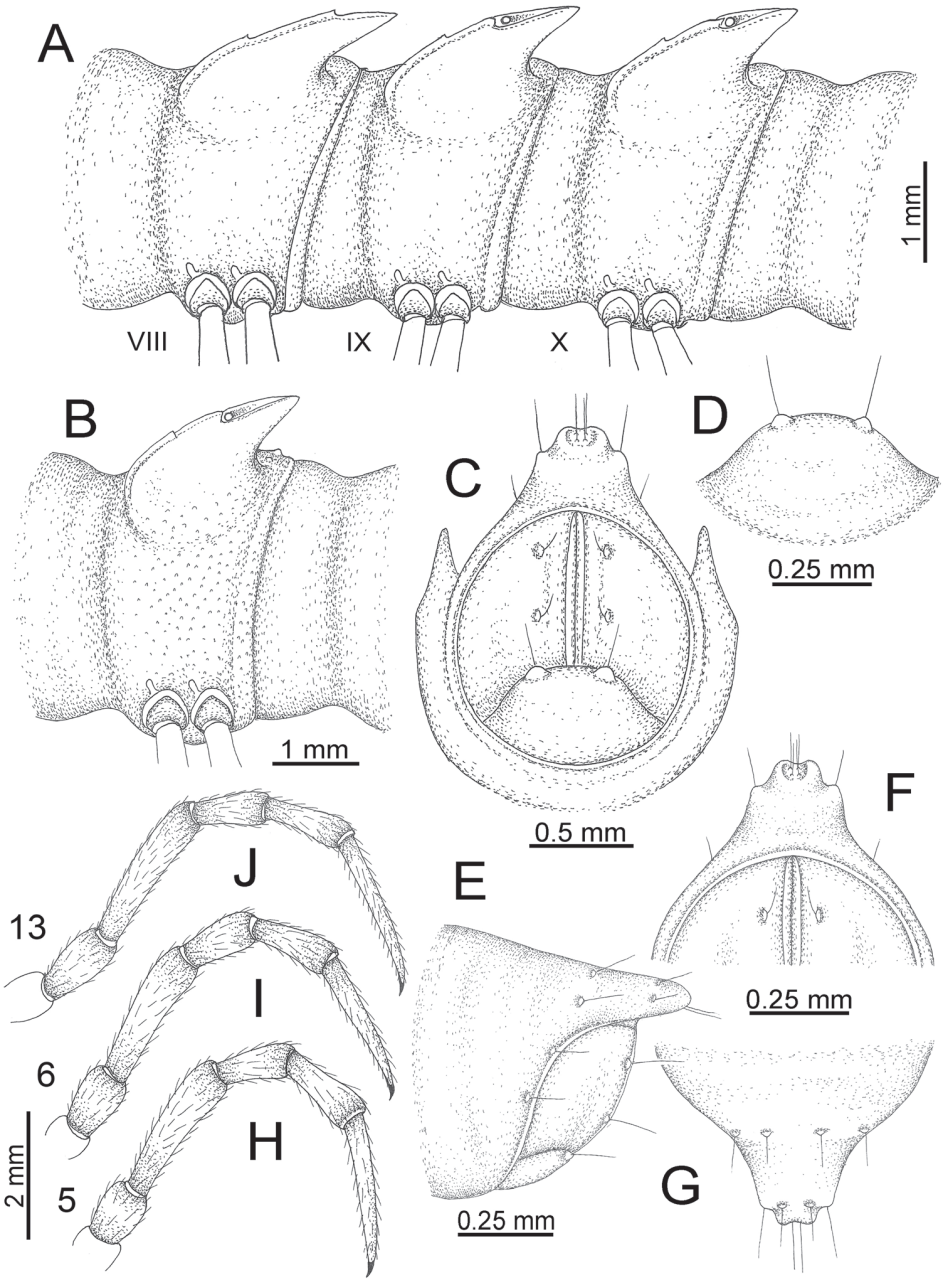
*Desmoxytes terae* (Jeekel, 1964), specimen from Tham Tone Din. **A** body rings 8–10 **B** sculpture of ring 10 **C, E** last ring and telson **D** hypoproct **F, G** epiproct **H** male leg 5 (right) **I** male leg 6 (right) **J** male leg 13 (right).

**Figure 91.**
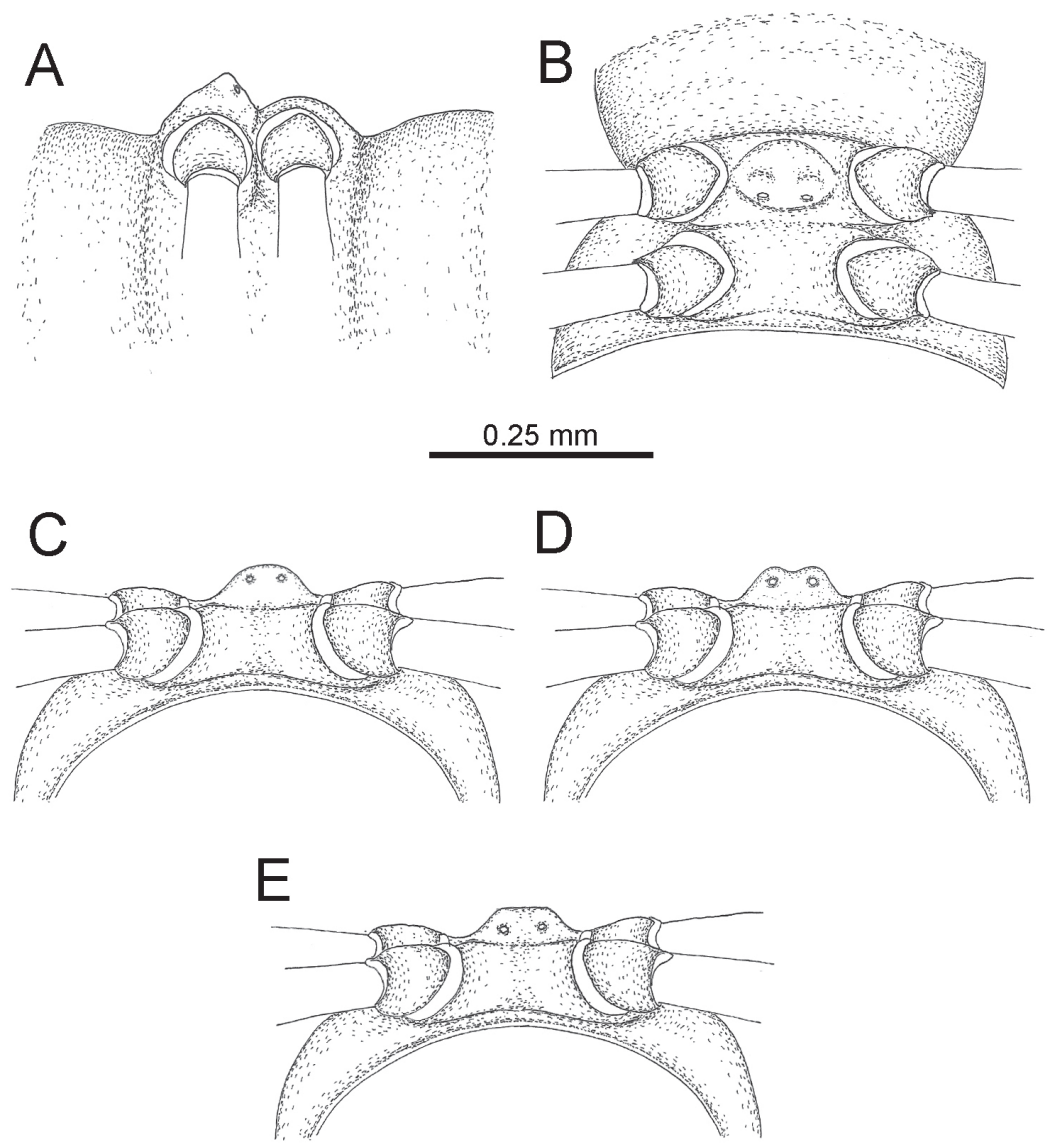
*Desmoxytes terae* (Jeekel, 1964), specimen from Tham Tone Din – sternal lobe between male coxae 4. **A** lateral view **B** mesal view **C–E** caudal view.

**Figure 92.**
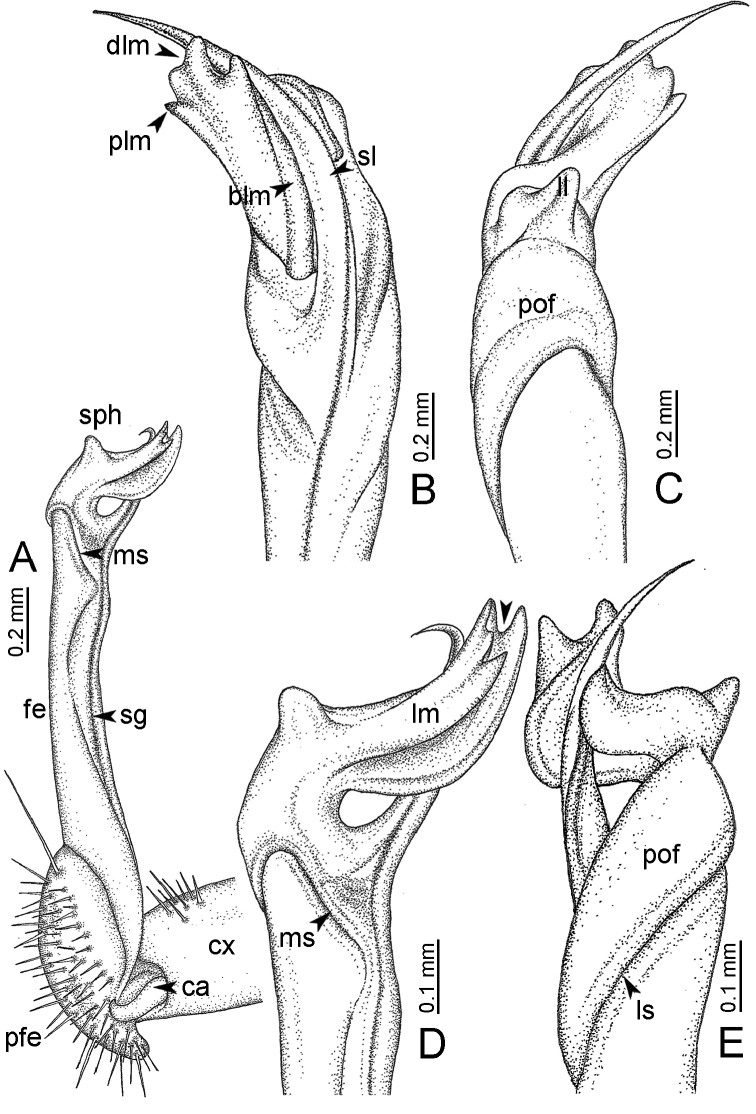
*Desmoxytes terae* (Jeekel, 1964), holotype – right gonopod. **A** mesal view **B** dorsal view **C** ventral view **D** submesal view (arrow = indentation) **E** lateral view.

Figs 88 , 89 , 90 , 91 , 92

Pratinus terae Jeekel, 1964: 69; 1968: 51.
Pteroxytes terae – Jeekel 1980a: 655.
Desmoxytes terae – Golovatch and Enghoff 1994: 59. Enghoff 2005: 97. Nguyen and Sierwald 2013: 1243.

########### Material examined.

**Holotype.** Male (NBC), MALAYSIA, Perlis, Kaki Bukit, near Kampong Wang Tangga, 19 December 1958, leg. W.S.S van der Feen-van Benthem Jutting.

**Paratypes.** 1 female, 1 female fragment (NBC), same data as holotype.

########### Further specimens, all from THAILAND, Satun Province:

1 male (ZMUC), Thale Ban National Park, in logs, litter, under stones, 6°42'N, 100°10'E, 8 November 1990, leg. M. Andersen and A. R. Rasmussen. 1 female (CUMZ), Khuan Don District, Thale Ban National Park, Tham Tone Din (Tone Din Cave), 6°43'35"N, 100°09'45"E, ca. 154 m a.s.l., 31 August 2015, leg. S. Sutcharit, A. Pholyotha, T. Seesamut, and R. Srisonchai. 2 males, 6 females (CUMZ), 1 female (ZMUC), Khuan Don District, Thale Ban National Park, Tham Tone Din (Tone Din Cave), 6°43'35"N, 100°09'45"E, ca. 154 m a.s.l., 7 July 2017, leg. S. Sutcharit, R. Srisonchai, and ASRU members.

########### Diagnosis.

Differs from all other *Desmoxytes* species by the combination of the following characters; body black or brownish black contrasting with yellowish white paraterga with a triangular dorsal, dark spot; sternal lobe between male coxae 4 short and stout, broad at base, trapeziform or semicircular; male femora 5 and 6 without modification; lamina lateralis (ll) with big and long lobe-like structure projecting ventroanteriad.

########### Type locality.

MALAYSIA, Perlis, Kaki Bukit, near Kampong Wang Tangga.

The redescription hereunder is modified from Jeekel (1964); we ‘harmonised’ descriptions of all morphological characters and added some morphological characteristics from additional specimens.

########### Redescription.

SIZE: Length 28–32 mm (male), 28–34 mm (female); width of midbody metazona ca. 2.4 mm (male), 3.1 mm (female). Width of head < collum ≤ body ring 2 ≤ 3 = 4 < 5–17, thereafter body gradually tapering toward telson.

COLOUR (Fig. 88A, B): In life with body black or brownish black; paraterga yellowish white (dorsal side with triangular dark brown spot); metaterga, surface below paraterga and prozona black or brownish black; head black; antenna brownish black (except distal part of antennomere 7 and antennomere 8 whitish); legs brown; sterna brown to yellowish brown; a few basal podomeres and epiproct pale brown. Colour in alcohol: after 49 years changed to whitish brown, 2–5 years changed to pale blackish brown.

ANTENNAE (Fig. 89D): Long and slender, reaching to body ring 6 (male), and 5 (female) when stretched dorsally.

COLLUM (Fig. 89A): With 3 transverse rows of setae and tubercles, 3+3 anterior setae, 1+1 intermediate tubercles and 2+2 posterior tubercles (tubercles small, without setae), lateral tubercles of posterior row located more anteriorly, almost halfway to intermediate row; paraterga of collum with two distinct setiferous notches on lateral margin, directed caudolaterad, almost horizontal.

TEGUMENT: Quite dull, sometimes shining; collum coarsely microgranulate; prozona, metaterga and surface below paraterga finely microgranulate; paraterga smooth (dorsal side finely microgranulate); sterna and epiproct smooth.

METATERGA (Fig. 89A–C): With 2 transverse rows of tubercles; metaterga 2–19 with 2+2 anterior and 2+2 posterior tubercles.

PARATERGA (Fig. 89E, F): Directed caudolaterad on body rings 2–17, elevated ca. 5°–10° above the horizontal plane in both sexes; directed increasingly caudad on body rings 18 and 19; anterior margin with 2 distinct notches, on lateral margin of body rings 9, 10, 12, 13, 15–18 with very small and tiny denticle near the tip.

TELSON (Fig. 90C–G): Epiproct: tip usually subtruncate (in some specimens slightly emarginate); lateral setiferous tubercles usually conspicuous (in some specimens inconspicuous), quite short; apical tubercles inconspicuous. Hypoproct trapeziform; caudal margin usually subtruncate (in some specimens slightly round), with big and conspicuous setiferous tubercles.

STERNA (Fig. 91): Cross-impressions quite deep. Sternal lobe between male coxae 4 swollen, short and stout, broad at base, usually trapeziform (in some specimens semicircular), tip usually truncate (in some specimens round).

LEGS (Fig. 90H–J): Long and slender. Male femora 5 and 6 without modification.

GONOPODS (Fig. 92): Coxa (cx) longer than prefemur. Cannula (ca) slender. Prefemur (pfe) ca. 2/3 as long as femur, quite short and stout. Femur (fe) long and slender. Mesal sulcus (ms) and lateral sulcus (ls) deep. Postfemur (pof) conspicuous, ventrally wide. Solenophore (sph) well-developed: lamina lateralis (ll) swollen, with a big and long lobe-like structure projecting ventroanteriad: lamina medialis (lm) well-developed, longer than lamina lateralis; process (plm) short; distal lobe (dlm) quite broad, distally with two lamellae (mesal lamella shorter than lateral one); broad lobe (blm) long and thick, obviously demarcated from distal lobe (dlm) by a slightly deep and wide indentation. Solenomere (sl) very long.

########### Distribution and habitat.

This species is known only from the Malaysia–Thailand borderland (Kaki Bukit and Thale Ban National Park). We noticed that it prefers to live on humid mosses, logs or litter in limestone habitats (Fig. 88C). The localities are all on limestone mountains. The species is probably distributed in a narrow range in limestone areas near the Thai–Malay border. *D. terae* has so far been recorded from three localities (near Kaki Bukit, Thale Ban National Park, and Tham Tone Din) which are located only 10–15 km apart. Despite several attempts by us, *D. terae* was not found in other areas. Hence, this species should be regarded as endemic to the Malaysia and Thailand faunas.

########### Remarks.

In the recent field surveys we noticed that the colour of living specimens is black or brownish black with contrasting white paraterga as reported earlier by Jeekel (1964) and Golovatch and Enghoff (1994). This species blends so perfectly with its environment that it is difficult to collect specimens without a flashlight.

In the original description, Jeekel (1964) stated about the collum: “near the anterior margin a transverse row of six hairs, which may be present partly rubbed off, the lateral pair placed on the low tubercles”. This means collum with one row of 3+3 anterior setae/tubercles. Moreover, Jeekel also described paraterga without a tiny denticle near the tip. After we examined all specimens, it is clear that:

– Collum with 3 rows of 3+3 anterior setae, 1+1 intermediate tubercles and 2+2 posterior tubercles (tubercles without setae, quite small but conspicuous).

– Paraterga of body rings 9, 10, 12, 13, 15–18 with tiny denticle near ther tip, albeit all quite small.

The sternal lobe between male coxae 4 shows some variation within populations; the lobe of some specimens is trapeziform whereas in others it is semicircular. We also found some variability on the telson: tip of epiproct subtruncate in some individuals, in others slightly emarginate; lateral setiferous tubercles conspicuous in some specimens, inconspicuous in others; caudal margin of hypoproct truncate in some individuals, slightly round in others.

########### Coexisting species.

This species and *D. delfae* are sympatric at Tam Ton Din.

########## Desmoxytes waepyanensis

Taxon classificationAnimaliaPolydesmidaParadoxosomatidae

Srisonchai, Enghoff & Panha sp. n.

http://zoobank.org/5CDE2570-0D2F-4574-B492-6AA807709F91

**Figure 93.**
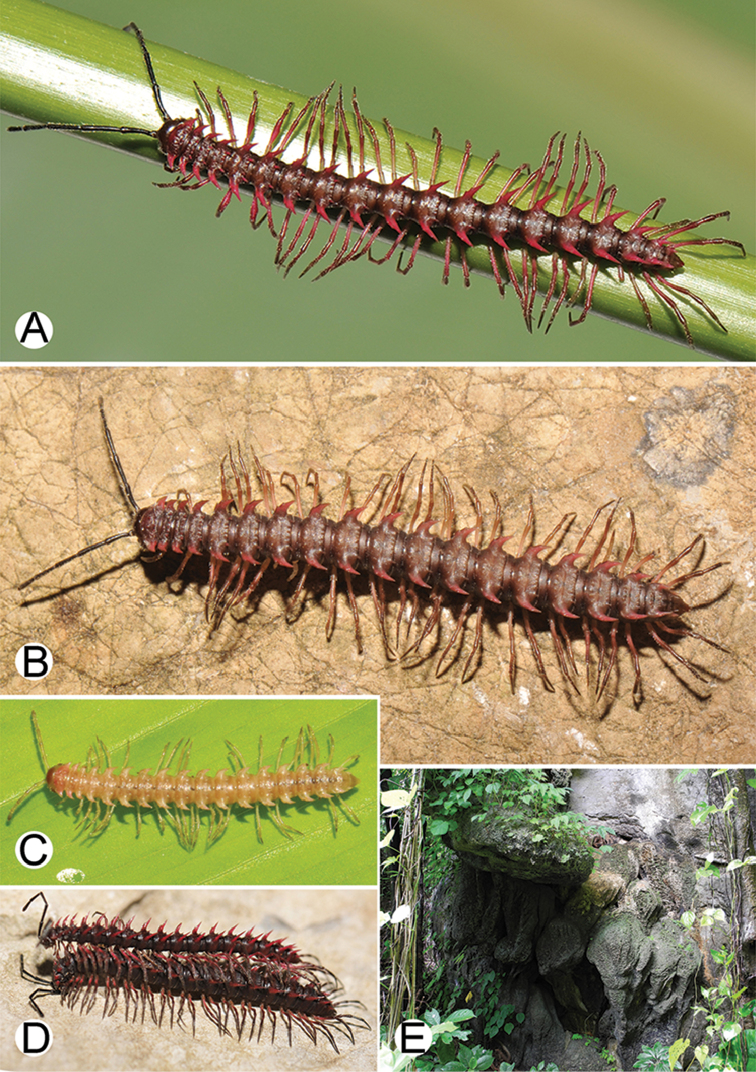
Photographs of live *Desmoxytes waepyanensis* sp. n. and habitat. **A** male paratype **B** female paratype **C** juvenile **D** mating couple **E** habitat.

**Figure 94.**
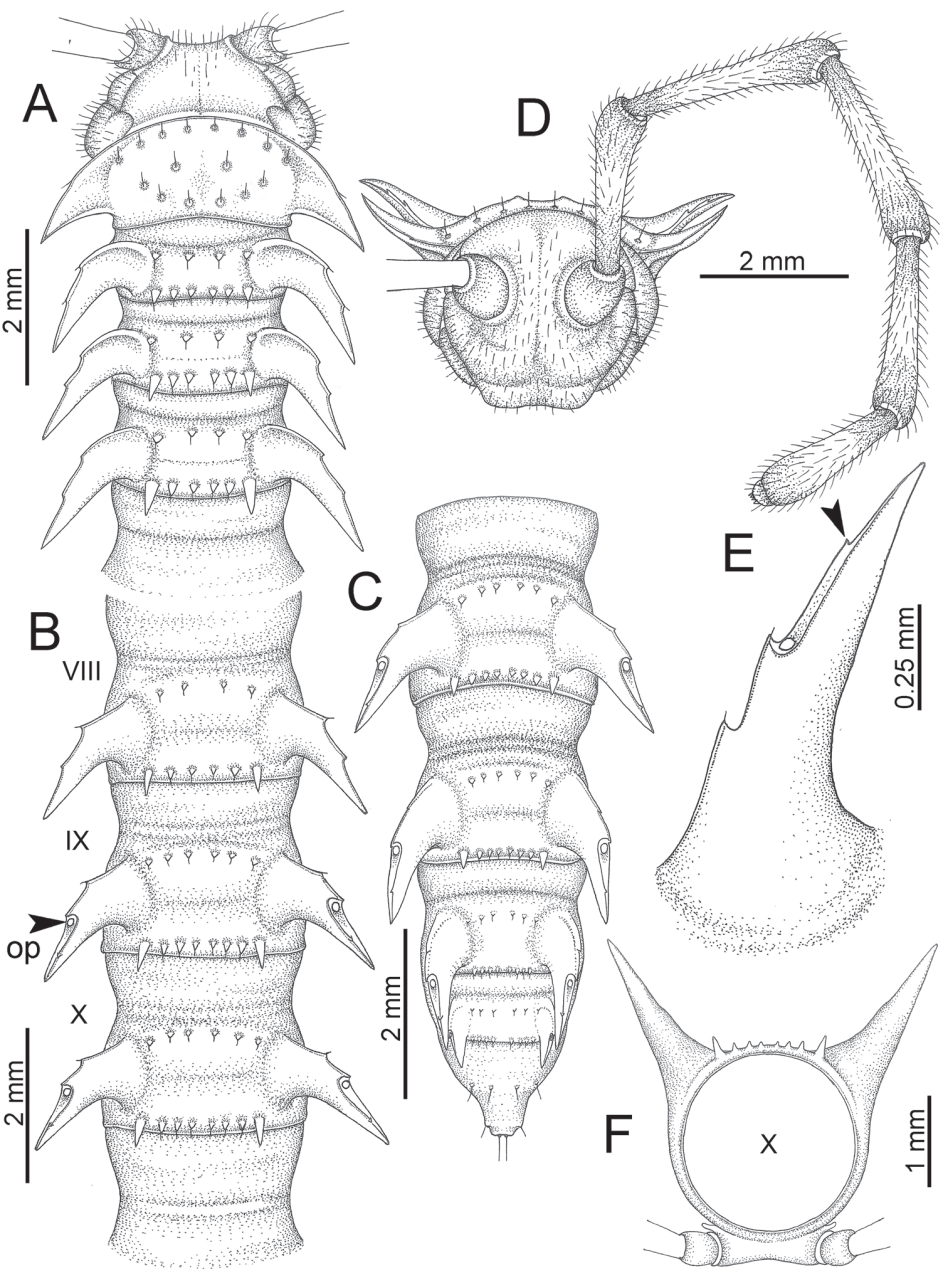
*Desmoxytes waepyanensis* sp. n. (male paratype). **A** anterior body part **B** body rings 8–10 (op = ozopore) **C** posteriormost body rings and telson **D** head and antenna **E** paraterga of ring 10 (arrow = tiny denticle) **F** body ring 10.

**Figure 95.**
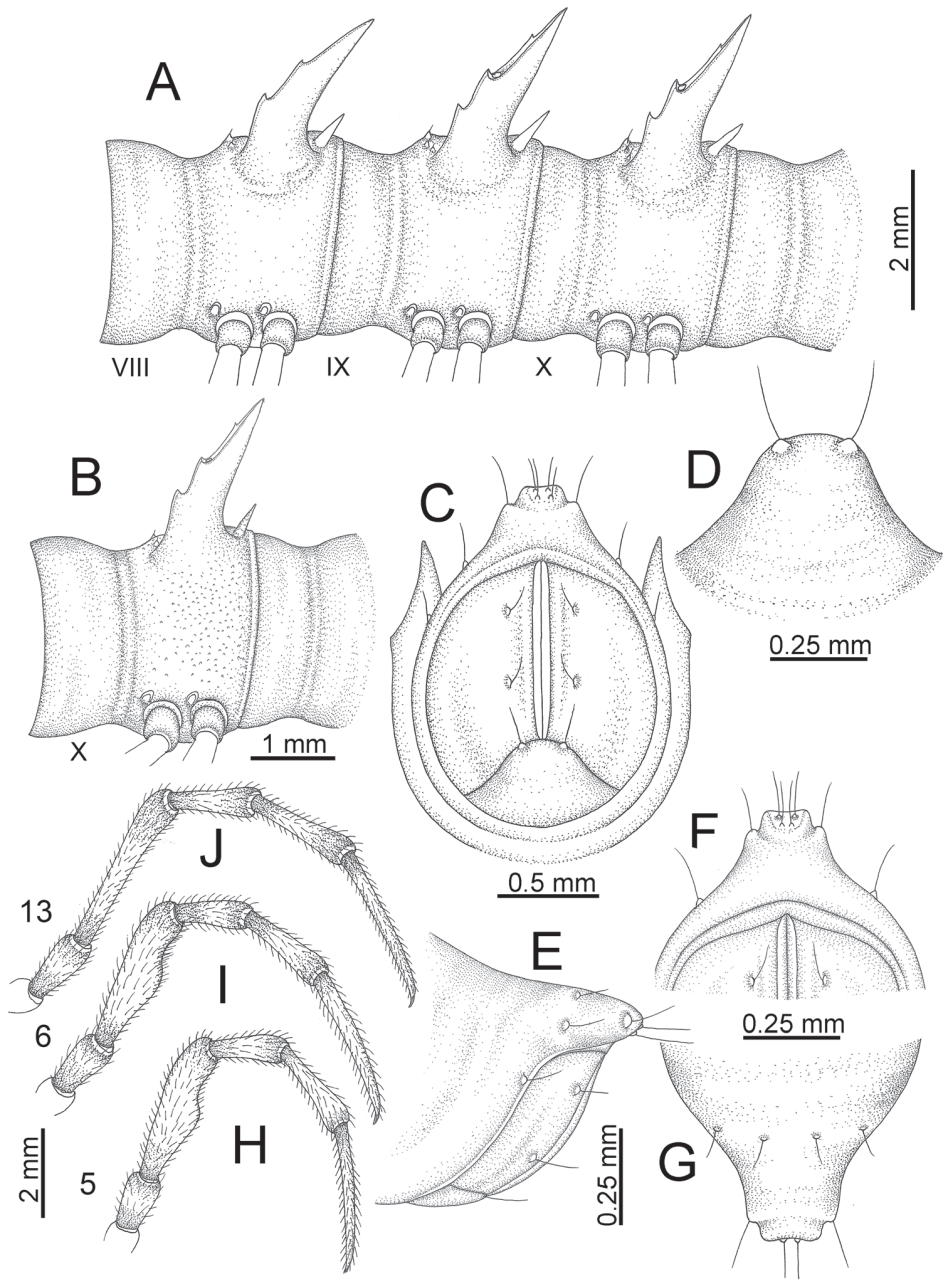
*Desmoxytes waepyanensis* sp. n. (male paratype). **A** body rings 8–10 **B** sculpture of ring 10 **C, E** last ring and telson **D** hypoproct **F, G** epiproct **H** male leg 5 (right) **I** male leg 6 (right) **J** male leg 13 (right).

**Figure 96.**
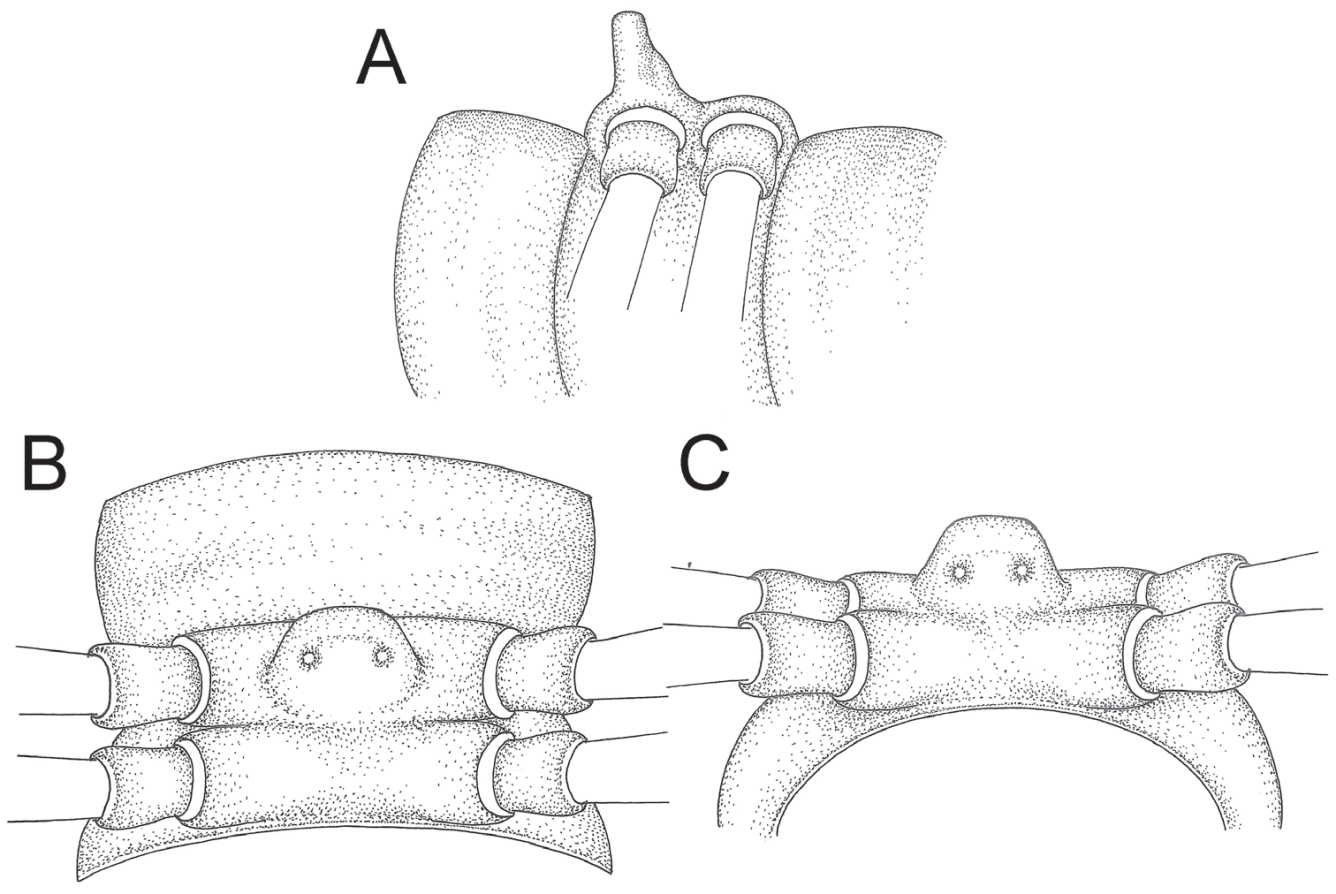
*Desmoxytes waepyanensis* sp. n. (male paratype) – sternal lobe between male coxae 4. **A** lateral view **B** ventral view **C** caudal view.

**Figure 97.**
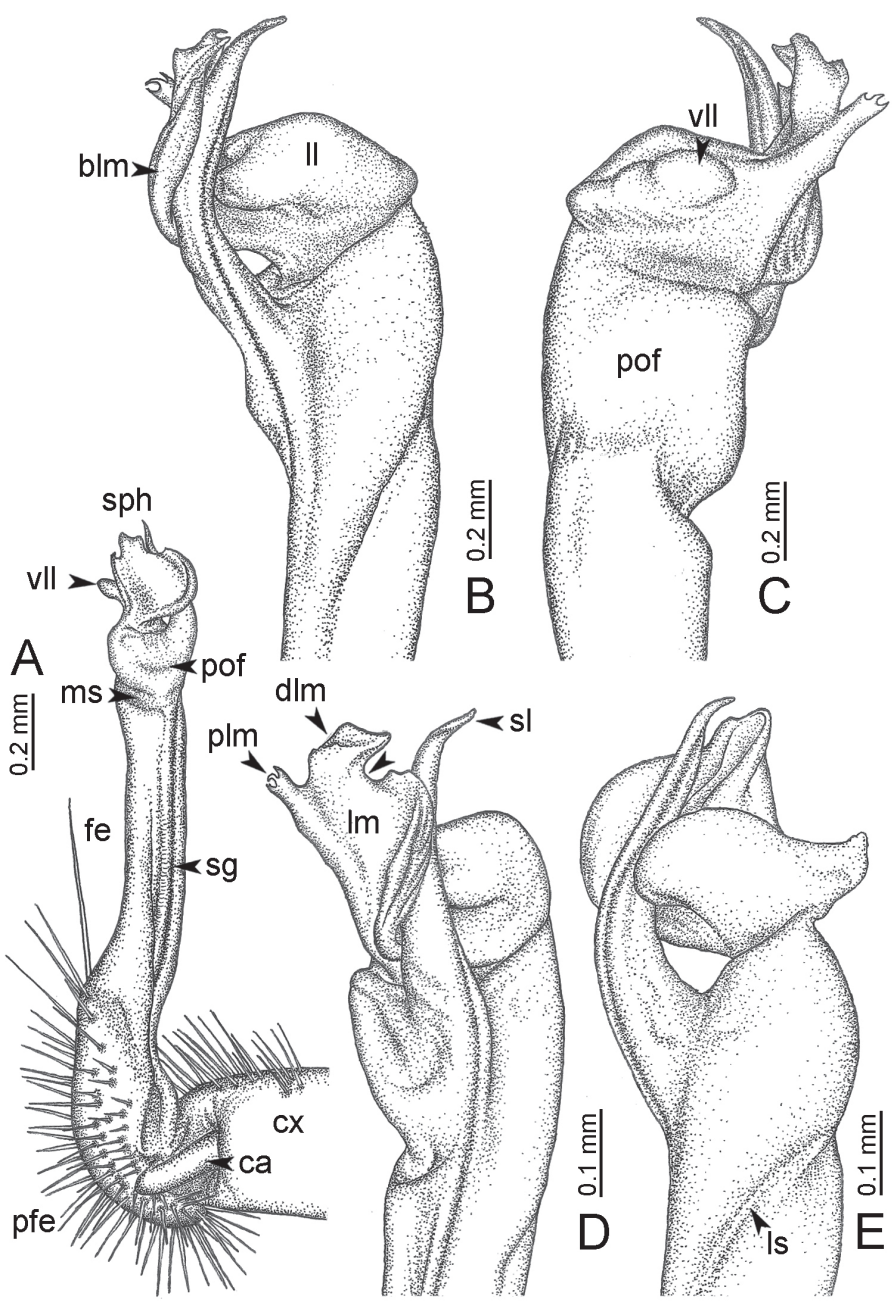
*Desmoxytes waepyanensis* sp. n. (paratype) – right gonopod. **A** mesal view **B** dorsal view **C** ventral view **D** subdorsal view (arrow = indentation) **E** lateral view.

**Figure 98.**
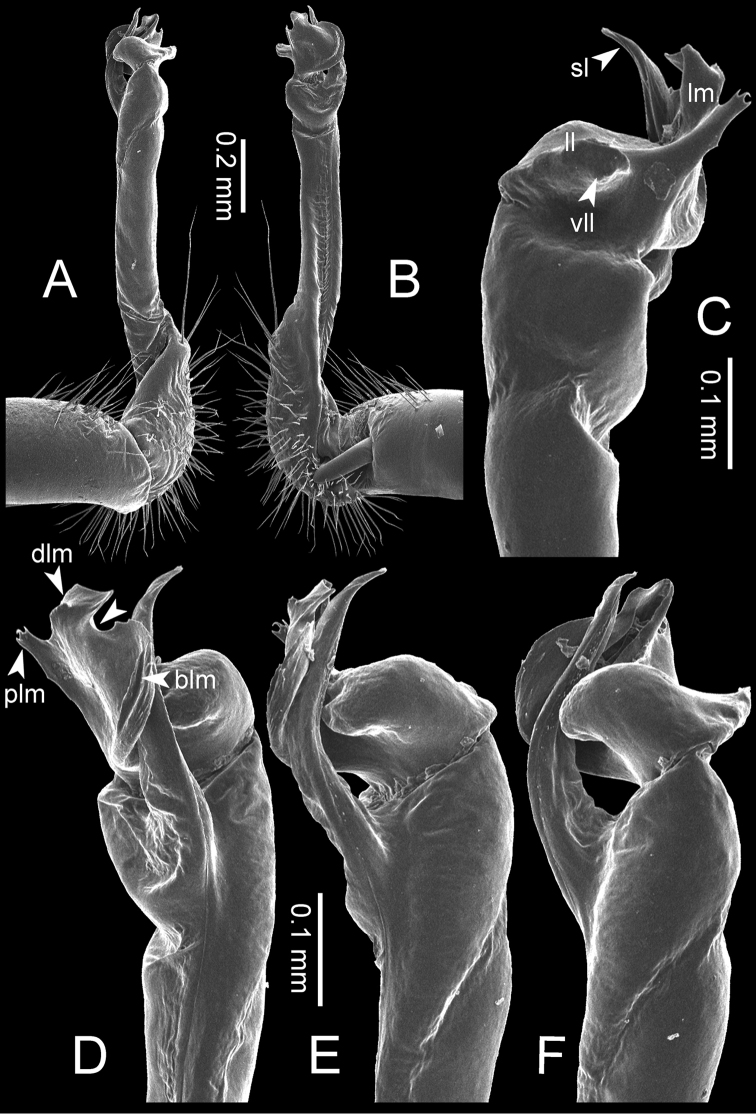
*Desmoxytes waepyanensis* sp. n. (paratype) – right gonopod. **A** lateral view **B** mesal view **C** ventral view **D** subdorsal view (arrow = indentation) **E** dorsal view **F** subdorsal view.

Figs 93 , 94 , 95 , 96 , 97 , 98

########### Holotype.

Male (CUMZ), MYANMAR, Kayin State, 12 km south of Kamarmuang City, Wae Pyan Cave, 17°13'38"N, 97°37'24"E, ca. 24 m a.s.l., 20 June 2015, leg. S. Panha and FFI staffs.

########### Paratypes.

5 males, 12 females, 1 juvenile (CUMZ), 1 male, 1 female (ZMUC), same data as holotype.

########### Diagnosis.

Differs from all congeners by having: metaterga 2–8 with two rows of 2+2 (anterior) setiferous cones and 3(2)+3(2) (posterior) setiferous spines; metaterga 9–18 with two rows of 3+3 (anterior) setiferous cones and 4(3)+4(3) (posterior) setiferous spines; ventral lobe (vll) of lamina lateralis short and stout, digitiform; process (plm) of lamina lateralis tube-like, quite long.

########### Etymology.

The name is a Latin adjective, referring to the type locality.

########### Description.

SIZE: Length 29–33 mm (male), 33–35 mm (female); width of midbody metazona ca. 2.2 mm (male), 2.8 mm (female). Width of head < collum = body ring 2 < 3 = 4 < 5–16, thereafter body gradually tapering towards telson.

COLOUR (Fig. 93A–D): In life with body pinkish brown; paraterga pink; antenna brownish black, except distal part of antennomere 7 and antennomere 8 whitish; collum and metaterga pinkish brown; head, surface below paraterga, sterna and epiproct brown; legs brownish black; a few basal podomeres pink.

ANTENNAE (Fig. 94D): Moderately long and slender, reaching to body ring 6 (male) and 4–5 (female) when stretched dorsally.

COLLUM (Fig. 94A): With 3 transverse rows of setiferous tubercles, 4(5)+4(5) anterior, 1+1 intermediate and 3(2)+3(2) posterior tubercles (lateral tubercles of anterior row located almost at base of paraterga in some specimens); paraterga of collum low, elevated at ca. 15°–20°, directed caudolaterad, with one conspicuous setiferous notch on lateral margin.

TEGUMENT: Quite dull, but slightly shining; collum and metaterga microgranulate; prozona finely shagreened; surface below paraterga finely microgranulate; sterna and epiproct somewhat smooth.

METATERGA (Fig. 94A–C): With 2 transverse rows of setiferous cones and spines; metaterga 2–8 with 2+2 anterior cones and 3(2)+3(2) posterior spines; metaterga 9–18 with 3+3 anterior cones and 4(3)+4(3) posterior spines; metatergum 19 with 3+3 anterior cones and 4+4 posterior cones.

PARATERGA (Fig. 94E, F): Directed caudolaterad on body rings 2–17, elevated at ca. 50° (male) 45° (female); directed increasingly caudad on body rings 18 and 19; anterior margin with 2 distinct notches, on lateral margin of body rings 9, 10, 12, 13, 15–18 with tiny denticle near the tip.

TELSON (Fig. 95C–G): Epiproct: tip subtruncate, lateral setiferous tubercles and apical tubercles inconspicuous. Hypoproct subtrapeziform; caudal margin round, with small but conspicuous setiferous tubercles.

STERNA (Fig. 96): Cross-impressions shallow. Sternal lobe between male coxae 4 trapeziform, tip truncate, thin in lateral view.

LEGS (Fig. 95H–J): Long and slender. Male femora 5 and 6 moderately humped ventrally in middle portion.

GONOPODS (Figs 97, 98): Coxa (cx) longer than prefemur. Cannula (ca) slender. Prefemur (pfe) ca. 2/3 as long as femur. Femur (fe) long and slender. Mesal sulcus (ms) and lateral sulcus (ls) conspicuous, deep. Postfemur (pof) conspicuous, ventrally wide. Solenophore (sph) well-developed: lamina lateralis (ll) swollen, broad; ventral lobe (vll) conspicuous, short and stout, digitiform: lamina medialis (lm) well-developed; process (plm) somewhat long, tube-like, tip usually terminating in three or two sharp small spines (in some specimens just one spine or tip blunt); distal lobe (dlm) distally with two lamellae, mesal lamella smaller and shorter than lateral one; broad lobe (blm) thick dorsally with ridges on edge. Solenomere (sl) relatively long.

########### Distribution and habitat.

Known only from the type locality in a limestone area (Fig. 93E). We assume that it is distributed in Kayin State. We made an intensive search for this species in September 2016 in Mawlamyine State (very close to Kayin State), however, no further specimens were found. Since it is so far known only from the type locality, *D. waepyanensis* sp. n. should be regarded as endemic to Myanmar.

########### Remarks.

The tip of process (plm) on lamina medialis is quite variable, having one, two, or three small spine(s), or being almost blunt in some specimens.

This species seems to be aposematic, to judge by the remarkable pink paraterga and pinkish brown body.

########### Coexisting species.

None known.

## Discussion

Our analyses of morphology, as well as our preliminary molecular phylogeny, supports the subdivision of *Desmoxytes* *s.l.* into five groups (*Desmoxytes* s.s., the ‘*acantherpestes*’group, the ‘*gigas*’group, the ‘*spiny*’group and *Hylomus*). All groups are clearly defined on morphological characters, especially of the gonopods and paraterga (Figs 2, 3). The distribution areas of the groups seem to be clear in their boundaries. The *Hylomus* group is more diverse in shape of paraterga, however, its members show a notable similarity in gonopod shape (Fig. 2E, F).

*Desmoxytes* s.s., the focus of the present paper, is well-defined based on gonopod characters especially the solenophore (lamina lateralis (ll) and lamina medialis (lm)). Species of *Desmoxytes* s.s. share several morphological similarities including wing-like paraterga; lamina lateralis (ll) swollen; lamina medialis (lm) comprised of process (plm), distal lobe (dlm) and broad lobe (blm); and the modification of male femora 5 and 6 only (exception: *D. terae*). Certain morphological characters show intra- and inter-population variations within the same species. The most variable characters within populations are:

– colour: variation seen in *D. cervina*, *D. delfae*, *D. takensis* and *D. euros* sp. n.

– tubercles/cones/spines on metaterga 9–19: number of tubercles/cones/spines sometimes decrease or increase in some rings – seen in *D. taurina*, *D. purpurosea*, *D. breviverpa*, *D. takensis*, *D. golovatchi* sp. n., *D. octoconigera* sp. n. and *D. waepyanensis* sp. n. This variable character is not significant for species identification.

– sternal lobe between male coxae 4: many species seem to be variable in the shape of the tip – seen in *D. planata*, *D. cervina*, *D. delfae*, *D. purpurosea*, *D. aurata* sp. n., *D. corythosaurus* sp. n. and *D. golovatchi* sp. n.

– process (plm) of lamina medialis: tip terminating in one or more spines – seen in *D. cervina*, *D. purpurosea* and *D. octoconigera* sp. n.: tip sharp or blunt – seen in *D. breviverpa*, *D. pinnasquali* and *D. takensis*.

Inter-population variation was also found in some species as follows:

– size: In *D. purpurosea*, specimens in the two main northern populations are clearly bigger than those from the two main southern populations. Specimens in a population of *D. planata* from Great Cocos Island seem to be smaller than others.

– colour: colour variation of living specimens of dragon millipedes is reported here for the first time. *D. cervina* includes brownish red as well as brown individuals. Specimens from the northern populations of *D. takensis* are red and those from southern populations are pink; however, the other morphological characters are identical.

Variation of colour and size within and between populations may at least in part be due to quality and quantity of food, differences in the physical environment (temperature, soil, humidity), like in other arthropods. Hagen (1881) and Buckton (1879) found that the colour of some arthropods was affected by nutrients and temperature. Many studies, e.g. Baker (1989) and Juliano (1986), have shown that food is one of the main factors controlling growth rate, body size, etc. in arthropods. For millipedes, there is the study by Berns and Keeton (1968) on *Narceus annularis* (order Spirobolida) showing that semi-starved individuals attained smaller body sizes than well-fed ones. David and Célérier (1997) showed that individuals of *Polydesmus angustus* kept on a diet of leaf litter plus yeast attained larger body sizes than individuals fed on leaf litter alone. On this background we assume that food and the physical environment may affect colour and size in *Desmoxytes*. Another possible factor controlling differences in colour and size might be the genetic variation within and between populations.

There are some species showing great resemblance in gonopod characters. In particular, *D. planata* and *D. euros* sp. n. are remarkable in having identical gonopods. Nevertheless, the yellow paraterga, shape of hypoproct and the initial study on mitochondrial COI gene supports to separate them as different species. The *D. planata-D. euros* sp. n. case reminds of what Pimvichai et al. (2011) found for *Thyropygus induratus* Attems, 1936 vs *T. quietus* Attems, 1938: a pair of species with virtually identical gonopods but significant genetic and non-gonopodal morphological differences.

*Desmoxytes* (and other dragon millipedes) are particularly attractive animals because of the peculiar paraterga, in combination with the unusual vivid colour in some species. The bright colour probably is a warning signal (Svadova et al. 2009, Shear 2015, Marek and Moore 2015). Many mating couples were found during our field surveys, and we collected some representative couples and reared a few specimens of *D. euros* sp. n. and *D. takensis* in acrylic boxes, feeding them with natural litter. We observed that the millipedes made moulting chambers using fecal material and silk as found in other polydesmidan families such as Polydesmidae and Pyrgodesmidae, as well as in the order Callipodida (Adis et al. 2000, Youngsteadt 2009, Reboleira and Enghoff 2016). We also observed a host-parasitic association between millipedes and mites in *D. cervina* (probably the mites belong in genus *Leptus* Latreille, 1976). We assume that the mite species is a parasite, like in other *Leptus* spp. (Southcott 1992) which use the millipede host for nourishment and dispersal purposes.

**Figure 99.**
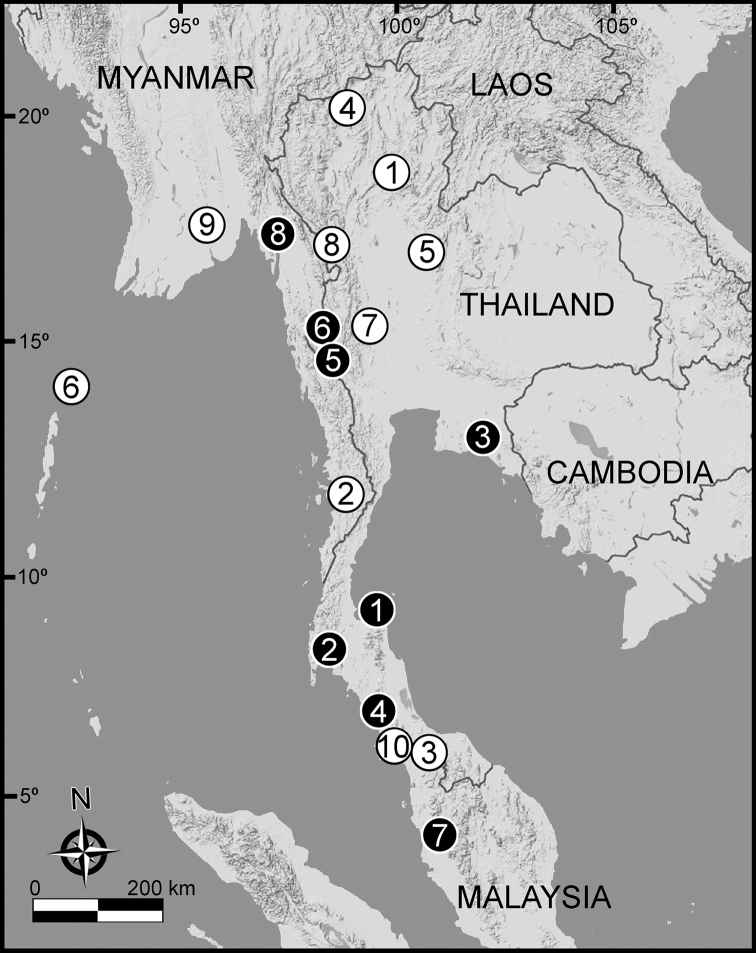
Type localities of all *Desmoxytes* species. white circle = described species (1 = *D. breviverpa*, 2 = *D. cervina*, 3 = *D. delfae*, 4 = *D. des*, 5 = *D. pinnasquali*, 6 = *D. planata*, 7 = *D. purpurosea*, 8 = *D. takensis*, 9 = *D. taurina*, 10 = *D. terae*). black circle = new species described in this study (1 = *D. aurata*, sp. n. 2 = *D. corythosaurus* sp. n., 3 = *D. euros* sp. n., 4 = *D. flabella* sp. n., 5 = *D. golovatchi* sp. n., 6 = *D. octoconigera* sp. n., 7 = *D. perakensis* sp. n., 8 = *D. waepyanensis* sp. n.).

**Figure 100.**
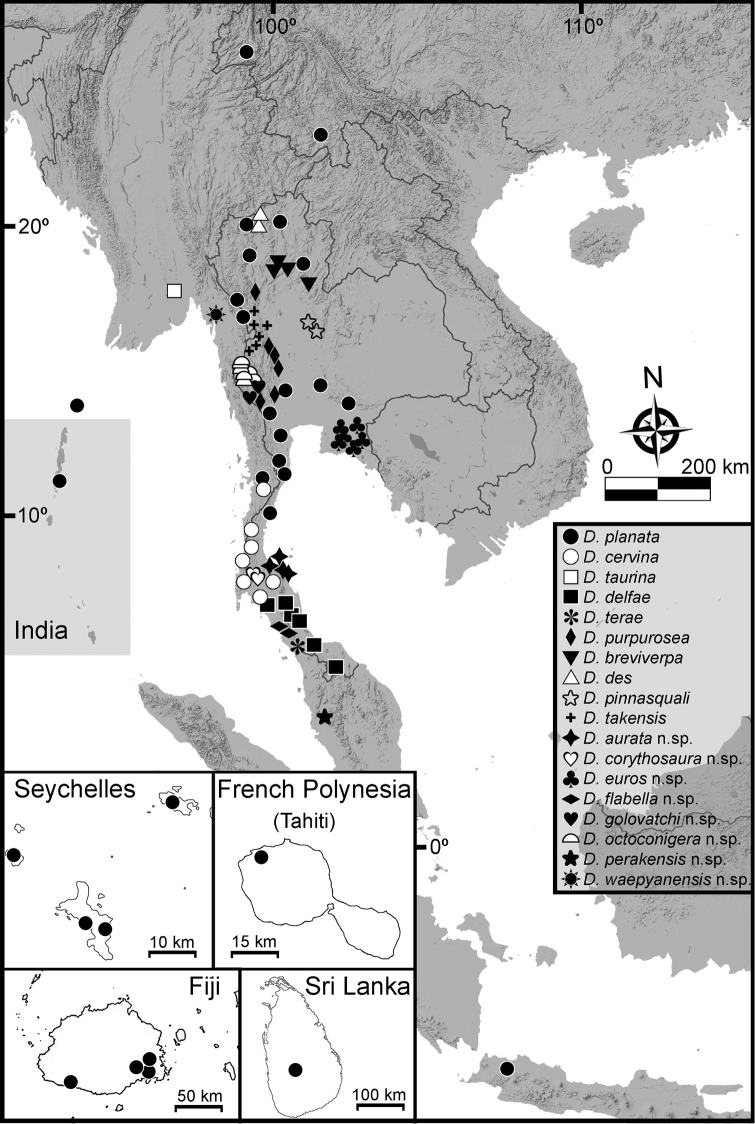
Known distribution of all *Desmoxytes* species based on all recorded data (*D. planata*, *D. cervina* and *D. delfae* are shown in the representative localities).

Figs 99 and 100 clearly show that all *Desmoxytes* species (except *D. planata*) are narrowly distributed, and all are restricted to limestone habitats or granitic mountains. The narrow distributional ranges of *Desmoxytes* species are perhaps the result of their poor dispersal capacities.

*Desmoxytes planata*, a pantropical species, has been recorded from widely scattered places. According to our survey we suspect that *D. planata* is probably originally native to Thailand or Myanmar. Especially in Thailand, we noticed that it ranges from Chiang Rai (northern end) to Chumphon Province (middle) (Fig. 100).

The species diversity of dragon millipedes is impressive. At the moment, Thailand, Malaysia, and Myanmar contain 18 species of *Desmoxytes*. We believe that many more new species remain to be discovered, especially in Thailand, Myanmar, Malaysia, Cambodia, and Laos.

## Supplementary Material

XML Treatment for Hylomus

XML Treatment for Desmoxytes

XML Treatment for Desmoxytes aurata

XML Treatment for Desmoxytes breviverpa

XML Treatment for Desmoxytes cervina

XML Treatment for Desmoxytes corythosaurus

XML Treatment for Desmoxytes delfae

XML Treatment for Desmoxytes des

XML Treatment for Desmoxytes euros

XML Treatment for Desmoxytes flabella

XML Treatment for Desmoxytes golovatchi

XML Treatment for Desmoxytes octoconigera

XML Treatment for Desmoxytes perakensis

XML Treatment for Desmoxytes pinnasquali

XML Treatment for Desmoxytes planata

XML Treatment for Desmoxytes purpurosea

XML Treatment for Desmoxytes takensis

XML Treatment for Desmoxytes taurina

XML Treatment for Desmoxytes terae

XML Treatment for Desmoxytes waepyanensis
